# Benefits and Harms of Digital Health Interventions Promoting Physical Activity in People With Chronic Conditions: Systematic Review and Meta-Analysis

**DOI:** 10.2196/46439

**Published:** 2023-07-06

**Authors:** Graziella Zangger, Alessio Bricca, Behnam Liaghat, Carsten B Juhl, Sofie Rath Mortensen, Rune Martens Andersen, Camma Damsted, Trine Grønbek Hamborg, Mathias Ried-Larsen, Lars Hermann Tang, Lau Caspar Thygesen, Søren T Skou

**Affiliations:** 1 The Research Unit PROgrez Department of Physiotherapy and Occupational Therapy Næstved-Slagelse-Ringsted Hospital Slagelse Denmark; 2 Research Unit for Musculoskeletal Function and Physiotherapy Department of Sports Science and Clinical Biomechanics University of Southern Denmark Odense Denmark; 3 Centre for Evidence-Based Orthopedics (CEBO) Department of Orthopaedic Surgery Zealand University Hospital Køge Denmark; 4 Department of Physiotherapy and Occupational Therapy University Hospital of Copenhagen Herlev and Gentofte Denmark; 5 Research Unit for Exercise Epidemiology Centre of Research in Childhood Health Department of Sports Science and Clinical Biomechanics, University of Southern Denmark Odense Denmark; 6 Department of Regional Health Research University of Southern Denmark Odense Denmark; 7 Centre for Physical Activity Research Copenhagen University Hospital Rigshospitalet Copenhagen Denmark; 8 National Institute of Public Health University of Southern Denmark Copenhagen Denmark

**Keywords:** digital health, eHealth, mobile health, mHealth, wearables, physical activity, physical function, chronic conditions, randomized controlled trials, systematic review, meta-analysis

## Abstract

**Background:**

Digital health interventions for managing chronic conditions have great potential. However, the benefits and harms are still unclear.

**Objective:**

This systematic review and meta-analysis aimed to investigate the benefits and harms of digital health interventions in promoting physical activity in people with chronic conditions.

**Methods:**

We searched the MEDLINE, Embase, CINAHL, and Cochrane Central Register of Controlled Trials databases from inception to October 2022. Eligible randomized controlled trials were included if they used a digital component in physical activity promotion in adults with ≥1 of the following conditions: depression or anxiety, ischemic heart disease or heart failure, chronic obstructive pulmonary disease, knee or hip osteoarthritis, hypertension, or type 2 diabetes. The primary outcomes were objectively measured physical activity and physical function (eg, walk or step tests). We used a random effects model (restricted maximum likelihood) for meta-analyses and meta-regression analyses to assess the impact of study-level covariates. The risk of bias was assessed using the Cochrane Risk of Bias 2 tool, and the certainty of the evidence was assessed using the Grading of Recommendations Assessment, Development, and Evaluation.

**Results:**

Of 14,078 hits, 130 randomized controlled trials were included. Compared with usual care or minimal intervention, digital health interventions increased objectively measured physical activity (end of intervention: standardized mean difference [SMD] 0.29, 95% CI 0.21-0.37; follow-up: SMD 0.17, 95% CI 0.04-0.31) and physical function (end of intervention: SMD 0.36, 95% CI 0.12-0.59; follow-up: SMD 0.29, 95% CI 0.01-0.57). The secondary outcomes also favored the digital health interventions for subjectively measured physical activity and physical function, depression, anxiety, and health-related quality of life at the end of the intervention but only subjectively measured physical activity at follow-up. The risk of nonserious adverse events, but not serious adverse events, was higher in the digital health interventions at the end of the intervention, but no difference was seen at follow-up.

**Conclusions:**

Digital health interventions improved physical activity and physical function across various chronic conditions. Effects on depression, anxiety, and health-related quality of life were only observed at the end of the intervention. The risk of nonserious adverse events is present during the intervention, which should be addressed. Future studies should focus on better reporting, comparing the effects of different digital health solutions, and investigating how intervention effects are sustained beyond the end of the intervention.

**Trial Registration:**

PROSPERO CRD42020189028; https://www.crd.york.ac.uk/prospero/display_record.php?RecordID=189028

## Introduction

### Background

Chronic conditions are the most significant contributors to the global burden of disease, which is continuously increasing [1]. Living with ≥1 chronic conditions significantly impacts a person’s life, family, and society [1]. Physical inactivity is a major risk factor for at least 35 chronic conditions, which has become a global health concern [2]. Physical inactivity is expensive, constituting up to 4.6% of a country’s national health care expenditure [3]. In contrast, being physically active can benefit physical and psychosocial health [4]. In fact, following a structured exercise program can reduce the symptoms of chronic conditions with no additional risk of serious adverse events [5]. However, a high proportion of people living with chronic conditions do not meet the recommended levels of physical activity [6,7], possibly because of various reasons such as lack of access; travel distance; time inconvenience; cost; or internal factors such as pain, lack of motivation, or reluctance to engage in group activities [8].

Regarding physical activity interventions, it is essential to understand and adapt care according to the individual’s needs, preferences, and abilities [9]. One way to deliver and make physical activity available, individually or as an add-on, is through digital health interventions [10]. Digital health involves multiple solutions, including telephone calls, SMS text messages, mobile apps, websites, videoconferencing, and wearables (eg, activity trackers). The potential advantages of digital health solutions are convenience in terms of reduced distance, time, and cost, thereby limiting some of the more traditional barriers to physical encounters [10]. Digital health solutions enable personalized and person-centered tailored health interventions. Such personalized care empowers people to manage their health, increases health care efficiency, and reduces health care costs [11]. Consequently, the focus on digital health interventions has steadily grown over the last 2 decades [12], accelerating further in response to the COVID-19 pandemic [13]. This has led to digital health interventions being initiated without fully knowing their effect, potentially introducing people to ineffective interventions [14].

Several systematic reviews have investigated the effect of digital health interventions on health outcomes in people with individual chronic conditions (eg, chronic obstructive pulmonary disease [COPD], heart disease, and diabetes) [15-26]. Some reviews have focused on physical activity [19-21,23,25,27], but these included few studies, as they focused on a single condition, solution (eg, only telephone calls or apps), or outcome measure (eg, pain). This challenges the assessment of the benefits and harms of digital health intervention. Furthermore, the proportion of people with >1 chronic condition is increasing. More than half of the people with chronic conditions have at least 2 conditions [28]; therefore, to increase the power of the results and better resemble real-life clinical practice, including studies on several chronic conditions is relevant. Therefore, a more comprehensive approach is needed to provide clinicians and decision makers with the best current evidence.

### Objectives

With this systematic review and meta-analysis, we investigated the benefits and harms of digital health interventions promoting physical activity among people with ≥1 chronic conditions on objectively and subjectively measured physical activity and physical function, depression, anxiety, health-related quality of life (HRQOL), and nonserious and serious adverse events.

## Methods

### Overview

The study was preregistered at PROSPERO (CRD42020189028) and the study protocol (including amendments) is available in Multimedia Appendix 1 [4,26,29-44]. We followed the Cochrane Handbook for Systematic Reviews [45] and reported according to the PRISMA (Preferred Reporting Items for Systematic Reviews and Meta-Analyses) guidelines [46]. The PRISMA checklist is available in Multimedia Appendix 2 [46].

### Ethical Considerations

As this research design only investigates aggregated data from published randomized controlled trials (RCTs) with no individual data, no ethics approval was required.

### Eligibility Criteria

We included all peer-reviewed RCTs that compared an intervention with a digital health component in promoting physical activity to usual care or minimal intervention (eg, education or blinded wearables). Digital health interventions were defined as interventions in which any solution or technology (eg, website, app, and wearable) was used to deliver health information between health care providers and participants over a distance [47]. Studies were included if (1) physical activity (any activity that was up to the participants to choose), exercise (any prescribed activity), or exercise therapy (any planned and structured prescribed activity) was used or promoted; (2) the primary or secondary aim was physical activity promotion; and (3) digital health intervention was delivered with or without additional pharmacotherapy or other nondigital adjuvant interventions (eg, rehabilitation, education, or counseling).

Moreover, studies had to include adults (aged ≥18 years) with ≥1 of the following chronic conditions: depression or anxiety (mental health condition), ischemic heart disease or heart failure (heart disease), COPD, osteoarthritis of the hip or knee, hypertension, or type 2 diabetes. The inclusion criteria focused on these specific conditions, as they are among the most common worldwide according to the global burden of disease [1]. Furthermore, they share the risk factors of physical inactivity and systemic low-grade inflammation, which can cause a chain of events that can lead to worse outcomes and other chronic conditions [4,48].

Studies were excluded if they included angina, valvular heart disease, congenital heart disease, cardiomyopathy, hypertension if systolic blood pressure ≤139 mm HG or diastolic blood pressure ≤89 mm Hg, mixed populations of type 1 and 2 diabetes, tested the same digital health intervention but in different settings, or used the digital health solution for monitoring without any interaction with or feedback to the participants (ie, electronic health records or passive use of telemonitoring or a blinded actigraphy device).

### Information Sources and Search Strategy

The MEDLINE, Embase, CINAHL, and Cochrane Central Register of Controlled Trials databases were searched via Ovid from inception to October 28, 2022, with no language restrictions. The search string was developed based on previous searches used in systematic reviews for people with chronic conditions [49,50], and validated RCT search filters were used if available and applicable in each database [51]. All search strings are presented in Multimedia Appendix 3 [52-191]. In addition, studies found during full-text screens were manually added.

### Selection and Data Collection Process

Initially, search duplicates were removed using the reference manager tool EndNote (version 20; Clarivate) [192] and the management tool Covidence (Veritas Health Innovation;) [193]. In total, 5 authors (AB, NN, FD, AL, and GZ) independently screened titles and abstracts, and 2 authors (AB and GZ) independently screened the full texts. Discrepancies were solved during regular meetings and resolved by discussing with a third study team member (CBJ). Although no restrictions were applied to the search, we only included studies if available in 1 of the following languages: English, Danish, Norwegian, Swedish, or Italian. Covidence was used throughout the selection process.

A standardized data extraction form (piloted in 5 studies) was used. A total of 4 authors (AB, BL, NN, and GZ; in pairs) independently extracted data at two time points: (1) immediately after the end of the intervention and (2) at follow-up, the time point closest to 12-month follow-up. Discrepancies were resolved by consensus or by referring to a third author (CBJ). WebPlotDigitizer (version 4.5; Ankit Rohatgi) was used to extract data from the figures [194]. The authors were contacted if the studies had missing or unclear information or data [195]. If the authors did not respond, the study eligibility was reassessed and consequently excluded.

### Outcomes

Outcomes were hierarchically ordered (Table 1), with objectively measured outcomes prioritized over subjective and generic tests over disease-specific tests. The primary outcomes were objectively measured physical activity and physical function. The secondary outcomes were subjectively measured physical activity and physical function, depression, anxiety, HRQOL, and adverse events (nonserious and serious).

**Table 1:** Outcome hierarchy.

Outcome			Hierarchy
**Primary outcomes**			
	Objectively measured physical activity	Accelerometer measures (eg, daily time spent in moderate to vigorous physical activity) Pedometer (eg, outcomes such as step counts) Any other outcome measure related to objectively measured physical activity	
	Objectively measured physical function	The 6-minute walk test Incremental shuttle walk test Any other outcome measure related to daily function (eg, Chair stand test)	
**Secondary outcomes**			
	Subjectively measured physical activity	The Global Physical Activity Questionnaire The Physical Activity Scale for the Elderly questionnaire The International Physical Activity Questionnaires, long form or short form Any other outcome measure related to subjectively measured physical activity	
	Subjectively measured physical function	The 36-item Short-Form Health Survey, as the Physical Function subscale or the Role Function subscale Any other self-reported measure of physical function	
	Depression	The Beck Depression Inventory Any other depression questionnaire (eg, HADS^a^ depression subscale) Any other assessment of depression (eg, clinical assessment)	
	Anxiety	State-Trait Anxiety Inventory questionnaire Any other anxiety questionnaire (eg, HADS anxiety) Any other assessment of anxiety (eg, clinical assessment)	
	Health-related quality of life	The EQ-5D questionnaire Any other generic health-related quality-of-life questionnaires Disease-specific health-related quality-of-life questionnaires (eg, the Minnesota Living with Heart Failure questionnaire)	
	Adverse events (nonserious and serious)	Extracted if reported in the included trials accordingly to the FDA^b^ recommendations of nonserious and serious adverse events [29]. Adverse events were defined accordingly to FDA as “any unfavorable and unintended sign, symptom or disease temporally associated with the use of a medicinal [investigational] product whether or not considered related to the medicinal [investigational] product and grouped in serious adverse events such as death, hospitalization, disability or permanent damage, and nonserious adverse events such as pain, falls and fatigue” [29].	

^a^HADS: Hospital Anxiety and Depression Scale.

^b^FDA: Food and Drug Administration.

### Data Extraction

Extracted data included characteristics of the (1) trial—study design, study authors, publication year, country, and setting; (2) participants—number randomized, age, sex, BMI, socioeconomic status, and index conditions (if a study included participants based on them having ≥2 chronic conditions, the study was categorized as multimorbidity); and (3) intervention—type, frequency, duration, delivery method (categorized as mobile health [mHealth; ie, telephone calls, SMS text messages, and apps], eHealth [eg, internet based, website, and emails], or digital device [eg, wearables and Bluetooth-connected devices]), type of comparator, physical activity (categorized as physical activity [any activity that was up to the participants to choose] or exercise therapy [any planned and structured prescribed activity]), phase (categorized as without, with, or after any other rehabilitation), use of theory or framework, intervention components (categorized as digital only or combined in-person and digital [ie, other in-person adjuvant intervention, as rehabilitation, education, or counseling]), and adherence.

### Risk of Bias Assessment and Certainty Assessment

The risk of bias was assessed for each outcome in pairs by 7 authors (CD, SRM, TGH, RMA, LHT, GZ, AB) independently using the Cochrane Risk of Bias 2 tool [30]. Disagreements were resolved by reaching a consensus or by including a third author (GZ or AB). The quality of evidence of each outcome was assessed using the Grading of Recommendations Assessment, Development, and Evaluation approach by 2 authors (GZ and AB) [31]. An agreement was reached through consensus.

### Synthesis of Results

Meta-analysis was conducted using a random effects model, adjusted to Hedges *g* and using a restricted maximum likelihood method, as heterogeneity was expected. The effect of the intervention on continuous outcomes was expressed as standardized mean difference (SMD) for outcomes reported with different measures and with 95% CIs. The mean differences with 95% CIs were calculated for data on the same metric scale. Risk ratios were calculated with 95% CIs for dichotomous outcomes (ie, adverse events). Meta-analyses were visually presented as forest plots and were performed using the *meta* command in Stata (version 17.0; StataCorp LLC) [196]. The pooled SMD effect sizes were interpreted as 0.20, 0.50, and 0.80, representing small, moderate, and large effects, respectively [32]. The inconsistency of the results was evaluated using the *I*^2^ and adjusted *R*^2^ statistics and by visual inspection of the bubble plots and meta-regressions [52].

### Meta-Regression and Subgroup Analyses

Univariable and multivariable meta-regression analyses were performed if ≥10 studies were included in the analyses to identify factors that could influence the effect estimates. For each outcome at the end of the intervention, the impact of (1) participant characteristics—age, sex, BMI, socioeconomic status, depression, and anxiety levels—and (2) intervention characteristics—number of digital and in-person sessions, frequency, duration, type of physical activity, phase, use of theory or framework, adherence, digital only, other in-person adjuvant intervention, and financial incentive—were assessed. Meta-regression analyses were performed using the *meta regress* command in Stata version 17.0 [196].

Subgroup analyses investigated whether the effects of digital health interventions varied among the included index conditions on all outcomes. Moreover, the delivery method and methodological quality according to the Cochrane Risk of Bias 2 tool (high, some concern, or low risk of bias) were assessed by subgroups for the primary outcomes.

### Reporting Bias Assessment

Small study bias was assessed using funnel plots and Egger test (or Harbord test for risk ratios) by 2 authors (GZ and AB), and agreement was reached by consensus.

## Results

### Study Selection and Characteristics

**Figure 1:**
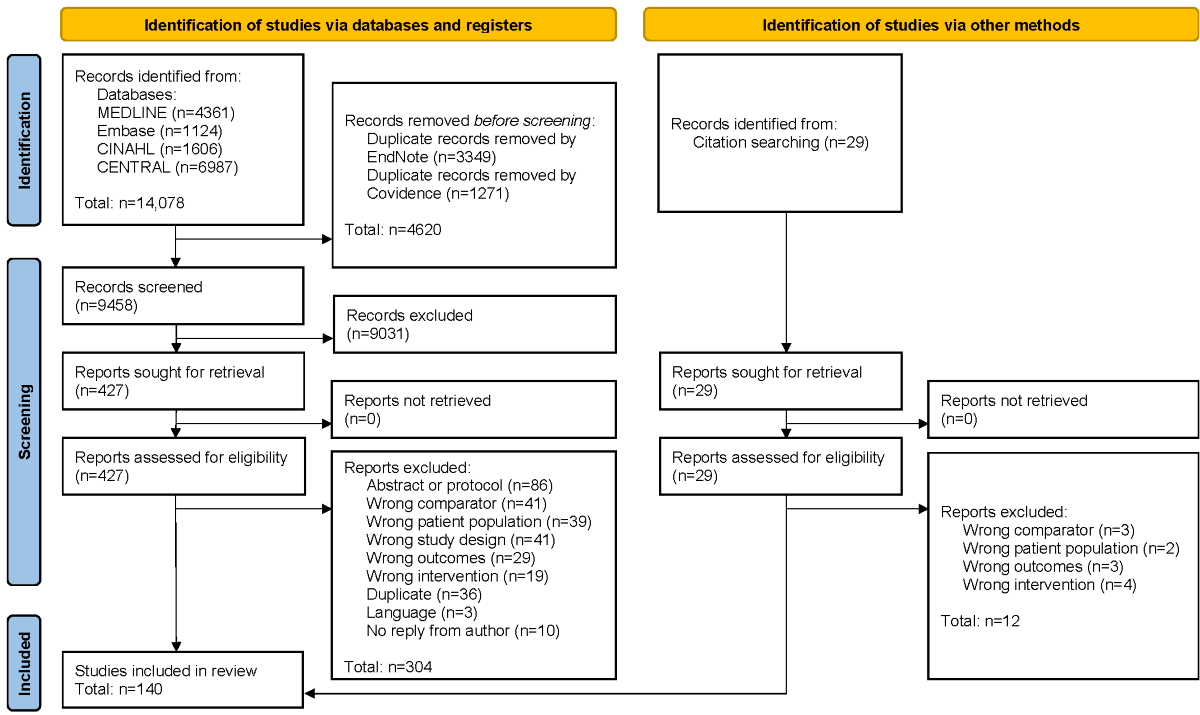
PRISMA (Preferred Reporting Items for Systematic Reviews and Meta-Analyses) flowchart of the study selection process.

The search retrieved 14,078 hits; 427 papers were full-text screened, and 140 papers were included, representing 130 RCTs (see Figure 1 for flowchart and refer to Multimedia Appendix 3 for the list of excluded studies). The included RCTs allowed for 136 comparisons, representing 20,094 participants (mean age 60.68, SD 7.17 years; 8440/20,094, 41.63% female). The interventions were conducted in 31 countries, mainly in the United States (24 RCTs) [53-75] and Australia (21 RCTs) [76-96]. Over half (91/140, 65%) of the RCTs were published between 2018 and 2022; however, the publication years ranged from 2003 to 2022. For study characteristics, refer to Multimedia Appendix 3.

### Intervention Components

In total, 80 RCTs used a digital-only approach [53,54,56,59-62,65,66,68-74,76,79,80,83,85,86,88-93,95-144], whereas 56 RCTs combined digital solutions with nondigital interventions (eg, counseling or in-person guided exercise) [55,57,58,63,64,66,67,75,77,78,81,82,84,87,94,142,145-188]. Of the included studies, 7 offered a preintervention period as run-in exercise (a period before randomization and intervention start where all participants performed exercise) or rehabilitation [63,98,108,111,120,160,183,189]. Furthermore, 13 RCTs offered rehabilitation as part of the intervention [64,75,80,82,147,149,151,152,157,160,163,169,178].

mHealth solutions (eg, telephone calls, text messages, and apps) were primarily used in 56 RCTs [53,56,57,59,63,67,69,78-80,​^83^-^86^,^90^,^92^,^93^,^96^,^99^,^101^,^102^,^105^,^108^,^110^,^112^-^115^,^119^,​^120^,^124^,^128^-^130^,^134^,^136^,^140^-^142^,^146^-^148^,^150^,^155^,^157^,^161^,^163^,​^164^,^167^,^168^,^172^,^176^,^177^,^179^,^181^,^185^], whereas 22 RCTs [58,60,61,65,70,81,87,111,116,117,126,133,134,145,149,151,154,156,​^165^,^171^,^173^,^174^] used mHealth combined with a device (eg, wearable, pedometer, activity tracker, or Bluetooth-connected pulse oximeter or glycosometer). Furthermore, 6 RCTs [75,89,118,158,169,183] combined mHealth and eHealth (eg, websites, web apps, and emails) technology. eHealth solutions were used by 29 RCTs [54,55,66,72-74,76,77,88,91,95,​^97^,^100^,^103^,^104^,^106^,^122^,^125^,^127^,^131^,^132^,^138^,^143^,^144^,^152^,^159^,^175^,^184^,^190^], whereas 6 RCTs [64,71,82,98,137,162,189] combined eHealth and a device. Digital devices alone were used in 14 RCTs [62,68,94,107,109,121,123,130,139,166,170,178,182,186,188,191]. Finally, 2 studies combined mHealth, eHealth, and a digital device [132,160].

The RCTs had a mean intervention duration of 22.94 (SD 18.72; range 4-104) weeks, and 60.9% (81/136) RCTs were placed in the physical activity category. A theory or framework most often (71/136, 52.6%) guided the interventions. The mean number of digital sessions was 97.98 (SD 109.29; range 2-377), whereas in-person sessions had a mean of 3.26 (SD 2.93; range 0-18). Of the included RCTs, 31 [54,58,62,65,70,76,​^77^,^82^,^84^,^86^,^88^,^92^,^94^,^95^,^100^,^101^,^112^,^121^,^132^,^135^,^143^,​^144^,^146^,^150^,^151^,^155^,^163^,^164^,^174^,^182^,^186^,^189^-^191^,^197^] had follow-up data, and the mean follow-up time was 45.38 (SD 21.74; range 12-104) weeks.

### Chronic Conditions

Type 2 diabetes [53,56,58,59,66,67,81,84,88,92,93,97,99,104,​^107^,^114^-^116^,^119^,^122^,^123^,^126^-^128^,^130^,^131^,^140^,^145^,^146^,^155^,^157^,^159^,^165^,^168^,​^172^,^182^,^184^] and heart disease [62,64,65,70,72,73,75,80,85,87,​^96^,^98^,^102^,^106^,^109^,^111^-^113^,^121^,^132^-^135^,^139^,^142^,^147^,^154^,^160^,^161^,^169^,^171^,​^173^-^181^,^185^,^189^] were the most frequent index conditions (41 RCTs each), followed by COPD (26 RCTs) [60,61,63,68,71,74,79,82,91,94,105,108,117,120,124,​^136^,^141^,^149^,^151^-^153^,^156^,^163^,^164^,^167^,^170^,^183^,^186^,^190^,^191^], osteoarthritis (18 RCTs) [54,55,57,76-78,86,89,90,100,110,​^118^,^129^,^138^,^148^,^158^,^166^,^188^], mental health (5 RCTs) [95,125,137,143,144,162,198], hypertension (3 RCTs) [83,101,103,197], and multimorbidity (2 RCTs) [69,150]. For the index conditions and comorbidities among the participants, refer to Multimedia Appendix 3.

### Effect on Primary Outcomes at the End of the Intervention

**Figure 2:**
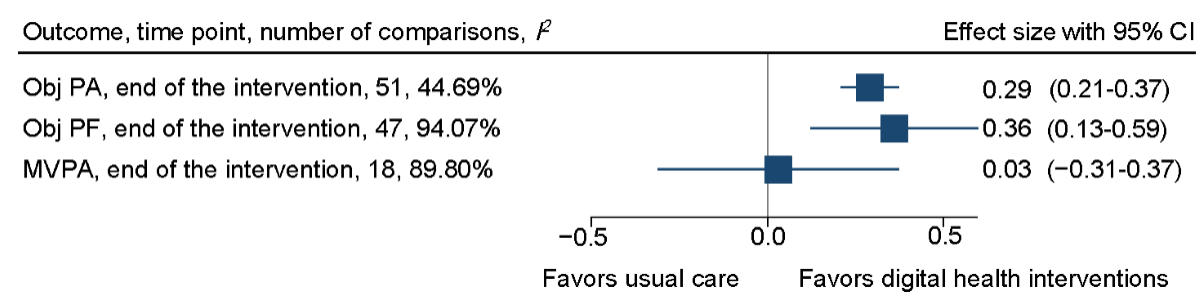
Overall forest plot of objectively measured physical activity, objectively measured physical function, and moderate to vigorous physical activity (MVPA) at the end of the intervention. Obj PA: objectively measured physical activity; Obj PF: objectively measured physical function.

At the end of the intervention, the digital health interventions, compared with usual care or minimal intervention, showed a small improvement in objectively measured physical activity SMD 0.29 (95% CI 0.21-0.37), corresponding to an average increase of 970.79 daily steps (95% CI 657.11-1284.47; *I*^2^=54.04%). Objectively measured physical function also showed a small improvement in favor of the digital health interventions (SMD 0.36, 95% CI 0.13-0.59) consistent with an improvement of 19.82 m (95% CI 9.45-30.02; *I*^2^=75.94%) on the 6-minute walk test (6MWT). There was no between-group difference in objectively measured moderate to vigorous physical activity (MVPA; SMD 0.03, 95% CI −0.31 to 0.37). However, objectively measured physical function and MVPA showed substantial heterogeneity. See Figure 2 for the overall forest plot and refer to Multimedia Appendix 4 [52-191] for a detailed forest plot of each outcome, including daily steps and 6MWT.

### Effect on Secondary Outcomes at the End of the Intervention

**Figure 3:**
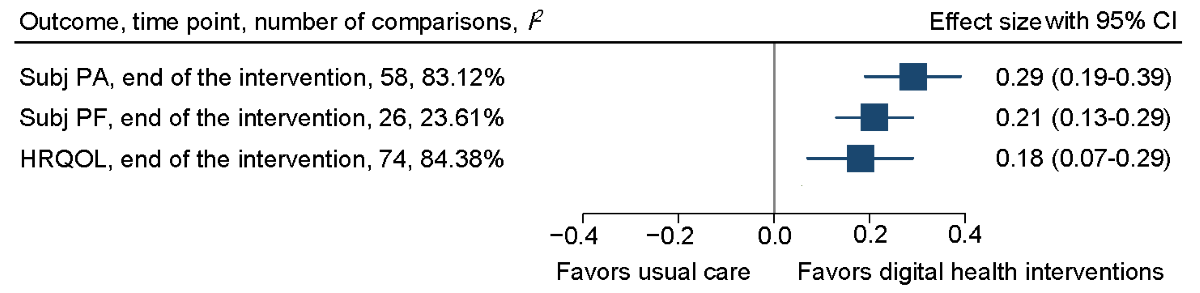
Overall forest plot of subjectively measured physical activity, physical function, and health-related quality of life (HRQOL) at the end of the intervention. Subj PA: subjectively measured physical activity; Subj PF: subjectively measured physical function.

**Figure 4:**
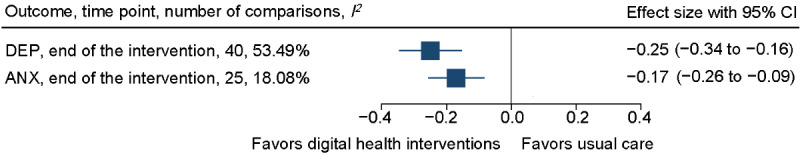
Overall forest plot of depression and anxiety at the end of the intervention. ANX: anxiety; DEP: depression.

End-of-intervention effects were found in favor of the digital health interventions for subjectively measured physical activity and physical function, depression, anxiety, and HRQOL. Subjectively measured physical activity and HRQOL showed substantial heterogeneity (Figures 3 and 4; Multimedia Appendix 4).

There was an increased risk of nonserious adverse events (eg, musculoskeletal problems, soreness, or pain) in the intervention groups (risk ratio 1.31, 95% CI 1.11-1.55), whereas the risk of serious adverse events did not differ (risk ratio 0.89, 95% CI 0.76 to 1.04; Multimedia Appendix 4).

### Effect on Primary Outcomes at Follow-Up

**Figure 5:**
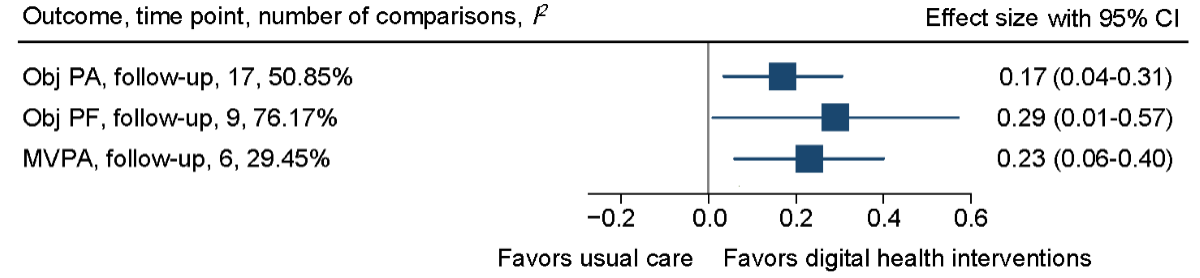
Overall forest plot of objectively measured physical activity, physical function, and moderate to vigorous physical activity (MVPA) at follow-up. Obj PA: objectively measured physical activity; Obj PF: objectively measured physical function.

At follow-up, objectively measured physical activity showed a small improvement (SMD 0.17, 95% CI 0.04-0.31; *I*^2^=50.85%) in favor of the digital health intervention groups but with no between-group difference in daily steps (mean daily steps 498.40, 95% CI −44.99 to 1041.80; *I*^2^=77.94%). For objectively measured physical function, a small improvement was found in favor of digital health interventions (SMD 0.29, 95% CI 0.01-0.57; *I*^2^=76.17%). The improvement in 6MWT was sustained (50.06 m, 95% CI 15.1-85.01; *I^2^*=71.28%). However, a small effect on objectively measured MVPA in favor of the intervention group was found at follow-up (SMD 0.23, 95% CI 0.06-.40, *I*^2^=29.45%). See Figure 5 for the overall forest plot and refer to Multimedia Appendix 4 for a detailed forest plot of each outcome, including daily steps and 6MWT.

### Effect on Secondary Outcomes at Follow-Up

**Figure 6:**
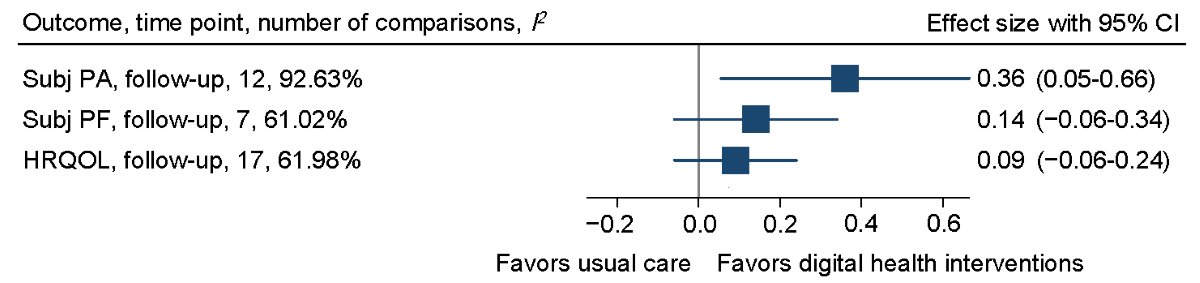
Overall forest plot of subjectively measured physical activity, physical function, and health-related quality of life (HRQOL) at follow-up. Subj PA: subjectively measured physical activity; Subj PF: subjectively measured physical function.

**Figure 7:**
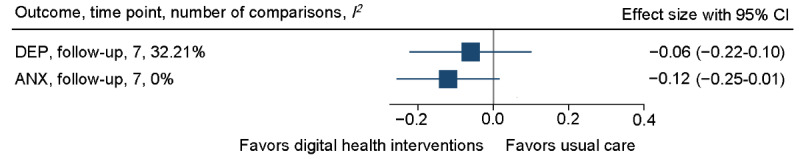
Overall forest plot of depression and anxiety at follow-up. ANX: anxiety; DEP: depression.

Only the effect on subjectively measured physical activity was sustained at the follow-up of the secondary outcomes (SMD 0.36, 95% CI 0.05-0.66; Figures 6 and 7; Multimedia Appendix 4). However, in the follow-up period, neither nonserious (risk ratio 1.35, 95% CI 0.71-2.54) nor serious adverse events (risk ratio 0.76, 95% CI 0.56-1.03) showed any between-group differences (Multimedia Appendix 4).

### Risk of Bias

**Figure 8:**
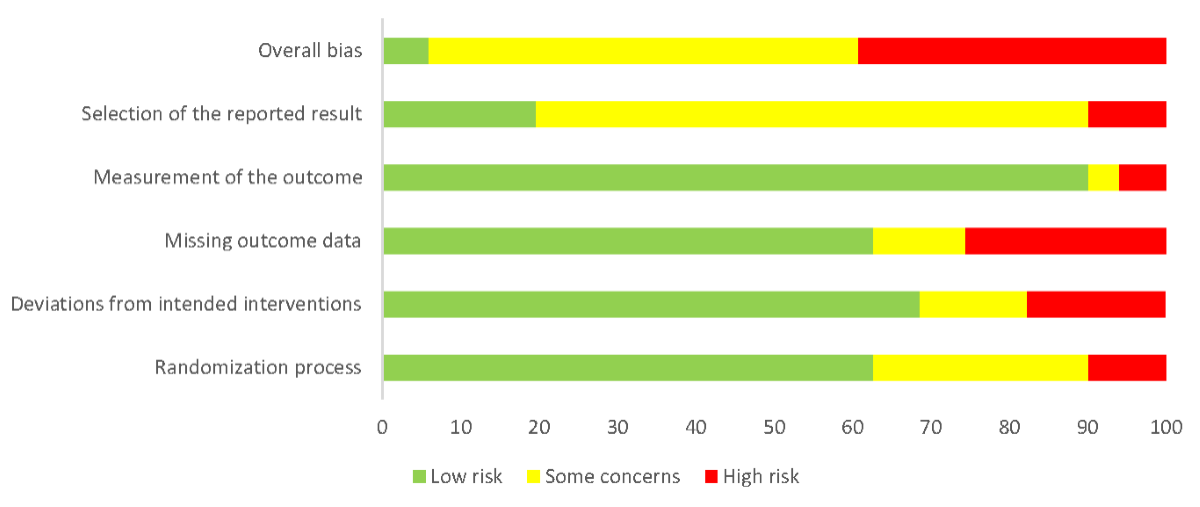
Risk of bias for objectively measured physical activity given as percentages.

**Figure 9:**
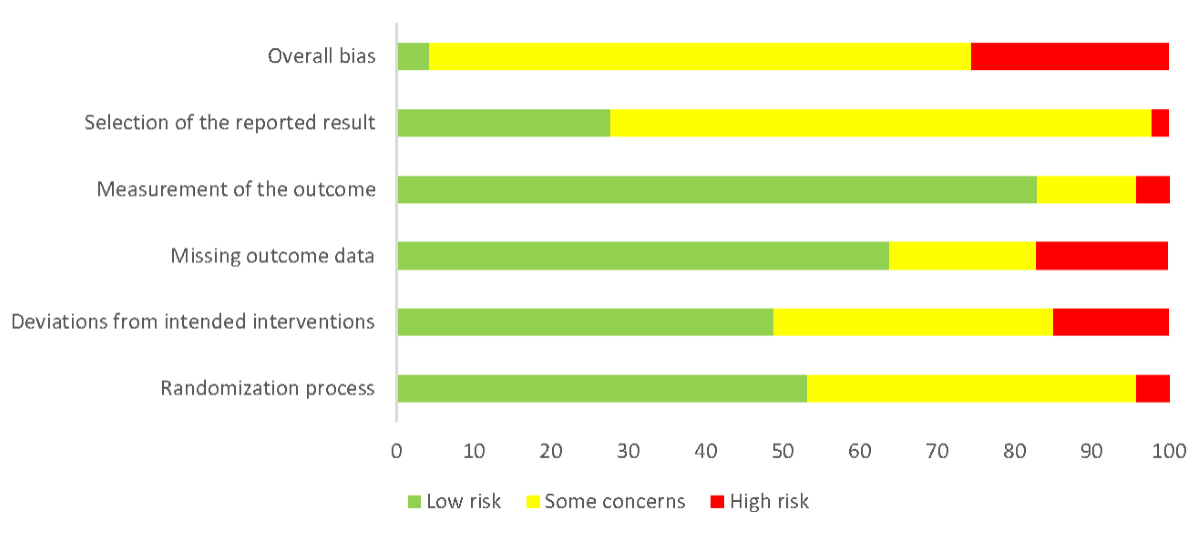
Risk of bias with objectively measured physical function given as percentages.

For objectively measured physical activity, 6% (3/51) of the RCTs were classified as low risk of bias [58,84,160], 55% (28/51) of the RCTs had some concerns [53,61,​^62^,^68^,^70^,^71^,^74^,^81^,^82^,^95^,^97^,^98^,^100^,^102^,^107^,^111^,^115^,​^117^,^136^,^143^,^144^,^147^,^148^,^155^,^156^,^166^,^180^,^182^,^183^,^188^-^191^], and 39% (20/51) of the RCTs were classified as high risk of bias [60,65,69,81,91,94,99,104,125,130-132,145,146,149,151,​^152^,^163^,^164^,^186^] (Figure 8). For objectively measured physical function, 4% (2/47) of the RCTs were classified as low risk of bias [110,158], 70% (33/47) of the RCTs had some concerns [54,55,64,71,74,79,82,87,94,96,97,102,105,108,117,​^118^,^120^,^121^,^124^,^133^,^136^,^147^,^156^,^159^,^167^,^171^,^175^,^178^,^183^,​^185^,^186^,^190^], and 26% (12/47) of the RCTs were classified as high risk of bias [57,63,91,112,126,149,151-154,163,164,​^170^,^176^] (Figure 9). The risk of bias profiles were similar for the secondary outcomes (Multimedia Appendix 5 [52-191]).

Small study bias was judged as present at the end of the intervention for objectively measured physical function, subjectively measured physical activity, and HRQOL and at follow-up for subjectively measured physical activity (Multimedia Appendix 6 [52-191]).

### Certainty of Evidence

The Grading of Recommendations Assessment, Development, and Evaluation assessment scores ranged from moderate to very low (Table 2). Downgrading was done owing to study limitations, imprecision of the findings (some conditions had a limited number of RCTs), and inconsistency (high statistical heterogeneity only partially explained by the meta-regression analyses).

**Table 2:** Grading of Recommendations Assessment, Development, and Evaluation summary of findings.

Outcome; time point	Number of participants (studies)	Relative effect (95% CI)	Anticipated absolute effects				Certainty		What happens
			Without digital health interventions, %	With digital health interventions (95% CI)	Difference				
Objectively measured physical activity; end of intervention: mean 20.7 (SD 16.6) weeks	6207 (51 RCTs^a^)	N/A^b^	N/A	N/A	SMD^c^ 0.29 SD higher (0.21 higher to 0.37 higher)	⊕⊕⊕○ Moderate^d,^^e^		Digital health interventions that promote physical activity likely slightly increase objectively measured physical activity. This may correspond to an average increase of 970.8 daily steps (95% CI 657.1 1284.5 steps; *I*^2^=54.0%; 30 RCTs; 3904 participants). However, there was no effect on objectively measured moderate to vigorous physical activity (SMD 0.03, 95% CI −0.31 to 0.37; *I*^2^=89.8%; 18 RCTs; 1921 participants).	
Objectively measured physical function; end of intervention: mean 17.5 (SD 18.0) weeks	6056 (47 RCTs)	N/A	N/A	N/A	SMD 0.36 SD higher (0.12 higher to 0.59 higher)	⊕○○○ Very low^f,g^^,^^h,i^		Digital health interventions promoting physical activity may slightly increase objectively measured physical function, but the evidence is very uncertain. However, at the end of the intervention (mean 16.8 weeks), the effect may correspond to an average increase of 19.8 m (95% CI 9.5-30.0 m; *I*^2^=75.9%; 34 RCTs; 4725 participants) on the 6-minute walk test.	
Subjectively measured physical activity; end of intervention: mean 27.3 (SD 21.4)	10,906 (58 RCTs)	N/A	N/A	N/A	SMD 0.29 SD higher (0.19 higher to 0.39 higher)	⊕○○○ Very low^d,f^^,^^g,i,j^		Digital health interventions promoting physical activity may slightly increase subjectively measured physical activity, but the evidence is very uncertain.	
Subjectively measured physical function; end of intervention: mean 18.5 (SD 10.5) weeks	4065 (26 RCTs)	N/A	N/A	N/A	SMD 0.21 SD higher (0.13 higher to 0.29 higher)	⊕⊕⊕○ Moderate^d,e,g^		Digital health interventions that promote physical activity likely slightly increase subjectively measured physical function.	
Depression assessed with subjective measures; end of intervention: mean 20 (SD 15.4) weeks	4604 (40 RCTs)	N/A	N/A	N/A	SMD 0.25 SD lower (0.35 lower to 0.16 lower)	⊕⊕⊕○ Moderate^d,e^^,^^g^		Digital health interventions that promote physical activity likely slightly reduce depression.	
Anxiety assessed with subjective measures; end of intervention: mean 16 (SD 11.1) weeks	2934 (25 RCTs)	N/A	N/A	N/A	SMD 0.17 SD lower (0.26 lower to 0.09 lower)	⊕⊕○○ Low^d,g,i,j,k^		Digital health interventions that promote physical activity may slightly reduce anxiety.	
Health-related quality of life assessed with subjective measures; end of intervention: mean 24.3 (SD 24.3) weeks	10,645 (74 RCTs)	N/A	N/A	N/A	SMD 0.18 SD higher (0.07 higher to 0.29 higher)	⊕⊕○○ Low^i,j,k^		Digital health interventions that promote physical activity may slightly increase health-related quality of life.	
Nonserious adverse events; end of intervention: mean 21.5 weeks	6813 (45 RCTs)	1.31 (1.11-1.55)^l^	8.5%	11.1% (9.4%-13.2%)	2.6% more (0.9 more to 4.7 more)	⊕⊕⊕○ Moderate^d,e^		Digital health interventions that promote physical activity likely slightly increase nonserious adverse events.	
Serious adverse events; end of intervention: mean 26.2 weeks	10,508 (61 RCTs)	0.89 (0.76-1.04)^l^	8.3%	7.4% (6.3%-8.7%)	0.9% fewer (2 fewer to 0.3 more)	⊕⊕⊕○ Moderate^d,e^		Digital health interventions that promote physical activity likely result in little to no difference in serious adverse events.	

^a^RCT: randomized controlled trial.

^b^N/A: not applicable.

^c^SMD: standardized mean difference.

^d^Some conditions are not represented, or the results only included a few studies within each condition.

^e^Moderate certainty: we are moderately confident in the effect estimate; the true effect is likely to be close to the estimate of the effect, but there is a possibility that it is substantially different.

^f^Very low certainty: we have very little confidence in the effect estimate; the true effect is likely to be substantially different from the estimate of effect.

^g^Studies with low risk of bias reported lower effect on the outcome.

^h^The 95% CI ranges from no effect to high effect.

^i^A possible presence of a small study bias from visual inspection of the funnel plot and Egger test.

^j^Results are inconsistent and measured using the *I*^2^ statistics.

^k^Low certainty: our confidence in the effect estimate is limited; the true effect may be substantially different from the estimate of the effect.

^l^The risk in the intervention group (and its 95% CI) was based on the assumed risk in the comparison group and the relative effect of the intervention (and its 95% CI).

### Meta-Regression, Subgroup Analyses, and Sensitivity Analysis

Univariate meta-regression analysis for objectively measured physical activity showed that increasing age was associated with a lower effect size (slope −0.01, 95% CI −0.03 to −0.00), suggesting an effect reduction of 0.01 SDs per increased year. For objectively measured physical function, a higher effect was found for interventions that combined exercise therapy and physical activity (coefficient [*β*]=1.40, 95% CI 0.42-2.36). A similar effect of age was found for subjectively measured physical activity (slope −0.02, 95% CI −0.03 to −0.00), suggesting an effect reduction of 0.02 SDs per increased year; however, BMI was also associated with a lower effect size (*β*=.04, 95% CI −0.07 to −0.01) for this outcome. Furthermore, for subjectively measured physical activity, shorter intervention duration showed a lower effect size (*β*=−.01, 95% CI −0.01 to −0.00), and the use of a theory or framework showed a decreased effect (*β*=−.31, 95% CI −0.50 to −0.11), which persisted in the multivariate model (adjusted for intervention session, duration, and frequency; *β*=−.28, 95% CI −0.46 to −0.09). For mental health conditions, the univariate analysis showed that participants with higher depression levels had a greater effect on depression symptoms (slope −0.01, 95% CI −0.02 to −0.00), which persisted in the multivariate model (adjusted for age and sex). There were no differences in the rest of the meta-regression analyses (Multimedia Appendix 7) or in the subgroup analyses stratified by conditions, delivery method, or methodology quality (Multimedia Appendix 8 [52-191]).

In an unplanned sensitivity analysis of the primary outcomes of RCTs with a digital-only approach, the results at the end of the intervention were in line with the main analyses (Multimedia Appendix 8).

## Discussion

### Principal Findings

Overall, digital health interventions had a small effect on physical activity and physical function in people with chronic conditions compared with usual care or minimal intervention at the end of the intervention and follow-up. Furthermore, subjectively measured physical function, depression, anxiety, and HRQOL showed a small improvement but only at the end of the intervention. The certainty of the findings was downgraded because of study limitations, imprecision, and inconsistency and was moderate to very low.

### Findings in Comparison With Prior Work

#### Overview

This systematic review was the first to focus primarily on physical activity in several major chronic conditions and across various digital health solutions. A previous systematic review found a large effect of eHealth interventions on physical activity in people with noncommunicable diseases but only included physical activity–integrated and diet-integrated interventions and combined objectively and subjectively physical activity outcomes, thereby limiting clinical interpretation and comparison [81]. Another systematic review focused on multimorbidity and the effect of digital telemedicine on health outcomes but did not report any physical activity outcomes [19]. A third review focused on exercise intervention in people with chronic conditions but solely assessed videoconferencing as the delivery method and found a moderate to small improvement in exercise capacity, which is not directly comparable with physical activity or physical function [25,199]. Finally, a fourth review assessed objectively and subjectively measured physical activity in people with chronic conditions and found a small effect in both outcomes but only significant for the subjective measured outcome [200]. However, this study was limited to the assessment of self-guided digital interventions.

#### Primary Outcomes

To ease the clinical interpretation of objectively measured physical activity, we pooled daily steps data and found an improvement (970.8 steps) comparable with findings in adults using physical activity monitors [201]. Clinically, a 1000-steps increase is considered important and showed a risk reduction in cardiovascular morbidity and all-cause mortality for each additional 1000 daily steps taken, even below the 10,000 daily recommended steps [202]. However, we did not find any improvement in MVPA at the end of the intervention. This intensity level may provide even greater health benefits compared with lower physical activity levels, but importantly, any increase in physical activity is associated with health benefits, regardless of the duration [203].

Assessing objectively measured physical function by pooling 6MWT data, we found an improvement (19.8 m) comparable with previous systematic reviews [15,16] and within the clinically relevant limits of 14.0-30.5 m that are considered clinically meaningful for people across different chronic conditions [204].

#### Secondary Outcomes

Subjectively measured physical activity and physical function showed almost the same effect as objectively measured outcomes at the end of the interventions, but it is known that people tend to overestimate their physical activity level when using subjective outcome measures [205]. It is problematic that the study participants could not be blinded to the intervention, especially when using subjective outcome measures, which is reflected in the higher risk of bias profile of the studies in the subjectively measured outcomes compared with the objectively measured outcomes.

The digital health intervention also showed end-of-intervention effects on other essential health parameters such as depression, anxiety, and HRQOL. Depression and anxiety often coexist with other chronic conditions but are often undetected and untreated [206]. However, digital health interventions may offer a dual effect, as we found both an effect on frequent physical conditions and mental health conditions. Furthermore, our meta-regression indicated that there might be a higher effect for people with higher levels of depression. A similar effect was found in a study that investigated in-person physical activity interventions [5].

HRQOL is an important person-reported outcome measure and is associated with higher rates of hospitalization, morbidity, and mortality [207-209]. Our findings are comparable with those of previous research [210], which also found a small effect on HRQOL in people with physical disabilities (eg, musculoskeletal conditions); however, we have overlapping studies. There has been a focus on measuring HRQOL in health research [208], and HRQOL was by far the most assessed outcome but also showed a high level of heterogeneity, highlighting the differences between trials and within-outcome assessment. This may hide the actual effect because of the differences in the intervention and outcome measures.

Although exercise therapy has proven safe and beneficial for people with multiple chronic conditions [5], safety is paramount when initiating a digital physical activity intervention. We found an increased risk of nonserious events in the intervention groups, commonly musculoskeletal problems, soreness, or pain, which can be expected after engaging in physical activity, even in face-to-face interventions [211]. The higher risk of nonserious events should also be seen in the light of including participants with COPD and heart failure, who often experience complications [212,213]. No increased risk of serious adverse events aligns with previous reviews of in-person physical activity interventions among people with chronic conditions and multimorbidities [5,211].

#### Follow-Up

Only objectively measured physical activity and physical function, including MVPA, effects were sustained at follow-up. Owing to the few studies assessing follow-up, more research is needed to address the long-term effects [214]. Nevertheless, for both subjectively measured physical activity and HRQOL, our meta-regression showed that a shorter intervention duration showed lower effect sizes for the digital health interventions. Although we prespecified our meta-regression analyses, they were exploratory and should be interpreted cautiously.

#### Age and Digital Health

It has been suggested that age and multimorbidity may affect the use of digital technologies [215]. Meta-regression analysis of objectively and subjectively measured physical activity showed that increasing age was associated with a lower effect size; however, the effect was negligible. Furthermore, the included studies mostly had an upper age limit, which is why the full effect of age is not known.

#### Heterogeneity

We expected heterogeneity in our meta-analyses, as there is a clinical diversity in including the different chronic conditions, but further methodological diversity is also present as outcome measures differed among each outcome. There is no consensus on how to measure physical activity best [216]. None of the meta-regression analyses could explain the substantial heterogeneity between studies.

#### Digital Health Interventions

Digital health solutions encompass many different technologies [217]. However, we did not find any difference in the effect of the interventions based on the delivery method. This could be owing to a wide variation across the included interventions. Although more than half of the interventions primarily involved solutions within 1 digital health category (ie, mHealth), commonly, the included studies incorporated >1 technology (eg, apps, telephone calls, and emails). Assessing the effect of different interventions is quite complex, and we encourage the use of reporting guidelines [218-220]. Furthermore, for our study, the interventions also applied different approaches to physical activity (ie, self-management of the physical intervention or structured exercise therapy), which further challenges cross-comparisons.

#### Chronic Conditions

The included studies were generally limited to 1 index condition or reported comorbidities very poorly, making investigations of the results across conditions challenging. Including only participants with 1 condition confines a silo-based approach. A more real-life approach allows participants with more chronic conditions to participate, reflecting that people often have >1 chronic condition [221]. It should also be emphasized that the World Health Organization’s guidelines on physical activity are not restricted to any single condition but apply to all people independent of health conditions [203].

### Strengths and Limitations

This study had some limitations. The risk of selection bias within the RCTs, our inclusion criteria, which focused on 8 specific chronic conditions, thereby excluded, for example, cancer, and the fact that only a few RCTs targeted hypertension, depression, anxiety, and multimorbidity limits the generalizability of our results. Furthermore, few RCTs had follow-up data that induced uncertainty regarding whether the effect was maintained beyond the end of the interventions. In addition, the reporting of digital health solutions and the type and dose of physical activity were poor for many RCTs, preventing us from conducting further analyses on the intervention characteristics. Another limitation is the lack of measuring digital health literacy within the included studies, as it is known that low literacy (both health and digital) affects participation and health outcomes and deepens health inequities [222]. Therefore, we lack information on whether the participants in the included studies were the ones with high or low levels of digital health literacy, as we may suspect that in groups with lower literacy, the effect would become even smaller [223].

The quality of the evidence had to be downgraded to low or very low for objectively measured physical function, subjectively measured physical activity, anxiety, and HRQOL, implying that the actual effect might be different from the estimated effect. In addition, a small study bias cannot be ruled out for some outcomes, although other factors, such as high heterogeneity and low quality of evidence, may contribute to funnel plot asymmetry.

Nevertheless, the strengths of this systematic review are the meticulous, state-of-the-art design and methodology, following the Cochrane handbook and reporting using the PRISMA guidelines. In addition, we assessed the effects across several chronic conditions, breaking down the more silo-based thinking of chronicity.

### Future Research

More high-quality research with better reporting, available protocols, blinding of assessors and study participants (when possible), better handling of missing data, and the use of common and clearly described measurement methods are needed for future research. In addition, such research should assess whether higher and more sustainable effects of digital health interventions that promote physical activity can be achieved. It should also focus on whether any digital health solution or features within these are more effective than others and whether a dose-response relationship exists. Studies focusing on the impact of comorbidities or multimorbidities are also warranted. Furthermore, if digital health solutions are to live up to their potential and be part of the solution to the health care shortage, the digital divide must be addressed by assessing if and how the participants’ digital health literacy levels affect digital health interventions.

### Conclusions and Implications

Engaging people in physical activity is considered a *polypill* that provides high health gains with low risk and low costs [224]. Digital health solutions as a delivery method can provide effective physical activity interventions across people with chronic conditions and may help make these types of interventions available for more people at a lower cost. Therefore, given the potential health benefits, digital health interventions promoting physical activity in people with ≥1 chronic condition should be considered in clinical practice, especially if the overall aim is to improve physical activity levels. However, educating participants about the potential risk of nonserious adverse events is essential. The findings of this study are also relevant for policy makers because easy and affordable access to high-quality health care services is at the top of the political agenda. However, it should be noted that the effects were small and, for most outcomes, limited to the end of the intervention, with analyses showing substantial heterogeneity between studies and many studies with some concerns for the overall risk of bias.

## Appendix

Multimedia Appendix 1 Study protocol including amendments to protocol.

Multimedia Appendix 2 PRISMA (Preferred Reporting Items for Systematic Reviews and Meta-Analyses) checklist.

Multimedia Appendix 3 Search string for each database, excluded studies, study characteristics of the included studies, and overview of index conditions and comorbidities in the included studies.

Multimedia Appendix 4 Meta-analyses: forest plots for each outcome of interest.

Multimedia Appendix 5 Risk of bias: Cochrane Risk of Bias 2 assessment of each outcome of interest.

Multimedia Appendix 6 Small study bias: funnel plot and Egger or Harbor test for each outcome of interest.

Multimedia Appendix 7 Meta-regression analyses.

Multimedia Appendix 8 Subgroup and sensitivity analyses.

## Abbreviations

6MWT: 6-minute walk test
COPD: chronic obstructive pulmonary disease
HRQOL: health-related quality of life
mHealth: mobile health
MVPA: moderate to vigorous physical activity
PRISMA: Preferred Reporting Items for Systematic Reviews and Meta-Analyses
RCT: randomized controlled trial
SMD: standardized mean difference
