# Supplementary material for: Benefits and Harms of Digital Health Interventions Promoting Physical Activity in People With Chronic Conditions: Systematic Review and Meta-Analysis
Source: J Med Internet Res. 2023 Jul 6;25:e46439. doi: 10.2196/46439 (PMC10359919; doi:10.2196/46439)
Supplement: Multimedia Appendix 1 [file jmir_v25i1e46439_app1.pdf]

Multimedia Appendix 1: Study Protocol

To manuscript: **Benefits and Harms of Digital Health Interventions Promoting Physical Activity in People with Chronic Conditions: A Systematic Review and Meta-Analysis**

Study Protocol

Final title

Benefits and harms of digital health interventions promoting physical activity in people with chronic conditions - A systematic review and meta-analysis

Running title

The effect of eHealth interventions targeting physical activity on physical and psychosocial outcomes in people with a single chronic condition or multimorbidity - A systematic review of randomised controlled trials

Working group

Graziella Zangger, Alessio Bricca, Behnam Liaghat, Carsten B Juhl, Sofie Rath Mortensen, Rune Martens Andersen, Camma Damsted, Trine Grønbek Hamborg, Mathias Ried-Larsen, Lars Herman Tang, Lau Caspar Thygesen, and Søren T Skou

Amendments to the Protocol

Changes were made before the analyses were initiated. The word eHealth was changed to digital health to better encompass all technologies included, as eHealth may be understood as only covering technologies that use the internet. Furthermore, in the outcome hierarchy, the VO_{2max} test (eg, indirect calorimetry or Watt-max test) was not performed because the 6-minute walk test was prioritized instead to reflect the more common clinically important assessment used. In addition, a manual search of specific journals, full-text reference lists, and Web of Science citation tracking was not performed because of the many full texts retrieved via the search. Furthermore, the planned meta-regression on health-related quality-of-life levels was not performed because of limitations in the data. For index condition, delivery method, and methodology quality, some categories had <10 RCTs contributing to the data, and a subgroup analysis was performed instead. Finally, the planned meta-regression of the impact of the number of chronic conditions and investigations of the effect of physical activity between people with different clusters of conditions were not performed because of the inconsistency of reporting or lack of information about comorbidity and few multimorbidity RCTs. Finally, the authors did not provide a narrative description of the studies that did not have data to be included in a meta-analysis, as this would not have improved the confidence of our results because of the large number of included studies in the meta-analyses.

Background

The focus on using information and communication technologies in health, also known as Digital health or eHealth [33], has steadily increased over the recent years. As the population worldwide is ageing, there is a higher prevalence of chronic conditions and multimorbidity, which demands long-term and often more complex healthcare services. At the same time, patients have rising expectations of getting the highest quality of care, and together this is pressuring the healthcare systems [34]. Digital health tools have been proposed as part of the solution to ease a challenged healthcare system [33, 35]. Moreover, by following a structured exercise therapy program, being physically active reduces symptoms of at least 26 chronic conditions with up to 30%, [4] and physical activity is associated with psychological benefits and maintained or improved well-being and quality of life [36]. Digital health tools have been proposed as a delivery mode for physical activity [37]. Although digital health technologies have great potential and despite political interest and investments in Europe, little is known on the effect of digital health solutions in promoting physical activity for people with multimorbidity or common chronic conditions within multimorbidity [26, 38].

In this review, we will focus on the following chronic conditions: osteoarthritis (knee and hip), ischemic heart disease, heart failure, hypertension, type 2 diabetes mellitus, chronic obstructive pulmonary disease, depression and anxiety. Focusing on these conditions is that they share a common health risk factor of physical inactivity and the pathogenesis of systemic low-grade inflammation [39]. Additionally, these conditions are also among the leading causes of global disability and affect hundreds of millions of people around the world [40] and are some of the most commonly reported conditions within multimorbidity research [41].

Research question

What is the effect of digital health interventions targeting physical activity on physical and psychosocial outcomes in people with a single chronic condition or with multimorbidity?

Objectives

With this systematic review, we aim to assess what digital health solutions targeting physical activity is available for people with multimorbidity or common chronic conditions within multimorbidity. Further, we aim to evaluate these solutions' effects on physical and psychosocial outcomes, as objectively measured or self-reported levels of physical activity, physical function, depression, anxiety and health-related quality of life. Additionally, we aim to explore if numbers or clusters of conditions are associated with the effect of using digital health solutions.

Population of interest

Adults (≥18 years of age), one or more of the following chronic conditions: osteoarthritis (knee or hip), ischemic heart disease, heart failure, hypertension, type 2 diabetes, chronic obstructive pulmonary disease, depression and anxiety.

Intervention

Any interventions in which a digital health solution targeting physical activity with or without additional pharmacotherapy or other adjuvant interventions (e.g. weight loss) are used, independent of if the physical activity intervention is the primary or secondary aim of the intervention. Digital health solutions are defined as any use of digital technologies to deliver or enhance the communication of health information between patient and health care provider over a distance[1] (e.g. app, texts, telecommunication etc.).

Comparator

Not exposed to the digital health solution (usual care, education or any other comparator that is not provided as a digital health solution).

Outcomes of interest

We will prioritise data extraction of outcome measures rated as important for the participants with multimorbidity [42]. The primary outcomes will be physical activity and physical function.

For objectively physical activity, we will prioritise: 1) accelerometers measures (e.g. daily time spent in moderate to vigorous physical activity); 2) pedometer (e.g. outcomes such as step counts); 3) any other outcome measure related to objectively measured physical activity.

For subjectively measured physical activity, we will prioritise: 1) the Global Physical Activity Questionnaire; 2) the Physical Activity Scale for the Elderly (PASE) Questionnaire; 3) the International Physical Activity Questionnaires (IPAQ) long- and short-form; 4) any other outcome measure related to subjectively measured physical activity.

For objectively measured physical function, we will prioritise: 1) Vo2max test (e.g. indirect calorimetry or watt-max test); 2) the 6-minute walk test; 3) incremental shuttle walk test; 4) any other outcome measure related to daily function (e.g. Chair stand test).

For self-reported physical function, we will prioritise: 1) the 36-item Short-Form Health Survey (SF-36), Physical Function subscale, Role Function subscale, or the Physical Summary Score; 3) any other self-reported measure of physical function.

The secondary outcomes will be health-related quality of life, depression, anxiety and adverse events.

For health-related quality of life outcomes, we will prioritise: 1) the EQ-5D questionnaire; 2) any other generic health-related quality of life questionnaires; 3) disease-specific health related quality of life questionnaires (e.g. The Minnesota living with heart failure questionnaire).

For depression, we will prioritise: 1) The Beck Depression Inventory (BDI); 2) any other depression questionnaire (e.g. the Hospital Anxiety and Depression Scale (HADS depression); 3) any other assessment of depression (e.g. clinical).

For anxiety, we will prioritise: 1) State Trait Anxiety Inventory questionnaire; 2) any other anxiety questionnaire (e.g. HADS anxiety); 3) any other assessment of anxiety (e.g. clinical).

If reported in included trials, adverse events are divided by types, i.e. serious or non-serious in concordance with the U.S. Food and Drug Administration [29].

Types of studies to be included: Randomised controlled trials published in peer review journals will be included. Unpublished studies (e.g. conference abstracts, trial protocols) will be excluded. We will include studies that comprise both participants with one of the conditions (e.g. 100% of the participants report having only osteoarthritis) and studies with more than one of the conditions (e.g. 100% of the participants reporting osteoarthritis and hypertension). We will label studies that include participants with more than one of the conditions as 'multimorbidity' [43].

Methods

The systematic review and meta-analyses will be cross-registered at the PROSPERO database and the Open Science Framework. The systematic review will follow the methods of the Cochrane Handbook for Systematic Reviews [44] and reported according to the PRISMA guidelines [46].

Information sources and search strategy

Studies will be identified by searching MEDLINE, EMBASE, CINAHL and CENTRAL. The search will be built by using index terms, MeSH terms, keywords, and appropriate synonyms of the words; digital health, eHealth, mHealth, physical activity, chronic conditions, and multimorbidity. No limits are set for publication date or language. Relevant literature will also be found by searching specific journals that focus on Digital health or eHealth, reference lists of relevant systematic reviews on digital health, eHealth, mHealth, physical activity and chronic conditions, or multimorbidity, as well as the reference lists of included studies. Moreover, citation tracking will be performed on the Web of Science.

Data management

Two independent reviewers (Graziella Zangger (GZ) and Alessio Bricca (AB)) will apply the selection criteria and screen the titles and abstracts using Covidence [193]. The included studies will subsequently be read in full text for a final selection. A third member (Carsten B. Juhl (CBJ)) of the study team will be consulted in case of disagreement throughout the review process.

Data collection process

Two independent reviewers (GZ and AB) will perform the data extraction of: study design, study population (sample size, age, gender, socioeconomic status, employment status, index condition(s), comorbidities (e.g. other conditions than the index condition(s) this can be other than the conditions of interest ex cancer), numbers of comorbidity, comorbidity index score (e.g. the Charlson Comorbidity Index), disease severity or morbidity burden (e.g. Chronic Disease Score), allocation of participants, and health/eHealth literacy level), type of intervention (digital health/eHealth mode, physical activity type and dose (frequency, intensity and duration)), and any other supporting intervention(s) (e.g. education, psychotherapy, diet), adherence and attrition to intervention, objectively measured outcomes (in concordance with the above mentions list), type of measurement (e.g. Actigraph, Actical) (for Actigraph, Actical, version, cut-off point (e.g. epoch and bout length), and placement (e.g. hip, wrist) will also be reported), patient-reported outcome measures (in concordance with the above mentions list) and study settings (site and country of intervention).

The results from both Intention-To-Treat (ITT) and Per Protocol (PP) will be extracted from the included studies. If any values are missing (e.g. standard deviation), they will, if possible, be calculated from the available data (p-, t- or f-values, confidence intervals, standard errors or measured on figures) following the Cochrane handbook. If necessary, authors will be contacted to obtain missing data related to the intervention or outcome.

Risk of bias

All included studies will, by two independent assessors, be evaluated for risk of bias and quality using the Cochrane risk of bias tool 2.0 [30]. Further, the Grading of Recommendations Assessment, Development and Evaluation (GRADE) method will be used in evaluating the quality of evidence of results on each of the outcomes. The GRADE evaluation will assess study quality, indirectness, imprecision and inconsistency and publication bias [31].

Data synthesis

We will perform a meta-analysis for each outcome domain of interest. We will perform a random-effects model as heterogeneity is expected due to differences in participants, interventions, outcome measures etc. Heterogeneity will be examined as between-study variance and calculated as the I-squared statistic

measuring the proportion of variation in the combined estimates due to between-study variance. An I-squared value of 0% indicates no inconsistency between individual trials' results, and an I-squared value of 100% indicates maximal inconsistency. Standardised mean differences (SMD) with 95% CIs will be calculated for outcome measures of continuous data and adjusted to Hedges g. The magnitude of the effect size of the pooled SMD will be interpreted as 0.2 representing a small effect, 0.5 a moderate effect, and 0.8 a large effect [32]. For outcome measures where a meta-analysis is not possible, a narrative data synthesis of the results from individual studies will be performed in line with the guidance from the Cochrane handbook [44].

Sub-group analyses will be performed to explore whether physical activity (exercise) affects people with different chronic conditions and in different clusters of conditions. Meta-regression analyses will be performed to identify factors (covariates) predicting a better outcome. Relevant study-level covariates are defined as ones able to decrease inconsistency measured as the between-study variance Tau-square (and thus the I-squared statistic). Finally, we will investigate the impact of participants (e.g. age, gender, level of socioeconomics, number and severity of the chronic conditions and quality of life), interventions (e.g. mobile or web-based tools) and methodological quality of the studies (by classifying studies at "low risk of bias", "some concerns", or "high risk of bias" according to the Cochrane Risk of Bias tool 2.0 [30]) on the outcomes of interest.

Dissemination plans

We will submit this systematic review for publication in a peer-reviewed journal. Further, the results will be part of a PhD thesis and presented at relevant national/international conferences and to relevant stakeholders. In addition, we will also deliver the results as an infographic, video, on social media and on the website of the Mobilize/Exercise first research programs.

This study has been presented and evaluated by a group of patient partners in line with the patient involvement of both the PhD project and the Mobilize project. Hence, the Danish layman description. An English layman description is also available.

English layman description

Living with multiple chronic conditions at the same time, is becoming more common as the population ages. Although people living with multiple chronic conditions are more frequent among the elderly, it is also very common in the working-age population. Studies have shown that almost 30% of those between 45-55 years old live with more than two chronic conditions at the same time, while this is true for almost every individual over 85 years. Physical activity is effective in managing chronic conditions and can help improve function and quality of life. However, only a small percentage of people living with chronic conditions meet the recommended physical activity levels. Evidence suggests that digital health solutions, the use of information

and communication technologies in healthcare, like smartphones apps or the Internet, have a positive impact on managing chronic conditions. However, there is a lack of knowledge about whether digital health solutions can increase physical activity among people living with multiple chronic conditions. The present study focuses on people with osteoarthritis of the knee or hip, heart failure, ischemic heart disease, high blood pressure, type 2 diabetes, chronic obstructive pulmonary disease, depression and anxiety. These conditions are among the leading causes of the global disease burden and affect hundreds of millions of people worldwide. In addition, these diseases are among some of the most common in the research field of multiple chronic conditions. Therefore, the purpose of this study is to systematically review current scientific literature to investigate if digital health solutions, aimed at promoting physical activity among people with one or more of the aforementioned chronic conditions are effective.

Anticipated or actual start date

May 2020

Corresponding authors

- Graziella Zangger, e-mail: gzangger@health.sdu.dk
- Alessio Bricca, e-mail: abricca@health.sdu.dk

References

See main paper for reference list:

Zangger G, Bricca A, Liaghat B, Juhl CB, Mortensen SR, Andersen RM, Damsted C, Hamborg TG, Ried-Larsen M, Tang LH, Thygesen LC, Skou ST. Benefits and Harms of Digital Health Interventions Promoting Physical Activity in People With Chronic Conditions: Systematic. Review and Meta-Analysis. J Med Internet Res 2023;25:e46439. URL: https://www.jmir.org/2023/1/e46439/ doi: 10.2196/46439
