# Supplementary material for: Benefits and Harms of Digital Health Interventions Promoting Physical Activity in People With Chronic Conditions: Systematic Review and Meta-Analysis
Source: J Med Internet Res. 2023 Jul 6;25:e46439. doi: 10.2196/46439 (PMC10359919; doi:10.2196/46439)
Supplement: Multimedia Appendix 3 [file jmir_v25i1e46439_app3.pdf]

Multimedia Appendix 3: Tables

To manuscript: **Benefits and Harms of Digital Health Interventions Promoting Physical Activity in People with Chronic Conditions: A Systematic Review and Meta-Analysis**

List of Tables

Supplementary Table 1. Search string for each database
Supplementary Table 2. List of excluded full-text studies and reason for exclusion
Supplementary Table 3. Study characteristics of the included studies
Supplementary Table 4. The percentage of comorbidities among the participants of each study

Supplementary Table 1. Search string for each database

MEDLINE	
1	Physical activity.mp.
2	Physical activity.ti,ab,id.
3	exp Sports/
4	Sport*.ti,ab,id.
5	Work-out.ti,ab,id.
6	Workout.ti,ab,id.
7	Working out.ti,ab,id.
8	Aerobics.ti,ab,id.
9	Fitness.ti,ab,id.
10	Physical Fitness/
11	Physical conditioning.ti,ab,id.
12	Physically fit.ti,ab,id.
13	Active lifestyle.ti,ab,id.
14	Exercise/
15	Exercis*.ti,ab,id.
16	exp Exercise Movement Techniques/
17	exp Exercise Therapy/
18	Exercise Therapy.ti,ab,id.
19	Locomotion/
20	exp Physical Therapy Modalities/
21	Physiotherapy.ti,ab,id.
22	Physical Therapy.ti,ab,id.
23	"Physical Education and Training"/
24	Motor Activity/
25	exp Resistance Training/
26	Resistance Training.ti,ab,id.
27	Strengthening.ti,ab,id.
28	Muscle strength.mp.
29	Muscle strength.ti,ab,id.
30	exp Walking/
31	Walking.ti,ab,id.
32	exp Running/
33	Running.ti,ab,id.
34	Jogging.mp.
35	Jogging.ti,ab,id.
36	Cycling.ti,ab,id.
37	exp Bicycling/
38	exp Swimming/
39	Swimming.ti,ab,id.
40	exp Gymnastics/
41	gymnastic*.ti,ab,id.

42 exp Yoga/
43 exp Dancing/
44 pilates.ti,ab,id.
45 or/1-44
46 digital health.mp.
47 exp Telemedicine/
48 telemedicine.ti,ab,id.
49 tele-medicine.ti,ab,id.
50 telecare.ti,ab,id.
51 tele-care.ti,ab,id.
52 telehomecare.ti,ab,id.
53 tele-homecare.ti,ab,id.
54 telehealthcare.ti,ab,id.
55 tele-healthcare.ti,ab,id.
56 tele-monitoring.ti,ab,id.
57 telemonitoring.ti,ab,id.
58 telehealth.ti,ab,id.
59 tele-health.ti,ab,id.
60 exp Telecommunications/
61 ehealth.mp.
62 ehealth.ti,ab,id.
63 e-health.mp.
64 e-health.ti,ab,id.
65 electronic health*.ti,ab,id.
66 mhealth.mp.
67 mhealth.ti,ab,id.
68 m-health.mp.
69 m-health.ti,ab,id.
70 mobile health.mp.
71 Electronic Mail/
72 e-mail*.ti,ab,id.
73 email*.ti,ab,id.
74 ("e-mail based" or e-mail-based or email-based or "email based").ti,ab,id.
75 exp Internet/
76 Internet-Based Intervention/
77 web-based.ti,ab,id.
78 internet-based.ti,ab,id.
79 exp Computer-Assisted Instruction/
80 exp Programmed Instruction as Topic/
81 exp communications media/
82 on-line communication.ti,ab,id.
83 online communication.ti,ab,id.

84 e-Portal.ti,ab,id.

85 ePortal.ti,ab,id.

86 Computer System*.mp.

87 Computers/

88 e-consultation.ti,ab,id.

89 Remote Consultation.ti,ab,id.

90 e-care.ti,ab,id.

91 eTherap*.ti,ab,id.

92 e-therap*.ti,ab,id.

93 teletherap*.ti,ab,id.

94 exp Telerehabilitation/

95 telerehabilitation.ti,ab,id.

96 tele-rehabilitation.ti,ab,id.

97 exp microcomputers/

98 (Computers or tablet* or android*).ti,ab,id.

99 (Ipad* or iphone* or ipod* or iso).ti,ab,id.

100 (ios or android$).tw.

101 exp Cell Phone/

102 Telephone/

103 Mobile phone.ti,ab,id.

104 Smartphone/

105 ("Mobile phone" or telephone or smartphone or "cell* ajd1 phone").ti,ab,id.

106 exp Text Messaging/

107 ((sms or mms or text) and messag$).tw.

108 "text messag$".tw.

109 multi-media.ti,ab,id.

110 multimedia.ti,ab,id.

111 mobile applications/

112 (smartphone adj3 app*).ti,ab,id.

113 app.ti,ab,id.

114 apps.ti,ab,id.

115 social media$.tw.

116 facebook.tw.

117 (twitter or tweet$).tw.

118 or/46-117

119 Multimorbidity/

120 Multimorbid*.ti,ab,id.

121 Multi-morbid*.ti,ab,id.

122 Multidisease?.ti,ab,id.

123 Multi-disease?.ti,ab,id.

124 Multiple disease?.ti,ab,id.

125 Multiple diagno*.ti,ab,id.

126	Multicondition?.ti,ab,id.
127	((Multi or multiple) adj2 (morbid* or disease? or condition? or ill* or syndrom* or symptom* or disorder? or health)).ti,ab,id.
128	((Associated or Co-existing or Concurrent or Cooccurring or Chronic*) adj (morbid* or disease? or condition? or ill* or syndrom* or symptom* or disorder? or health)).ti,ab,id.
129	Polypathology.mp.
130	Pluripathology.mp.
131	Comorbidity/
132	Comorbid*.ti,ab,id.
133	Co-morbid*.ti,ab,id.
134	(Pattern* adj2 disease?).ti,ab,id.
135	(Cluster* adj2 disease?).ti,ab,id.
136	Multiple Chronic Conditions/
137	Noncommunicable Diseases/
138	or/119-137
139	Hypertension/
140	hypertension.ti,ab,id.
141	hypertens*.ti,ab,id.
142	high blood pressure.mp.
143	or/139-142
144	myocardial ischemia/
145	acute coronary syndrome/
146	coronary disease/
147	myocardial infarction/
148	Coronary Artery Disease/
149	(myocard* adj (ischem* or ischaem* or infact*)).ti,ab,id.
150	((ischem* or ischaem*) adj heart disease).ti,ab,id.
151	or/144-150
152	Heart Failure/
153	Heart Failure.ti,ab,id.
154	or/152-153
155	exp Pulmonary Disease, Chronic Obstructive/
156	COPD.ti,ab,id.
157	Chronic Obstructive Lung Disease.ti,ab,id.
158	Chronic bronchitis.ti,ab,id.
159	Pulmonary emphysema.ti,ab,id.
160	or/155-159
161	exp Depression/
162	Depress*.ti,ab,id.
163	depressive disorder, major/
164	dysthymic disorder/
165	dysthymic disorder.ti,ab,id.
166	dysthymi*.ti,ab,id.

167	affect* disorder.ti,ab,id.
168	or/161-167
169	diabetes mellitus/
170	diabetes mellitus, type 2/
171	Type 2 diab*.ti,ab,id.
172	Type II diab*.ti,ab,id.
173	Non-Insulin-Dependent Diabetes Mellitus.ti,ab,id.
174	NIDDM.ti,ab,id.
175	diabetes mellitus.ti,ab,id.
176	or/171-175
177	exp Osteoarthritis/
178	exp Osteoarthritis, Knee/
179	exp Osteoarthritis, hip/
180	Osteoarthritis.ti,ab,id.
181	Osteoarthr$.ti,ab,id.
182	(degenerative adj2 arthritis).ti,ab,id.
183	arthrosis.ti,ab,id.
184	or/177-183
185	exp Anxiety Disorders/
186	Anxiety/
187	Anxiety.ti,ab,id.
188	or/185-187
189	randomized controlled trial.pt.
190	controlled clinical trial.pt.
191	randomized.ab.
192	placebo.ab.
193	drug therapy.fs.
194	randomly.ab.
195	trial.ab.
196	groups.ab.
197	or/189-196
198	exp animals/ not humans.sh.
199	197 not 198
200	138 or 143 or 151 or 154 or 160 or 168 or 176 or 184 or 188
201	45 and 118 and 200 and 199
**Embase**	
1	Physical activity.mp.
2	Physical activity.ti,ab,kw.
3	exp Sports/
4	Sport*.ti,ab,kw.
5	Work-out.ti,ab,kw.
6	Workout.ti,ab,kw.
7	Working out.ti,ab,kw.

8 Aerobics.ti,ab,kw.
9 Fitness.ti,ab,kw.
10 Physical Fitness/
11 Physical conditioning.ti,ab,kw.
12 Physically fit.ti,ab,kw.
13 Active lifestyle.ti,ab,kw.
14 Exercise/
15 Exercis*.ti,ab,kw.
16 exp Exercise Movement Techniques/
17 exp Exercise Therapy/
18 Exercise Therapy.ti,ab,kw.
19 Locomotion/
20 exp Physical Therapy Modalities/
21 Physiotherapy.ti,ab,kw.
22 Physical Therapy.ti,ab,kw.
23 exp physical education/
24 Motor Activity/
25 exp Resistance Training/
26 Resistance Training.ti,ab,kw.
27 Strengthening.ti,ab,kw.
28 Muscle strength.mp.
29 Muscle strength.ti,ab,kw.
30 exp Walking/
31 Walking.ti,ab,kw.
32 exp Running/
33 Running.ti,ab,kw.
34 Jogging.mp.
35 Jogging.ti,ab,kw.
36 Cycling.ti,ab,kw.
37 exp Bicycling/
38 exp Swimming/
39 Swimming.ti,ab,kw.
40 exp Gymnastics/
41 gymnastic*.ti,ab,kw.
42 exp Yoga/
43 exp Dancing/
44 pilates.ti,ab,kw.
45 or/1-44
46 digital health.mp.
47 exp Telemedicine/
48 telemedicine.ti,ab,kw.
49 tele-medicine.ti,ab,kw.
50 telecare.ti,ab,kw.

51 tele-care.ti,ab,kw.
52 telehomecare.ti,ab,kw.
53 tele-homecare.ti,ab,kw.
54 telehealthcare.ti,ab,kw.
55 tele-healthcare.ti,ab,kw.
56 tele-monitoring.ti,ab,kw.
57 telemonitoring.ti,ab,kw.
58 telehealth.ti,ab,kw.
59 tele-health.ti,ab,kw.
60 exp Telecommunications/
61 ehealth.mp.
62 ehealth.ti,ab,kw.
63 e-health.mp.
64 e-health.ti,ab,kw.
65 electronic health*.ti,ab,kw.
66 mhealth.mp.
67 mhealth.ti,ab,kw.
68 m‐health.mp.
69 m-health.ti,ab,kw.
70 mobile health.mp.
71 Electronic Mail/
72 e-mail*.ti,ab,kw.
73 email*.ti,ab,kw.
74 ("e-mail based" or e-mail-based or email-based or "email based").ti,ab,kw.
75 exp Internet/
76 Internet-Based Intervention/
77 web-based.ti,ab,kw.
78 internet-based.ti,ab,kw.
79 exp Computer-Assisted Instruction/
80 exp Programmed Instruction as Topic/
81 exp communications media/
82 on-line communication.ti,ab,kw.
83 online communication.ti,ab,kw.
84 e‐Portal.ti,ab,kw.
85 ePortal.ti,ab,kw.
86 Computer System*.mp.
87 Computers/
88 e-consultation.ti,ab,kw.
89 Remote Consultation.ti,ab,kw.
90 e-care.ti,ab,kw.
91 eTherap*.ti,ab,kw.
92 e‐therap*.ti,ab,kw.
93 teletherap*.ti,ab,kw.

94	exp Telerehabilitation/
95	telerehabilitation.ti,ab,kw.
96	tele-rehabilitation.ti,ab,kw.
97	exp microcomputers/
98	(Computers or tablet* or android*).ti,ab,kw.
99	(Ipad* or iphone* or ipod* or iso).ti,ab,kw.
100	(ios or android$).tw.
101	exp Cell Phone/
102	Telephone/
103	Mobile phone.ti,ab,kw.
104	Smartphone/
105	("Mobile phone" or telephone or smartphone or "cell* ajd1 phone").ti,ab,kw.
106	exp Text Messaging/
107	((sms or mms or text) and messag$).tw.
108	text messag$.tw.
109	multi‐media.ti,ab,kw.
110	multimedia.ti,ab,kw.
111	mobile applications/
112	(smartphone adj3 app*).ti,ab,kw.
113	app.ti,ab,kw.
114	apps.ti,ab,kw.
115	social media$.tw.
116	facebook.tw.
117	(twitter or tweet$).tw.
118	or/46-117
119	Multimorbidity/
120	Multimorbid*.ti,ab,kw.
121	Multi-morbid*.ti,ab,kw.
122	Multidisease?.ti,ab,kw.
123	Multi-disease?.ti,ab,kw.
124	Multiple disease?.ti,ab,kw.
125	Multiple diagno*.ti,ab,kw.
126	Multicondition?.ti,ab,kw.
127	((Multi or multiple) adj2 (morbid* or disease? or condition? or ill* or syndrom* or symptom* or disorder? or health)).ti,ab,kw.
128	((Associated or Co-existing or Concurrent or Cooccurring or Chronic*) adj (morbid* or disease? or condition? or ill* or syndrom* or symptom* or disorder? or health)).ti,ab,kw.
129	Polypathology.mp.
130	Pluripathology.mp.
131	Comorbidity/
132	Comorbid*.ti,ab,kw.
133	Co-morbid*.ti,ab,kw.
134	(Pattern* adj2 disease?).ti,ab,kw.
135	(Cluster* adj2 disease?).ti,ab,kw.

136 Multiple Chronic Conditions/
137 Noncommunicable Diseases/
138 or/119-137
139 Hypertension/
140 hypertension.ti,ab,kw.
141 hypertens*.ti,ab,kw.
142 high blood pressure.mp.
143 or/139-142
144 myocardial ischemia/
145 acute coronary syndrome/
146 coronary disease/
147 myocardial infarction/
148 Coronary Artery Disease/
149 (myocard* adj (ischem* or ischaem* or infact*)).ti,ab,kw.
150 ((ischem* or ischaem*) adj heart disease).ti,ab,kw.
151 or/144-150
152 Heart Failure/
153 Heart Failure.ti,ab,kw.
154 or/152-153
155 exp Pulmonary Disease, Chronic Obstructive/
156 COPD.ti,ab,kw.
157 Chronic Obstructive Lung Disease.ti,ab,kw.
158 Chronic bronchitis.ti,ab,kw.
159 Pulmonary emphysema.ti,ab,kw.
160 or/155-159
161 exp Depression/
162 Depress*.ti,ab,kw.
163 depressive disorder, major/
164 dysthymic disorder/
165 dysthymic disorder.ti,ab,kw.
166 dysthymi*.ti,ab,kw.
167 affect* disorder.ti,ab,kw.
168 or/161-167
169 diabetes mellitus/
170 diabetes mellitus, type 2/
171 Type 2 diab*.ti,ab,kw.
172 Type II diab*.ti,ab,kw.
173 Non-Insulin-Dependent Diabetes Mellitus.ti,ab,kw.
174 NIDDM.ti,ab,kw.
175 diabetes mellitus.ti,ab,kw.
176 or/171-175
177 exp Osteoarthritis/
178 exp Osteoarthritis, Knee/

179 exp Osteoarthritis, hip/<br>180 Osteoarthritis.ti,ab,kw.<br>181 Osteoarthr$.ti,ab,kw.<br>182 (degenerative adj2 arthritis).ti,ab,kw.<br>183 arthrosis.ti,ab,kw.<br>184 or/177-183<br>185 exp Anxiety Disorders/<br>186 Anxiety/<br>187 Anxiety.ti,ab,kw.<br>188 or/185-187<br>189 random:.tw.<br>190 placebo:.mp.<br>191 double-blind:.tw.<br>192 or/189-191<br>193 138 or 143 or 151 or 154 or 160 or 168 or 176 or 184 or 188<br>194 45 and 118 and 193 and 192<br>195 exp animals/ not exp human/<br>196 (45 and 118 and 193 and 192) not 195	
CINAHL	
S9	S1 AND S2 AND S3 AND S8
S8	S4 NOT S7
S7	S5 NOT S6
S6	(MH human)
S5	(MH animals+) OR (MH animal studies) OR (TI animal model*)
S4	(MH randomized controlled trials) OR (MH double-blind studies) OR (MH single-blind studies) (MH random assignment) OR (MH pretest-posttest design) OR (MH cluster sample) OR (TI randomised OR randomized) OR (AB random*) OR (TI trial) OR ((MH sample size) AND (AB assigned OR allocated OR control)) OR (MH placebos) OR (PT randomized controlled trial) OR (AB control W5 group) OR (MH crossover design) OR (MH comparative studies) OR (AB cluster W3 RCT)
S3	"Multimorbidity" OR TI Multimorbid* OR AB Multimorbid* OR TI Multi-morbid* OR AB Multi-morbid* OR TI Multidisease# OR AB Multidisease# OR TI Multi-disease# OR AB Multi-disease# TI "Multiple disease#" OR AB "Multiple disease#" OR TI "Multiple diagno*" OR AB "Multiple diagno*" OR TI Multicondition# OR AB Multicondition# OR Polypathology OR Pluripathology OR (MH "Comorbidity+") OR TI Comorbid* OR AB Comorbid* OR TI Co-morbid* OR AB Co-morbid* OR ((TI Cluster* OR AB Cluster*) N2 (TI disease# OR AB disease#)) OR TI "Multiple Chronic Conditions" OR AB "Multiple Chronic Conditions" OR (MH "Noncommunicable Diseases") OR (MH "Hypertension+") OR (MH "Hypertension, Isolated Systolic") OR TI hypertension OR AB hypertension OR TI hypertens* OR AB hypertens* OR (TI "high blood pressure") OR (AB "high blood pressure") OR (MH "myocardial ischemia+") OR (MH "acute coronary syndrome") OR (MH "coronary disease+") OR (MH "myocardial infarction+") OR ((TI myocard* OR AB myocard) N2 (TI ischem* OR AB ischem* OR TI ischaem* OR AB ischaem* OR TI infact* OR AB infact*)) OR ((TI ischem* OR AB ischem* OR TI ischaem* OR AB ischaem*) N4 (TI "heart disease" OR AB "heart disease")) OR (MH "Heart Failure+") OR TI "Heart Failure" OR AB "Heart Failure" OR (MH "Pulmonary Disease, Chronic Obstructive+") OR TI COPD OR AB COPD OR TI "Chronic Obstructive Lung Disease" OR AB "Chronic Obstructive Lung Disease" OR (MH "Bronchitis+") OR TI "Chronic bronchitis" OR AB "Chronic bronchitis" OR (MH "Emphysema") OR TI "Pulmonary emphysema" OR AB "Pulmonary emphysema" OR (MH "Depression+") OR TI Depress* OR AB Depress* OR TI "Major depressive disorder" OR AB "Major depressive disorder" OR TI "major depression" OR AB "major depression" OR (MH "Dysthymic Disorder") OR TI "dysthymic disorder" OR AB "dysthymic disorder" OR TI dysthymi* OR AB dysthymi* OR TI "affect* disorder" OR AB "affect* disorder" OR (MH "Diabetes Mellitus+") OR (MH "Diabetes Mellitus, Type 2") OR TI "Type diab*" OR AB "Type diab*" OR TI "Type II diab*" OR AB "Type II diab*" OR TI "Non-Insulin-Dependent Diabetes

	Mellitus" OR AB "Non-Insulin-Dependent Diabetes Mellitus" OR TI NIDDM OR AB NIDDM OR TI "diabetes mellitus" OR AB "diabetes mellitus" OR (MH "Osteoarthritis+") OR TI Osteoarthritis OR AB Osteoarthritis OR TI Osteoarthr* OR AB Osteoarthr* OR (TI degenerative OR AB degenerative N2 TI arthritis OR AB arthritis) OR TI arthrosis OR AB arthrosis OR (MH "Osteoarthritis, Knee") OR (MH "Osteoarthritis, Hip") OR (MH "Anxiety Disorders+") OR (MH "Anxiety+") OR TI Anxiety OR AB Anxiety
S2	TI "digital health" OR AB "digital health" OR (MH "Telemedicine+") OR TI telemedicine OR AB telemedicine OR TI tele-medicine OR AB tele-medicine OR TI telecare OR AB telecare OR TI tele-care OR AB tele-care OR TI telehomecare OR AB telehomecare OR TI tele-homecare OR AB tele-homecare OR TI telehealthcare OR AB telehealthcare OR TI tele-healthcare OR AB tele-healthcare OR TI tele-monitoring OR AB tele-monitoring OR TI telemonitoring OR AB telemonitoring OR (MH "Telehealth+") OR TI telehealth OR AB telehealth OR TI tele-health OR AB tele-health OR (MH "Telecommunications+") OR ehealth OR TI ehealth OR AB ehealth OR e-health OR TI e-health OR AB e-health OR TI "electronic health*" OR AB "electronic health*" OR mhealth OR TI mhealth OR AB mhealth OR m-health OR TI m-health OR AB m-health OR TI "mobile health" OR AB "mobile health" OR (MH "Internet+") OR TI Internet-Based or AB Internet-Based OR TI web-based OR AB web-based OR TI e-Portal OR AB e-Portal OR TI ePortal OR AB ePortal OR TI e-consultation OR AB e-consultation OR TI "Remote Consultation" OR AB "Remote Consultation" OR (MH "Videoconferencing+") OR TI e-care OR AB e-care OR TI eTherap* OR AB eTherap* OR TI e-therap* OR AB e-therap* OR TI teletherap* OR AB teletherap* OR (MH "Telerehabilitation") OR TI telerehabilitation OR AB telerehabilitation OR TI tele-rehabilitation OR AB tele-rehabilitation OR TI tablet* OR TI tablet* OR AB tablet* OR TI android* OR AB android* OR TI Ipad* OR AB ipad* OR TI iphone* OR AB iphone* OR TI ipod* OR AB iphone OR TI iso OR AB iso) OR (MH "Cellular Phone+") OR (MH "Telephone+") OR (MH "Smartphone") OR TI "Mobile phone" OR AB "Mobile phone" OR TI telephone OR AB telephone OR TI smartphone OR AN smartphone OR TI (cell* N1 phone) OR AB (cell* N1 phone) OR (MH "Text Messaging+") OR TI multi-media OR AB multi-media OR TI multimedia OR AB multimedia OR (MH "mobile applications") OR TI app OR AB app OR TI apps OR AB apps OR TI "social media*" OR AB "social media*" OR TI facebook OR AB facebook OR (TI twitter OR AB twitter OR TI tweet* OR AB tweet*)
S1	TI "Physical activity" OR AB "Physical activity" OR (MH "Sports+") OR TI Sport* OR AB Sport* OR TI Work-out OR AB Work-out OR TI Workout OR AB Workout OR TI "Working out" OR AB "Working out" OR TI Aerobics OR AB Aerobics OR TI Fitness OR AB Fitness OR (MH "Physical Fitness+") OR TI "Physical conditioning" OR AB "Physical conditioning" OR TI "Physically fit" OR AB "Physically fit" OR TI "Active lifestyle" OR AB "Active lifestyle" OR (MH "Exercise+") OR TI Exercis* OR AB Exercis* OR AB exercise therapy or TI exercise therapy OR (MH "Therapeutic Exercise+") OR (MH "Locomotion") OR (MH "Physical Therapy+") OR TI Physiotherapy OR AB Physiotherapy OR TI "Physical Therapy" OR AB "Physical Therapy" OR (MH "Physical Education and Training+") OR (MH "Motor Activity+") OR (MH "Resistance Training+") OR TI "Resistance Training" OR AB "Resistance Training" OR TI Strengthening OR AB Strengthening OR "Muscle strength" OR TI "Muscle strength" OR AB "Muscle strength" OR (MH "Walking+") OR TI Walking OR AB Walking OR (MH "Running+") OR TI Running OR AB Running OR TI Jogging OR AB Jogging OR TI Cycling OR AB Cycling OR (MH "Cycling") OR (MH "Bicycles") OR (MH "Aquatic Sports+") OR TI Swimming OR AB Swimming OR (MH "Gymnastics") OR TI gymnastic* OR AB gymnastic* OR (MH "Yoga") OR (MH "Dancing") OR TI pilates OR AB pilates

CENTRAL

#1	("Physical activity"):ti,ab,kw
#2	MeSH descriptor: [Sports] explode all trees
#3	(Sport*):ti,ab,kw
#4	(Work-out):ti,ab,kw
#5	(Workout):ti,ab,kw
#6	("Working out"):ti,ab,kw
#7	(Aerobics):ti,ab,kw
#8	(Fitness):ti,ab,kw
#9	MeSH descriptor: [Physical Fitness] explode all trees
#10	("Physical conditioning"):ti,ab,kw

#11	("Physically fit"):ti,ab,kw
#12	("Active lifestyle"):ti,ab,kw
#13	MeSH descriptor: [Exercise] explode all trees
#14	(Exercis*):ti,ab,kw
#15	MeSH descriptor: [Exercise Movement Techniques] explode all trees
#16	MeSH descriptor: [Exercise Therapy] explode all trees
#17	("Exercise Therapy"):ti,ab,kw
#18	MeSH descriptor: [Locomotion] explode all trees
#19	MeSH descriptor: [Physical Therapy Modalities] explode all trees
#20	(Physiotherapy):ti,ab,kw
#21	("Physical Therapy"):ti,ab,kw
#22	MeSH descriptor: [Physical Education and Training] explode all trees
#23	MeSH descriptor: [Motor Activity] explode all trees
#24	MeSH descriptor: [Resistance Training] explode all trees
#25	("Resistance Training"):ti,ab,kw
#26	(Strengthening):ti,ab,kw
#27	("Muscle strength"):ti,ab,kw
#28	MeSH descriptor: [Walking] explode all trees
#29	(Walking):ti,ab,kw
#30	MeSH descriptor: [Running] explode all trees
#31	(Running):ti,ab,kw
#32	(Jogging):ti,ab,kw
#33	(Cycling):ti,ab,kw
#34	MeSH descriptor: [Bicycling] explode all trees
#35	MeSH descriptor: [Swimming] explode all trees
#36	(Swimming):ti,ab,kw
#37	MeSH descriptor: [Gymnastics] explode all trees
#38	(gymnastic*):ti,ab,kw
#39	MeSH descriptor: [Yoga] explode all trees
#40	MeSH descriptor: [Dancing] explode all trees
#41	(pilates):ti,ab,kw
#42	#1 OR #2 OR #3 OR #4 OR #5 OR #6 OR #7 OR #8 OR #9 OR #10 OR #11 OR #12 OR #13 OR #14 OR #15 OR #16 OR #17 OR #18 OR #19 OR #20 OR #21 OR #22 OR #23 OR #24 OR #25 OR #26 OR #27 OR #28 OR #29 OR #30 OR #31 OR #32 OR #33 OR #34 OR #35 OR #36 OR #37 OR #38 OR #39 OR #40 OR #41
#43	("digital health"):ti,ab,kw
#44	MeSH descriptor: [Telemedicine] explode all trees
#45	(telemedicine OR tele-medicine):ti,ab,kw
#46	(telecare OR tele-care):ti,ab,kw
#47	(telehomecare OR tele-homecare):ti,ab,kw
#48	(telehealthcare OR tele-healthcare):ti,ab,kw
#49	(telemonitoring OR tele-monitoring):ti,ab,kw
#50	(telehealth OR tele-health):ti,ab,kw

#51	MeSH descriptor: [Telecommunications] explode all trees
#52	(ehealth OR e-health):ti,ab,kw
#53	("electronic health*"):ti,ab,kw
#54	(mhealth OR m-health):ti,ab,kw
#55	("mobile health"):ti,ab,kw
#56	MeSH descriptor: [Electronic Mail] explode all trees
#57	(e-mail* OR email*):ti,ab,kw
#58	("e-mail based" OR e-mail-based OR email-based OR "email based"):ti,ab,kw
#59	MeSH descriptor: [Internet] explode all trees
#60	MeSH descriptor: [Internet-Based Intervention] explode all trees
#61	(Internet-based OR web-based):ti,ab,kw
#62	MeSH descriptor: [Computer-Assisted Instruction] explode all trees
#63	MeSH descriptor: [Programmed Instructions as Topic] explode all trees
#64	MeSH descriptor: [Communications Media] explode all trees
#65	("on-line communication" OR "online communication"):ti,ab,kw
#66	(e-Portal OR eportal):ti,ab,kw
#67	("Computer System"):ti,ab,kw
#68	MeSH descriptor: [Computers] explode all trees
#69	(e-consultation):ti,ab,kw
#70	("Remote Consultation"):ti,ab,kw
#71	(e-care):ti,ab,kw
#72	(eTherap* OR e-Therap*):ti,ab,kw
#73	(teletherap*):ti,ab,kw
#74	MeSH descriptor: [Telerehabilitation] explode all trees
#75	(telerehabilitation OR tele-rehabilitation):ti,ab,kw
#76	MeSH descriptor: [Microcomputers] explode all trees
#77	(Computers OR tablet* OR android*):ti,ab,kw
#78	(Ipad* OR iphone* OR ipod* OR iso):ti,ab,kw
#79	MeSH descriptor: [Cell Phone] explode all trees
#80	MeSH descriptor: [Telephone] explode all trees
#81	MeSH descriptor: [Smartphone] 1 tree(s) exploded
#82	("Mobile phone" OR telephone OR smartphone OR "cell* ajd1 phone"):ti,ab,kw
#83	((sms or mms or text) and messag*):ti,ab,kw
#84	MeSH descriptor: [Text Messaging] explode all trees
#85	(multi-media OR multimedia):ti,ab,kw
#86	MeSH descriptor: [Mobile Applications] explode all trees
#87	(smartphone NEAR/3 app*):ti,ab,kw
#88	(app OR apps):ti,ab,kw
#89	("social media*"):ti,ab,kw
#90	(facebook):ti,ab,kw
#91	(twitter OR tweet*):ti,ab,kw
#92	#43 #44 # 45 #46 OR #47 OR #48 OR #49 OR #50 OR #51 OR #52 OR #53 OR #54 OR #55 OR #56 OR #57 OR #58 OR #59 OR #60 OR #61 OR #62 OR #63 OR #64 OR #65 OR #66 OR #67 OR #68 OR

	#69 OR #70 OR #71 OR #72 OR #73 OR #74 OR #75 OR #76 OR #77 OR #78 OR #79 OR #80 OR #81 OR #82 OR #83 OR #84 OR #85 OR #86 OR #87 OR #88 OR #89 OR #90 OR #91
#93	MeSH descriptor: [Multimorbidity] explode all trees
#94	(Multimorbid* OR Multi-morbid*):ti,ab,kw
#95	(Multidisease? OR Multi-disease? OR "Multiple disease?"):ti,ab,kw
#96	(Multicondition?):ti,ab,kw
#97	((Multi or multiple) NEAR/2 (morbid* or disease? or condition? or ill* OR syndrom* or symptom* or disorder? or health)):ti,ab,kw
#98	((Associated or Co-existing or Concurrent or Cooccurring or Chronic*) NEAR (morbid* or disease? or condition? or ill* OR syndrom* or symptom* or disorder? or health)):ti,ab,kw
#99	(Polypathology OR Pluripathology):ti,ab,kw
#100	MeSH descriptor: [Comorbidity] explode all trees
#101	(Comorbid* OR Co-morbid*):ti,ab,kw
#102	(Pattern* NEAR/2 disease?):ti,ab,kw
#103	(Cluster* NEAR/2 disease?):ti,ab,kw
#104	MeSH descriptor: [Multiple Chronic Conditions] explode all trees
#105	MeSH descriptor: [Noncommunicable Diseases] explode all trees
#106	#93 OR #94 OR #95 OR #96 OR #97 OR #98 OR #99 OR #100 OR #101 OR #102 OR #103 OR #104 OR #105
#107	MeSH descriptor: [Hypertension] explode all trees
#108	hypertension:ti,ab,kw
#109	hypertens*:ti,ab,kw
#110	("high blood pressure"):ti,ab,kw
#111	#107 OR #108 OR #109 OR #110
#112	MeSH descriptor: [Myocardial Ischemia] explode all trees
#113	MeSH descriptor: [Coronary Disease] explode all trees
#114	MeSH descriptor: [Acute Coronary Syndrome] explode all trees
#115	MeSH descriptor: [Myocardial Infarction] explode all trees
#116	MeSH descriptor: [Coronary Artery Disease] explode all trees
#117	(myocard* NEAR (ischem* or ischaem* or infact*)):ti,ab,kw
#118	((ischem* or ischaem*) NEAR heart disease):ti,ab,kw
#119	#112 OR #113 OR #114 OR #115 OR #116 OR #117 OR #118
#120	MeSH descriptor: [Heart Failure] explode all trees
#121	("Heart Failure"):ti,ab,kw
#122	#120 OR #121
#123	MeSH descriptor: [Pulmonary Disease, Chronic Obstructive] explode all trees
#124	(COPD):ti,ab,kw
#125	("Chronic Obstructive Lung Disease"):ti,ab,kw
#126	Chronic bronchitis:ti,ab,kw
#127	Pulmonary emphysema:ti,ab,kw
#128	MeSH descriptor: [Bronchitis] explode all trees
#129	MeSH descriptor: [Emphysema] explode all trees
#130	#123 OR #124 OR #125 OR #126 OR #127 OR #128 OR #129
#131	MeSH descriptor: [Depression] explode all trees

#132	Depress*:ti,ab,kw
#133	MeSH descriptor: [Depressive Disorder, Major] explode all trees
#134	MeSH descriptor: [Dysthymic Disorder] explode all trees
#135	dysthymic disorder:ti,ab,kw
#136	dysthymi*:ti,ab,kw
#137	affect* disorder:ti,ab,kw
#138	#131 OR #132 OR #133 OR #134 OR #135 OR #136 OR #137
#139	MeSH descriptor: [Diabetes Mellitus] explode all trees
#140	MeSH descriptor: [Diabetes Mellitus, Type 2] explode all trees
#141	Non-Insulin-Dependent Diabetes Mellitus:ti,ab,kw
#142	NIDDM:ti,ab,kw
#143	diabetes mellitus:ti,ab,kw
#144	#139 OR #140 #141 OR #142 OR #143
#145	MeSH descriptor: [Osteoarthritis] explode all trees
#146	Osteoarthritis:ti,ab,kw
#147	Osteoarthr*:ti,ab,kw
#148	(degenerative NEAR/2 arthritis):ti,ab,kw
#149	arthrosis:ti,ab,kw
#150	MeSH descriptor: [Osteoarthritis, Knee] explode all trees
#151	MeSH descriptor: [Osteoarthritis, Hip] explode all trees
#152	#145 OR #146 OR #147 OR #148 OR #149 OR #150 OR #151
#153	MeSH descriptor: [Anxiety Disorders] explode all trees
#154	MeSH descriptor: [Anxiety] explode all trees
#155	Anxiety:ti,ab,kw
#156	#153 OR #154 OR #155
#157	#106 OR #111 OR #119 OR #122 OR #130 OR #138 OR #144 OR #152 OR #156
#158	#42 AND #92 AND #157

Supplementary Table 2. List of excluded full-text studies and reason for exclusion	
**Study**	**Reason for exclusion**
Ababneh, A. et al. Usability and effectiveness of a self care mhealth app in heart failure: a preliminary report. (2021). Cardiopulmonary physical therapy journal	Abstract, protocol, or trial registration
Actrn, A healthy mind in a healthy body: a two-group randomised pilot trial evaluating web-based physical activity interventions to improve mental and physical health in people with depression. (2013). http://www.who.int/trialsearch/Trial2.aspx?TrialID=ACTRN12613001215718	Abstract, protocol, or trial registration
Actrn, A micro randomised trial (MRT) to test the effects of app-based motivational messages on physical activity and heart rate variability. (2022). https://trialsearch.who.int/Trial2.aspx?TrialID=ACTRN12622000731796	Abstract, protocol, or trial registration
Actrn, Evaluation of an online support program for Type 2 diabetes self-management and dysphoria (depression, anxiety, and diabetes-specific distress). (2012). http://www.who.int/trialsearch/Trial2.aspx?TrialID=ACTRN12612000620820	Abstract, protocol, or trial registration
Actrn, Heart Exercise And Remote Technologies Trial. (2011). http://www.who.int/trialsearch/Trial2.aspx?TrialID=ACTRN12611000117910	Abstract, protocol, or trial registration
Actrn, Telerehabilitation in people with Chronic Obstructive Pulmonary Disease (COPD). (2012). http://www.who.int/trialsearch/Trial2.aspx?TrialID=ACTRN12612001263886	Abstract, protocol, or trial registration
Anderson, D. et al. Managing the space between visits: Telephonic disease management for underserved patients with diabetes. (2010). Journal of general internal medicine	Abstract, protocol, or trial registration
Baker, K. et al. Automated telephone-linked communication: a novel approach to enhance long-term adherence to resistance training exercise among people with knee osteoarthritis. (2013). Arthritis and rheumatism.	Abstract, protocol, or trial registration
Baker, K.et al. A randomized trial of automated telephone-linked communication to improve exercise adherence for a progressive resistance training program in people with knee osteoarthritis. (2016). Arthritis & rheumatology	Abstract, protocol, or trial registration
Banos, R. M. et al. An internet-based self-administered intervention for promoting healthy habits and weight loss in hypertensive people who are overweight or obese: a randomized controlled trial. (2015). BMC cardiovascular disorders	Abstract, protocol, or trial registration
Barnes, A. et al. Purposeful physical activity in COPD patients comparing standard and web based pulmonary rehabilitation. (2016). European respiratory journal	Abstract, protocol, or trial registration
Bartlett, A. S. et al. The impact of an aerobic and resistive exercise program with telehealth monitoring on quality of life in individuals at risk for type 2 diabetes. (2011). Cardiopulmonary Physical Therapy Journal (American Physical Therapy Association, Cardiopulmonary Section)	Abstract, protocol, or trial registration
Bennell, K. L. et al. Comparing video-based telehealth-delivered exercise and weight loss programs with online education on outcomes of knee osteoarthritis: a randomized trial. (2022). Osteoarthritis and cartilage	Abstract, protocol, or trial registration
Benzo, R. et al. Randomized study of home-based rehabilitation with health coaching in chronic obstructive pulmonary disease. (2020). American journal of respiratory and critical care medicine	Abstract, protocol, or trial registration
Benzo, R. P. et al. Home rehabilitation for copd: a randomized study of remote activity monitoring and health coaching. (2022). Chest	Abstract, protocol, or trial registration
Bonn, S. E. et al. App-technology to increase physical activity among patients with diabetes type 2 - the DiaCert-study, a randomized controlled trial. (2018). BMC public health	Abstract, protocol, or trial registration
Brouwers, R. et al. Effectiveness of cardiac telerehabilitation with relapse prevention compared to centrebased cardiac rehabilitation: Results from the Smart Care-CAD randomised controlled trial. (2021). European journal of preventive cardiology	Abstract, protocol, or trial registration

Burner, E. et al. 211 Long Term Effects of Mobile Health and Augmented Social Support on Emergency Department Patients With Diabetes. (2021). Annals of emergency medicine	Abstract, protocol, or trial registration
Cameron-Tucker, H. et al. A randomized controlled trial of telephone health-mentoring including home-based walking (TELE-REHAB) before Group rehabilitation versus usual care and subsequent group rehabilitation (Group-Rehab). (2014). Respirology (Carlton, Vic.)	Abstract, protocol, or trial registration
Celano, C. M. et al. A Positive Psychology-Based Health Behavior Intervention in Heart Failure: results from the Reach for Health Pilot Trial. (2020). Psychosomatic medicine	Abstract, protocol, or trial registration
ChiCtr, The effects of telerehabilitation on patients with knee osteoarthritis: a randomized controlled clinical trial. (2019). http://www.who.int/trialsearch/Trial2.aspx?TrialID=ChiCTR1900022757	Abstract, protocol, or trial registration
Connelly, J. et al. Using the internet to effectively promote physical activity in people with Type 2 diabetes in the Highlands: DAVE (Diabetes and Virtual Exercise). (2015). Diabetic medicine.	Abstract, protocol, or trial registration
Coultas, D. et al. A lifestyle physical activity intervention for COPD improved mental health-related quality of life among severely impaired patients. (2017). American journal of respiratory and critical care medicine	Abstract, protocol, or trial registration
Ctri, A study to assess the effectiveness of a mobile app based intervention to improve activity, pain and movement of knee in patient with Knee Osteoarthritis attending Orthopaedic OPD, PGIMER, Chandigarh. (2019). http://www.who.int/trialsearch/Trial2.aspx?TrialID=CTRI/2019/07/019989	Abstract, protocol, or trial registration
De Las Heras, J. C. et al. Is Virtual Autonomous Physiotherapist Tele-rehabilitation Program in Chronic Obstructive Pulmonary Disease equal to Hospital-Based Pulmonary Rehabilitation?. (2020). European respiratory journal	Abstract, protocol, or trial registration
Dedier, J. J. et al. Randomized controlled trial of a culturally adapted, automated telephone exercise coach to improve physical activityamong hypertensive African-Americans. (2014). Journal of general internal medicine	Abstract, protocol, or trial registration
Dor-Haim, H. et al. A Novel Digital Platform for a Monitored Home-based Cardiac Rehabilitation Program. (2019). Journal of visualized experiments : JoVE	Abstract, protocol, or trial registration
Drks, Pro-Active Rehabilitation and Telephone Intervention in Type 2 Diabetics: a randomized controlled trial of patients enrolled in a DMP. (2008). http://www.who.int/trialsearch/Trial2.aspx?TrialID=DRKS00000058	Abstract, protocol, or trial registration
Drks, The effectiveness of smartphone app guided interval-walking training compared to continuous aerobic walking training for people with type 2 diabetes or prediabetes. A pilot study. (2019). http://www.who.int/trialsearch/Trial2.aspx?TrialID=DRKS00016370	Abstract, protocol, or trial registration
Drks, The usability and preliminary effectiveness of an app-based training and educational program (Join2Move) in people with hip and/ or knee osteoarthritis – a randomized controlled pilot study. (2021). https://trialsearch.who.int/Trial2.aspx?TrialID=DRKS00027164	Abstract, protocol, or trial registration
Dunkel, A. et al. Telemedically assisted lifestyle intervention to improve glycemic control and self-management in type 2 diabetics. (2020). Diabetes technology & therapeutics	Abstract, protocol, or trial registration
Dunn, S. L. et al. Improving physical activity and decreasing hopelessness in patients with ischemic heart disease: testing of the heart up! Intervention. (2017). Circulation	Abstract, protocol, or trial registration
Duruturk, N. et al. Effects of Telerehabilitation on Dyspnea and Exercise Capacity in Patients with Type 2 Diabetes Mellitus Who Smoked. (2017). European respiratory journal	Abstract, protocol, or trial registration
Eakin, E. G. et al. Living Well with Diabetes: a randomized controlled trial of a telephone-delivered intervention for maintenance of weight loss, physical activity and glycaemic control in adults with type 2 diabetes. (2010). BMC public health	Abstract, protocol, or trial registration

Evangelista, . LS. et al. Reduced body weight, improved physical activity and healthier eating with fitness intensive therapy (get FIT) vs. get fit+: a feasibility study. (2018). Journal of heart and lung transplantation	Abstract, protocol, or trial registration
Feldman, D. I. et al. Physical activity tracking studies: the effect of an entirely mobile (mhealth) platform for data capture. (2014). Circulation	Abstract, protocol, or trial registration
Frederix, I. et al. Do we keep cardiac patients out of hospital by adding telerehabilitation to standard rehabilitation?. (2015). Cardiology (switzerland)	Abstract, protocol, or trial registration
Frederix, I. et al. How to keep coronary artery disease patients active after the acute rehabilitation phase? the value of an internet-based telerehabilitation programme. (2012). Acta cardiologica	Abstract, protocol, or trial registration
Frederix, I. et al. Increasing the medium-term clinical benefits of hospital-based cardiac rehabilitation by physical activity telemonitoring in coronary artery disease patients. (2015). European journal of preventive cardiology	Abstract, protocol, or trial registration
Frei, A. et al. Effectiveness of a long-term home-based maintenance exercise training program using minimal equipment vs. usual care in COPD Patients: HOMEX-1 randomized controlled trial. (2021). American journal of respiratory and critical care medicine	Abstract, protocol, or trial registration
Galdiz, J. B. et al. Telerehabilitation Programme as a Maintenance Strategy for COPD Patients: A 12-Month Randomized Clinical Trial. (2020). Archivos de bronconeumologia	Abstract, protocol, or trial registration
Gloeckl, R. et al. Influence of Adherence to an App-Based Pulmonary Rehabilitation Maintenance Program on Physical Activity and Quality of Life in COPD Patients - a Subgroup Analysis of a Randomized Controlled Trial. (2022). American journal of respiratory and critical care medicine	Abstract, protocol, or trial registration
Glynn, L. G. et al. Effectiveness of a smartphone application to promote physical activity in primary care: randomised controlled trial. (2014). European journal of general practice	Abstract, protocol, or trial registration
Gohir, S. et al. A Randomised Controlled Trial Evaluating the Efficacy of Internet-based Exercises Aimed at Treating Knee Osteoarthritis (iBEAT-OA). (2020). Arthritis and rheumatology	Abstract, protocol, or trial registration
Grant, E. et al. Engagement and outcomes among older adults with mobile health (mHealth) cardiac rehabilitation: pilot study. (2018). Journal of the American Geriatrics Society	Abstract, protocol, or trial registration
Hansen, H. et al. 1-year follow-up of pulmonary tele-rehabilitation versus conventional pulmonary rehabilitation: a multicenter, single blinded, superiority RCT. (2019). European respiratory journal	Abstract, protocol, or trial registration
Hansen, H. et al. Pulmonary tele-rehabilitation versus conventional pulmonary rehabilitation a multicenter, single blinded, superiority rct. (2019). American journal of respiratory and critical care medicine	Abstract, protocol, or trial registration
Holmen, H. et al. Patient-reported outcomes and the use of a diabetes diary mobile application to attain lifestyle changes for persons with type 2 diabetes. (2015). Quality of Life Research	Abstract, protocol, or trial registration
Hornikx, M. et al. The effects of physical activity coaching in patients with COPD after an acute exacerbation. (2014). European respiratory journal	Abstract, protocol, or trial registration
Huo, X. H. et al. Effects of mobile text messaging on glycemic control in patients with coronary heart disease and diabetes mellitus: a randomized controlled trial. (2019). European heart journal	Abstract, protocol, or trial registration
Huq, F. N. et al. Effect of telephone-monitored home-based cardiac rehabilitation exercise on functional capacity and quality of life in heart failure patients in a lower-middle-income country. (2022). European journal of preventive cardiology	Abstract, protocol, or trial registration
Isrctn, A 12-week digital care program for chronic knee pain involving exercise, education, and psychosocial support. (2017). http://www.who.int/trialsearch/Trial2.aspx?TrialID=ISRCTN13307390	Abstract, protocol, or trial registration

Isrctn, A randomised controlled trial of the effect of exercise training on exercise capacity in older patients with heart failure. (2007). http://www.who.int/trialsearch/Trial2.aspx?TrialID=ISRCTN51615566	Abstract, protocol, or trial registration
Jehn, M. et al. Impact of climate change in patients with COPD: results of telemedical patient monitoring. (2013). American journal of respiratory and critical care medicine	Abstract, protocol, or trial registration
Jiang, Y. et al. HEART exercise and remote technologies (HEART): a randomized controlled trial. (2013). Clinical trials (London, England)	Abstract, protocol, or trial registration
Jolly, K. et al. Telephone health coaching in primary care patients with MRC I/II COPD: randomised controlled trial. (2017). European respiratory journal	Abstract, protocol, or trial registration
Krause, K. et al. An individualized proactive e-health intervention promoting a lifestyle against depression: results of a randomized controlled trial over 24 months. (2021). Gesundheitswesen, supplement	Abstract, protocol, or trial registration
Kyriakou, M. et al. Supportive care reinforces telemonitoring in heart failure patients: pilot results of the Support Heart. (2021). European heart journal	Abstract, protocol, or trial registration
Martinez, C. H. et al. Taking Healthy Steps: rationale, design and baseline characteristics of a randomized trial of a pedometer-based Internet-mediated walking program in veterans with chronic obstructive pulmonary disease. (2014). BMC pulmonary medicine	Abstract, protocol, or trial registration
Nct, RCT of Sensor-controlled Digital Game for Heart Failure Self-management. (2021). https://clinicaltrials.gov/show/NCT05056129	Abstract, protocol, or trial registration
Piette, J. et al. 12-month outcomes from a randomized trial of telephone cognitive behavioral therapy for depressed patients with type 2 diabetes. (2010). Journal of general internal medicine	Abstract, protocol, or trial registration
Piotrowicz, E. et al. Hybrid telerehabilitation and telecare is more efffective than usual care in improving peak vo2 and 6-minute walk distance but is not associated with reduction of 24-months mortality except in the most experienced center -result of the first randomized, prospective, open-label, parallel group, controlled, multi-center-telereh-HF study. (2019). Circulation	Abstract, protocol, or trial registration
Piotrowicz, E. et al. Relationships between physical capacity and depression in heart failure patients undergoing hybrid comprehensive telerehabilitation vs usual care results of the TELEREH-HF randomized clinical trial. (2021). European heart journal	Abstract, protocol, or trial registration
Priori, R. et al. Automated coaching for physical activity in COPD patients: results from a pilot study. (2017). European respiratory journal	Abstract, protocol, or trial registration
Reid, R. D. et al. The cardiofit expert system to prescribe and track physical activity in patients with coronary artery disease (CAD): Results from a randomized controlled trial. (2008). Journal of cardiopulmonary rehabilitation and prevention	Abstract, protocol, or trial registration
Robinson, S. A. et al. Results from a multi-site web-based physical activity intervention in COPD: between group and site differences. (2020). American journal of respiratory and critical care medicine	Abstract, protocol, or trial registration
Robinson, S. A. et al. A web-based physical activity intervention benefits persons with copd and low self-efficacy: a randomized controlled trial. (2019). American journal of respiratory and critical care medicine	Abstract, protocol, or trial registration
Robinson, S. A. et al. Baseline functional status is associated with response to a web-based physical activity intervention in copd. (2021). American journal of respiratory and critical care medicine	Abstract, protocol, or trial registration
Scalvini, S. et al. Multidisciplinary telehealth program for patients affected by chronic heart failure and chronic obstructive pulmonary disease. (2016). European journal of heart failure.	Abstract, protocol, or trial registration
Sears, L. E. et al. One drop digital app and coaching improves lifestyle risks, glycemic control and psychological wellbeing in people with hypertension and type 2 diabetes. (2021). Circulation	Abstract, protocol, or trial registration

Semanik, P. et al. Using Fitbits. Fitabase®, and remote coaching to increase physical activity in employees with knee osteoarthritis symptoms. (2017). Arthritis & rheumatology	Abstract, protocol, or trial registration
Sengupta, S. P. et al. Haemodynamic effects of a personalized, home-based physical activity intervention for heart failure with preserved ejection fraction. (2021). European journal of heart failure	Abstract, protocol, or trial registration
Shapiro, S. et al. Smartphone technology versus paper-based logs for type II diabetes prevention: psychological and behavioral outcomes. (2011). Canadian journal of cardiology.	Abstract, protocol, or trial registration
Sharma, A. et al. Utilizing mobile technologies to improve physical activity and medication adherence in patients with heart failure and diabetes mellitus: rationale and design of the TARGET-HF-DM Trial. (2019). American heart journal	Abstract, protocol, or trial registration
Skrepnik, N. et al. Impact of novel smartphone application on pain and mobility in osteoarthritis patients treated with hylan g-f 20. (2016). Arthritis & rheumatology	Abstract, protocol, or trial registration
Stenlund, T. et al. Web-based support for self-management strategies versus usual care for people with COPD: 3 months follow up in a randomised controlled trial. (2021). European respiratory journal	Abstract, protocol, or trial registration
Sylvia, L. et al. Effectiveness of online psychotherapies to increase physical activity in individuals with depression. (2021). Bipolar Disorders	Abstract, protocol, or trial registration
Tamban, C. A. et al. A randomized controlled trial on the use of short message services for improving adherence to diet and exercise among patients with type 2 diabetes mellitus at the university of the Philippines-Philippine general hospital diabetes clinic. (2013). Endocrine reviews	Abstract, protocol, or trial registration
Tsai, L. et al. Telerehabilitation improves exercise capacity and quality of life in people with chronic obstructive pulmonary disease (COPD): a randomised controlled trial. (2016). Respirology (Carlton, Vic.)	Abstract, protocol, or trial registration
Tsai, L. et al. Telerehabilitation in people with chronic obstructive pulmonary disease (COPD): a randomised controlled trial. (2016). European respiratory journal	Abstract, protocol, or trial registration
Tsygankova, O. et al. Effect of mobile applications for physical activity tracking on exercise tolerance in patients with hypertension and coronary artery disease. (2022). Journal of hypertension	Abstract, protocol, or trial registration
Vasilopoulou, M. et al. Effectiveness of home telerehabilitation on functional capacity and daily physical activity in COPD patients. (2015). European respiratory journal. (var.pagings)	Abstract, protocol, or trial registration
Vonk, T. et al. Effect of a personalised mHealth home-based training application on physical activity levels during and after centre-based cardiac rehabilitation: rationale and design of the Cardiac RehApp randomised control trial. (2021). BMJ open sport and exercise medicine	Abstract, protocol, or trial registration
Walters, J. A. E. et al. Effect of health-mentoring to increase daily physical activity in chronic obstructive pulmonary disease (COPD). (2012). Respirology (Carlton, Vic.)	Abstract, protocol, or trial registration
Wijsman, C. A. et al. Effects of a web-based intervention on physical activity and metabolism in older adults: randomized controlled trial. (2015). Diabetes technology & therapeutics	Abstract, protocol, or trial registration
Zhou, H. et al. Effect of a web-based management platform on hypertension control in community: a randomized clinical trial. (2022). Journal of hypertension	Abstract, protocol, or trial registration
Abu-Saad, K. et al. Development and Efficacy of an Electronic, Culturally Adapted Lifestyle Counseling Tool for Improving Diabetes-Related Dietary Knowledge: randomized Controlled Trial Among Ethnic Minority Adults With Type 2 Diabetes Mellitus. (2019). Journal of medical Internet research	Wrong comparator
Alasfour, M. et al. The effect of innovative smartphone application on adherence to a home-based exercise programs for female older adults with knee osteoarthritis in Saudi Arabia: a randomized controlled trial. (2020). Disability and rehabilitation	Wrong comparator

Andreae, S. J. et al. Peer-Delivered Cognitive Behavioral Training to Improve Functioning in Patients With Diabetes: A Cluster-Randomized Trial. (2020). Annals of family medicine	Wrong comparator
Antypas, K. et al. An Internet- and mobile-based tailored intervention to enhance maintenance of physical activity after cardiac rehabilitation: short-term results of a randomized controlled trial. (2014). Journal of medical Internet research	Wrong comparator
Bourne, S. et al. Online versus face-to-face pulmonary rehabilitation for patients with chronic obstructive pulmonary disease: randomised controlled trial. (2017). BMJ open	Wrong comparator
Chaplin, E. et al. An interactive web-based pulmonary rehabilitation programme: a randomised controlled feasibility trial. (2016). European respiratory journal	Wrong comparator
Edelman, D. et al. Nurse-led behavioral management of diabetes and hypertension in community practices: a randomized trial. (2015). Journal of general internal medicine	Wrong comparator
Godtfredsen, N. et al. 12-months follow-up of pulmonary tele-rehabilitation versus standard pulmonary rehabilitation: A multicentre randomised clinical trial in patients with severe COPD. (2020). Respiratory medicine	Wrong comparator
Hansen, H. et al. Supervised pulmonary tele-rehabilitation versus pulmonary rehabilitation in severe COPD: a randomised multicentre trial. (2020). Thorax	Wrong comparator
Holmes-Rovner, M. et al. Does outpatient telephone coaching add to hospital quality improvement following hospitalization for acute coronary syndrome?. (2008). Journal of general internal medicine	Wrong comparator
Hwang R. et al. Home-based telerehabilitation is not inferior to a centre-based program in<br>patients with chronic heart failure: a randomised trial. (2017). J Physiother	Wrong comparator
Johnston, N. et al. Effects of interactive patient smartphone support app on drug adherence and lifestyle changes in myocardial infarction patients: A randomized study. (2016). American heart journal	Wrong comparator
Kloek, C. et al. Effectiveness of a Blended Physical Therapist Intervention in People With Hip Osteoarthritis, Knee Osteoarthritis, or Both: A Cluster-Randomized Controlled Trial. (2018). Physical therapy	Wrong comparator
Kraal, J. J. et al. Clinical and cost-effectiveness of home-based cardiac rehabilitation compared to conventional, centre-based cardiac rehabilitation: Results of the FIT@Home study. (2017). European journal of preventive cardiology	Wrong comparator
Lawford, B, J. et al. Moderators of Effects of Internet-Delivered Exercise and Pain Coping Skills Training for People With Knee Osteoarthritis: Exploratory Analysis of the IMPACT Randomized Controlled Trial. (2018). Journal of medical Internet research	Wrong comparator
Lawler, S. P. et al. Moderators of health behavior initiation and maintenance in a randomized telephone counseling trial. (2014). Preventive medicine	Wrong comparator
Lim, S. et al. Multifactorial intervention in diabetes care using real-time monitoring and tailored feedback in type 2 diabetes. (2016). Acta diabetologica	Wrong comparator
Liu, F. et al. Effects of an animated diagram and video-based online breathing program for dyspnea in patients with stable COPD. (2013). Patient preference and adherence	Wrong comparator
MacPherson, M. M. et al. Effects of Mobile Health Prompts on Self-Monitoring and Exercise Behaviors Following a Diabetes Prevention Program: Secondary Analysis From a Randomized Controlled Trial. (2019). JMIR mHealth and uHealth	Wrong comparator
Marios, T. et al. The Effect of Tele-Monitoring on Exercise Training Adherence, Functional Capacity, Quality of Life and Glycemic Control in Patients With Type II Diabetes. (2012). Journal of sports science & medicine	Wrong comparator
McKay, H. G. et al. The diabetes network internet-based physical activity intervention: a randomized pilot study. (2001). Diabetes care	Wrong comparator
Mecklenburg G. et al. Effects of a 12-Week Digital Care Program for Chronic Knee Pain on Pain, Mobility, and Surgery Risk: Randomized Controlled Trial. (2018). J Med Internet	Wrong comparator

Nguyen, H. Q. et al. Pilot study of a cell phone-based exercise persistence intervention post-rehabilitation for COPD. (2009). International journal of chronic obstructive pulmonary disease	Wrong comparator
Nguyen, H. Q. et al. Randomized controlled trial of an internet-based versus face-to-face dyspnea self-management program for patients with chronic obstructive pulmonary disease: pilot study. (2008). Journal of medical Internet research	Wrong comparator
Odole, A. C et al. A Telephone-based Physiotherapy Intervention for Patients with Osteoarthritis of the Knee. (2013). International journal of telerehabilitation	Wrong comparator
Park, S. K. et al. Evaluating the effect of a smartphone app-based self-management program for people with COPD: A randomized controlled trial. (2020). Applied nursing research : ANR	Wrong comparator
Patnaik, L. et al. Effectiveness of mobile application for promotion of physical activity among newly diagnosed patients of type II diabetes -A randomized controlled trial. (2022). International journal of preventive medicine	Wrong comparator
Ramirez M. et al. Phone Messaging to Prompt Physical Activity and Social Support Among Low-Income Latino Patients With Type 2 Diabetes: A Randomized Pilot Study. (2017). JMIR diabetes	Wrong comparator
Rebar A. et al. Healthy mind, healthy body: a randomized trial testing the efficacy of a computer-tailored vs. interactive web-based intervention for increasing physical activity and reducing depressive symptoms. (2016). Mental health and physical activity. 11 (pp 29-37), 2016. Date of publication: 01 oct 2016.	Wrong comparator
Saleh, Z. T. et al. Effect of a Home-Based Mobile Health App Intervention on Physical Activity Levels in Patients With Heart Failure: a Randomized Controlled Trial. (2022). Journal of cardiovascular nursing	Wrong comparator
Santiworakul, A. et al. A home-based multimedia pulmonary rehabilitation program improves clinical symptoms and physical performance of patients with chronic obstructive pulmonary disease. (2021). International journal of environmental research and public health	Wrong comparator
Schlenk, E. A. et al. Promoting Physical Activity in Older Adults With Knee Osteoarthritis and Hypertension: a Randomized Controlled Trial. (2020). Journal of aging and physical activity	Wrong comparator
Schlenk, E. A. et al. Promoting Physical Activity in Older Adults With Knee Osteoarthritis and Hypertension: A Randomized Controlled Trial. (2021). Journal of aging and physical activity	Wrong comparator
Skrepnik, N. et al. Assessing the Impact of a Novel Smartphone Application Compared With Standard Follow-Up on Mobility of Patients With Knee Osteoarthritis Following Treatment With Hylan G-F 20: A Randomized Controlled Trial. (2017). JMIR mHealth and uHealth	Wrong comparator
Stanton, T. R. et al. A pain science education and walking program to increase physical activity in people with symptomatic knee osteoarthritis: a feasibility study. (2020). Pain reports	Wrong comparator
Swoboda, S. M. et al. Setting Single or Multiple Goals for Diet and Physical Activity Behaviors Improves Cardiovascular Disease Risk Factors in Adults With Type 2 Diabetes: A Pragmatic Pilot Randomized Trial. (2016). The Diabetes educator	Wrong comparator
Thiengwittayaporn, S. et al. Development of a mobile application to improve exercise accuracy and quality of life in knee osteoarthritis patients: a randomized controlled trial. (2021). Archives of orthopaedic and trauma surgery	Wrong comparator
Thorsen, I. K. et al. The Effects of a Lifestyle Intervention Supported by the InterWalk Smartphone App on Increasing Physical Activity Among Persons With Type 2 Diabetes: Parallel-Group, Randomized Trial. (2022). JMIR mHealth and uHealth	Wrong comparator
Timurtas, E. et al. Technology-based and supervised exercise interventions for individuals with type 2 diabetes: randomized controlled trial. (2021). Primary care diabetes	Wrong comparator

Valentiner, L. S. et al. Effect of ecological momentary assessment, goal-setting and personalized phone-calls on adherence to interval walking training using the InterWalk application among patients with type 2 diabetes-A pilot randomized controlled trial. (2019). PloS one	Wrong comparator
Varnfield, M. et al. Smartphone-based home care model improved use of cardiac rehabilitation in postmyocardial infarction patients: results from a randomised controlled trial. (2014). Heart (British Cardiac Society)	Wrong comparator
Wang X. et al. The role of text messaging intervention inInner Mongolia among patients with type 2diabetes mellitus: a randomized controlledtrial. (2020). BMC Med Inform Decis Mak	Wrong comparator
Wang, C-H. et al. Mobile-phone-based home exercise training program decreases systemic inflammation in COPD: a pilot study. (2014). BMC pulmonary medicine	Wrong comparator
Yom-Tov, E. et al. Encouraging Physical Activity in Patients With Diabetes: Intervention Using a Reinforcement Learning System. (2017). Journal of medical Internet research	Wrong comparator
Actrn, Text4Heart: a text message and Internet-based comprehensive cardiac rehabilitation intervention. (2013). http://www.who.int/trialsearch/Trial2.aspx?TrialID=ACTRN12613000901707	Wrong patient population
Bae, J-W. et al. mHealth Interventions for Lifestyle and Risk Factor Modification in Coronary Heart Disease: Randomized Controlled Trial. (2021). JMIR mHealth and uHealth	Wrong patient population
Bai, Y. et al. A randomized trial to promote physical activity in adult pre-hypertensive and hypertensive patients. (2022). Journal of Sports Sciences	Wrong patient population
Batalik, L. et al. Long-term exercise effects after cardiac telerehabilitation in patients with coronary artery disease: 1-year follow-up results of the randomized study. (2021). European journal of physical and rehabilitation medicine	Wrong patient population
Batalik, L. et al. Long-term exercise effects after cardiac telerehabilitation in patients with coronary artery disease: 1-year follow-up results of the randomized study. (2021). European journal of physical and rehabilitation medicine	Wrong patient population
Chiang, S-L. et al. Effectiveness of a Home-Based Telehealth Exercise Training Program for Patients With Cardiometabolic Multimorbidity: A Randomized Controlled Trial. (2020). The Journal of cardiovascular nursing	Wrong patient population
Dale, L. P. et al. Text Message and Internet Support for Coronary Heart Disease Self-Management: Results From the Text4Heart Randomized Controlled Trial. (2015). Journal of medical Internet research	Wrong patient population
Dawes, D. et al. Preventing diabetes in primary care: a feasibility cluster randomized trial. (2014). Canadian journal of diabetes	Wrong patient population
Dunn S. L. Enhancing physical activity in cardiac patients who report hopelessness: Feasibility testing of an intervention. (2019). Health Education Journal	Wrong patient population
Fuller, A. S. et al. A preliminary evaluation of a novel lifestyle intervention in patients with hypertrophic cardiomyopathy. (2022). European journal of heart failure	Wrong patient population
Gill, D. P. et al. The HealtheSteps TM lifestyle prescription program to improve physical activity and modifiable risk factors for chronic disease: a pragmatic randomized controlled trial. (2019). BMC public health	Wrong patient population
Glynn, L. G. et al. Effectiveness of a smartphone application to promote physical activity in primary care: the SMART MOVE randomised controlled trial. (2014). The British journal of general practice : the journal of the Royal College of General Practitioners	Wrong patient population
Hemnes, A. R. et al. A Mobile Health Intervention to Increase Physical Activity in Pulmonary Arterial Hypertension. (2021). Chest	Wrong patient population
Herring, L. Y. et al. Physical activity after cardiac EventS (PACES): a group education programme with subsequent text message support designed to increase physical activity in individuals with diagnosed coronary heart disease: a randomised controlled trial. (2021). Open heart	Wrong patient population

Houchen-Wolloff, L. et al. Web-based cardiac RE habilitatio N alternative for those declining or dropping out of conventional rehabilitation: results of the WREN feasibility randomised controlled trial. (2018). Open heart	Wrong patient population
Joosen, P. et al. A smartphone-based solution to monitor daily physical activity in a care home. (2019). Journal of telemedicine and telecare	Wrong patient population
Kayser J. W. et al. A web-based tailored nursing intervention (TAVIE en m@rche) aimed at increasing walking after an acute coronary syndrome: Multicentre randomized trial. (2019). Journal of Advanced Nursing	Wrong patient population
Lisón J. F. et al. Impact of a Web-Based Exercise and Nutritional Education Intervention in Patients Who Are Obese With Hypertension: Randomized Wait-List Controlled Trial. (2020). J Med Internet Res	Wrong patient population
Lisón J. F. et al. Impact of a Web-Based Exercise and Nutritional Education Intervention in Patients Who Are Obese With Hypertension: Randomized Wait-List Controlled Trial. (2020). Journal of medical Internet research	Wrong patient population
Lo, Y-P. et al. Effects of Individualized Aerobic Exercise Training on Physical Activity and Health-Related Physical Fitness among Middle-Aged and Older Adults with Multimorbidity: A Randomized Controlled Trial. (2020). International journal of environmental research and public health	Wrong patient population
Lunde, P. et al. Long-term follow-up with a smartphone application improves exercise capacity post cardiac rehabilitation: a randomized controlled trial. (2020). European journal of preventive cardiology	Wrong patient population
Maddison, R. et al. A mobile phone intervention increases physical activity in people with cardiovascular disease: results from the HEART randomized controlled trial. (2015). European journal of preventive cardiology	Wrong patient population
Maddison, R. et al. The HEART Mobile Phone Trial: The Partial Mediating Effects of Self-Efficacy on Physical Activity among Cardiac Patients. (2014). Frontiers in public health	Wrong patient population
Manzoor, S. et al. Effectiveness of Mobile Health Augmented Cardiac Rehabilitation on Behavioural Outcomes among Post-acute Coronary Syndrome Patients: A Randomised Controlled Trial. (2021). Journal of the College of Physicians and Surgeons--Pakistan : JCPSP	Wrong patient population
Mensorio, M. S. et al. Analysis of the efficacy of an internet-based self-administered intervention ("Living Better") to promote healthy habits in a population with obesity and hypertension: An exploratory randomized controlled trial. (2019). International journal of medical informatics	Wrong patient population
Migneault J. P. et al. A Culturally Adapted Telecommunication System to Improve Physical Activity, Diet Quality, and Medication Adherence Among Hypertensive African–Americans: A Randomized Controlled Trial. (2012). Ann Behav Med	Wrong patient population
Muralidharan, S. et al. Engagement and Weight Loss: Results from the Mobile Health and Diabetes Trial. (2019). Diabetes technology & therapeutics	Wrong patient population
Owolabi, E. O. et al. Impact of mobile phone text messaging intervention on adherence among patients with diabetes in a rural setting: a randomized controlled trial. (2020). Medicine	Wrong patient population
Park, L. G. Mobile Health Intervention Promoting Physical Activity in Adults Post Cardiac Rehabilitation: Pilot Randomized Controlled Trial. (2021). JMIR formative research	Wrong patient population
Persell, S. D. et al. Effect of Home Blood Pressure Monitoring via a Smartphone Hypertension Coaching Application or Tracking Application on Adults With Uncontrolled Hypertension: A Randomized Clinical Trial. (2020). JAMA network open	Wrong patient population
Plotnikoff, R. C. et al. Integrating smartphone technology, social support and the outdoor physical environment to improve fitness among adults at risk of, or diagnosed with, Type 2 Diabetes: Findings from the 'eCoFit' randomized controlled trial. (2017). Preventive medicine	Wrong patient population

Saitoh, M. et al. Remote Cardiac Rehabilitation in Older Cardiac Disease: A Randomized Case Series Feasibility Study. (2022). Cardiology Research	Wrong patient population
Scherrenberg, M. et al. EU-CaRE study: could exercise-based cardiac telerehabilitation also be cost-effective in elderly?. (2021). International journal of cardiology	Wrong patient population
Schneider, L. H. et al. Efficacy of internet-delivered cognitive behavioural therapy following an acute coronary event: A randomized controlled trial. (2020). Internet interventions	Wrong patient population
Schroer S. et al. Effect of telerehabilitation on long-term adherence to yoga as an antihypertensive lifestyle intervention: Results of a randomized controlled trial. (2019). Complementary therapies in clinical practice	Wrong patient population
Taylor, A. et al. Randomised controlled trial of an augmented exercise referral scheme using web-based behavioural support for inactive adults with chronic health conditions: the e-coachER trial. (2021). British journal of sports medicine	Wrong patient population
Van de Winckel, A. et al. Combining a Hudl App With Telehealth to Increase Home Exercise Program Adherence in People With Chronic Diseases Experiencing Financial Distress: Randomized Controlled Trial. (2021). JMIR formative research	Wrong patient population
Vandelanotte, C. et al. Impact of a web-based personally tailored physical activity intervention on depression, anxiety, stress and quality of life: Secondary outcomes from a randomized controlled trial. (2022). Mental Health and Physical Activity	Wrong patient population
Volders, E. et al. The Effect of Active Plus, a Computer-Tailored Physical Activity Intervention, on the Physical Activity of Older Adults with Chronic Illness(es)-A Cluster Randomized Controlled Trial. (2020). International journal of environmental research and public health	Wrong patient population
Weinstock R. S. et al. Lessened decline in physical activity and impairment of older adults with diabetes with telemedicine and pedometer use: results from the IDEATel study. (2011). Age and ageing	Wrong patient population
Wilczynska, M. et al. Mediating Effects of the 'eCoFit' Physical Activity Intervention for Adults at Risk of, or Diagnosed with, Type 2 Diabetes. (2019). International journal of behavioral medicine	Wrong patient population
Ababneh, A. F. et al. Usability and effectiveness of a self care mhealth app in heart failure: A preliminary report. (2021). Cardiopulmonary Physical Therapy Journal	Wrong study design
Actrn, The Effectiveness of a Personalised Digital Health Intervention (Naluri) on Self-Care Behaviours and Quality of Life Outcomes among Cardiac Patients at the Malaysian National Heart Institute: a Randomized Controlled Trial. (2019). http://www.who.int/trialsearch/Trial2.aspx?TrialID=ACTRN12619000104156	Wrong study design
Actrn, While you're waiting - A waiting room-based, tablet-delivered program of educational videos designed to increase motivation to change lifestyle behaviours and improve clinic experience. (2018). http://www.who.int/trialsearch/Trial2.aspx?TrialID=ACTRN12618001725257	Wrong study design
Alghafri, T. S. et al. Acceptability of the "MOVEdiabetes" physical activity intervention in diabetes primary care settings in Oman: findings from participants and practitioners. (2020). BMC public health	Wrong study design
Alwakeel, A. J. et al. The Accessibility, Feasibility, and Safety of a Standardized Community-based Tele-Pulmonary Rehab Program for Chronic Obstructive Pulmonary Disease A 3-Year Real-World Prospective Study. (2022). Annals of the American Thoracic Society	Wrong study design
Batalik, L. et al. Cardiac rehabilitation based on the walking test and telerehabilitation improved cardiorespiratory fitness in people diagnosed with coronary heart disease during the covid-19 pandemic. (2021). International journal of environmental research and public health	Wrong study design
Beauchamp, T. et al. Accelerometer-Based Physical Activity Patterns and Associations With Outcomes Among Individuals With Osteoarthritis. (2022). Journal of clinical rheumatology : practical reports on rheumatic & musculoskeletal diseases	Wrong study design

Benzo, R. P. et al. Development and Feasibility of a Home Pulmonary Rehabilitation Program With Health Coaching. (2018). Respiratory care	Wrong study design
Boekhout, J. M. et al. An eHealth Intervention to Promote Physical Activity and Social Network of Single, Chronically Impaired Older Adults: Adaptation of an Existing Intervention Using Intervention Mapping. (2017). JMIR research protocols	Wrong study design
Burkow, T. M. et al. Promoting exercise training and physical activity in daily life: a feasibility study of a virtual group intervention for behaviour change in COPD. (2018). BMC medical informatics and decision making	Wrong study design
Carrillo, A. et al. An Adaptive Text Message Intervention to Promote Well-Being and Health Behavior Adherence for Patients With Cardiovascular Disease: Intervention Design and Preliminary Results. (2021). Journal of the Academy of Consultation-Liaison Psychiatry	Wrong study design
Celano, C. M. et al. An Adaptive, Algorithm-based Text Message Intervention to Promote Health Behavior Adherence in Type 2 Diabetes: treatment Development and Proof-of-Concept Trial. (2021). Journal of diabetes science and technology	Wrong study design
Deng, N. et al. A home-based pulmonary rehabilitation mHealth system to enhance the exercise capacity of patients with COPD: development and evaluation. (2021). BMC Medical Informatics & Decision Making	Wrong study design
Glowczynska, R. et al. Effects of hybrid comprehensive telerehabilitation on cardiopulmonary capacity in heart failure patients depending on diabetes mellitus: subanalysis of the TELEREH-HF randomized clinical trial. (2021). Cardiovascular diabetology	Wrong study design
Glowczynska, R. et al. Effects of hybrid comprehensive telerehabilitation on cardiopulmonary capacity in heart failure patients depending on diabetes mellitus: subanalysis of the TELEREH-HF randomized clinical trial. (2021). Cardiovascular diabetology	Wrong study design
Goode, A. D. et al. A telephone-delivered physical activity and dietary intervention for type 2 diabetes and hypertension: does intervention dose influence outcomes?. (2011). American Journal of Health Promotion	Wrong study design
Goode, A. D. et al. Relationship between intervention dose and outcomes in living well with diabetes--a randomized trial of a telephone-delivered lifestyle-based weight loss intervention. (2015). American Journal of Health Promotion	Wrong study design
Hwang, R. et al. Cost-Utility Analysis of Home-based Telerehabilitation Compared with Centre-based Rehabilitation in Patients with Heart Failure. (2018). Heart lung and circulation	Wrong study design
Lawford, B. et al. Association Between Therapeutic Alliance and Outcomes Following Telephone-Delivered Exercise by a Physical Therapist for People With Knee Osteoarthritis: Secondary Analyses From a Randomized Controlled Trial. (2021). JMIR rehabilitation and assistive technologies	Wrong study design
Lawler, S. P. et al,. Multiple health behavior changes and co-variation in a telephone counseling trial. (2010). Annals of behavioral medicine : a publication of the Society of Behavioral Medicine	Wrong study design
Lee, W. Y. H. et al. Responsiveness to Change of Self-Report and Device-Based Physical Activity Measures in the Living Well With Diabetes Trial. (2015). Journal of physical activity & health	Wrong study design
Legler, S. et al. Use of text messages to increase positive affect and promote physical activity in patients with heart disease: The Promoting Activity in Cardiac Patients via Text Messages (PACT) pilot study. (2020). Current psychology (New Brunswick, N.J.)	Wrong study design
Martinez-Garcia, M. D. M. et al. Effectiveness of Smartphone Devices in Promoting Physical Activity and Exercise in Patients with Chronic Obstructive Pulmonary Disease: A Systematic Review. (2017). COPD	Wrong study design

Moy, M. L. et al. Use of pedometer and Internet-mediated walking program in patients with chronic obstructive pulmonary disease. (2010). Journal of Rehabilitation Research & Development	Wrong study design
Nelligan, R. et al. Moderators of the Effect of a Self-directed Digitally Delivered Exercise Program for People With Knee Osteoarthritis: exploratory Analysis of a Randomized Controlled Trial. (2021). Journal of medical Internet research	Wrong study design
Nyberg A. et al. Can the COPD web be used to promote self-management in patients with COPD in swedish primary care: a controlled pragmatic pilot trial with 3 month- and 12 month follow-up. (2019). Scandinavian journal of primary health care	Wrong study design
Pelle, T. et al. Use and usability of the dr. Bart app and its relation with health care utilisation and clinical outcomes in people with knee and/or hip osteoarthritis. (2021). BMC health services research	Wrong study design
Piette, J. et al. Clinical complexity and the effectiveness of an intervention for depressed diabetes patients. (2011). Chronic illness	Wrong study design
Pignato, M. et al. Level of participation in physical therapy or an internet-based exercise training program: associations with outcomes for patients with knee osteoarthritis. (2018). BMC musculoskeletal disorders	Wrong study design
Robinson, S. A. et al. A web-based physical activity intervention benefits persons with low self-efficacy in COPD: results from a randomized controlled trial. (2019). Journal of behavioral medicine	Wrong study design
Simmich J. et al. A Co-Designed Active Video Game for Physical Activity Promotion in People With Chronic Obstructive Pulmonary Disease: Pilot Trial. (2021). JMIR serious games	Wrong study design
Simmich, J. et al. A Co-Designed Active Video Game for Physical Activity Promotion in People With Chronic Obstructive Pulmonary Disease: Pilot Trial. (2021). JMIR serious games	Wrong study design
Sittig, S. et al. Incorporating Behavioral Trigger Messages Into a Mobile Health App for Chronic Disease Management: Randomized Clinical Feasibility Trial in Diabetes. (2020). JMIR mHealth and uHealth	Wrong study design
Stickland, M. et al. Using Telehealth technology to deliver pulmonary rehabilitation to patients with chronic obstructive pulmonary disease. (2011). Canadian respiratory journal [revue canadienne de pneumologie]	Wrong study design
Torri, A. et al. Promotion of Home-Based Exercise Training as Secondary Prevention of Coronary Heart Disease: A PILOT WEB-BASED INTERVENTION. (2018). Journal of cardiopulmonary rehabilitation and prevention	Wrong study design
Verwey, R. et al. Process evaluation of physical activity counselling with and without the use of mobile technology: A mixed methods study. (2016). International journal of nursing studies	Wrong study design
Vorrink, S. Nw. et al. A Mobile Phone App to Stimulate Daily Physical Activity in Patients with Chronic Obstructive Pulmonary Disease: Development, Feasibility, and Pilot Studies. (2016). JMIR mHealth and uHealth	Wrong study design
Waki, K. et al. DialBetics: Smartphone-Based Self-Management for Type 2 Diabetes Patients on Insulin Injections. (2016). Journal of diabetes science and technology	Wrong study design
Waki, K. et al. Dialbetics: smartphone-based selfmanagement for type 2 diabetes patients. (2013). Diabetes technology & therapeutics	Wrong study design
Waters, L. A. et al. The impact of behavioural screening on intervention outcomes in a randomised, controlled multiple behaviour intervention trial. (2011). The international journal of behavioral nutrition and physical activity	Wrong study design
Wong, E. M. et al. Randomized controlled trial on amount of physical exercise of a home-based e-health educational intervention for middleaged adults with coronary heart disease. (2016). Journal of the hong kong college of cardiology	Wrong study design

Alasfour, M. et al. The effect of innovative smartphone application on adherence to a home-based exercise programs for female older adults with knee osteoarthritis in Saudi Arabia: a randomized controlled trial. (2022). Disability & Rehabilitation	Wrong outcomes
Al-Ghafri, T. S. et al. Changes in Self-Efficacy and Social Support after an Intervention to Increase Physical Activity Among Adults with Type 2 Diabetes in Oman: A 12-month follow-up of the MOVEdiabetes trial. (2021). Sultan Qaboos University medical journal	Wrong outcomes
Azma, K. et al. Efficacy of tele-rehabilitation compared with office-based physical therapy in patients with knee osteoarthritis: A randomized clinical trial. (2018). Journal of telemedicine and telecare	Wrong outcomes
Bi, J. et al. WeChat as a Platform for Baduanjin Intervention in Patients With Stable Chronic Obstructive Pulmonary Disease in China: Retrospective Randomized Controlled Trial. (2021). JMIR mHealth and uHealth	Wrong outcomes
Castelnuovo, G. et al. TECNOB study: ad interim results of a randomized controlled trial of a multidisciplinary telecare intervention for obese patients with type-2 diabetes. (2011). Clinical practice and epidemiology in mental health	Wrong outcomes
Crooks, M. G. et al. Evidence generation for the clinical impact of mycopd in patients with mild, moderate and newly diagnosed copd: a randomised controlled trial. (2020). ERJ open research	Wrong outcomes
Drks, The influence of mobile phone-based health reminders on patient adherence to drugs and healthy lifestyle recommendation for effective management of type 2 diabetes: a randomised control trial in Dhaka, Bangladesh. (2019). http://www.who.int/trialsearch/Trial2.aspx?TrialID=DRKS00017647	Wrong outcomes
Glasgow RE. et al. Outcomes of minimal and moderate support versions of an internet-based diabetes self-management support program. . (2010). Journal of general internal medicine	Wrong outcomes
Graven, L. J. et al. The coping in heart failure (COPE-HF) partnership intervention for heart failure symptoms: Implications for palliative care. (2022). Progress in Palliative Care	Wrong outcomes
Hallgren, M. et al. Physical exercise and internet-based cognitive-behavioural therapy in the treatment of depression: randomised controlled trial. (2015). The British journal of psychiatry : the journal of mental science	Wrong outcomes
Hidrus, A. et al. Effects of technology-supported brain breaks videos on exercise self-efficacy among type 2 diabetes mellitus Malaysians. (2022). Scientific reports	Wrong outcomes
Holmen H. et al. Stages of change for physical activity and dietary habits in persons with type 2 diabetes included in a mobile health intervention: the Norwegian study in RENEWING HEALTH. (2016). BMJ Open Diabetes Res Care	Wrong outcomes
Jahan, Y. et al. Awareness Development and Usage of Mobile Health Technology Among Individuals With Hypertension in a Rural Community of Bangladesh: randomized Controlled Trial. (2020). Journal of medical Internet research	Wrong outcomes
Joshi, R.et al. Improving adherence and outcomes in diabetic patients. (2017). Patient preference and adherence	Wrong outcomes
Kang, S. H. et al. Management of cardiovascular disease using an mHealth tool: a randomized clinical trial. (2021). npj Digital Medicine	Wrong outcomes
Kim, HS. et al. Adherence to diabetes control recommendations: impact of nurse telephone calls. (2003). Journal of Advanced Nursing (Wiley-Blackwell)	Wrong outcomes
Lee, S. E. et al. Effects of Short-term Mobile Application Use on Weight Reduction for Patients with Type 2 Diabetes. (2021). Journal of Obesity and Metabolic Syndrome	Wrong outcomes
Lindberg I. et al. Telemonitoring and Health Counseling for Self-Management Support of Patients With Type 2 Diabetes: A Randomized Controlled Trial. (2017). JMIR Diabetes	Wrong outcomes
Miller, S. et al. Feasibility of resp-fit: technology-enhanced self-management intervention for adults with copd. (2021). International journal of COPD	Wrong outcomes

Oh, S. W. et al. Effect of an Integrative Mobile Health Intervention in Patients With Hypertension and Diabetes: Crossover Study. (2022). JMIR mHealth and uHealth	Wrong outcomes
Orme, M. W. et al. Findings of the Chronic Obstructive Pulmonary Disease-Sitting and Exacerbations Trial (COPD-SEAT) in Reducing Sedentary Time Using Wearable and Mobile Technologies With Educational Support: Randomized Controlled Feasibility Trial. (2018). JMIR mHealth and uHealth	Wrong outcomes
Pelle, T. et al. Use and usability of the dr. Bart app and its relation with health care utilisation and clinical outcomes in people with knee and/or hip osteoarthritis. (2021). BMC health services research	Wrong outcomes
Sacco, W. P. et al. Effect of a brief, regular telephone intervention by paraprofessionals for type 2 diabetes. (2009). Journal of behavioral medicine	Wrong outcomes
Sadanshiv, M. et al. Feasibility of computer-generated telephonic message-based follow-up system among healthcare workers with diabetes: a randomized controlled trial. (2020). BMJ open diabetes research & care	Wrong outcomes
Thakkar J. et al. Patterns, predictors and effects of texting intervention on physical activity in CHD - insights from the TEXT ME randomized clinical trial. (2016). European journal of preventive cardiology	Wrong outcomes
Torbjørnsen A. et al. A Low-Intensity Mobile Health Intervention With and Without Health Counseling for Persons With Type 2 Diabetes, Part 1: Baseline and Short-Term Results From a Randomized Controlled Trial in the Norwegian Part of RENEWING HEALTH. (2014). JMIR mHealth and uHealth	Wrong outcomes
Torbjørnsen A. et al. Acceptability of an mHealth App Intervention for Persons With Type 2 Diabetes and its Associations With Initial Self-Management: Randomized Controlled Trial . (2018). JMIR Mhealth Uhealth.	Wrong outcomes
Turkstra, E. et al. Cost-effectiveness of a coronary heart disease secondary prevention program in patients with myocardial infarction: results from a randomised controlled trial (ProActive Heart). (2013). BMC cardiovascular disorders	Wrong outcomes
Van Dyck, D. et al. The relationship between changes in steps/day and health outcomes after a pedometer-based physical activity intervention with telephone support in type 2 diabetes patients. (2013). Health education research	Wrong outcomes
Wang, J. et al. A Behavioral Lifestyle Intervention Enhanced With Multiple-Behavior Self-Monitoring Using Mobile and Connected Tools for Underserved Individuals With Type 2 Diabetes and Comorbid Overweight or Obesity: Pilot Comparative Effectiveness Trial. (2018). JMIR mHealth and uHealth	Wrong outcomes
Wang, X. et al. The role of text messaging intervention in Inner Mongolia among patients with type 2 diabetes mellitus: a randomized controlled trial. (2020). BMC medical informatics and decision making	Wrong outcomes
Yasmin, F. et al. The influence of mobile phone-based health reminders on patient adherence to medications and healthy lifestyle recommendations for effective management of diabetes type 2: a randomized control trial in Dhaka, Bangladesh. (2020). BMC health services research	Wrong outcomes
Glozier N. et al. Internet-delivered cognitive behavioural therapy for adults with mild to moderate depression and high cardiovascular disease risks: a randomised attention-controlled trial. (2013). PloS one	Not targeting physical activity
Glozier N. et al. Internet-delivered cognitive behavioural therapy for adults with mild to moderate depression and high cardiovascular disease risks: a randomised attention-controlled trial. (2013). PLoS One	Not targeting physical activity
Kirkman, M. S.et al. A telephone-delivered intervention for patients with NIDDM. Effect on coronary risk factors. (1994). Diabetes care	Not targeting physical activity
Quynh p. et al. Feasibility and Efficacy of an mHealth Game for Managing Anxiety: "Flowy" Randomized Controlled Pilot Trial and Design Evaluation. (2016). Games for health journal	Not targeting physical activity

Shahid, M. et al. Mobile phone intervention to improve diabetes care in rural areas of Pakistan: a randomized controlled trial. (2015). Journal of the College of Physicians and Surgeons--Pakistan : JCPSP	Not targeting physical activity
Shea S. et al. A randomized trial comparing telemedicine case management with usual care in older, ethnically diverse, medically underserved patients with diabetes mellitus: 5 year results of the IDEATel study. (2009). J Am Med Inform Assoc	Not targeting physical activity
Sun, C. et al. Mobile Phone-Based Telemedicine Practice in Older Chinese Patients with Type 2 Diabetes Mellitus: randomized Controlled Trial. (2019). JMIR mHealth and uHealth	Not targeting physical activity
Walters J. et al. Effects of telephone health mentoring in community-recruited chronic obstructive pulmonary disease on self-management capacity, quality of life and psychological morbidity: a randomised controlled trial. . (2013). BMJ Open	Not targeting physical activity
Bi, J. et al. WeChat as a Platform for Baduanjin Intervention in Patients With Stable Chronic Obstructive Pulmonary Disease in China: retrospective Randomized Controlled Trial. (2021). JMIR mHealth and uHealth	Wrong intervention
Bozorgi, A. et al. The effect of the mobile "blood pressure management application" on hypertension self-management enhancement: a randomized controlled trial. (2021). Trials	Wrong intervention
Bravo-Escobar, R. et al. Effectiveness of e-Health cardiac rehabilitation program on quality of life associated with symptoms of anxiety and depression in moderate-risk patients. (2021). Scientific reports	Wrong intervention
Bravo-Escobar, R. et al. Effectiveness of e-Health cardiac rehabilitation program on quality of life associated with symptoms of anxiety and depression in moderate-risk patients. (2021). Scientific reports	Wrong intervention
Hunter, D. J. et al. Effectiveness of a new service delivery model for management of knee osteoarthritis in primary care: a cluster randomised controlled trial. (2022). Arthritis care & research	Wrong intervention
Jiang, Y. et al. Evaluating an intervention program using wechat for patients with chronic obstructive pulmonary disease: randomized controlled trial. (2020). Journal of medical Internet research	Wrong intervention
Johansson, P. et al. Trajectories and associations between depression and physical activity in patients with cardiovascular disease during participation in an internet-based cognitive behavioural therapy programme. (2021). European journal of cardiovascular nursing	Wrong intervention
Kaldo, V. et al. Effects of internet-based cognitive behavioural therapy and physical exercise on sick leave and employment in primary care patients with depression: two subgroup analyses. (2018). Occupational and environmental medicine	Wrong intervention
King D. K. et al. Outcomes of a multifaceted physical activity regimen as part of a diabetes self-management intervention. (2009). Annals of behavioral medicine	Wrong intervention
Ku, E. J. et al. Clinical efficacy and plausibility of a smartphone-based integrated online real-time diabetes care system via glucose and diet data management: a pilot study. (2020). Internal medicine journal	Wrong intervention
Liu, W. T. et al. Efficacy of a cell phone-based exercise programme for COPD. (2008). The European respiratory journal	Wrong intervention
Lukkahatai, N. et al. Feasibility of Using Mobile Technology to Improve Physical Activity Among People Living with Diabetes in Asia. (2021). Asian/Pacific Island nursing journal	Wrong intervention
Mayberry, L. S. et al. Mixed-Methods Randomized Evaluation of FAMS: a Mobile Phone-Delivered Intervention to Improve Family/Friend Involvement in Adults' Type 2 Diabetes Self-Care. (2020). Annals of Behavioral Medicine	Wrong intervention
Nystrom, M. B. T. et al. Behavioral activation versus physical activity via the internet: A randomized controlled trial. (2017). Journal of affective disorders	Wrong intervention

Rollman, B. L. et al. Efficacy of Blended Collaborative Care for Patients With Heart Failure and Comorbid Depression: a Randomized Clinical Trial. (2021). JAMA internal medicine	Wrong intervention
Allen K. D. et al. Physical therapy vs internet-based exercise training for patients with knee osteoarthritis: results of a randomized controlled trial. (2018). Osteoarthritis and Cartilage	Duplicate
Allen, K. D. et al. Stepped exercise program for patients with knee osteoarthritis a randomized controlled trial. (2021). Annals of Internal Medicine	Duplicate
Anderson, D. et al. Managing the space between visits: Telephonic disease management for underserved patients with diabetes. (2010). J Gen Intern Med.	Duplicate
Baker, K. et al. Efficacy of Computer-Based Telephone Counseling on Long-Term Adherence to Strength Training in Elderly Patients With Knee Osteoarthritis: a Randomized Trial. (2020). Arthritis care & research	Duplicate
Benzo, R. P. et al. Feasibility of a Health Coaching and Home-Based Rehabilitation Intervention With Remote Monitoring for COPD. (2021). Respiratory care	Duplicate
Chaplin, E. et al. Interactive web-based pulmonary rehabilitation programme: a randomised controlled feasibility trial. (2017). BMJ open	Duplicate
Coombes J. et al. Personal Activity Intelligence e-Health Program in People with Type 2 Diabetes: a Pilot Randomized Controlled Trial.. (2022). Medicine and science in sports and exercise	Duplicate
Deka, P. et al. MOVE-HF: an internet-based pilot study to improve adherence to exercise in patients with heart failure. (2019). European journal of cardiovascular nursing : journal of the Working Group on Cardiovascular Nursing of the European Society of Cardiology	Duplicate
Demeyer H. et al. Physical activity is increased by a 12-week semiautomated telecoaching programme in patients with COPD: a multicentre randomised controlled trial. (2017). Thorax	Duplicate
Demeyer H. et al. Physical activity is increased by a 12-week semiautomated telecoaching programme in patients with COPD: a multicentre randomised controlled trial. (2017). Thorax	Duplicate
Galdiz, J. B. et al. Telerehabilitation Programme as a Maintenance Strategy for COPD Patients: A 12-Month Randomized Clinical Trial. (2020). Archivos de bronconeumologia	Duplicate
Galdiz, J. B. et al. Telerehabilitation Programme as a Maintenance Strategy for COPD Patients: A 12-Month Randomized Clinical Trial. (2021). Archivos de bronconeumologia	Duplicate
Gohir, S. A. et al. Effectiveness of Internet-Based Exercises Aimed at Treating Knee Osteoarthritis: The iBEAT-OA Randomized Clinical Trial. (2021). JAMA network open	Duplicate
Hidrus, A. et al. Effects of Brain Breaks Video Intervention of Decisional Balance among Malaysians with Type 2 Diabetes Mellitus: A Randomised Controlled Trial. (2021). International journal of environmental research and public health	Duplicate
Hinman, R. S. et al. Does telephone-delivered exercise advice and support by physiotherapists improve pain and/or function in people with knee osteoarthritis? Telecare randomised controlled trial. (2020). British journal of sports medicine	Duplicate
Hochsmann, C. et al. Effectiveness of a Behavior Change Technique-Based Smartphone Game to Improve Intrinsic Motivation and Physical Activity Adherence in Patients With Type 2 Diabetes: Randomized Controlled Trial. (2019). JMIR serious games	Duplicate
Hsu, Y- I. et al. Effects of Diet Control and Telemedicine-Based Resistance Exercise Intervention on Patients with Obesity and Knee Osteoarthritis: A Randomized Control Trial. (2021). International journal of environmental research and public health	Duplicate
Huo X. et al. Effects of Mobile Text Messaging on Glycemic Control in Patients With Coronary Heart Disease and Diabetes Mellitus: A Randomized Clinical Trial. (2019). Circulation. Cardiovascular quality and outcomes	Duplicate

Jaarsma T. et al. Effects of exergaming on exercise capacity in patients with heart failure: results of an international multicentre randomized controlled trial. (2021). Eur J Heart Fail	Duplicate
Jaarsma, T. et al. Effects of exergaming on exercise capacity in patients with heart failure: results of an international multicentre randomized controlled trial. (2021). European journal of heart failure	Duplicate
Li, J. et al. Efficiency of an mHealth App and Chest-Wearable Remote Exercise Monitoring Intervention in Patients With Type 2 Diabetes: A Prospective, Multicenter Randomized Controlled Trial. (2021). JMIR mHealth and uHealth	Duplicate
Lim, S. L. et al. Effect of a Smartphone App on Weight Change and Metabolic Outcomes in Asian Adults with Type 2 Diabetes: A Randomized Clinical Trial. (2021). JAMA network open	Duplicate
Nelligan, R. et al. Effects of a Self-directed Web-Based Strengthening Exercise and Physical Activity Program Supported by Automated Text Messages for People with Knee Osteoarthritis: A Randomized Clinical Trial. (2021). JAMA internal medicine	Duplicate
Nelligan, R. et al. Effects of self-directed web-based strengthening exercise and physical activity supported by automated sms on pain and function in people with knee osteoarthritis: A randomised controlled trial. (2021). Internal Medicine Journal	Duplicate
Piotrowicz, E. et al. Effects of a 9-Week Hybrid Comprehensive Telerehabilitation Program on Long-term Outcomes in Patients With Heart Failure: the Telerehabilitation in Heart Failure Patients (TELEREH-HF) Randomized Clinical Trial. (2020). JAMA cardiology	Duplicate
Piotrowicz, E. et al. Relationship between physical capacity and depression in heart failure patients undergoing hybrid comprehensive telerehabilitation vs. usual care: subanalysis from the TELEREH-HF Randomized Clinical Trial. (2022). European journal of cardiovascular nursing	Duplicate
Robinson, S. A. et al. A randomised trial of a web-based physical activity self-management intervention in COPD. (2021). ERJ open research	Duplicate
Snoek, J. A. et al. The sustained effects of extending cardiac rehabilitation with a six-month telemonitoring and telecoaching programme on fitness, quality of life, cardiovascular risk factors and care utilisation in CAD patients: The TeleCaRe study. (2021). Journal of telemedicine and telecare	Duplicate
Strom, M. et al. Internet-delivered therapist-guided physical activity for mild to moderate depression: a randomized controlled trial. (2013). PeerJ	Duplicate
Tabak, M. et al. A telecare programme for self-management of COPD exacerbations and promotion of an active lifestyle. (2013). European respiratory journal	Duplicate
Thiengwittayaporn, S. et al. Development of a mobile application to improve exercise accuracy and quality of life in knee osteoarthritis patients: a randomized controlled trial. (2021). Archives of orthopaedic and trauma surgery	Duplicate
Thorsen, I. K. et al. The Effects of a Lifestyle Intervention Supported by the InterWalk Smartphone App on Increasing Physical Activity Among Persons With Type 2 Diabetes: Parallel-Group, Randomized Trial. (2022). JMIR mHealth and uHealth	Duplicate
Tomita, M. R. et al. Effects of multidisciplinary Internet-based program on management of heart failure. (2008). Journal of multidisciplinary healthcare	Duplicate
Turan Kavradim, S. et al. The effect of education and telephone follow-up intervention based on the Roy Adaptation Model after myocardial infarction: randomised controlled trial. (2020). Scandinavian Journal of Caring Sciences	Duplicate
Waller, K. et al. Effectiveness and acceptability of a text message intervention (DTEXT) on HbA1c and self-management for people with type 2 diabetes. A randomized controlled trial. (2020). Patient education and counseling	Duplicate
Waller, K. et al. Effectiveness and acceptability of a text message intervention (DTEXT) on HbA1c and self-management for people with type 2 diabetes. A randomized controlled trial. (2021). Patient education and counseling	Duplicate

Wang, L. et al. A mobile health application to support self-management in patients with chronic obstructive pulmonary disease: a randomised controlled trial. (2021). Clinical rehabilitation	Duplicate
Etemadifar, S. et al. The effects of implementation cardiac rehabilitation program using a mobile application on activity tolerance, fatigue, and dyspnea in patients with myocardial infraction; a randomized clinical trial study. (2021). Journal of Isfahan Medical School	Wrong language
Lange, I. et al. Effect of a tele-care model on self-management and metabolic control among patients with type 2 diabetes in primary care centers in Santiago, Chile. (2010). Revista medica de Chile	Wrong language
Munoz-Fonseca, F. et al. [Effects of a telehealth-based physical exercise program on functional capacity in patients with osteoarthritis]. (2022). Efectos en la capacidad funcional de un programa de ejercicio fisico terapeutico basado en telesalud en personas con diagnostico de osteoartritis de rodilla y cadera.	Wrong language
Huo X. et al. Effects of Mobile Text Messaging on Glycemic Control in Patients With Coronary Heart Disease and Diabetes Mellitus.. (2019). Circulation Cardiovascular quality and outcomes	Insufficient data - no reply from authors
Zheng X, et al. Effect of Text Messaging on Risk Factor Management in Patients With Coronary Heart Disease: the CHAT Randomized Clinical Trial. (2019). Circulation Cardiovascular quality and outcomes	Insufficient data - no reply from authors
Barnason S. et al. Influence of an early recovery telehealth intervention on physical activity and functioning after coronary artery bypass surgery among older adults with high disease burden. . (2009). Heart & Lung	Insufficient data - no reply from authors
Chen, T-Y. et al. A web-based self-care program to promote healthy lifestyles and control blood pressure in patients with primary hypertension: A randomized controlled trial. (2022). Journal of nursing scholarship : an official publication of sigma theta tau international honor society of nursing	Insufficient data - no reply from authors
Franke K. J. et al. Telemonitoring of home exercise cycle training in patients with COPD. (2016). International journal of chronic obstructive pulmonary disease	Insufficient data - no reply from authors
Hansel B. et al. Fully Automated Web-Based Program Improves Lifestyle Habits and HbA1c in Patients With Type 2 Diabetes and Abdominal Obesity: Randomized Trial of Patient E-Coaching Nutritional Support (The ANODE Study).. (2017). Journal of medical Internet research	Insufficient data - no reply from authors
Mayberry, L. S. et al. Mixed-Methods Randomized Evaluation of FAMS: A Mobile Phone-Delivered Intervention to Improve Family/Friend Involvement in Adults' Type 2 Diabetes Self-Care. (2021). Annals of behavioral medicine : a publication of the Society of Behavioral Medicine	Insufficient data - no reply from authors
Tabak M. et al. A telehealth program for self-management of COPD exacerbations and promotion of an active lifestyle: a pilot randomized controlled trial. (2014). International journal of chronic obstructive pulmonary disease	Insufficient data - no reply from authors
von Storch, K. et al. Telemedicine-Assisted Self-Management Program for Type 2 Diabetes Patients. (2019). Diabetes Technology & Therapeutics	Insufficient data - no reply from authors
Vorrink S. N. et al. Efficacy of an mHealth intervention to stimulate physical activity in COPD patients after pulmonary rehabilitation. (2016). The European respiratory journal	Insufficient data - no reply from authors

Supplementary Table 3. Study characteristics of the included studies

**Study:** Author (publication year), [reference number in main paper], country	**Participants:** Number of participants randomized, (intervention group/usual care group), mean age, proportion female, mean body-mass index, low socioeconomic status (explanation)	**Comparator:** Usual care and waiting list/minimal intervention/rehabilitation (type of minimal intervention/rehabilitation)	**Intervention:** Digital only or combined digital and in-person intervention, intervention duration in weeks, (follow-up duration in weeks), physical activity category	**Digital health intervention:** Type of digital solution(s) [name of solution], (primary digital health category)	**Outcomes:**	**Adverse events:**
Agboola et al. (2016), [53], USA	126 (64/62), mean age 51, 52% female, BMI NR, not low SES (12% Unemployed)	UC + MI (Device (blinded))	Digital only, 26 wks, Physical activity	Text messages (mHealth)	Obj PA (Pedometer [steps/mo])	NON-SAE NR, S-AE (Hospitalization)
Akinci et al. (2018), [97], Turkey	44 (22/22), mean age 52, 67% female, 32, SES NR	UC + MI (Educational brochure)	Digital only, 8 wks, Exercise therapy	Website + telephone calls or text messages (eHealth)	Obj PA (Pedometer [steps/d]), Obj PF (6MWT), HRQOL (EQ-5D)	NON-SAE (No events occurred), S-AE (No events occurred)
Alghafri et al. (2018), [145], Oman	232 (122/110), mean age 42, 59% female, 33, Low SES (54% Income <1000 Omani rials per month)	UC	Combined digital and in-person, 52 wks, Physical activity	Wearable [Pedometer, Yamax Digi-Walker SW-200, Yamasa Tokei Keiki, Tokyo, Japan] + App [WhatsApp]+ Telephone (mHealth + Device)	Obj PA (Accelerometer [steps/d])	NON-SAE (Joint pain + accelerometer discomfort), S-AE NR
Allen et al. (2018), [54], USA	210 (142/68), mean age 65, 72% female, 31, not low SES (20% low income)	UC (WL)	Digital only, 17 wks, (52 wks), Exercise therapy	Website + videos (eHealth)	Obj PF (TUG), Subj PA (PASE [total])	NON-SAE (Knee/shoulder/ankle pain), S-AE NR
Allen et al. (2021), [55], USA	345 (230/115), mean age 60, 15% female, 34, not low SES (23% Percived low income)	UC + MI (Educational materials via mail every 2 wks for 9 mo)	Combined digital and in-person, 13 wks, Exercise therapy	Website + tablet + telephone calls (eHealth)	Obj PF (6MWT), Subj PA (PASE)	NON-SAE (Hip pain), S-AE NR
Alonso-Dominguez et al. (2019), [146], Spain	204 (102/102), mean age 61, 46% female, BMI NR, not low SES (49% Elementary school)	UC	Combined digital and in-person, 13 wks, (52 wks), Exercise therapy	App [EVIDENT II] (mHealth)	Obj PA (Pedometer [steps/d])	NON-SAE NR, S-AE NR

Anderson et al. (2010), [56], USA	295 (146/149), mean age 0, 58% female, 35, not low SES (39% Low education)	UC	Digital only, 52 wks,	Telephone calls + e-mails (mHealth)	Subj PA (RAPA)	NON-SAE (Mental illness), S-AE (Terminal illness + death(s))
Andrade et al. (2021), [147], Brazil	28 (14/14), mean age 60, 39% female, 28, SES NR	UC + Rehab (Cardiac rehabilitation)	Combined digital and in-person, 12 wks, Exercise therapy	Telephone calls (mHealth)	Obj PA (Accelerometer [steps/d]), Obj PF (6MWT), DEP (SF-36 [Mental health]), HRQOL (MLHFQ)	NON-SAE NR, S-AE (Heart failiure + diabetes decompensation + pacemaker implantation)
Avila et al. (2018 + 2019), [98, 189], Belgium	60 (30/30), mean age 62, 11% female, BMI NR, SES NR	UC + MI (One contact at 3 mo)	Digital only, 13 wks, (52 wks), Exercise therapy	Weareble [Garmin fortrunner 210] + web-app + telephone calls or e-mails (eHealth + Device)	Obj PA (Accelerometer [steps/d]), Subj PF (SF-36 [PF]), HRQOL (SF-36 [PCS])	NON-SAE NR, S-AE (Stroke + prostate cancer)
Bailey et al. (2020), [99], UK	20 (10/10), mean age 56, 50% female, 31, SES NR	UC	Digital only, 8 wks, Physical activity	App + text messages (mHealth)	Obj PA (Accelerometer [steps])	NON-SAE NR, S-AE NR
Baker et al. (2019), [57], USA	104 (52/52), mean age 65, 81% female, 31, SES NR	UC + MI (Monthly reminder telephone calls)	Combined digital and in-person, 104 wks, Exercise therapy + physical activity	Telephone calls (mHealth)	Obj PF (TUG), Subj PA (Adherence to strength training),	NON-SAE NR, S-AE NR
Bartholdy et al. (2019), [148], Denmark	38 (19/19), mean age 65, 76% female, 28, SES NR	UC	Combined digital and in-person, 6 wks, Exercise therapy + physical activity	Text messages (mHealth)	Obj PA (Accelerometer [min spent moving]), Subj PF (KOOS [PF]), HRQOL (KOOS [QOL])	NON-SAE NR, S-AE NR
Bender et al. (2017), [58], USA	45 (22/23), mean age 58, 62% female, 30, not low SES (20% Low income)	UC (WL) + MI (Device + education on hepatitis)	Combined digital and in-person, 13 wks, (13 wks), Physical activity	Wearable [Fitbit Zip] + app [Fitbit] + Facebook group [private] (mHealth + Device)	Obj PA (Accelerometer [step counts])	NON-SAE NR, S-AE NR
Bennell et al. (2017), [76], Australia	148 (74/74), mean age 61, 56% female, 31, not low SES (6% Education level)	UC + MI (Online educational material)	Digital only, 13 wks, (39 wks), Exercise therapy	Video conferences [Skype] + website + e-mails (eHealth)	Subj PF (WOMAC [PF]), HRQOL (AQoL-2)	NON-SAE (Knee pain + muscle cramping/soreness + pain in other area + swelling), S-AE NR
Bennell et al. (2018), [77], Australia	144 (73/71), mean age 61, 57% female, 29, not low SES (3% Unemployed)	UC + MI (Exercise program + 5 PT sessions (in-person))	Combined digital and in-person, 8 wks, (52 wks), Exercise therapy	Website + e-mails (eHealth)	Subj PA (PASE), Subj PF (WOMAC [PF]), DEP (DASS DEP), ANX (DASS [ANX]), HRQOL (AQoL-2)	NON-SAE (Pain including hip + muscle soreness + numbness), S-AE NR

Bennell et al. (2020), [78], Australia	110 (56/54), mean age 62, 77% female, 37, SES NR	UC	Combined digital and in-person, 24 wks, Exercise therapy	Text messages (mHealth)	Subj PA (PASE), Subj PF (KOOS [PF]), HRQOL (AQoL)	NON-SAE (Knee/other areas pain), S-AE ()
Benson et al. (2019), [59], USA	118 (60/58), mean age 60, 45% female, 37, not low SES (0% Education)	UC + MI (Consultation with study RDN (5 min) + results of diabetes measures)	Digital only, 52 wks, Physical activity	Telephone calls (mHealth)	Subj PA (PA [150 min of MPA/wk])	NON-SAE (No events occurred), S-AE (No events occurred)
Bentley et al. (2020), [149], UK	30 (19/11), mean age 68, 57% female, BMI NR, SES NR	UC + MI + Rehab (Device (blinded)) (Pulmonary rehabilitation)	Combined digital and in-person, 14 wks, Physical activity	App [MyHealthAvatar -Diabetes] + wearable [Fitbit, Charge HR, Charge 2 or One] (mHealth + Device)	Obj PA (Fitbit [steps]), Obj PF (ISWT), DEP (PHQ-9), HRQOL (EQ-5D)	NON-SAE NR, S-AE NR
Benzo et al. (2021), [60], USA	146 (72/74), mean age 69, 51% female, BMI NR, SES NR	UC (WL)	Digital only, 8 wks, Exercise therapy	Wearable [Vívofit activity monitor, Garmin, Schaffhausen, Switzerland + pulse oximeter, 3150 WristOx2, Nonin Medical, Plymouth, Minnesota] + tablet [Android, Google, Menlo Park, California] + website + telephone calls (mHealth + Device)	Obj PA (Accelerometer [steps]), HRQOL (CRQ [Physical summary])	NON-SAE (No information), S-AE NR
Benzo et al. (2022), [61], USA	375 (188/187), mean age 69, 57% female, 29, not low SES (33% <High school)	UC (WL) + MI (Educational packet of 12 self-management themes)	Digital only, 12 wks, (24 wks), Exercise therapy	Wearable [activity monitor, Garmin Vivofit + oximeter, Nonin 3150] + Tablet + exercise videos + health coach telephone calls and messages (mHealth + Device)	Obj PA (Accelerometer [steps/d]), Subj PF (SMAS), DEP (PHQ-9), ANX (GAD-2), HRQOL (CRQ [Physical])	NON-SAE (), S-AE (Disease progression + death(s))
Bernocchi et al. (2018), [150], Italy	112 (56/56), mean age 71, 18% female, 28, SES NR	UC + MI (Education session)	Combined digital and in-person, 17 wks, (26 wks), Exercise therapy + physical activity	Telephone calls + device [portable one-lead electrocardiograph, Card Guard Scientific Survival Ltd., Rehovot, Israel] + wearable [pulse oximeter, GIMA,	Obj PF (6MWT), Subj PA (PASE), HRQOL (MLHFQ)	NON-SAE NR, S-AE (Hospitalization + death(s))

				Milan, Italy + pedometer] (mHealth)		
Bossen et al. (2013), [100], The Netherlands	199 (100/99), mean age 62, 64% female, 28, not low SES (14% Low education)	UC (WL) + MI (Education)	Digital only, 13 wks, (52 wks), Physical activity	website + text messages [automatic] + e-mails (eHealth)	Obj PA (Accelerometer [total PA min/d]), Subj PF (KOOS/HOOS [PF]), DEP (HADS-D), ANX (HADS-A), HRQOL (KOOS/HOOS [QOL])	NON-SAE (Extreme pain + injuries), S-AE (No events occurred)
Bozorgi et al. (2021), [101], Iran	120 (60/60), mean age 52, 41% female, 29, not low SES (47% <Education level, diploma)	UC	Digital only, 8 wks,	App (mHealth)	Subj PA (GPAQ)	NON-SAE NR, S-AE NR
Cameron-Tucker et al. (2016), [79], Australia	65 (35/30), mean age 69, 55% female, BMI NR, SES NR	UC	Digital only, 8 wks, Physical activity	Telephone calls (mHealth)	Obj PF (6MWT), Subj PA (SNAPPS [min/d walking]), HRQOL (CAT)	NON-SAE NR, S-AE NR
Cerdan-De-las-heras et al. (2022), [151], Denmark	54 (27/27), mean age 70, 50% female, BMI NR, SES NR	UC + Rehab (Pulmonary rehabilitation)	Combined digital and in-person, 8 wks, (26 wks), Exercise therapy	App + device [Virtual Autonomous Physiotherapist Agent (VAPA), Physio R&D and Cortrium, Copenhagen, Denmark, and bookBeo, Le Faou, France and Laster Technologies, Paris, France] (mHealth + Device)	Obj PA (Pedometer [steps/wk]), Obj PF (6MWT), ANX (GAD7), HRQOL (SGRQ)	NON-SAE NR, S-AE NR
Chan et al. (2022), [102], China	139 (70/69), mean age 60, 29% female, 26, SES NR	UC + MI (Brief session on healthy eating and breathing exercise information (15 min) + picture e-messages)	Digital only, 12 wks, Exercise therapy	App [ZTExApp] + WhatsApp (ZTEx picture e-messages as exercise reminders) (mHealth)	Obj PA (Pedometer [steps/d]), Obj PF (30s chair stand test), HRQOL (WHOQOL-BREF)	NON-SAE (), S-AE NR
Chaplin et al. (2016 + 2022), [152, 153], UK	103 (51/52), mean age 66, 31% female, 29, SES NR	UC + Rehab (Pulmonary rehabilitation)	Combined digital and in-person, 12 wks, Exercise therapy	Website + email or telephone call (eHealth)	Obj PA (Accelerometer [steps/d]), Obj PF (ESWT)	NON-SAE (Comorbidities + COPD ill health), S-AE ()
Chokshi et al. (2017), [62], USA	105 (50/55), mean age 60, 31% female, 31, not low SES (5%	UC + MI (Device)	Digital only, 16 wks, (24 wks), Physical activity	Wearable [Misfit Shine activity tracker] + app + text messages, e-mails, or	Obj PA (Activity tracker [steps/d])	NON-SAE (No events occurred), S-AE (Hospitalization)

	Some high school)			interactive voice recordings (Device)		
Chow et al. (2015), [80], Australia	710 (352/358), mean age 58, 18% female, 30, SES NR	UC + MI + Rehab (Text messages) (Pulmonary rehabilitation)	Digital only, 24 wks, Physical activity	Text messages (mHealth)	Subj PA (GPAQ [METs min/wk])	NON-SAE NR, S-AE (Death(s))
Cicolini et al. (2014), [103], Italy	203 (102/101), mean age 59, 49% female, 29, SES NR	UC + MI (Education)	Digital only, 26 wks, Physical activity	E-mails + telephone calls (eHealth)	Subj PA (PA [min/d])	NON-SAE NR, S-AE NR
Clays et al. (2021), [154], Belgium + Italy	61 (38/23), mean age 63, 23% female, 29, SES NR	UC	Combined digital and in-person, 26 wks, Exercise therapy	Smartphone [Nokia 6 TA_1021] + App [HeartMan] + Weareble [wristband sensor developed by BITTIUM, Oulo, Finlan)] + Device [Blood pressure monitor + Weight scale + Pill organizer] (mHealth + Device)	Obj PF (6MWT), DEP (BDI-II), ANX (STAI-Y), HRQOL (MLHFQ)	NON-SAE NR, S-AE (Hospitalisation + death(s))
Connelly et al. (2017), [97], Scotland	16 (11/5), mean age 67, 38% female, 33, SES NR	UC + MI (Leaflets on diabetes-specific physical activity advice based on the website material)	Digital only, 26 wks, Physical activity	Website (eHealth)	Obj PA (Accelerometer [steps/wk])	NON-SAE (), S-AE NR
Connelly et al. (2017), [104], Scotland	15 (10/5), mean age 66, 20% female, 31, SES NR	UC + MI (Leaflets on diabetes-specific physical activity advice based on the website material)	Digital only, 26 wks, Physical activity	Website (eHealth)	Obj PA (Accelerometer [steps/wk])	NON-SAE (Ill health), S-AE NR
Coombes et al. (2021), [187], Australia	30 (14/16), mean age 58, 33% female, BMI NR, not low SES (23% High school)	UC + MI (4 exercise sessions (1h))	Combined digital and in-person, 12 wks, Physical activity	Wearable [Lynk2 wrist-worn monitor, Accuro, Oakbrook Terrace, IL] + app [PAI Health app, PAI Health, Vancouver, Canada] (mHealth + Device)	Obj PA (Accelerometer [MVPA min·wk−1]), Subj PF (SF-36 [PF])	NON-SAE (Mild hypoglycemic episode + health reason), S-AE NR
Coultas et al. (2016), [63], USA	305 (149/156), mean age 70, 50% female, BMI NR, SES NR	UC	Combined digital and in-person, 20 wks, Physical activity	Telephone calls [manual + automated] (mHealth)	Obj PF (6MWT), Subj PA (RAPA active [no])	NON-SAE (Injury/fall + other medical conditions), S-AE (Cardiac exacerbation +

						hospitalization + death(s))
Cox et al. (2022), [82], Australia	96 (47/49), mean age 0, 0% female, BMI NR, SES NR	UC + Rehab (Pulmonary rehabilitation)	Combined digital and in-person, 8 wks, (52 wks), Exercise therapy	Video conferencing [Zoom] + Tablet [Apple iPad, Apple, Cupertino, California, USA] + Device [pulse oximeter, Nonin Palmsat 2500A, Nonin Medical, Plymouth, Minnesota, USA] (eHealth + Device)	Obj PA (Accelerometer [MVPA min/d]), Obj PF (6MWT), DEP (HADS-D), ANX (HADS-A), HRQOL (SF-36 v2 [PCS])	NON-SAE NR, S-AE NR
De Greef et al. (2011), [155], Belgium	92 (60/32), mean age 62, 31% female, 30, SES NR	UC	Combined digital and in-person, 24 wks, (52 wks), Physical activity	Telephone calls + wearable [pedometer] (mHealth)	Obj PA (Accelerometer [total active min/d])	NON-SAE NR, S-AE (Hospitalization)
de Sousa Pinto et al. (2014), [105], Spain	50 (29/21), mean age 70, 5% female, 26, Low SES (88% No education or primary school)	UC + MI (Nursing counseling)	Digital only, 12 wks, Exercise therapy	Telephone calls (mHealth)	Obj PF (6MWT), HRQOL (SGRQ)	NON-SAE (No events occurred), S-AE NR
Deka et al. (2018), [64], USA	30 (15/15), mean age 68, 37% female, BMI NR, SES NR	UC + MI (Exercise therapy + device) (Cardiac rehabilitation)	Combined digital and in-person, 8 wks, Exercise therapy	Wearable [Fitbit Charge HR] + video conferences [Vidyo software] (eHealth + Device)	Obj PF (6MWT), Subj PA (Exercise [min/wk])	NON-SAE (), S-AE NR
Demeyer et al. (2017), [156], Belgium + England + Greece + Switzerland + the Netherlands	343 (171/172), mean age 67, 36% female, 26, SES NR	UC + MI (Discussion with the investigator (5 to 10 min))	Combined digital and in-person, 12 wks, Physical activity	Wearable [Fitbug Air] + app [Fitbug] + text messages + telephone calls (mHealth + Device)	Obj PA (Accelerometer [steps/d]), Obj PF (6MWT)	NON-SAE (Exacerbations + musculoskeletal pain), S-AE NR
Dobler et al. (2018), [157], Germany	249 (123/126), mean age 52, 29% female, 36, not low SES (30% Unemployed)	UC + MI (Education) (Rehabilitation program (3 wks))	Combined digital and in-person, 52 wks, Physical activity	Telephone calls (mHealth)	Subj PA (Exercise index), DEP (PHQ-9)	NON-SAE NR, S-AE (Severe illness)
Doiron-Cadrin et al. (2020), [158], Canada	23 (12/11), mean age 68, 65% female, 31, not low SES (35% <50.000 dollars/y)	UC + MI (Home visit from a community-based PT)	Combined digital and in-person, 12 wks, Exercise therapy	App [REACTS LiteVR medical consultation application, Technologies innovatrices d'imagerie, Montreal, Canada] +	Obj PF (TUG), HRQOL (SF-36 [PCS])	NON-SAE NR, S-AE NR

				Skype® [Microsoft Corporation, Redmond, USA] + Facetime® [Apple, Cupertino, USA] + tablet [Ipad®] (mHealth + eHealth)		
Duan et al. (2018), [106], China	114 (60/54), mean age 49, 57% female, 24, SES NR	UC (WL)	Digital only, 8 wks, Physical activity	Website + e-mails + text messages + telephone calls (eHealth)	Subj PA (IPAQ [total PA min/wk]), DEP (CES-D), HRQOL (WHO [HRQOL])	NON-SAE NR, S-AE NR
Duruturk et al. (2019), [159], Turkey	50 (25/25), mean age 53, 41% female, 31, SES NR	UC + MI (Education session)	Combined digital and in-person, 6 wks, Exercise therapy	Video conferences (eHealth)	Obj PF (6MWT), DEP (BDI)	NON-SAE NR, S-AE NR
Dyson et al. (2010), [107], UK	42 (21/21), mean age 61, 57% female, 31, SES NR	UC (WL)	Digital only, 26 wks, Physical activity	Video (Device)	Obj PA (Pedometer [steps/d]), HRQOL (WHO-5 [Well-being])	NON-SAE NR, S-AE NR
Eakin et al. (2009 + 2010), [83, 197], Australia	434 (228/206), mean age 58, 61% female, 31, Low SES (55% Less than high school)	UC + MI (Feedback on their assessment results + project newsletter)	Digital only, 52 wks, (78 wks), Physical activity	Telephone calls (mHealth)	Subj PA (AAS [MVPA min/wk])	NON-SAE (Health reason + personal illness), S-AE (Death(s))
Eakin et al. (2014), [84], Australia	302 (151/151), mean age 58, 44% female, 33, not low SES (12% Education less than high school)	UC	Combined digital and in-person, 78 wks, (104 wks), Physical activity	Telephone calls (mHealth)	Obj PA (Accelerometer [MVPA min/wk])	NON-SAE (Musculoskeletal problems + digestive disturbance), S-AE (Hospitalization + death(s))
Felker et al. (2022), [65], USA	187 (92/95), mean age 59, 35% female, 35, SES NR	UC + MI (One wkly text message)	Digital only, 12 wks, (26 wks), Physical activity	App + Wearable [Withings Go, Issy-les-Moulineaux, France] + Text messages + Telephone call + Device [D-3 Pillbox, Plectronic tool] (mHealth + Device)	Obj PA (Accelerometer [steps/d]), HRQOL (KCCQ)	NON-SAE NR, S-AE NR
Frederix et al. (2015), [160], Belgium	140 (70/70), mean age 61, 18% female, 28, SES NR	UC + Rehab (Cardiac rehabilitation (12 wks))	Combined digital and in-person, 24 wks, Exercise therapy + physical activity	E-mails + text messages + wearable [Yorbody accelerometer, Belgium] +	Obj PA (Accelerometer [steps/d]), HRQOL (HeartQoL [Physical])	NON-SAE NR, S-AE (Lung cancer)

				website (mHealth + eHealth + Device)		
Furuya et al. (2014), [161], Brazil	90 (45/45), mean age 62, 43% female, BMI NR, SES NR	UC	Combined digital and in-person, 26 wks, Physical activity	Telephone calls (mHealth)	Subj PF (SF-36 [PF]), DEP (HADS-D), ANX (HADS-A), HRQOL (SF-36 [PCS])	NON-SAE NR, S-AE NR
Galdiz et al. (2021), [108], Spain	94 (46/48), mean age 63, 33% female, 27, SES NR	UC	Digital only, 52 wks, Exercise therapy	Mobile phone + app + website + wearable [pulse oximeter] (mHealth)	Obj PF (6MWT), HRQOL (SF-36 [PCS])	NON-SAE NR, S-AE (Exacerbation + exacerbation with ICU admission + long-lasting hospital stay)
Gingele et al. (2019), [102], The Netherlands	382 (197/185), mean age 71, 41% female, BMI NR, SES NR	UC	Digital only, 52 wks, Physical activity	Device [Health buddy messages] (Device)	Subj PA (METs), HRQOL (EQ-5D [index])	NON-SAE NR, S-AE (Death(s))
Glasgow et al. (2012), [66], USA	235 (169/66), mean age 59, 48% female, 35, not low SES (48% Income less than 50000 USD)	UC + MI (Computer-based health risk appraisal feedback)	Digital only, 52 wks, Physical activity	Website [My Path to Healthy Life] + e-mails + telephone calls [computer-based] (eHealth)	Subj PA (CHAMPS [Calories/wk])	NON-SAE NR, S-AE NR
Glasgow et al. (2012), [66], USA	228 (162/66), mean age 59, 53% female, 35, not low SES (48% Income less than 50000 USD)	UC + MI (Computer-based health risk appraisal feedback)	Combined digital and in-person, 52 wks, Physical activity	Website [My Path to Healthy Life] + e-mails + telephone calls [computer-based] (eHealth)	Subj PA (CHAMPS [Calories/wk])	NON-SAE NR, S-AE NR
Gohir et al. (2021), [110], UK	146 (67/79), mean age 67, 68% female, 31, SES NR	UC	Digital only, 6 wks, Exercise therapy	App [Joint Academy] + e-mails + chat + telephone calls (mHealth)	Obj PF (TUG), HRQOL (MSK-HQ)	NON-SAE (LBP + dizziness), S-AE (No events occurred)
Guiraud et al. (2012), [111], France	29 (19/10), mean age 57, 17% female, 27, not low SES (1% Unemployed)	UC	Digital only, 8 wks, Exercise therapy	Wearable [MyWellness Key] + web-app + telephone calls (mHealth + Device)	Obj PA (Accelerometer [active EE kcal/wk])	NON-SAE NR, S-AE NR
Haller et al. (2018), [162], Germany	20 (14/6), mean age 45, 65% female, 27, not low SES	UC	Combined digital and in-person, 8 wks, Exercise therapy	Website + wearable [heart rate monitor, Polar FT1, Polar Electro, Büttelborn, Germany] + text messages [web-based] (eHealth + Device)	Subj PA (BHPAQ), Subj PF (SF-36 [PF]), DEP (QIDS-SR)	NON-SAE NR, S-AE NR

Hanssen et al. (2007), [113], Norway	288 (156/132), mean age 60, 19% female, 26, not low SES (30% Primary school)	UC	Digital only, 26 wks, Physical activity	Telephone calls (mHealth)	Subj PA (How often on average are you exercising each wk?), Subj PF (SF-36 [PF]), HRQOL (SF-36 [PCS])	NON-SAE NR, S-AE (Surgery + death(s))
Hawkes et al. (2013), [85], Australia	430 (215/215), mean age 61, 25% female, BMI NR, not low SES (36% Unemployed/low education)	UC + MI (Quarterly informative newsletter)	Digital only, 26 wks, Physical activity	Telephone calls (mHealth)	Subj PA (AAS [min/wk]), Subj PF (SF-36 [PF]), HRQOL (SF-36 [PCS])	NON-SAE NR, S-AE (Depressed/suicidal + death(s))
Hidrus et al. (2020), [114], Malaysia	100 (50/50), mean age 58, 44% female, BMI NR, not low SES	UC + MI (Education brochure)	Digital only, 17 wks, Physical activity	App [WhatsApp group] (mHealth)	Subj PA (IPAQ [total min])	NON-SAE NR, S-AE NR
Hinman et al. (2019), [86], Australia	175 (87/88), mean age 62, 63% female, 31, not low SES (51% Unemployed)	UC + MI (One call from a nurse, with additional calls if required)	Digital only, 26 wks, (52 wks), Exercise therapy	Telephone calls + website (mHealth)	Subj PA (PASE), Subj PF (WOMAC [PF]), HRQOL (AQoL-2)	NON-SAE (Unwell + musculoskeletal problems), S-AE NR
Hochsmann et al. (2019), [115], Switzerland	36 (18/18), mean age 59, 47% female, 32, not low SES	UC + MI (Individual lifestyle counseling (60 min))	Digital only, 24 wks, Physical activity	App (mHealth)	Obj PA (Accelerometer [steps/d])	NON-SAE NR, S-AE NR
Holmen et al. (2014), [116], Norway	76 (51/25), mean age 58, 36% female, 32, not low SES (44% Unemployed)	UC	Digital only, 52 wks, Physical activity	Smartphone + app + device [glucometer, LifeScan OneTouch Ultra Easy] (mHealth + Device)	Subj PA (How often do you exercise?), DEP (CES-D), HRQOL (SF-36 [PCS])	NON-SAE NR, S-AE (Serious disease + death(s))
Holmen et al. (2014), [116], Norway	75 (50/25), mean age 57, 36% female, 31, not low SES (40% Unemployed)	UC	Digital only, 52 wks, Physical activity	Smartphone + app + device [glucometer, LifeScan OneTouch Ultra Easy] + telephone calls + text messages (mHealth + Device)	Subj PA (How often do you exercise?), DEP (CES-D), HRQOL (SF-36 [PCS])	NON-SAE NR, S-AE (Serious disease + death(s))
Hornikx et al. (2015), [117], Belgium	30 (15/15), mean age 67, 0% female, 27, not low SES	UC	Digital only, 4 wks, Physical activity	Wearable [Fitbit Ultra®] + telephone calls (mHealth + Device)	Obj PA (Accelerometer [steps/d]), Obj PF (6MWT)	NON-SAE NR, S-AE (Rehospitalization + death(s))
Horton et al. (2018 + 2021), [163, 164], UK	287 (145/142), mean age 68, 35% female, 28, SES NR	UC + Rehab (Pulmonary rehabilitation)	Combined digital and in-person, 7 wks, (26 wks), Exercise therapy	Telephone calls (mHealth)	Obj PA (Accelerometer [steps/d]), Obj PF (ISWT), DEP (HADS-D), ANX (HADS-A)	NON-SAE NR, S-AE (Hospitalization + death(s))

Hsu et al. (2021), [118], Taiwan	44 (22/22), mean age 66, 48% female, 31, SES NR	UC + MI (Wkly phone calls using app over 12 wks + dietician support)	Digital only, 12 wks, Exercise therapy	Telephone calls + video conference [LINE, FaceTime] (mHealth + eHealth)	Obj PF (TUG)	NON-SAE (No events occurred), S-AE NR
Indraratna et al. (2022), [87], Australia	164 (81/83), mean age 62, 21% female, 29, SES NR	UC	Combined digital and in-person, 26 wks, Physical activity	App + Wearable [activity tacker, Xiaomi MiBand 2] + Device [sphygmomanometer, A&D Medical UA-651BLE + digital weighing scale, A&D Medical UC-352BLE] (mHealth + Device)	Obj PF (6MWT), HRQOL (EQ-5D [VAS])	NON-SAE NR, S-AE (Readmissions + death(s))
Jayasree et al. (2019), [119], India	50 (25/25), mean age 61, 63% female, BMI NR, not low SES (12% Illiterate/primary school)	UC	Digital only, 8 wks, Physical activity	Telephone calls (mHealth)	Subj PA (Instrument not described [Recreational to MPA min/wk])	NON-SAE NR, S-AE NR
Jennings et al. (2019), [88], Australia	436 (220/216), mean age 58, 48% female, 34, not low SES (16% Unemployed)	UC + MI (Modified version of the website)	Digital only, 12 wks, (36 wks), Physical activity	Website + e-mails (eHealth)	Subj PA (IPAQ-P7 [Total PA])	NON-SAE NR, S-AE NR
Jiménez-Reguera et al. (2020), [120], Spain	44 (20/24), mean age 68, 39% female, 26, SES NR	UC	Digital only, 52 wks, Physical activity	App [HappyAir, web-based] (mHealth)	Obj PF (6MWT), HRQOL (EQ-5D)	NON-SAE NR, S-AE NR
Jiwani et al. (2020), [67], USA	17 (11/6), mean age 55, 57% female, 38, Low SES (53% Income <13000 USD)	UC	Combined digital and in-person, 26 wks, Physical activity	Smartphone + apps [LoseIt!, FitNow, Inc, Boston, Massachusetts + Diabetes Connect app, PHRQL Inc, Pittsburgh, Pennsylvania] + device [glucometer, Entra Health Systems LLC, San Diego, California] (mHealth)	Subj PF (PROMIS-57 [PF]), DEP (PROMIS [DEP]), ANX (PROMIS [ANX]), HRQOL (PROMIS [GPH])	NON-SAE NR, S-AE NR
Jaarsma et al. (2021), [121], Germany + Israel + Italy + the Netherlands	605 (305/300), mean age 67, 29% female, 28, not low SES (25% Low education (only primary school))	UC + MI (Education + telephone calls)	Digital only, 13 wks, (52 wks), Exercise therapy	Device [Nintendo Wii] + telephone calls (Device)	Obj PF (6MWT), Subj PA (1h or more of PA/wk [no])	NON-SAE (Medical related issues), S-AE (Death(s))

+ Sweden + USA						
Kavradim et al. (2020), [181], Turkey	66 (33/33), mean age 58, 18% female, BMI NR, Low SES (53% Unemployed)	UC	Combined digital and in-person, 12 wks, Physical activity	Telephone calls (mHealth)	Subj PA (PA [no]), HRQOL (MIDAS [PA])	NON-SAE NR, S-AE NR
Kim et al. (2006), [122], South Korea	51 (28/23), mean age 55, 47% female, BMI NR, not low SES	UC	Digital only, 12 wks, Physical activity	Website + telephone calls (eHealth)	Subj PA (METs*h/wk)	NON-SAE NR, S-AE NR
Kooiman et al. (2018), [123], The Netherlands	72 (40/32), mean age 56, 47% female, 33, not low SES	UC + MI (Visits with a diabetes nurse every 3 months)	Digital only, 12 wks, Physical activity	Wearable [Fitbit Zip; Fitbit Inc, San Francisco, CA] + website + web messages (Device)	Subj PA (MVPA ds/wk)	NON-SAE NR, S-AE NR
Kwon et al. (2018), [124], South Korea	44 (30/14), mean age 64, 18% female, 23, not low SES	UC	Digital only, 12 wks, Physical activity	Apps + wearable [pulse oximeter, Checkme O2, Viatom, China] + website (mHealth)	Obj PF (6MWT)	NON-SAE NR, S-AE NR
Kwon et al. (2018), [124], South Korea	41 (27/14), mean age 64, 18% female, 23, not low SES	UC	Digital only, 12 wks, Exercise therapy + physical activity	Apps + wearable [pulse oximeter, Checkme O2, Viatom, China] + website (mHealth)	Obj PF (6MWT)	NON-SAE NR, S-AE NR
Lambert et al. (2018), [125], UK	62 (32/30), mean age 38, 84% female, BMI NR, not low SES (11% Unemployed)	UC (WL)	Digital only, 8 wks, Physical activity	Website + telephone calls + e-mails (eHealth)	Obj PA (Accelerometer [MVPA min/wk]), DEP (PHQ-8), ANX (GAD-7)	NON-SAE NR, S-AE NR
Lee et al. (2018), [165], South Korea	148 (74/74), mean age 52, 37% female, 26, SES NR	UC	Combined digital and in-person, 26 wks, Physical activity	App [Switch, Huraypositive Inc, Seoul, Korea] + Device [glucometer, CareSens N NFC; i-SENS, Seoul, South Korea] + Wearable [fitness tracker, Fitbit flex; Fitbit Inc., San Francisco, CA, USA] (mHealth + Device)	Subj PA (SDSCA [exercise frequency])	NON-SAE NR, S-AE NR
Lee et al. (2021), [112], South Korea	66 (28/38), mean age 62, 18% female, BMI NR, not low SES (44% <Total monthly family income (10,000 won))	UC	Digital only, 4 wks, (12 wks), Physical activity	Telephone calls (mHealth)	Obj PF (6MWT), Subj PA (METs min/wk), HRQOL (KCCQ)	NON-SAE NR, S-AE NR

Li et al. (2018), [188], Canada	61 (30/31), mean age 62, 82% female, 29, not low SES (5% Low income less than 24000 CAD dollars)	UC (WL)	Combined digital and in-person, 9 wks, Physical activity	Wearable [Fitbit Flex] + telephone calls + e-mails (Device)	Obj PA (Accelerometer [MVPA min/wk]), Subj PF (KOOS ADL), HRQOL (KOOS [QOL])	NON-SAE (No events occurred), S-AE (No events occurred)
Li et al. (2020), [166], Canada	51 (26/25), mean age 65, 82% female, 29, not low SES (4% Gross annual household income <24,000 dollars)	UC (WL) + MI (Education)	Combined digital and in-person, 12 wks, Physical activity	Wearable [Fitbit Flex-2 wristband activity tracker, Fitbit Inc] + web-app [FitViz] (Device)	Obj PA (Accelerometer [steps]), Subj PF (KOOS ADL), DEP (PHQ-9), HRQOL (KOOS [QOL])	NON-SAE (Muscle pain + falls + ankle sprain), S-AE (Vertebral compression during a fall)
Li et al. (2021), [126], China	101 (55/46), mean age 48, 24% female, 25, SES NR	UC + MI (Education + telephone follow-up)	Digital only, 13 wks, Exercise therapy	App [R Plus Health app, Recovery Plus Inc] + weareble [chest-worn heart rate band, Recovery Plus Inc] + telephone calls (mHealth + Device)	Obj PF (YMCA 3-min step-test)	NON-SAE NR, S-AE NR
Li et al. (2022), [167], China	99 (50/49), mean age 65, 22% female, 24, SES NR	UC + MI (Education session (2h))	Combined digital and in-person, 52 wks, Exercise therapy	App [WeChat] + Videos (mHealth)	Obj PF (6MWT), DEP (BDI), ANX (SAI), HRQOL (CAT)	NON-SAE NR, S-AE (Acute exacerbation + hospitalization + ED visits)
Liebreich et al. (2009), [127], Canada	49 (25/24), mean age 54, 59% female, 34, not low SES (33% Unemployed)	UC (WL)	Digital only, 12 wks, Physical activity	Website + message board + e-mails (eHealth)	Subj PA (GLTEQ [METs min/wk])	NON-SAE NR, S-AE NR
Lim et al. (2021), [168], Singapore	204 (105/99), mean age 51, 35% female, 31, SES NR	UC + MI (Device)	Combined digital and in-person, 26 wks, Physical activity	App [the nBuddy Diabetes] + instant messages + device [glucometer, Abbott Laboratories] (mHealth)	Subj PA (PA change [min/wk]),	NON-SAE (Mild hypoglycemia), S-AE (Breast cancer)
Michelsen et al. (2022), [169], Sweden	150 (101/49), mean age 61, 22% female, 27, SES NR	UC + Rehab (Cardiac rehabilitation)	Combined digital and in-person, 25 wks, Physical activity	Web-app [LifePod web-based mobile device app, Cross Technology Solutions AB] (mHealth + eHealth)	Subj PA (Haskell questions on PA)	NON-SAE NR, S-AE (Rehospitalization + death(s))
Moore et al. (2009), [170], UK	27 (14/13), mean age 70, 50% female, BMI NR, not low SES	UC	Combined digital and in-person, 6 wks, Exercise therapy	DVD video (Device)	Obj PF (ISWT)	NON-SAE (Knee pain), S-AE NR

Moy et al. (2015 + 2016), [65, 191], USA	239 (155/84), mean age 67, 6% female, BMI NR, not low SES	UC (WL) + MI (Device)	Digital only, 17 wks, (52 wks), Physical activity	Weareble [Omron HJ-720 ITC pedometer, Omron Healthcare, Inc.] + website + telephone calls (Device)	Obj PA (Pedometer [steps/d]), Subj PF (SGRQ ADL), HRQOL (SGRQ)	NON-SAE NR, S-AE (Death(s))
Nagatomi et al. (2022), [171], Japan	30 (15/15), mean age 64, 47% female, 21, SES NR	UC	Combined digital and in-person, 13 wks, Exercise therapy	Wearable [Fitbit®, Inspire HR, Inc., Miami, FL, USA] + App [Fitbit®] + videos + telephone (mHealth + Device)	Obj PF (6MWT), HRQOL (KCCQ)	NON-SAE (Musculoskeletal pain + chest symptoms/wamble), S-AE (Readmissions)
Namjoo Nasab et al. (2017), [172], Iran	68 (34/34), mean age 52, 52% female, 27, Low SES (68% Elementary or less)	UC	Combined digital and in-person, 13 wks, Physical activity	Telephone calls (mHealth)	Subj PA (DEMQ PA)	NON-SAE NR, S-AE NR
Nelligan et al. (2021), [98], Australia	206 (103/103), mean age 60, 61% female, 31, not low SES (47% Secondary education level)	UC + MI (Access to website + text messages)	Digital only, 24 wks, Exercise therapy	Website [My Knee Exercise] + text messages [automated] + e-mails (mHealth + eHealth)	Subj PA (PASE), Subj PF (WOMAC [PF]), HRQOL (AQoL)	NON-SAE (Knee/other places pain), S-AE
O'Brien et al. (2018), [90], Australia	119 (59/60), mean age 62, 62% female, 33, Low SES (77% Education level (<High school))	UC	Digital only, 26 wks, Physical activity	Telephone calls (mHealth)	Subj PA (AAS [MVPA min/wk]), Subj PF (WOMAC [PF]), DEP (DASS-21 [DEP]), ANX (DASS-21 [ANX]), HRQOL (SF-12 v2 [PCS])	NON-SAE (Diabetes + depression episode + sleep disorder + vestibular disorder + hypertension + skin disorder + headache + influenza + asthma + intestinal disorder + joint disorder/sprain/strain + dorsalgia+ urinary disorder + unspecific falls), S-AE (Chronic viral hepatitis + heart disease + pneumonia + emphysema + diverticular disease + rheumatoid arthritis + lever

						fibrosis/cirrhosis + kidney disease + cystitis + COPD + Gout + open foot/ankle wound)
Osteresch et al. + Wienbergen et al. (2019 + 2021), [174, 173], Germany	281 (138/143), mean age 57, 19% female, 28, SES NR	UC	Combined digital and in-person, 52 wks, (104 wks), Physical activity	Telephone calls + wearable [step counter, Incutex step counter/activity trackers, Medisana ViFit] + emails (mHealth + Device)	Subj PA (IPAQ [kcal/wk]), DEP (PHQ-9), HRQOL (EQ-5D-5L)	NON-SAE NR, S-AE (Stroke + reinfarction + unplanned/cardiovascular rehospitalization + death(s))
Pamungkas et al. (2022), [128], Indonesia	60 (30/30), mean age 55, 72% female, 24, Low SES (53% Education level (<High school))	UC (WL)	Digital only, 12 wks, Physical activity	App (mHealth)	Subj PA (DSMQ [PA subscale])	NON-SAE NR, S-AE NR
Pelle et al. (2020), [129], The Netherlands	427 (214/213), mean age 62, 72% female, 28, not low SES (22% Low education <12y)	UC	Digital only, 26 wks, Physical activity	App [dr. Bart] + e-mails (mHealth)	Subj PA (SQUASH [total PA]), Subj PF (KOOS/HOOS [ADL]), HRQOL (EQ-5D-3L)	NON-SAE NR, S-AE NR
Peng et al. (2018), [175], China	98 (49/49), mean age 66, 41% female, BMI NR, Low SES (60% Monthly family income, Yuan <3000)	UC	Combined digital and in-person, 8 wks, Exercise therapy	Video conferences + instant message [QQ + Wechat software] + telephone calls (eHealth)	Obj PF (6MWT), DEP (HADS-D), ANX (HADS-A), HRQOL (MLHFQ)	NON-SAE NR, S-AE (No events occurred)
Piette et al. (2011), [69], USA	291 (145/146), mean age 56, 52% female, 38, not low SES (32% High school or less)	UC + MI (Education)	Digital only, 52 wks, Physical activity	Telephone calls (mHealth)	Obj PA (Pedometer [Step counts]), Subj PF (SF-12 [PF]), DEP (BDI), HRQOL (SF-12 [PCS])	NON-SAE NR, S-AE NR
Piotrowicz et al. (2015), [176], Poland	111 (77/34), mean age 57, 11% female, 28, not low SES	UC	Combined digital and in-person, 8 wks, Exercise therapy	Telephone calls + device [EHO mini device + blood pressure device + body-weight scale, Pro Plus Company] (mHealth)	Obj PF (6MWT)	NON-SAE (Minor skin reactions due to the electrodes), S-AE (No events occurred)
Piotrowicz et al. (2016), [177], Poland	(46/23), mean age 56, 12% female, BMI NR, not low SES	UC	Combined digital and in-person, 8 wks, Exercise therapy	Telephone calls + device [EHO mini device + blood pressure device + body-weight scale, Pro Plus	DEP (BDI)	NON-SAE NR, S-AE NR

				Company] (mHealth)		
Piotrowicz et al. (2019), [178], Poland	850 (425/425), mean age 62, 11% female, 29, not low SES	UC (Cardiac rehabilitation (optional))	Combined digital and in-person, 9 wks, Exercise therapy	Device [EHO mini device + blood pressure device + body-weight scale, Pro Plus Company] + mobile phone + telephone calls (Device)	Obj PF (6MWT), HRQOL (MOS SF-36)	NON-SAE NR, S-AE (Death(s))
Pitta et al. (2022), [179], Brazil	110 (54/56), mean age 64, 35% female, BMI NR, SES NR	UC + MI (Education)	Combined digital and in-person, 12 wks, Physical activity	Telephone calls (mHealth)	Subj PA (BHPAQ), DEP (HADS-D), ANX (HADS-A)	NON-SAE NR, S-AE (Death(s))
Plotnikoff et al. (2013), [130], Canada	190 (96/94), mean age 62, 46% female, 30, not low SES (57% <$60,000)	UC	Digital only, 52 wks, Physical activity	Telephone calls (mHealth)	Obj PA (Pedometer [3-d steps total]), HRQOL (EQ-5D)	NON-SAE NR, S-AE NR
Plotnikoff et al. (2013), [130], Canada	144 (97/47), mean age 62, 46% female, BMI NR, not low SES (56% <$60,001)	UC	Digital only, 52 wks, (52 wks), Physical activity	Wearable [pedometer] (Device)	Obj PA (Pedometer [3-d steps total]), HRQOL (EQ-5D)	NON-SAE NR, S-AE NR
Poppe et al. (2019), [131], Belgium	42 (24/18), mean age 63, 37% female, 31, not low SES	UC (WL)	Digital only, 5 wks, Physical activity	Website + app + e-mails + telephone calls (eHealth)	Obj PA (Accelerometer [total PA min/d])	NON-SAE NR, S-AE NR
Radhakrishnan et al. (2021), [70], USA	38 (19/19), mean age 0, 47% female, BMI NR, not low SES (0% Education level (<High school))	UC + MI (Devices + transmit daily weight and PA data)	Digital only, 12 wks, (24 wks), Physical activity	App [Heart Health Mountain + Withings Health Mate app] + Wearable [Withings Go] + Device [Withings Body smart weighing scale] (mHealth + Device)	Obj PA (Accelerometer [steps/d]), Subj PF (KCCQ [PF]), HRQOL (KCCQ)	NON-SAE NR, S-AE NR
Reid et al. (2012), [132], Canada + UK	223 (115/108), mean age 56, 16% female, 29, not low SES (53% Working full time)	UC	Digital only, 26 wks, (52 wks), Exercise therapy	Website + e-mails (eHealth)	Obj PA (Pedometer [steps/d]), Subj PF (MacNew [Physical]), HRQOL (MacNew)	NON-SAE NR, S-AE (Hospitalization + surgery + death(s))
Reid et al. (2021), [180], Canada	135 (68/67), mean age 66, 100% female, 28, SES NR	UC	Combined digital and in-person, 50 wks, Physical activity	Wearable [Pedometer] + teleconference + telephone calls (mHealth + eHealth + Device)	Obj PA (Accelerometer [MVPA min/wk]), HRQOL (EQ-5D [VAS])	NON-SAE (Clinical reason/health issue), S-AE (Clinical reason/health issue

						disconnected intervention)
Reid et al. (2021), [180], Canada	314 (158/156), mean age 63, 0% female, 29, SES NR	UC	Combined digital and in-person, 50 wks, Physical activity	Wearable [Pedometer] + teleconference + telephone calls (mHealth + eHealth + Device)	Obj PA (Accelerometer [MVPA min/wk]), HRQOL (EQ-5D [VAS])	NON-SAE (Clinical reason/health issue), S-AE (Clinical reason/health issue disconnected intervention)
Robinson et al. (2021), [71], USA	153 (75/78), mean age 70, 7% female, BMI NR, not low SES (44% Annual income <USD 30000)	UC + MI (Education)	Digital only, 24 wks, Physical activity	Wearable [Fitbit Zip pedometer, Fitbit, San Francisco, CA, USA] + website (eHealth + Device)	Obj PA (Pedometer [steps/d]), Obj PF (6MWT), HRQOL (SGRQ-TS)	NON-SAE (Musculoskeletal problems), S-AE (Serious non-COPD adverse event)
Shi et al. (2022), [133], China	54 (27/27), mean age 51, 22% female, BMI NR, Low SES (57% Monthly family income, CNY (<5000))	UC + MI (Telephone call once a month)	Digital only, 9 wks, Exercise therapy	Device [ECG monitoring] + App [exercise regime + online + real-time chat] (mHealth + Device)	Obj PF (6MWT)	NON-SAE NR, S-AE NR
Snoek et al. (2019), [134], The Netherlands	122 (61/61), mean age 60, 18% female, 28, SES NR	UC + MI (Telephone calls)	Digital only, 26 wks, Physical activity	Smartphone [Samsung Galaxy Ace GT-s5830i, Korea] + wearable [heart rate belt, Zephyr, Annapolis, USA] + website [via smartphone] + telephone calls (mHealth + Device)	Subj PA (IPAQ [total PA METs/min-wk]), DEP (HADS-D), ANX (HADS-A), HRQOL (QLMI)	NON-SAE (Musculoskeletal problems), S-AE (Major cardiac event)
Snoek et al. (2021), [135], Denmark + France + Spain + Switzerland + the Netherlands	179 (89/90), mean age 73, 19% female, 27, SES NR	UC (Monthly telephone calls)	Digital only, 26 wks, (52 wks), Physical activity	Telephone calls [smartphone] + heart rate belt (mHealth)	DEP (PHQ-9), ANX (GAD-7), HRQOL (SF-36 v2)	NON-SAE NR, S-AE NR
Southard et al. (2003), [72], USA	104 (53/51), mean age 62, 25% female, 30, not low SES (14% Income less than 20,000 USD per year)	UC	Digital only, 26 wks, Physical activity	Website + instant messages + discussion group + e-mails (eHealth)	Subj PA (Min/wkly exercise), Subj PF (DASI), DEP (BDI)	NON-SAE NR, S-AE (Major cardiovascular-related event)

Spielmanns et al. (2022), [136], Germany + Switzerland	67 (33/34), mean age 64, 49% female, 24, SES NR	UC + MI (Device + smartphone for the assessments)	Digital only, 26 wks, Exercise therapy	App [Kaia COPD] + wearable [activity tracker, POLAR A370 watch, Polar Electro Europe AG, Steinhausen, Switzerland] + telephone calls (mHealth)	Obj PA ([steps/d]), Obj PF (Sit-to-Stand Test), DEP (HADS-D), ANX (HADS-A), HRQOL (CAT)	NON-SAE NR, S-AE NR
Strom et al. (2013), [137], Sweden	48 (24/24), mean age 49, 83% female, BMI NR, not low SES (21% Less than college)	UC (WL)	Digital only, 9 wks, Physical activity	Wearable [pedometer] + website + instant messages (eHealth + Device)	Subj PA (IPAQ), DEP (BDI-II), ANX (BAI), HRQOL (QOLI)	NON-SAE NR, S-AE NR
Tomita et al. (2009), [73], USA	40 (16/24), mean age 76, 68% female, BMI NR, not low SES (20% Income less than 10,000 USD)	UC	Digital only, 52 wks, Exercise therapy	Computer [PC] + website + videos + e-mails (eHealth)	Subj PA (Total exercise among exercisers [2 times or more/wk]), HRQOL (Perceived QOL)	NON-SAE NR, S-AE (Worsening of CHF + death(s))
Tore et al. (2022), [138], Turkey	50 (25/25), mean age 56, 90% female, 32, Low SES (56% Education level (<High school))	UC + MI (Exercise brochure)	Digital only, 8 wks, Exercise therapy	Video conference [Zoom Metings] (eHealth)	Obj PF (30s chair stand test), Subj PA (IPAQ [METs min/wk]), DEP (HADS-D), ANX (HADS-A), HRQOL (KOOS [QOL])	NON-SAE NR, S-AE (No events occurred)
Tsai et al. (2017), [91], Australia	37 (20/17), mean age 74, 50% female, 28, not low SES	UC	Digital only, 9 wks, Exercise therapy	Laptop computer [HP EliteBook 8560p, CA, USA] + video conferences [VSee, CA, USA] + wearable [pulse oximeter, Nonin Onyx Vantage 9590, MN, USA.] (eHealth)	Obj PA (Accelerometer [steps/d]), Obj PF (6MWT), DEP (HADS-D), ANX (HADS-A), HRQOL (CRDQ)	NON-SAE (No events occurred), S-AE (Death(s))
Utriyaprasit (2010), [139], Thailand	103 (51/52), mean age 63, 30% female, BMI NR, Low SES (65% Low income)	UC + MI (1 visit by researcher + 2 telephone calls)	Digital only, 4 wks, Physical activity	Audiotape (Device)	Subj PF (SIP [total PF]), DEP (POMS [DEP/dejection]), ANX (POMS [ANX/tension])	NON-SAE NR, S-AE NR
van der Weegen et al. (2015), [182], The Netherlands	133 (65/68), mean age 58, 53% female, 29, not low SES (26% Low education)	UC + MI (Device)	Combined digital and in-person, 26 wks, (39 wks), Physical activity	Wearable [Personal Activity Monitor AM300] + app + web-app (Device)	Obj PA (Accelerometer [MVPA min/d (≥3METS)]), HRQOL (RAND-36 [PCS])	NON-SAE NR, S-AE NR

Varney et al. (2014), [92], Australia	94 (47/47), mean age 62, 32% female, 32, SES NR	UC	Digital only, 26 wks, (52 wks), Physical activity	Telephone calls (mHealth)	Subj PA (NZPAQ [min/wk]), DEP (KPDS)	NON-SAE NR, S-AE NR
Vasilopoulou et al. (2014), [183], Greece	100 (50/50), mean age 65, 16% female, 27, SES NR	UC	Combined digital and in-person, 52 wks, Exercise therapy	Telephone calls + video conference + video + wearable [pedometer] + device [spirometry + oximetry, MIR Spirodoc, Spirodoc®, Spiro+Oxi, Rome, Italy] + tablet [Lenovo Smart Tab II7, Bratislava, Slovakia]+ website (mHealth + eHealth)	Obj PA (Accelerometer [MPA min/d]), Obj PF (6MWT), HRQOL (SGRQ)	NON-SAE NR, S-AE NR
Vinitha et al. (2019), [140], India	248 (126/122), mean age 43, 32% female, 27, not low SES (37% Not working)	UC + MI (Personal advice)	Digital only, 104 wks, Physical activity	Text messages (mHealth)	Subj PA (RPAQ [total PA kJ/kg/d]), HRQOL (EQ-5D)	NON-SAE NR, S-AE NR
Vluggen et al. (2021), [184], The Netherlands	478 (234/244), mean age 60, 32% female, 31, not low SES (27% Under-average income)	UC (WL)	Combined digital and in-person, 26 wks,	Website + text messages [web-based] + video messages (eHealth)	Subj PA (SQUASH)	NON-SAE NR, S-AE NR
Waller et al. (2021), [93], Australia	395 (197/198), mean age 62, 49% female, 34, not low SES (32% Education level < 12y)	UC	Digital only, 26 wks, Physical activity	Text messages (mHealth)	Subj PA (Sessions [wk]), HRQOL (SF-12 [PCS])	NON-SAE (Unwell due to health condition), S-AE (Death(s))
Wan et al. (2017), [74, 190], USA	114 (60/54), mean age 69, 2% female, 29, SES NR	UC + MI (Device + written materials about exercise + upload step counts at least monthly via the website)	Digital only, 13 wks, (52 wks), Physical activity	Website + online community forum + wearable [Omron HJ-720 ITC pedometer] (eHealth)	Obj PA (Pedometer [steps/d]), Obj PF (6MWT), DEP (BDI), HRQOL (SGRQ)	NON-SAE (Pulmonary events + headache + congestion + ear pain + rash), S-AE (Abdominal pain + anxiety + skin abscess + kidney problems + broken toe + car accident)

Wang et al. (2021), [141], China	78 (39/39), mean age 64, 29% female, BMI NR, SES NR	UC	Digital only, 52 wks, Physical activity	App (mHealth)	Subj PA (Not exercised [>3 ds/wk]), HRQOL (CAT)	NON-SAE NR, S-AE (Death(s))
Widmer et al. (2017), [75], USA	80 (40/40), mean age 63, 28% female, 31, SES NR	UC + Rehab (Cardiac rehabilitation)	Combined digital and in-person, 13 wks, Exercise therapy	Website or app (mHealth + eHealth)	Subj PA (Exercise change [min/wk]), DEP (Instrument not described), HRQOL (Dartmouth [QOL])	NON-SAE NR, S-AE (ED visits + rehospitalization)
Witham et al. (2011), [185], Scotland	107 (53/54), mean age 80, 33% female, BMI NR, SES NR	UC	Combined digital and in-person, 24 wks, Exercise therapy	Telephone calls (mHealth)	Obj PF (6MWT), DEP (HADS-D), ANX (HADS-A), HRQOL (EQ-5D)	NON-SAE (Joint pain + sciatic pain + angina + increased breathlessness+ bilateral ankle edema + musculoskeletal problems), S-AE (Hospitalizations + death(s))
Wong et al. (2020), [142], China	245 (219/26), mean age 52, 34% female, 26, not low SES (13% Education <primary)	UC	Combined digital and in-person, 26 wks, Physical activity	Website (eHealth)	Subj PA (GSLTPA)	NON-SAE NR, S-AE NR
Wong et al. (2021), [198], China	79 (39/40), mean age 33, 85% female, BMI NR, not low SES (18% Education level (<High school))	UC (WL)	Digital only, 8 wks, Physical activity	App [Lifestyle Hub] + videos (mHealth)	Subj PA (IPAQ [total score METs-min/wk]), Subj PF (SDS), DEP (PHQ-9), ANX (GAD-7), HRQOL (SF-6D)	NON-SAE (No events occurred), S-AE NR
Wootton et al. (2018 + 2019), [94, 186], Australia	95 (49/46), mean age 70, 42% female, 25, SES NR	UC + MI (Walking training)	Combined digital and in-person, 9 wks, Exercise therapy	Wearable [G-Sensor accelerometer, Pedometers Australia, Cannington, WA, Australia] + telephone calls (Device)	Obj PA (Accelerometer [steps/d]), Obj PF (6MWT), HRQOL (SGRQ)	NON-SAE NR, S-AE NR
Young et al. + Drew et al. (2021 + 2022a + 2022b), [95, 143, 144], Australia	125 (62/63), mean age 48, 0% female, 33, not low SES (15% <Post-school qualifications)	UC (WL)	Digital only, 13 wks, (26 wks), Physical activity	Website + text messages (eHealth)	Obj PA (Accelerometer [MVPA % time spent/wk]), DEP (PHQ-9), ANX (GAD7)	NON-SAE NR, S-AE NR

Yudi et al. (2021), [91], Australia	206 (103/103), mean age 57, 16% female, 30, SES NR	UC	Digital only, 8 wks, Physical activity	App [S-CRP] (mHealth)	Obj PF (6MWT), DEP (DS-SF), ANX (HADS-A), HRQOL (EQ-5D)	NON-SAE NR, S-AE (Cardiac/other hospitalization)

A, anxiety; ANX, anxiety; BMI, Body-mass-index; d(s), day(s); D, depression; DEP, depression; HADS, the Hospital Anxiety and Depression scale; HRQOL, health-related quality of life; MI, minimal intervention; min, minutes; mo, month(s); MPA, moderate physical activity; MVPA, moderate-to-vigorous physical activity; NR, Not reported; NON-SAE, non-serious adverse event; Obj PA, objectively measured physical activity; Obj PF, objectively measured physical function; PA, physical activity; PN, project nurse; PT, physiotherapist; SAE, serious adverse event; SES, socioeconomic status; Subj PA, subjectively measured physical activity; Subj PF, subjectively measured physical function; TUG, Timed-up and go; UC, usual care, UK, United Kingdom; UAS, United States of America; WL, waiting list; wk(s), week(s); 6MWT, six-minute walk-test

Supplementary Table 4. The percentage of comorbidities among the participants of each study

Author (year)	DEP	ANX	HF	IHD	COPD	HTN	T2DM	KOA	HOA
Agboola et al. 2016	7.0	.	.	.	.	.	100.0	.	.
Akinci et al. 2018	.	.	.	10.0	.	65.0	100.0	.	.
Alghafri et al. 2018	.	.	.	.	.	45.3	100.0	.	.
Allen et al. 2018	5.3	.	.	.	.	.	.	100.0	.
Allen et al. 2021	.	.	.	.	.	.	.	100.0	.
Alonso-Dominguez et al. 2019	.	.	.	.	.	53.0	100.0	.	.
Anderson et al. 2010	35.3	.	2.7	14.2	3.7	74.6	100.0	.	.
Andrade et al. 2021	.	.	100.0	.	.	43.5	.	.	.
Avila et al. 2018 + 2019	.	.	.	100.0	.	74.0	9.8	.	.
Bailey et al. 2020	.	.	.	.	.	.	100.0	.	.
Baker et al. 2019	.	.	.	.	.	.	.	100.0	.
Bartholdy et al. 2019	.	.	.	.	.	.	.	100.0	.
Bender et al. 2017	.	.	.	.	.	.	100.0	.	.
Bennell et al. 2017	.	.	.	.	.	.	.	100.0	.
Bennell et al. 2018	.	.	.	.	.	.	.	.	100.0
Bennell et al. 2020	.	.	.	.	.	.	.	100.0	.
Benson et al. 2019	25.4	.	.	.	.	.	100.0	.	.
Bentley et al. 2020	.	.	.	.	100.0	.	.	.	.
Benzo et al. 2021	.	.	.	.	100.0	.	.	.	.
Benzo et al. 2022	.	.	.	.	100.0	.	.	.	.
Bernocchi et al. 2018	.	.	100.0	.	100.0	.	.	.	.
Bossen et al. 2013	.	.	.	.	.	.	.	71.0	28.0
Bozorgi et al. 2021	.	.	.	.	.	100.0	.	.	.
Cameron-Tucker et al. 2016	.	.	.	.	100.0	.	.	.	.
Cerdan-De-las-heras et al. 2022	.	15.0	.	.	100.0	.	.	.	.
Chan et al. 2022	.	.	.	100.0	.	.	.	.	.
Chaplin et al. 2016 + 2022	30.0	46.0	.	.	100.0	.	.	.	.
Chokshi et al. 2017	.	.	.	100.0	.	82.8	31.4	.	.
Chow et al. 2015	.	.	.	100.0	.	62.0	32.3	.	.
Cicolini et al. 2014	.	.	.	.	.	100.0	50.0	.	.
Clays et al. 2021	.	.	100.0	.	14.3	55.6	41.8	.	.
Connelly et al. 2017	.	.	.	.	.	.	100.0	.	.
Connelly et al. 2017	.	.	.	.	.	.	100.0	.	.
Coombes et al. 2021	.	.	.	.	.	.	100.0	.	.
Coombes et al. 2022	.	.	.	.	.	.	100.0	.	.
Coultas et al. 2016	34.4	.	14.8	12.8	100.0	57.0	20.3	.	.
Cox et al. 2022	.	.	.	.	100.0	.	.	.	.
De Greef et al. 2011	.	.	.	.	.	.	100.0	.	.
de Sousa Pinto et al. 2014	.	.	.	.	100.0	.	.	.	.
Deka et al. 2018	.	.	100.0	.	16.0	66.0	33.0	.	.
Demeyer et al. 2017	.	.	.	.	100.0	.	.	.	.
Dobler et al. 2018	55.0	.	.	.	.	.	100.0	.	.

Doiron-Cadrin et al. 2020	.	.	.	.	.	.	.	52.2	47.8
Duan et al. 2018	.	.	100.0	.	.	.	.	.	.
Duruturk et al. 2019	60.0	.	.	.	.	.	100.0	.	.
Dyson et al. 2010	.	.	.	.	.	.	100.0	.	.
Eakin et al. 2009	.	.	.	.	.	85.5	45.4	.	.
Eakin et al. 2014	.	.	.	79.5	.	.	100.0	.	.
Felker et al. 2022	.	.	100.0	.	.	80.0	97.0	.	.
Frederix et al. 2015	.	.	6.4	92.9	.	60.0	.	.	.
Furuya et al. 2014	.	.	.	100.0	.	96.7	.	.	.
Galdiz et al. 2021	.	.	.	.	100.0	.	.	.	.
Gingele et al. 2019	.	.	100.0	.	.	.	.	.	.
Glasgow et al. 2012	.	.	.	.	.	.	100.0	.	.
Glasgow et al. 2012	.	.	.	.	.	.	100.0	.	.
Gohir et al. 2021	.	.	.	.	.	.	.	100.0	.
Guiraud et al. 2012	.	.	.	100.0	.	17.2	17.2	.	.
Haller et al. 2018	100.0	.	.	.	.	.	.	.	.
Hanssen et al. 2007	.	.	.	100.0	.	.	29.5	.	.
Hawkes et al. 2013	.	.	.	100.0	.	54.9	22.3	.	.
Hidrus et al. 2020	.	.	.	.	.	.	100.0	.	.
Hinman et al. 2019	.	.	.	.	.	.	.	100.0	.
Hochsmann et al. 2019	.	.	.	.	.	63.9	100.0	.	.
Holmen et al. 2014	.	.	.	.	.	.	100.0	.	.
Holmen et al. 2014	.	.	.	.	.	.	100.0	.	.
Hornikx et al. 2015	.	.	.	.	100.0	.	.	.	.
Horton et al. 2018 + 2021	30.0	47.7	.	.	100.0	.	.	.	.
Hsu et al. 2021	.	.	.	.	.	.	.	100.0	.
Indraratna et al. 2022	.	.	22.0	78.0	.	51.8	.	.	.
Jayasree et al. 2019	.	.	.	.	.	.	100.0	.	.
Jennings et al. 2019	.	.	.	.	.	.	100.0	.	.
Jiménez-Reguera et al. 2020	.	.	.	.	100.0	.	.	.	.
Jiwani et al. 2020	.	.	.	.	.	.	100.0	.	.
Jaarsma et al. 2021	28.1	23.4	100.0	42.0	18.0	44.0	27.0	.	.
Kavradim 2020	.	.	.	100.0	.	22.6	.	.	.
Kim et al. 2006	.	.	.	.	.	0.5	100.0	.	.
Kooiman et al. 2018	.	.	.	.	.	.	100.0	.	.
Kwon et al. 2018	.	.	.	.	100.0	.	.	.	.
Kwon et al. 2018	.	.	.	.	100.0	.	.	.	.
Lambert et al. 2018	100.0	73.0	.	.	.	.	.	.	.
Lee et al. 2018	.	.	.	.	.	.	100.0	.	.
Lee et al. 2021	.	.	100.0	.	.	24.2	25.8	.	.
Li et al. 2018	.	.	.	.	.	.	.	100.0	.
Li et al. 2020	.	.	.	.	.	.	.	100.0	.
Li et al. 2021	.	.	.	.	.	24.8	100.0	.	.
Li et al. 2022	5.0	58.0	.	15.0	100.0	14.0	.	.	.
Liebreich et al. 2009	.	.	.	.	.	.	100.0	.	.

Lim et al. 2021	.	.	.	.	.	68.1	100.0	.	.
Michelsen et al. 2022	.	.	3.3	100.0	.	39.3	.	.	.
Moore et al. 2009	30.0	50.0	.	.	100.0	.	.	.	.
Moy et al. 2015	.	.	.	.	100.0	.	.	.	.
Nagatomi et al. 2022	.	.	100.0	.	.	20.0	.	.	.
Namjoo Nasab et al. 2017	.	.	.	.	.	.	100.0	.	.
Nelligan et al. 2021	14.1	.	.	.	.	35.4	7.8	100.0	.
O'Brien et al. 2018	.	.	.	.	.	.	.	100.0	.
Osteresch et al. + Wienbergen et al. 2019 + 2021	.	.	.	100.0	.	.	.	.	.
Pamungkas et al. 2022	.	.	.	.	.	.	100.0	.	.
Pelle et al. 2020	.	.	.	.	.	.	.	73.3	26.7
Peng et al. 2018	.	.	100.0	.	.	.	.	.	.
Piette et al. 2011	100.0	.	.	.	.	.	100.0	.	.
Piotrowicz et al. 2015	.	.	100.0	.	.	.	.	.	.
Piotrowicz et al. 2016	39.1	.	100.0	75.4	.	.	.	.	.
Piotrowicz et al. 2019	21.8	.	100.0	.	.	62.8	.	.	.
Pitta et al. 2022	.	.	.	100.0	.	88.2	.	.	.
Plotnikoff et al. 2013	.	.	.	.	.	.	100.0	.	.
Plotnikoff et al. 2013	.	.	.	.	.	.	100.0	.	.
Poppe et al. 2019	.	.	.	.	.	.	100.0	.	.
Radhakrishnan et al. 2021	26.0	.	100.0	.	.	53.0	.	.	.
Reid et al. 2012	.	.	.	100.0	.	.	.	.	.
Reid et al. 2021	.	.	0.7	100.0	.	61.5	.	.	.
Reid et al. 2021	.	.	1.0	100.0	.	56.4	.	.	.
Robinson et al. 2021	.	.	.	.	100.0	.	.	.	.
Shi et al. 2022	.	.	.	100.0	.	.	.	.	.
Snoek et al. 2019	.	.	.	100.0	.	45.9	13.1	.	.
Snoek et al. 2021	.	.	.	100.0	.	74.3	.	.	.
Southard et al. 2003	47.0	.	.	100.0	.	.	.	.	.
Spielmanns et al. 2022	.	.	.	.	100.0	.	.	.	.
Strom et al. 2013	100.0	86.0	.	.	.	.	.	.	.
Tomita et al. 2009	.	.	100.0	.	.	23.0	.	.	.
Tore et al. 2022	.	.	.	.	.	.	.	100.0	.
Tsai et al. 2017	8.1	2.7	.	.	100.0	47.2	.	.	11.1
Utriyaprasit 2010	.	.	.	100.0	.	.	.	.	.
van der Weegen et al. 2015	5.1	.	.	.	41.0	32.3	58.6	.	.
Varney et al. 2014	.	.	.	.	.	.	100.0	.	.
Vasilopoulou et al. 2014	.	.	.	.	100.0	.	.	.	.
Vinitha et al. 2019	.	.	.	.	.	.	100.0	.	.
Vluggen et al. 2021	5.4	.	.	.	.	.	100.0	.	.
Waller et al. 2021	.	.	3.0	.	.	.	100.0	.	.
Wan et al. 2017	25.7	.	7.3	21.1	100.0	57.8	.	.	.
Wang et al. 2021	.	.	.	.	100.0	26.9	11.5	.	.
Widmer et al. 2017	.	.	.	100.0	.	61.3	.	.	.

Witham et al. 2011	19.1	10.6	100.0	62.6	21.5	.	.	.	.
Wong et al. 2020	.	.	.	100.0	.	.	.	.	.
Wong et al. 2021	100.0	69.0	.	.	.	.	.	.	.
Wootton et al. 2018 + 2019	10.5	13.7	.	.	100.0	42.1	.	.	.
Young et al. + Drew et al. 2021 + 2022a + 2022b	100.0	.	.	.	.	.	.	.	.
Yudi et al. 2021	59.0	66.0	1.2	100.0	.	45.2	.	.	.

ANX, anxiety; DEP, depression; COPD, chronic obstructive pulmonary disease; HF, heart failure; HTN, hypertension; HOA, osteoarthritis of the hip; IHD, ischemic heart disease; KOA osteoarthritis of the knee; T2DM, type 2 diabetes.

100.0 indicates the index condition, while lower percentages represent a comorbidity
