# Supplementary material for: Benefits and Harms of Digital Health Interventions Promoting Physical Activity in People With Chronic Conditions: Systematic Review and Meta-Analysis
Source: J Med Internet Res. 2023 Jul 6;25:e46439. doi: 10.2196/46439 (PMC10359919; doi:10.2196/46439)

Multimedia Appendix 4: Forest plots

To manuscript: **Benefits and Harms of Digital Health Interventions Promoting Physical Activity in People with Chronic Conditions: A Systematic Review and Meta-Analysis**

List of forest plots

Introduction to the forest plots
Supplementary Figure 1. Forest plot for objectively measured physical activity at end-of-intervention
Supplementary Figure 2. Forest plot for objectively measured physical function at end-of-intervention
Supplementary Figure 3. Forest plot for objectively measured moderate-to-vigorous physical activity at end-of-intervention
Supplementary Figure 4. Forest plot for daily steps at end-of-intervention
Supplementary Figure 5. Forest plot for six-minute walk test at end-of-intervention
Supplementary Figure 6. Forest plot for subjectively measured physical activity at end-of-intervention
Supplementary Figure 7. Forest plot for subjectively measured physical function at end-of-intervention
Supplementary Figure 8. Forest plot for depression at end-of-intervention
Supplementary Figure 9. Forest plot for anxiety at end-of-intervention
Supplementary Figure 10. Forest plot for health-related quality of life at end-of-intervention
Supplementary Figure 11. Forest plot for adverse events, non-serious at end-of-intervention
Supplementary Figure 12. Forest plot for adverse events, serious at end-of-intervention
Supplementary Figure 13. Forest plot for objectively measured physical activity at follow-up
Supplementary Figure 14. Forest plot for objectively measured physical function at follow-up
Supplementary Figure 15. Forest plot for objectively measured moderate-to-vigorous physical activity at follow-up
Supplementary Figure 16. Forest plot for daily steps at follow-up
Supplementary Figure 17. Forest plot for six-minute walk test at follow-up
Supplementary Figure 18. Forest plot for subjectively measured physical activity at follow-up
Supplementary Figure 19. Forest plot for subjectively measured physical function at follow-up
Supplementary Figure 20. Forest plot for depression at follow-up
Supplementary Figure 21. Forest plot for anxiety at follow-up
Supplementary Figure 22. Forest plot for health-related quality of life at follow-up
Supplementary Figure 23. Forest plot for adverse events, non-serious at follow-up
Supplementary Figure 24. Forest plot for adverse events, serious at follow-up

Introduction to the forest plots

In the following, we have presented a forest plot for each outcome at end-of-intervention and follow-up. Primary outcomes are objectively measured physical activity and physical function. Secondary outcomes are subjectively measured physical activity and physical function, depression, anxiety, health-related quality of life, and non-serious and serious adverse events.

Each Forest plot summarizes the results of the included studies for the individual outcome, sub-grouped under the included chronic conditions (depression or anxiety (mental health condition), ischemic heart disease or heart failure (Heart disease), chronic obstructive pulmonary disease, knee or hip osteoarthritis (Osteoarthritis), hypertension, type 2 diabetes or multimorbidity (two or more of the conditions)).
The effect size and 95% confidence intervals are displayed for each study (blue square with lines) and overall for each subgroup (red diamond). The effect size in each study is calculated as a standardized mean difference (SMD) to allow for interpretation and pooling across different measurements tools and further given a weight based on the precision of the estimate. Studies with narrower 95% confidence intervals and more precise results get weighted higher. The forest plot also shows the overall effect of the summary estimate across all subgroups and is represented by the green line and green diamond, whereas the grey line represents the null line if there were no effect of the digital health interventions.

Abbreviations:
N, numbers
Non-SAE, Non-serious adverse event
No, Participants without an adverse event
SAE, Serious adverse event
SD, Standard deviation
SMD, Standardized mean difference
Yes, Participants with an adverse event
95% CI, 95% confidence interval

Supplementary Figure 1. Forest plot for objectively measured physical activity at end-of-intervention

Study	Treatment N	Treatment Mean	Treatment SD	Control N	Control Mean	Control SD	SMD with 95% CI	Weight (%)
**Mental health condition (depression or anxiety)**								
Lambert et al. 2018	13	97.6	86.4	11	13.0	97.3	0.89 [0.08, 1.71]	0.82
Young et al. + Drew et al. 2021 + 2022a + 2022b	62	0.8	2.2	63	-0.1	2.2	0.41 [0.06, 0.76]	2.59
Heterogeneity: τ^2 = 0.01, I^2 = 11.34%, H^2 = 1.13							0.51 [0.13, 0.88]	
Test of $\theta_i = \theta_j$: Q(1) = 1.13, p = 0.29								
Test of θ = 0: z = 2.65, p = 0.01								
**Heart disease (heart failure or ischemic heart disease)**								
Guiraud et al. 2012	19	543.7	144.1	10	266.7	107.4	2.02 [1.12, 2.93]	0.68
Reid et al. 2012	115	7079.0	3159.0	108	6168.0	3159.0	0.29 [0.02, 0.55]	3.31
Frederix et al. 2015	69	8233.0	5501.0	70	5265.0	5541.4	0.53 [0.20, 0.87]	2.70
Chokshi et al. 2017	49	8705.0	3107.0	55	6841.0	3254.0	0.58 [0.19, 0.97]	2.33
Avila et al. 2018 + 2019	24	6469.0	4090.8	26	6408.0	2231.2	0.02 [-0.53, 0.56]	1.53
Andrade et al. 2021	11	6873.0	2819.0	12	6541.0	2225.0	0.13 [-0.66, 0.92]	0.87
Radhakrishnan et al. 2021	19	2887.0	2821.0	19	2541.0	1604.0	0.15 [-0.48, 0.77]	1.26
Reid et al. 2021	68	225.2	163.0	67	216.9	179.8	0.05 [-0.29, 0.38]	2.71
Reid et al. 2021	158	284.5	205.3	156	284.8	202.3	-0.00 [-0.22, 0.22]	3.69
Chan et al. 2022	23	10206.0	5534.0	21	8068.0	4994.8	0.40 [-0.19, 0.98]	1.38
Felker et al. 2022	74	3752.0	2902.8	78	2919.6	2167.1	0.32 [0.01, 0.64]	2.84
Heterogeneity: τ^2 = 0.06, I^2 = 59.85%, H^2 = 2.49							0.32 [0.12, 0.51]	
Test of $\theta_i = \theta_j$: Q(10) = 27.95, p = 0.00								
Test of θ = 0: z = 3.20, p = 0.00								
**Chronic obstructive pulmonary disease**								
Vasilopoulou et al. 2014	47	349.9	167.6	50	172.5	192.5	0.97 [0.55, 1.39]	2.15
Hornikx et al. 2015	12	984.0	1208.0	15	1013.0	1275.0	-0.02 [-0.76, 0.71]	0.97
Moy et al. 2015	133	3922.0	2491.0	68	3200.0	2489.0	0.29 [-0.00, 0.58]	3.06
Chaplin et al. 2016 + 2022	20	6111.7	2464.2	34	5409.4	3377.7	0.23 [-0.32, 0.77]	1.53
Demeyer et al. 2017	140	4767.0	3606.7	140	4059.0	2746.7	0.22 [-0.01, 0.45]	3.57
Tsai et al. 2017	19	3176.0	2004.0	17	2878.0	2852.0	0.12 [-0.52, 0.76]	1.21
Wan et al. 2017	57	804.0	3954.6	52	0.0	3954.6	0.20 [-0.17, 0.58]	2.43
Wootton et al. 2018 + 2019	23	4526.0	2527.4	20	5241.0	2276.3	-0.29 [-0.88, 0.30]	1.36
Horton et al. 2018 + 2021	26	1074.0	971.8	25	-464.0	2376.6	0.84 [0.28, 1.40]	1.46
Bentley et al. 2020	19	4258.0	2509.1	11	5124.9	2615.2	-0.33 [-1.06, 0.40]	0.99
Benzo et al. 2021	56	-344.8	1657.7	63	-975.8	2596.3	0.28 [-0.08, 0.64]	2.54
Robinson et al. 2021	75	672.9	3399.0	78	-639.4	3596.7	0.37 [0.05, 0.69]	2.85
Benzo et al. 2022	188	7796.1	3337.5	187	7047.5	3248.7	0.23 [0.02, 0.43]	3.86
Cerdan-De-las-heras et al. 2022	27	8999.8	4241.7	27	11984.6	10783.0	-0.36 [-0.89, 0.17]	1.59
Cox et al. 2022	47	-1.0	25.5	49	-3.0	36.6	0.06 [-0.33, 0.46]	2.28
Spielmanns et al. 2022	33	5016.3	5397.2	34	3105.1	2789.3	0.44 [-0.04, 0.92]	1.82
Heterogeneity: τ^2 = 0.04, I^2 = 51.51%, H^2 = 2.06							0.24 [0.10, 0.39]	
Test of $\theta_i = \theta_j$: Q(15) = 29.43, p = 0.01								
Test of θ = 0: z = 3.26, p = 0.00								
**Osteoarthritis (knee or hip)**								
Bossen et al. 2013	27	361.0	131.2	30	358.0	135.5	0.02 [-0.49, 0.53]	1.67
Li et al. 2018	30	29.2	32.9	31	10.8	19.2	0.68 [0.17, 1.19]	1.68
Bartholdy et al. 2019	19	-5.6	64.6	19	14.8	64.6	-0.31 [-0.94, 0.32]	1.25
Li et al. 2020	26	7133.3	3603.3	25	6232.7	3086.1	0.26 [-0.28, 0.81]	1.54
Heterogeneity: τ^2 = 0.09, I^2 = 53.23%, H^2 = 2.14							0.19 [-0.21, 0.58]	
Test of $\theta_i = \theta_j$: Q(3) = 6.42, p = 0.09								
Test of θ = 0: z = 0.91, p = 0.36								
**Type 2 diabetes**								
Dyson et al. 2010	21	1266.0	3212.5	18	-712.0	3212.5	0.60 [-0.03, 1.23]	1.24
De Greef et al. 2011	60	323.0	87.0	32	306.0	102.0	0.18 [-0.24, 0.61]	2.11
Plotnikoff et al. 2013	68	21493.0	7289.7	78	20097.0	7339.2	0.19 [-0.13, 0.51]	2.80
Plotnikoff et al. 2013	62	20821.0	7283.5	39	20097.0	7339.2	0.10 [-0.30, 0.50]	2.28
Eakin et al. 2014	151	1.4	3.7	151	0.0	3.7	0.38 [0.15, 0.61]	3.64
van der Weegen et al. 2015	65	48.2	23.8	68	39.6	19.5	0.39 [0.05, 0.73]	2.67
Agboola et al. 2016	64	14180.0	55038.5	62	8220.0	41592.6	0.12 [-0.23, 0.47]	2.62
Bender et al. 2017	22	7483.0	2415.0	23	6735.0	2363.0	0.31 [-0.27, 0.89]	1.41
Connelly et al. 2017	11	30058.0	16116.0	5	34597.0	17675.0	-0.26 [-1.26, 0.74]	0.57
Connelly et al. 2017	10	37083.0	25387.0	5	34597.0	17675.0	0.10 [-0.91, 1.11]	0.57
Akinci et al. 2018	21	7310.3	914.3	22	6535.9	1869.8	0.51 [-0.08, 1.11]	1.35
Al-Ghafri et al. 2018	33	7034.0	2408.0	36	6278.0	1270.0	0.39 [-0.08, 0.86]	1.86
Alonso-Dominguez et al. 2019	94	10714.0	4550.0	91	8464.0	4731.0	0.48 [0.19, 0.77]	3.07
Hochsmann et al. 2019	18	9783.0	1334.0	17	6552.0	1280.0	2.41 [1.55, 3.27]	0.75
Poppe et al. 2019	26	241.9	47.9	17	267.2	83.1	-0.39 [-0.99, 0.22]	1.32
Bailey et al. 2020	9	7109.3	2986.7	9	7492.2	2769.1	-0.13 [-1.01, 0.75]	0.72
Coombes et al. 2021	12	484.6	216.3	13	441.2	342.6	0.15 [-0.61, 0.90]	0.92
Heterogeneity: τ^2 = 0.05, I^2 = 50.04%, H^2 = 2.00							0.31 [0.15, 0.47]	
Test of $\theta_i = \theta_j$: Q(16) = 37.05, p = 0.00								
Test of θ = 0: z = 3.71, p = 0.00								
**Multimorbidity**								
Piette et al. 2011	145	4499.0	2612.0	146	3314.0	2516.0	0.46 [0.23, 0.69]	3.59
Heterogeneity: τ^2 = 0.00, I^2 = .%, H^2 = .							0.46 [0.23, 0.69]	
Test of $\theta_i = \theta_j$: Q(0) = 0.00, p = .								
Test of θ = 0: z = 3.89, p = 0.00								
**Overall**							0.29 [0.21, 0.37]	
Heterogeneity: τ^2 = 0.03, I^2 = 44.69%, H^2 = 1.81								
Test of $\theta_i = \theta_j$: Q(50) = 107.04, p = 0.00								
Test of θ = 0: z = 7.09, p = 0.00								
Test of group differences: $Q_b(5)$ = 3.85, p = 0.57								

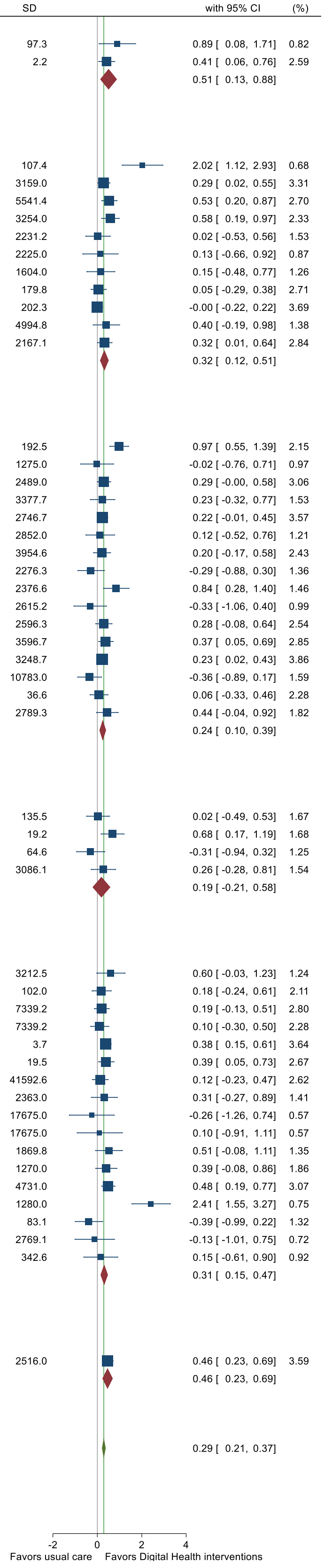

Supplementary Figure 2. Forest plot for objectively measured physical function at end-of-intervention

Study	Treatment N	Treatment Mean	Treatment SD	Control N	Control Mean	Control SD	SMD with 95% CI	Weight (%)
**Heart disease (heart failure or ischemic heart disease)**								
Witham et al. 2011	43	-5.3	64.0	44	0.0	64.0	-0.08 [-0.50, 0.33]	2.24
Piotrowicz et al. 2015	75	480.0	87.0	32	465.0	91.0	0.17 [-0.24, 0.58]	2.24
Deka et al. 2018	15	399.0	102.0	15	368.0	131.0	0.26 [-0.44, 0.96]	1.99
Peng et al. 2018	42	418.3	62.7	41	406.4	80.5	0.16 [-0.26, 0.59]	2.23
Piotrowicz et al. 2019	422	450.0	109.5	423	432.0	106.7	0.17 [0.03, 0.30]	2.38
Jaarsma et al. 2021	243	454.0	123.0	243	420.0	127.0	0.27 [0.09, 0.45]	2.37
Andrade et al. 2021	11	500.7	86.1	12	538.6	72.3	-0.46 [-1.26, 0.34]	1.89
Clays et al. 2021	30	0.9	88.8	17	4.8	39.4	-0.05 [-0.64, 0.53]	2.10
Haejung et al. 2021	7	407.7	67.7	5	421.0	108.5	-0.14 [-1.20, 0.92]	1.62
Yudi et al. 2021	83	564.0	102.9	85	534.6	112.6	0.27 [-0.03, 0.57]	2.31
Chan et al. 2022	70	16.9	6.4	69	15.9	5.0	0.17 [-0.16, 0.50]	2.29
Indraratna et al. 2022	25	469.0	114.0	21	463.0	125.0	0.05 [-0.52, 0.62]	2.11
Nagatomi et al. 2022	15	52.1	43.9	15	-4.3	38.8	1.32 [0.55, 2.10]	1.92
Shi et al. 2022	25	469.4	57.5	26	432.8	67.1	0.58 [0.02, 1.13]	2.13
Heterogeneity: τ^2 = 0.00, I^2 = 0.00%, H^2 = 1.00							0.20 [0.11, 0.28]	
Test of $\theta_i = \theta_j$: Q(13) = 16.84, p = 0.21								
Test of θ = 0: z = 4.56, p = 0.00								
**Chronic obstructive pulmonary disease**								
Moore et al. 2009	10	45.0	51.0	10	-15.0	39.1	1.26 [0.34, 2.19]	1.76
de Sousa Pinto et al. 2014	23	260.0	116.0	18	172.0	93.0	0.81 [0.18, 1.44]	2.05
Vasilopoulou et al. 2014	47	420.2	74.9	50	339.9	110.1	0.84 [0.43, 1.25]	2.24
Hornikx et al. 2015	12	67.0	84.0	15	64.0	59.0	0.04 [-0.70, 0.78]	1.95
Cameron-Tucker et al. 2016	35	0.0	41.0	30	12.0	39.0	-0.30 [-0.78, 0.19]	2.18
Coultas et al. 2016	113	343.1	99.8	134	324.1	107.5	0.18 [-0.07, 0.43]	2.34
Chaplin et al. 2016 + 2022	51	189.0	211.1	52	184.5	247.4	0.02 [-0.36, 0.40]	2.26
Demeyer et al. 2017	150	457.0	108.0	150	449.0	118.0	0.07 [-0.16, 0.30]	2.35
Tsai et al. 2017	19	403.0	82.0	17	374.0	136.0	0.26 [-0.39, 0.90]	2.04
Wan et al. 2017	57	-0.9	55.8	52	2.6	46.7	-0.07 [-0.44, 0.31]	2.27
Kwon et al. 2018	24	389.1	91.4	11	381.3	73.9	0.09 [-0.61, 0.79]	1.99
Kwon et al. 2018	16	381.3	75.9	11	381.3	73.9	0.00 [-0.74, 0.74]	1.94
Wootton et al. 2018 + 2019	42	479.0	86.0	35	468.0	100.0	0.12 [-0.33, 0.56]	2.21
Horton et al. 2018 + 2021	85	281.2	147.9	85	309.5	156.4	-0.19 [-0.49, 0.11]	2.31
Bentley et al. 2020	9	280.0	131.4	6	308.3	61.1	-0.24 [-1.22, 0.73]	1.71
Jiménez-Reguera et al. 2020	17	357.4	112.5	19	339.9	75.9	0.18 [-0.46, 0.82]	2.04
Galdiz et al. 2021	38	441.8	106.3	40	423.7	101.6	0.17 [-0.27, 0.61]	2.22
Robinson et al. 2021	75	25.1	83.2	78	37.4	83.4	-0.15 [-0.46, 0.17]	2.30
Cerdan-De-las-heras et al. 2022	27	434.6	84.5	27	387.1	100.9	0.50 [-0.03, 1.04]	2.14
Cox et al. 2022	47	20.0	51.1	49	26.0	62.7	-0.10 [-0.50, 0.29]	2.25
Li et al. 2022	50	416.3	35.6	49	421.1	66.6	-0.09 [-0.48, 0.30]	2.25
Spielmanns et al. 2022	30	22.7	7.2	30	19.7	6.4	0.42 [-0.08, 0.93]	2.17
Heterogeneity: τ^2 = 0.04, I^2 = 48.21%, H^2 = 1.93							0.13 [-0.01, 0.26]	
Test of $\theta_i = \theta_j$: Q(21) = 39.80, p = 0.01								
Test of θ = 0: z = 1.87, p = 0.06								
**Osteoarthritis (knee or hip)**								
Allen et al. 2018	142	-0.9	5.2	68	-0.2	6.9	-0.11 [-0.40, 0.18]	2.32
Baker et al. 2019	52	-7.5	3.9	52	-7.7	7.2	0.04 [-0.34, 0.43]	2.26
Doiron-Cadrin et al. 2020	12	-8.5	1.4	11	-11.8	4.4	0.99 [0.15, 1.83]	1.85
Allen et al. 2021	230	-5.5	183.4	115	-15.5	236.0	0.05 [-0.17, 0.27]	2.35
Gohir et al. 2021	48	1.8	1.5	57	0.0	1.5	1.19 [0.78, 1.61]	2.24
Hsu et al. 2021	21	-9.4	1.6	21	-9.9	1.7	0.33 [-0.27, 0.93]	2.08
Tore et al. 2022	24	17.3	1.1	24	12.8	2.3	2.45 [1.70, 3.19]	1.95
Heterogeneity: τ^2 = 0.71, I^2 = 94.58%, H^2 = 18.44							0.66 [0.01, 1.32]	
Test of $\theta_i = \theta_j$: Q(6) = 66.80, p = 0.00								
Test of θ = 0: z = 1.98, p = 0.05								
**Type 2 diabetes**								
Akinci et al. 2018	21	539.3	37.4	22	490.9	59.7	0.95 [0.33, 1.57]	2.06
Duruturk et al. 2019	23	554.4	139.0	21	450.9	165.8	0.67 [0.07, 1.26]	2.08
Li et al. 2021	44	-2.0	4.8	41	1.0	5.2	-0.59 [-1.03, -0.16]	2.23
Heterogeneity: τ^2 = 0.62, I^2 = 88.98%, H^2 = 9.08							0.32 [-0.63, 1.27]	
Test of $\theta_i = \theta_j$: Q(2) = 20.63, p = 0.00								
Test of θ = 0: z = 0.66, p = 0.51								
**Multimorbidity**								
Bernocchi et al. 2018	48	60.0	15.4	44	-15.0	10.2	5.63 [4.72, 6.54]	1.78
Heterogeneity: τ^2 = 0.00, I^2 = .%, H^2 = .							5.63 [4.72, 6.54]	
Test of $\theta_i = \theta_j$: Q(0) = 0.00, p = .								
Test of θ = 0: z = 12.14, p = 0.00								
**Overall**							0.36 [0.12, 0.59]	
Heterogeneity: τ^2 = 0.61, I^2 = 94.07%, H^2 = 16.86								
Test of $\theta_i = \theta_j$: Q(46) = 287.35, p = 0.00								
Test of θ = 0: z = 2.93, p = 0.00								
Test of group differences: $Q_b(4)$ = 139.95, p = 0.00								

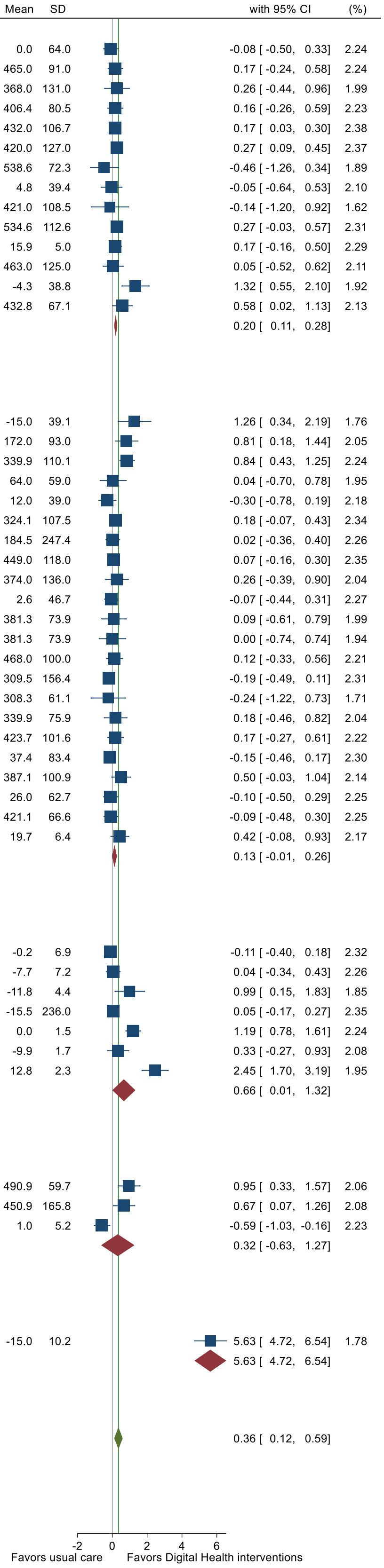

Supplementary Figure 3. Forest plot for objectively measured moderate-to-vigorous physical activity at end-of-intervention

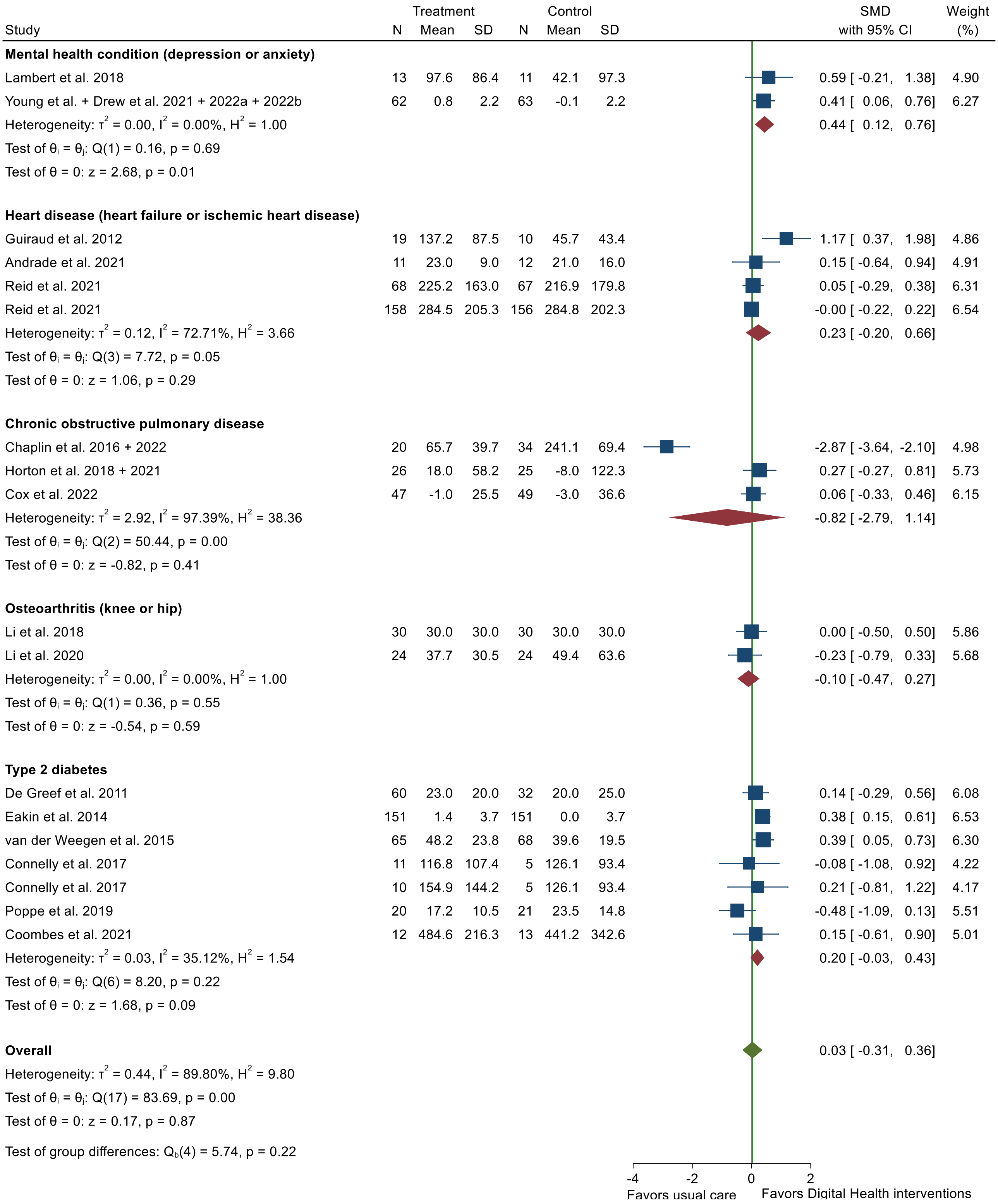

Supplementary Figure 4. Forest plot for daily steps at end-of-intervention

Study	Treatment N	Treatment Mean	Treatment SD	Control N	Control Mean	Control SD	MD with 95% CI	Weight (%)
**Heart disease (heart failure or ischemic heart disease)**								
Reid et al. 2012	115	7079.0	3159.0	108	6168.0	3159.0	911.00 [81.36, 1740.64]	4.76
Frederix et al. 2015	69	8233.0	5501.0	70	5265.0	5541.4	2968.00 [1132.17, 4803.83]	2.07
Chokshi et al. 2017	49	8705.0	3107.0	55	6841.0	3254.0	1864.00 [637.45, 3090.55]	3.41
Avila et al. 2018 + 2019	24	6469.0	4090.8	26	6408.0	2231.2	61.00 [-1746.31, 1868.31]	2.12
Andrade et al. 2021	11	6873.0	2819.0	12	6541.0	2225.0	332.00 [-1734.07, 2398.07]	1.74
Radhakrishnan et al. 2021	19	2887.0	2821.0	19	2541.0	1604.0	346.00 [-1113.16, 1805.16]	2.80
Chan et al. 2022	23	10206.0	5534.0	21	8068.0	4994.8	2138.00 [-987.86, 5263.86]	0.88
Felker et al. 2022	74	3752.0	2902.8	78	2919.6	2167.1	832.40 [20.78, 1644.02]	4.83
Heterogeneity: $\tau^2 = 0.00$, $I^2 = 0.00\%$, $H^2 = 1.00$							1027.72 [582.49, 1472.94]	
Test of $\theta_i = \theta_j$: Q(7) = 9.23, p = 0.24								
Test of θ = 0: z = 4.52, p = 0.00								
**Chronic obstructive pulmonary disease**								
Hornikx et al. 2015	12	984.0	1208.0	15	1013.0	1275.0	-29.00 [-974.80, 916.80]	4.33
Moy et al. 2015	133	3922.0	2491.0	68	3200.0	2489.0	722.00 [-5.65, 1449.65]	5.16
Chaplin et al. 2016 + 2022	20	6111.7	2464.2	34	5409.4	3377.7	702.30 [-996.38, 2400.98]	2.31
Demeyer et al. 2017	140	4767.0	3606.7	140	4059.0	2746.7	708.00 [-42.96, 1458.96]	5.06
Tsai et al. 2017	19	3176.0	2004.0	17	2878.0	2852.0	298.00 [-1298.60, 1894.60]	2.51
Wan et al. 2017	57	804.0	3954.6	52	0.0	3954.6	804.00 [-682.36, 2290.36]	2.74
Wootton et al. 2018 + 2019	23	4526.0	2527.4	20	5241.0	2276.3	-715.00 [-2161.75, 731.75]	2.83
Horton et al. 2018 + 2021	26	1074.0	971.8	25	-464.0	2376.6	1538.00 [548.54, 2527.46]	4.17
Bentley et al. 2020	19	4258.0	2509.1	11	5124.9	2615.2	-866.91 [-2758.59, 1024.77]	1.98
Benzo et al. 2021	56	-344.8	1657.7	63	-975.8	2596.3	631.00 [-162.86, 1424.86]	4.90
Robinson et al. 2021	75	672.9	3399.0	78	-639.4	3596.7	1312.28 [202.51, 2422.05]	3.77
Benzo et al. 2022	188	7796.1	3337.5	187	7047.5	3248.7	748.60 [81.90, 1415.30]	5.40
Spielmanns et al. 2022	33	5016.3	5397.2	34	3105.1	2789.3	1911.20 [-137.15, 3959.55]	1.77
Heterogeneity: $\tau^2 = 0.00$, $I^2 = 0.00\%$, $H^2 = 1.00$							672.99 [391.68, 954.30]	
Test of $\theta_i = \theta_j$: Q(12) = 14.14, p = 0.29								
Test of θ = 0: z = 4.69, p = 0.00								
**Osteoarthritis (knee or hip)**								
Li et al. 2020	26	7133.3	3603.3	25	6232.7	3086.1	900.60 [-944.02, 2745.22]	2.06
Heterogeneity: $\tau^2 = 0.00$, $I^2 = .\%$, $H^2 = .$							900.60 [-944.02, 2745.22]	
Test of $\theta_i = \theta_j$: Q(0) = 0.00, p = .								
Test of θ = 0: z = 0.96, p = 0.34								
**Type 2 diabetes**								
Dyson et al. 2010	21	1266.0	3212.5	18	-712.0	3212.5	1978.00 [-44.45, 4000.45]	1.80
Bender et al. 2017	22	7483.0	2415.0	23	6735.0	2363.0	748.00 [-648.08, 2144.08]	2.96
Akinci et al. 2018	21	7310.3	914.3	22	6535.9	1869.8	774.42 [-112.19, 1661.03]	4.55
Al-Ghafri et al. 2018	33	7034.0	2408.0	36	6278.0	1270.0	756.00 [-141.71, 1653.71]	4.50
Alonso-Dominguez et al. 2019	94	10714.0	4550.0	91	8464.0	4731.0	2250.00 [912.61, 3587.39]	3.11
Hochsmann et al. 2019	18	9783.0	1334.0	17	6552.0	1280.0	3231.00 [2363.92, 4098.08]	4.62
Bailey et al. 2020	9	7109.3	2986.7	9	7492.2	2769.1	-382.90 [-3043.79, 2277.99]	1.16
Heterogeneity: $\tau^2 = 965997.32$, $I^2 = 72.43\%$, $H^2 = 3.63$							1461.61 [566.18, 2357.03]	
Test of $\theta_i = \theta_j$: Q(6) = 24.90, p = 0.00								
Test of θ = 0: z = 3.20, p = 0.00								
**Multimorbidity**								
Piette et al. 2011	145	4499.0	2612.0	146	3314.0	2516.0	1185.00 [595.75, 1774.25]	5.70
Heterogeneity: $\tau^2 = 0.00$, $I^2 = .\%$, $H^2 = .$							1185.00 [595.75, 1774.25]	
Test of $\theta_i = \theta_j$: Q(0) = 0.00, p = .								
Test of θ = 0: z = 3.94, p = 0.00								
**Overall**							970.79 [657.11, 1284.47]	
Heterogeneity: $\tau^2 = 358910.08$, $I^2 = 54.04\%$, $H^2 = 2.18$								
Test of $\theta_i = \theta_j$: Q(29) = 60.31, p = 0.00								
Test of θ = 0: z = 6.07, p = 0.00								
Test of group differences: $Q_b(4) = 5.14$, p = 0.27								

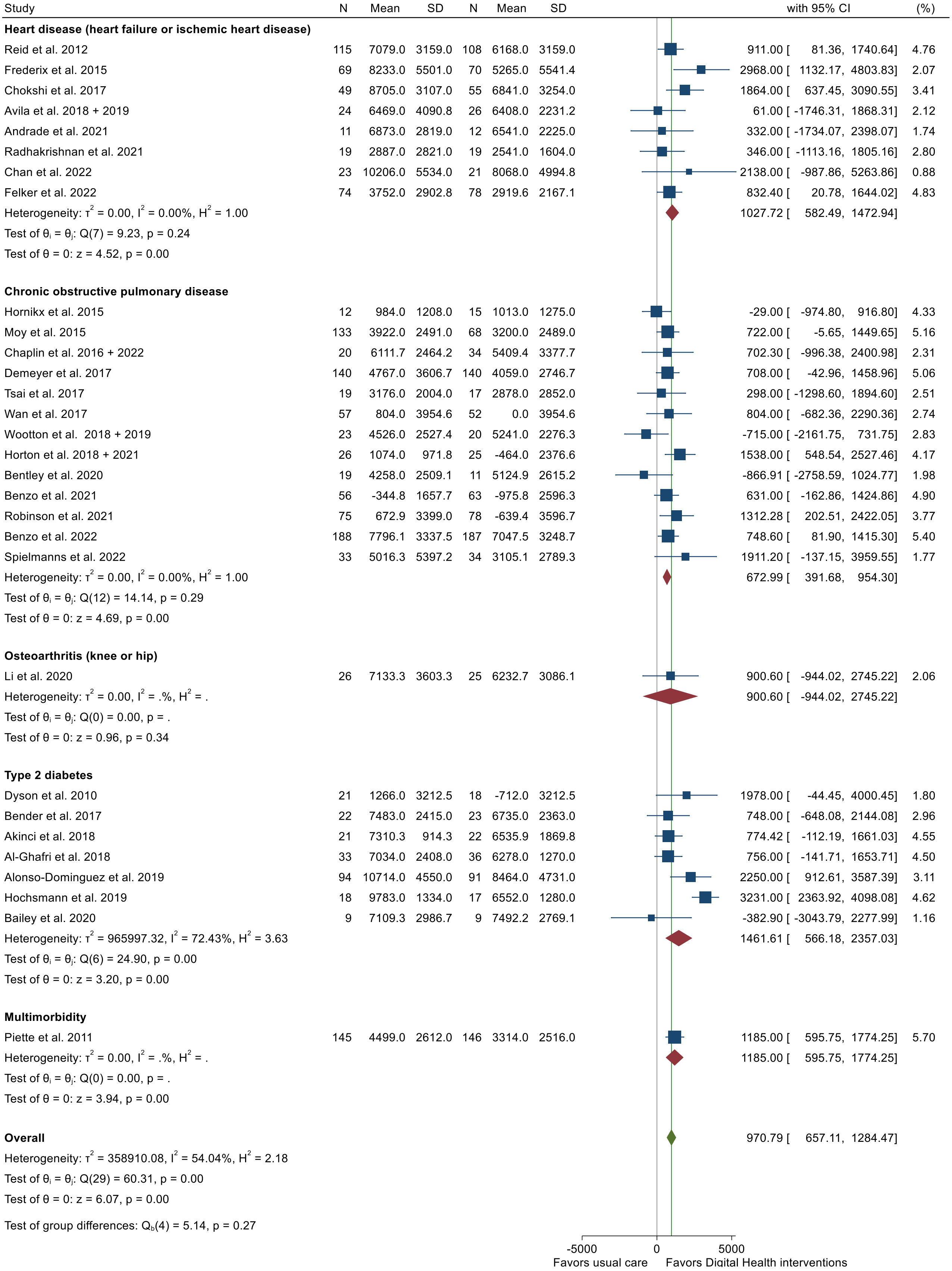

Supplementary Figure 5. Forest plot for six-minute walk test at end-of-intervention

Study	Treatment N	Treatment Mean	Treatment SD	Control N	Control Mean	Control SD	MD with 95% CI	Weight (%)
**Heart disease (heart failure or ischemic heart disease)**								
Witham et al. 2011	43	-5.3	64.0	44	0.0	64.0	-5.30 [-32.21, 21.61]	3.80
Piotrowicz et al. 2015	75	480.0	87.0	32	465.0	91.0	15.00 [-21.50, 51.50]	3.13
Deka et al. 2018	15	399.0	102.0	15	368.0	131.0	31.00 [-53.02, 115.02]	1.17
Peng et al. 2018	42	418.3	62.7	41	406.4	80.5	11.87 [-19.13, 42.87]	3.51
Piotrowicz et al. 2019	422	450.0	109.5	423	432.0	106.7	18.00 [3.42, 32.58]	4.65
Jaarsma et al. 2021	243	454.0	123.0	243	420.0	127.0	34.00 [11.77, 56.23]	4.14
Andrade et al. 2021	11	500.7	86.1	12	538.6	72.3	-37.90 [-102.67, 26.87]	1.71
Clays et al. 2021	30	0.9	88.8	17	4.8	39.4	-3.90 [-48.56, 40.76]	2.63
Haejung et al. 2021	7	407.7	67.7	5	421.0	108.5	-13.33 [-112.47, 85.81]	0.90
Yudi et al. 2021	83	564.0	102.9	85	534.6	112.6	29.40 [-3.24, 62.04]	3.40
Indraratna et al. 2022	25	469.0	114.0	21	463.0	125.0	6.00 [-63.11, 75.11]	1.56
Nagatomi et al. 2022	15	52.1	43.9	15	-4.3	38.8	56.40 [26.75, 86.05]	3.61
Shi et al. 2022	25	469.4	57.5	26	432.8	67.1	36.55 [2.20, 70.90]	3.28
Heterogeneity: τ^2 = 139.72, I^2 = 34.27%, H^2 = 1.52							19.71 [7.95, 31.46]	
Test of $\theta_i = \theta_j$: Q(12) = 17.15, p = 0.14								
Test of θ = 0: z = 3.29, p = 0.00								
**Chronic obstructive pulmonary disease**								
de Sousa Pinto et al. 2014	23	260.0	116.0	18	172.0	93.0	88.00 [22.26, 153.74]	1.67
Vasilopoulou et al. 2014	47	420.2	74.9	50	339.9	110.1	80.30 [42.59, 118.01]	3.05
Hornikx et al. 2015	12	67.0	84.0	15	64.0	59.0	3.00 [-50.96, 56.96]	2.15
Cameron-Tucker et al. 2016	35	0.0	41.0	30	12.0	39.0	-12.00 [-31.55, 7.55]	4.33
Coultas et al. 2016	113	343.1	99.8	134	324.1	107.5	19.00 [-7.05, 45.05]	3.87
Demeyer et al. 2017	150	457.0	108.0	150	449.0	118.0	8.00 [-17.60, 33.60]	3.90
Tsai et al. 2017	19	403.0	82.0	17	374.0	136.0	29.00 [-43.46, 101.46]	1.46
Wan et al. 2017	57	-0.9	55.8	52	2.6	46.7	-3.50 [-22.92, 15.92]	4.34
Kwon et al. 2018	24	389.1	91.4	11	381.3	73.9	7.80 [-53.91, 69.51]	1.82
Kwon et al. 2018	16	381.3	75.9	11	381.3	73.9	0.00 [-57.66, 57.66]	1.98
Wootton et al. 2018 + 2019	42	479.0	86.0	35	468.0	100.0	11.00 [-30.54, 52.54]	2.81
Jiménez-Reguera et al. 2020	17	357.4	112.5	19	339.9	75.9	17.50 [-44.60, 79.60]	1.81
Galdiz et al. 2021	38	441.8	106.3	40	423.7	101.6	18.10 [-28.04, 64.24]	2.54
Robinson et al. 2021	75	25.1	83.2	78	37.4	83.4	-12.27 [-38.68, 14.14]	3.84
Cerdan-De-las-heras et al. 2022	27	434.6	84.5	27	387.1	100.9	47.41 [-2.23, 97.05]	2.36
Cox et al. 2022	47	20.0	51.1	49	26.0	62.7	-6.00 [-28.93, 16.93]	4.09
Li et al. 2022	50	416.3	35.6	49	421.1	66.6	-4.82 [-25.80, 16.16]	4.23
Heterogeneity: τ^2 = 349.66, I^2 = 57.13%, H^2 = 2.33							11.12 [-1.64, 23.87]	
Test of $\theta_i = \theta_j$: Q(16) = 33.42, p = 0.01								
Test of θ = 0: z = 1.71, p = 0.09								
**Osteoarthritis (knee or hip)**								
Allen et al. 2021	230	-5.5	183.4	115	-15.5	236.0	10.00 [-35.31, 55.31]	2.59
Heterogeneity: τ^2 = 0.00, I^2 = .%, H^2 = .							10.00 [-35.31, 55.31]	
Test of $\theta_i = \theta_j$: Q(0) = -0.00, p = .								
Test of θ = 0: z = 0.43, p = 0.67								
**Type 2 diabetes**								
Akinci et al. 2018	21	539.3	37.4	22	490.9	59.7	48.36 [18.43, 78.29]	3.59
Duruturk et al. 2019	23	554.4	139.0	21	450.9	165.8	103.49 [13.36, 193.62]	1.05
Heterogeneity: τ^2 = 345.74, I^2 = 22.75%, H^2 = 1.29							58.86 [16.43, 101.29]	
Test of $\theta_i = \theta_j$: Q(1) = 1.29, p = 0.26								
Test of θ = 0: z = 2.72, p = 0.01								
**Multimorbidity**								
Bernocchi et al. 2018	48	60.0	15.4	44	-15.0	10.2	75.00 [69.59, 80.41]	5.04
Heterogeneity: τ^2 = 0.00, I^2 = .%, H^2 = .							75.00 [69.59, 80.41]	
Test of $\theta_i = \theta_j$: Q(0) = 0.00, p = .								
Test of θ = 0: z = 27.19, p = 0.00								
**Overall**							19.82 [9.45, 30.20]	
Heterogeneity: τ^2 = 547.65, I^2 = 75.86%, H^2 = 4.14								
Test of $\theta_i = \theta_j$: Q(33) = 315.03, p = 0.00								
Test of θ = 0: z = 3.75, p = 0.00								
Test of group differences: $Q_b(4)$ = 134.93, p = 0.00								

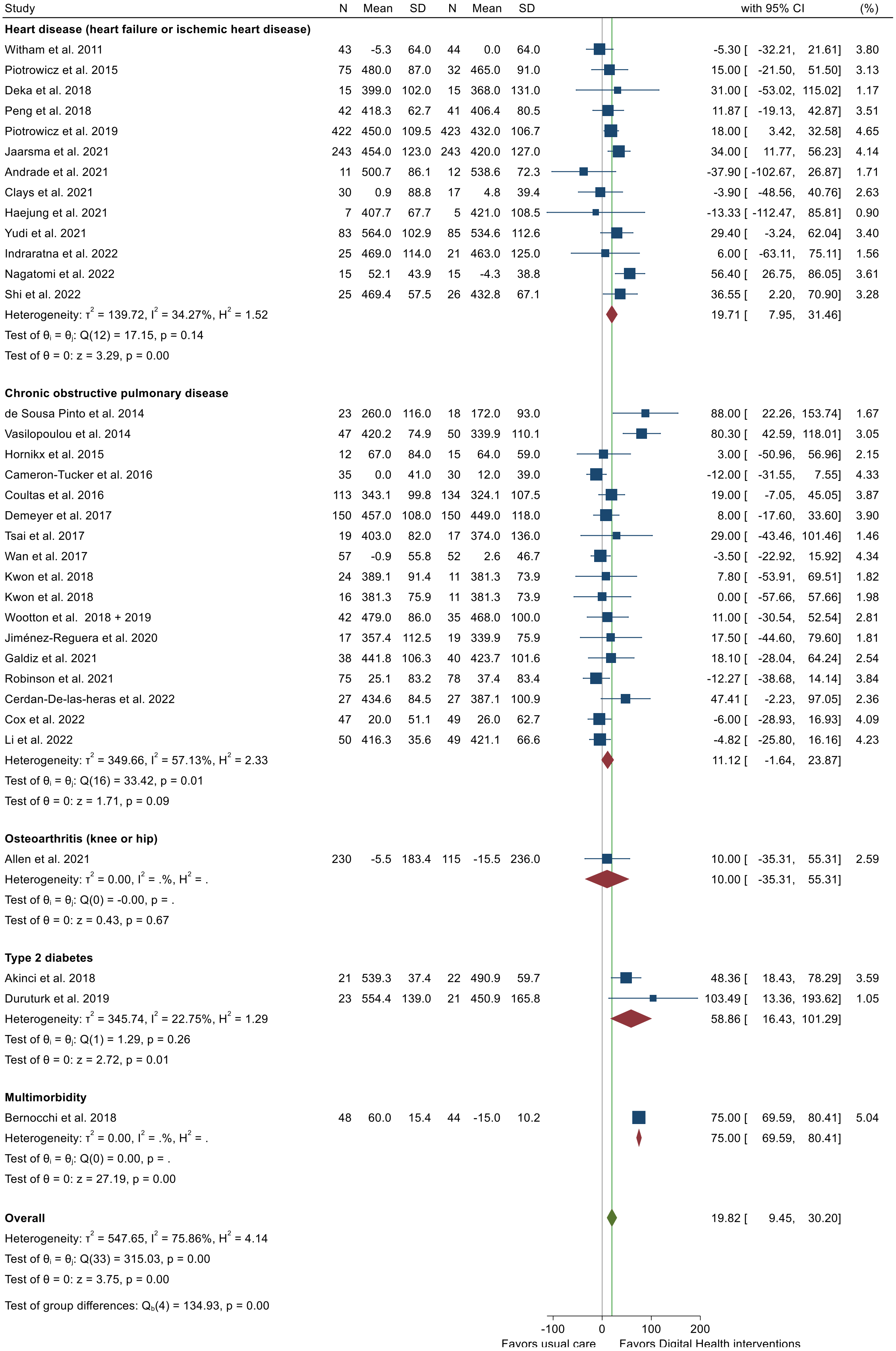

Supplementary Figure 6. Forest plot for subjectively measured physical activity at end-of-intervention

Study	Treatment N	Treatment Mean	Treatment SD	Control N	Control Mean	Control SD	SMD with 95% CI	Weight (%)
**Mental health condition (depression or anxiety)**								
Strom et al. 2013	24	1331.0	990.0	24	1143.0	918.0	0.19 [-0.36, 0.75]	1.36
Haller et al. 2018	14	8.3	1.5	6	7.3	2.2	0.58 [-0.36, 1.51]	0.79
Wong et al. 2021	39	1802.0	2398.1	40	757.6	1232.3	0.54 [0.10, 0.99]	1.60
Heterogeneity: $\tau^2 = 0.00$, $I^2 = 0.00\%$, $H^2 = 1.00$							0.43 [0.10, 0.75]	
Test of $\theta_i = \theta_j$: Q(2) = 1.04, p = 0.59								
Test of $\theta = 0$: z = 2.58, p = 0.01								
**Heart disease (heart failure or ischemic heart disease)**								
Southard et al. 2003	51	208.4	122.4	49	165.0	122.4	0.35 [-0.04, 0.74]	1.71
Hanssen et al. 2007	156	0.9	66.6	132	0.0	66.6	0.01 [-0.22, 0.24]	2.05
Tomita et al. 2009	19	1.4	7.9	13	0.0	7.9	0.17 [-0.52, 0.86]	1.12
Hawkes et al. 2013	156	208.7	207.9	170	200.8	212.8	0.04 [-0.18, 0.25]	2.08
Chow et al. 2015	352	932.0	1024.2	358	587.0	1013.6	0.34 [0.19, 0.49]	2.18
Widmer et al. 2017	37	179.2	109.1	34	139.0	87.7	0.40 [-0.07, 0.87]	1.55
Deka et al. 2018	15	88.2	81.9	15	85.9	44.2	0.03 [-0.66, 0.73]	1.11
Duan et al. 2018	44	218.2	141.7	39	136.2	148.0	0.56 [0.13, 1.00]	1.62
Gingele et al. 2019	179	4.4	1.9	173	3.9	1.7	0.25 [0.04, 0.46]	2.09
Osteresch et al. + Wienbergen et al. 2019 + 2021	134	1850.0	1695.6	136	608.0	1130.4	0.86 [0.61, 1.11]	2.02
Turan Kavradim 2020	31	1.6	21.2	31	0.0	21.2	0.08 [-0.42, 0.57]	1.50
Wong et al. 2020	219	18.2	13.5	219	14.8	12.6	0.26 [0.07, 0.45]	2.13
Jaarsma et al. 2021	201	0.1	4.5	203	0.0	4.5	0.02 [-0.17, 0.22]	2.11
Snoek et al. 2021	61	5008.0	3931.0	61	5265.0	4682.0	-0.06 [-0.41, 0.29]	1.80
Haejung et al. 2021	17	399.8	461.2	17	99.7	282.7	0.77 [0.08, 1.45]	1.13
Michelsen et al. 2022	96	2.6	2.6	48	1.8	2.2	0.32 [-0.03, 0.67]	1.81
Pitta et al. 2022	54	7.9	1.8	56	6.9	1.9	0.55 [0.18, 0.93]	1.74
Heterogeneity: $\tau^2 = 0.05$, $I^2 = 69.08\%$, $H^2 = 3.23$							0.28 [0.15, 0.41]	
Test of $\theta_i = \theta_j$: Q(16) = 48.70, p = 0.00								
Test of $\theta = 0$: z = 4.16, p = 0.00								
**Chronic obstructive pulmonary disease**								
Cameron-Tucker et al. 2016	35	14.0	26.0	30	16.0	40.0	-0.06 [-0.54, 0.42]	1.52
Coultas et al. 2016	113	0.0	4.0	134	0.0	4.0	0.00 [-0.25, 0.25]	2.01
Wang et al. 2021	39	-1.2	4.8	39	0.0	4.8	-0.25 [-0.70, 0.19]	1.60
Heterogeneity: $\tau^2 = 0.00$, $I^2 = 0.00\%$, $H^2 = 1.00$							-0.06 [-0.26, 0.14]	
Test of $\theta_i = \theta_j$: Q(2) = 0.99, p = 0.61								
Test of $\theta = 0$: z = -0.59, p = 0.56								
**Osteoarthritis (knee or hip)**								
Bennell et al. 2018	66	189.0	111.0	65	186.0	80.0	0.03 [-0.31, 0.37]	1.83
Allen et al. 2018	142	-11.5	84.4	68	-4.7	112.4	-0.07 [-0.36, 0.22]	1.94
O'Brien et al. 2018	37	179.7	324.8	52	185.3	383.6	-0.02 [-0.43, 0.40]	1.66
Baker et al. 2019	52	3.6	3.0	52	4.0	4.0	-0.11 [-0.49, 0.27]	1.73
Hinman et al. 2019	83	190.0	91.0	82	172.0	99.0	0.19 [-0.12, 0.49]	1.90
Bennell et al. 2020	56	190.5	111.3	54	174.0	71.0	0.17 [-0.20, 0.55]	1.76
Pelle et al. 2020	214	26.5	17.6	213	26.3	18.1	0.01 [-0.18, 0.20]	2.12
Allen et al. 2021	230	24.6	109.2	115	14.3	143.3	0.08 [-0.14, 0.31]	2.06
Nelligan et al. 2021	103	157.5	75.6	103	144.5	73.0	0.17 [-0.10, 0.45]	1.97
Tore et al. 2022	24	1225.6	829.4	24	727.6	360.8	0.77 [0.19, 1.34]	1.32
Heterogeneity: $\tau^2 = 0.00$, $I^2 = 0.00\%$, $H^2 = 1.00$							0.07 [-0.02, 0.17]	
Test of $\theta_i = \theta_j$: Q(9) = 9.41, p = 0.40								
Test of $\theta = 0$: z = 1.55, p = 0.12								
**Hypertension**								
Eakin et al. 2009	228	71.2	214.4	206	82.2	214.0	-0.05 [-0.24, 0.14]	2.12
Cicolini et al. 2014	100	23.0	7.3	98	13.0	8.1	1.29 [0.99, 1.60]	1.90
Bozorgi et al. 2021	58	260.9	115.9	60	154.6	135.5	0.84 [0.46, 1.21]	1.75
Heterogeneity: $\tau^2 = 0.46$, $I^2 = 95.70\%$, $H^2 = 23.24$							0.68 [-0.10, 1.47]	
Test of $\theta_i = \theta_j$: Q(2) = 60.19, p = 0.00								
Test of $\theta = 0$: z = 1.70, p = 0.09								
**Type 2 diabetes**								
Kim et al. 2006	28	26.7	10.2	23	14.9	7.7	1.27 [0.67, 1.87]	1.29
Liebreich et al. 2009	23	654.0	659.0	21	490.0	562.0	0.26 [-0.32, 0.85]	1.31
Anderson et al. 2010	94	-0.0	2.2	117	0.0	1.7	-0.02 [-0.29, 0.25]	1.97
Glasgow et al. 2012	169	3307.0	3276.0	66	2882.0	2437.2	0.14 [-0.15, 0.42]	1.95
Glasgow et al. 2012	162	3174.0	3245.6	66	2882.0	2437.2	0.10 [-0.19, 0.38]	1.94
Holmen et al. 2014	37	-0.2	24.3	20	0.0	24.3	-0.01 [-0.54, 0.53]	1.40
Holmen et al. 2014	40	-0.3	25.0	20	0.0	25.0	-0.01 [-0.54, 0.52]	1.42
Varney et al. 2014	38	183.0	243.4	43	119.0	243.7	0.26 [-0.17, 0.69]	1.62
Namjoo Nasab et al. 2017	30	8.1	1.7	30	5.8	2.6	1.06 [0.53, 1.60]	1.41
Dobler et al. 2018	98	1.2	2.0	101	0.3	1.9	0.50 [0.21, 0.78]	1.95
Kooiman et al. 2018	36	1.5	3.0	30	0.0	1.8	0.59 [0.10, 1.08]	1.50
Lee et al. 2018	72	3.5	1.8	64	2.9	1.4	0.37 [0.03, 0.70]	1.83
Benson et al. 2019	60	0.1	2.0	58	0.0	2.0	0.07 [-0.29, 0.42]	1.79
Jayasree et al. 2019	25	229.0	379.5	24	197.5	241.6	0.10 [-0.45, 0.65]	1.37
Jennings et al. 2019	195	906.7	3007.9	202	738.2	2376.4	0.06 [-0.13, 0.26]	2.11
Vinitha et al. 2019	126	10.2	10.7	122	10.1	9.6	0.01 [-0.24, 0.26]	2.02
Hidrus et al. 2020	37	357.8	300.7	33	0.0	300.7	1.18 [0.67, 1.68]	1.47
Lim et al. 2021	99	71.4	207.9	105	9.0	122.0	0.37 [0.09, 0.64]	1.96
Vluggen et al. 2021	111	-0.1	1.0	177	0.0	1.0	-0.13 [-0.37, 0.10]	2.04
Waller et al. 2021	197	2.3	4.1	198	2.0	4.4	0.07 [-0.13, 0.26]	2.11
Pamungkas et al. 2022	30	6.9	1.4	30	3.8	1.3	2.34 [1.69, 3.00]	1.18
Heterogeneity: $\tau^2 = 0.24$, $I^2 = 89.44\%$, $H^2 = 9.47$							0.37 [0.14, 0.60]	
Test of $\theta_i = \theta_j$: Q(20) = 106.05, p = 0.00								
Test of $\theta = 0$: z = 3.20, p = 0.00								
**Multimorbidity**								
Bernocchi et al. 2018	48	18.1	64.6	44	-21.3	47.2	0.69 [0.27, 1.10]	1.66
Heterogeneity: $\tau^2 = 0.00$, $I^2 = .\%$, $H^2 = .$							0.69 [0.27, 1.10]	
Test of $\theta_i = \theta_j$: Q(0) = 0.00, p = .								
Test of $\theta = 0$: z = 3.22, p = 0.00								
**Overall**							0.29 [0.19, 0.39]	
Heterogeneity: $\tau^2 = 0.12$, $I^2 = 83.12\%$, $H^2 = 5.93$								
Test of $\theta_i = \theta_j$: Q(57) = 257.05, p = 0.00								
Test of $\theta = 0$: z = 5.52, p = 0.00								
Test of group differences: $Q_b(6) = 24.84$, p = 0.00								

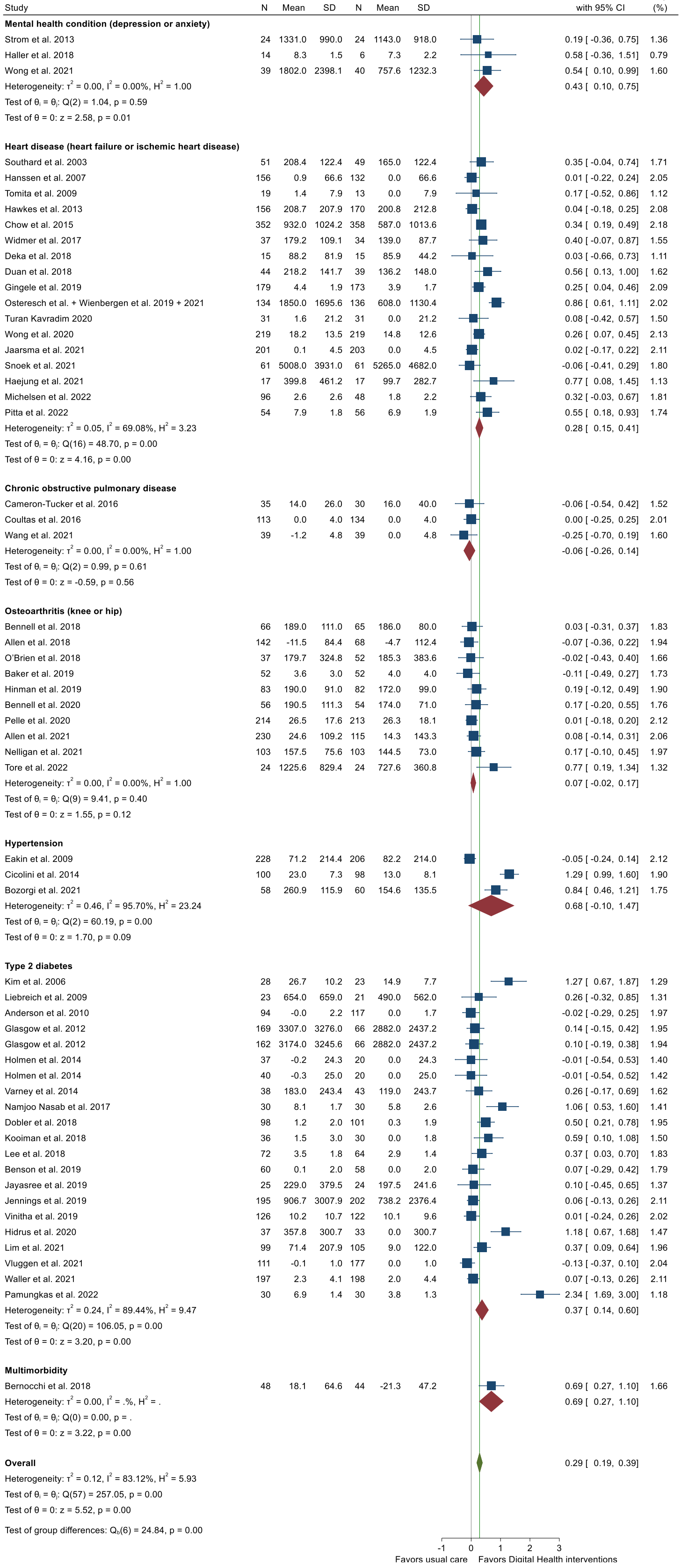

Supplementary Figure 7. Forest plot for subjectively measured physical function at end-of-intervention

Study	Treatment N	Treatment Mean	Treatment SD	Control N	Control Mean	Control SD	SMD with 95% CI	Weight (%)
**Mental health condition (depression or anxiety)**								
Haller et al. 2018	14	83.9	18.3	6	75.8	11.1	0.47 [-0.46, 1.39]	0.68
Wong et al. 2021	39	-7.0	5.9	40	-8.5	7.8	0.21 [-0.22, 0.65]	2.70
Heterogeneity: τ^2 = 0.00, I^2 = 0.00%, H^2 = 1.00							0.26 [-0.14, 0.66]	
Test of $\theta_i = \theta_j$: Q(1) = 0.23, p = 0.63								
Test of θ = 0: z = 1.29, p = 0.20								
**Heart disease (heart failure or ischemic heart disease)**								
Southard et al. 2003	51	44.9	14.6	49	42.3	14.6	0.18 [-0.21, 0.57]	3.28
Hanssen et al. 2007	108	6.6	7.8	99	5.5	7.6	0.15 [-0.12, 0.42]	5.63
Utriyaprasit 2010	51	-15.3	8.1	52	-16.7	8.7	0.17 [-0.21, 0.56]	3.36
Reid et al. 2012	115	5.9	0.9	108	5.7	1.1	0.20 [-0.06, 0.46]	5.91
Hawkes et al. 2013	142	45.5	10.9	156	46.0	10.7	-0.05 [-0.27, 0.18]	7.11
Furuya et al. 2014	30	72.5	23.9	30	64.5	27.8	0.30 [-0.20, 0.81]	2.13
Avila et al. 2018 + 2019	28	91.9	7.6	26	84.3	16.1	0.60 [0.06, 1.14]	1.88
Radhakrishnan et al. 2021	19	3.7	14.1	19	4.1	12.6	-0.03 [-0.65, 0.59]	1.45
Heterogeneity: τ^2 = 0.00, I^2 = 4.69%, H^2 = 1.05							0.14 [0.02, 0.26]	
Test of $\theta_i = \theta_j$: Q(7) = 6.37, p = 0.50								
Test of θ = 0: z = 2.20, p = 0.03								
**Chronic obstructive pulmonary disease**								
Moy et al. 2015	145	60.4	22.7	79	61.9	21.4	-0.07 [-0.34, 0.21]	5.60
Benzo et al. 2022	188	69.1	12.2	187	65.2	12.7	0.31 [0.11, 0.52]	8.06
Heterogeneity: τ^2 = 0.06, I^2 = 79.10%, H^2 = 4.78							0.13 [-0.24, 0.51]	
Test of $\theta_i = \theta_j$: Q(1) = 4.78, p = 0.03								
Test of θ = 0: z = 0.71, p = 0.48								
**Osteoarthritis (knee or hip)**								
Bossen et al. 2013	84	67.8	40.2	80	61.3	27.8	0.19 [-0.12, 0.49]	4.78
Bennell et al. 2017	70	-18.3	10.7	69	-27.6	11.7	0.83 [0.48, 1.17]	3.99
Bennell et al. 2018	66	-17.7	18.0	65	-18.0	12.9	0.02 [-0.32, 0.36]	4.06
Li et al. 2018	30	76.0	16.1	31	71.0	16.5	0.30 [-0.20, 0.80]	2.16
O'Brien et al. 2018	37	36.5	13.2	51	32.8	15.1	0.26 [-0.17, 0.68]	2.88
Bartholdy et al. 2019	19	-1.8	11.3	19	-4.4	11.3	0.23 [-0.40, 0.85]	1.44
Hinman et al. 2019	83	-18.4	11.3	82	-22.0	12.5	0.30 [-0.00, 0.61]	4.78
Bennell et al. 2020	56	72.4	17.6	54	70.0	21.1	0.12 [-0.25, 0.49]	3.54
Li et al. 2020	24	75.0	13.1	24	70.3	16.9	0.31 [-0.25, 0.87]	1.76
Pelle et al. 2020	214	62.1	20.8	213	58.6	19.3	0.17 [-0.02, 0.36]	8.67
Nelligan et al. 2021	103	-16.6	13.0	103	-20.7	13.9	0.30 [0.03, 0.58]	5.58
Heterogeneity: τ^2 = 0.01, I^2 = 33.62%, H^2 = 1.51							0.27 [0.14, 0.40]	
Test of $\theta_i = \theta_j$: Q(10) = 14.02, p = 0.17								
Test of θ = 0: z = 4.09, p = 0.00								
**Type 2 diabetes**								
Jiwani et al. 2020	11	40.2	8.2	6	45.1	11.9	-0.48 [-1.44, 0.47]	0.64
Coombes et al. 2021	12	93.3	9.1	13	85.8	23.0	0.41 [-0.36, 1.18]	0.98
Heterogeneity: τ^2 = 0.20, I^2 = 50.83%, H^2 = 2.03							0.01 [-0.86, 0.88]	
Test of $\theta_i = \theta_j$: Q(1) = 2.03, p = 0.15								
Test of θ = 0: z = 0.02, p = 0.98								
**Multimorbidity**								
Piette et al. 2011	145	39.5	11.7	146	36.3	12.2	0.27 [0.04, 0.50]	6.98
Heterogeneity: τ^2 = 0.00, I^2 = .%, H^2 = .							0.27 [0.04, 0.50]	
Test of $\theta_i = \theta_j$: Q(0) = -0.00, p = .								
Test of θ = 0: z = 2.27, p = 0.02								
**Overall**							0.21 [0.13, 0.29]	
Heterogeneity: τ^2 = 0.01, I^2 = 23.61%, H^2 = 1.31								
Test of $\theta_i = \theta_j$: Q(25) = 30.55, p = 0.20								
Test of θ = 0: z = 5.31, p = 0.00								
Test of group differences: $Q_b(5)$ = 2.74, p = 0.74								

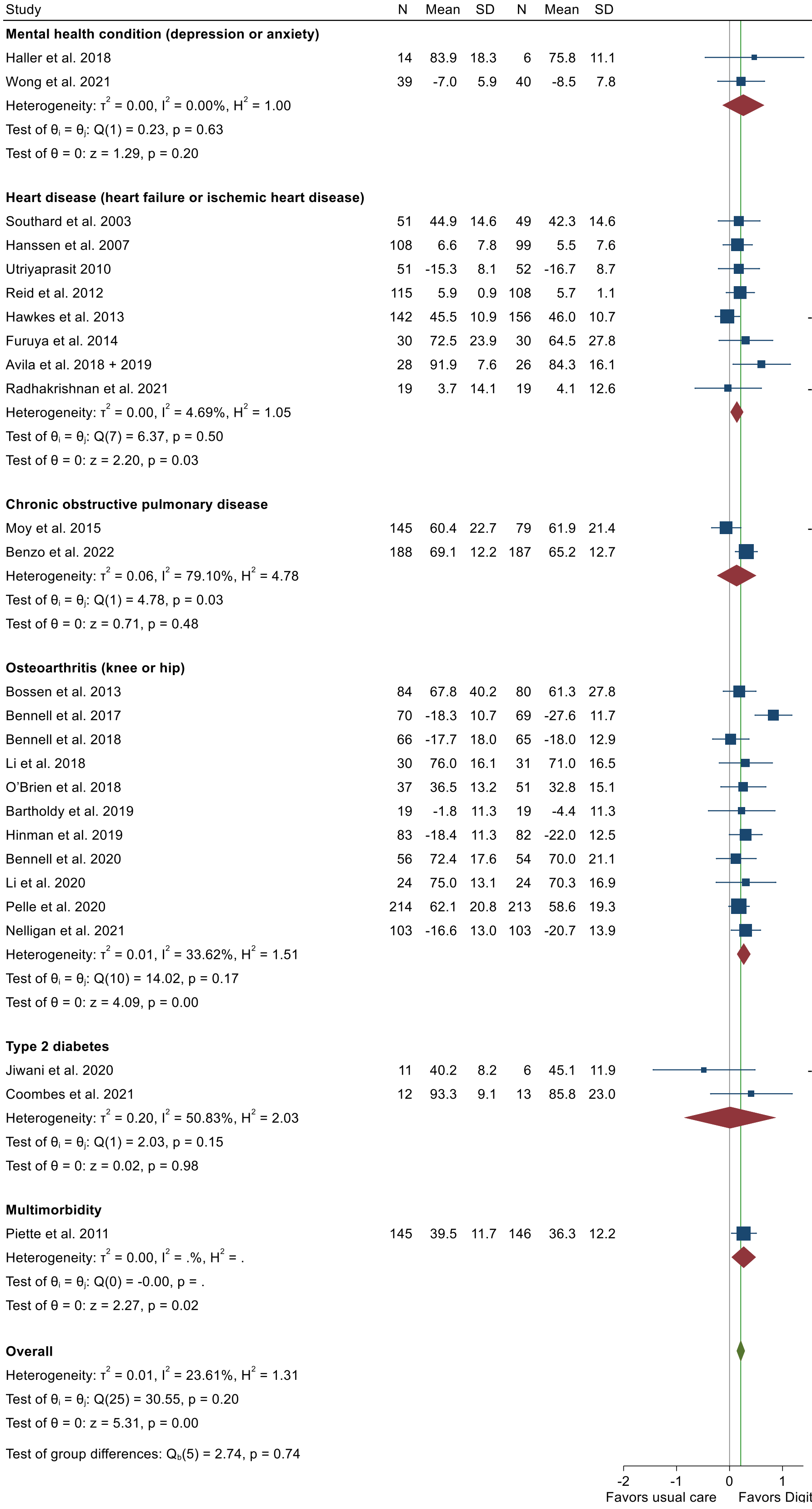

Supplementary Figure 8. Forest plot for depression at end-of-intervention

Study	Treatment N	Treatment Mean	Treatment SD	Control N	Control Mean	Control SD	SMD with 95% CI	Weight (%)
**Mental health condition (depression or anxiety)**								
Strom et al. 2013	24	17.9	11.3	24	24.0	6.9	-0.65 [-1.22, -0.08]	1.83
Haller et al. 2018	14	11.2	3.8	6	11.0	5.2	0.05 [-0.87, 0.96]	0.91
Lambert et al. 2018	25	8.7	4.8	25	12.9	4.2	-0.92 [-1.49, -0.34]	1.82
Wong et al. 2021	39	8.8	3.8	40	11.6	4.7	-0.65 [-1.10, -0.20]	2.44
Young et al. + Drew et al. 2021 + 2022a + 2022b	62	-4.2	4.3	63	-1.8	4.6	-0.54 [-0.89, -0.18]	3.05
Heterogeneity: τ^2 = 0.00, I^2 = 0.00%, H^2 = 1.00							-0.60 [-0.83, -0.38]	
Test of $\theta_i = \theta_j$: Q(4) = 3.28, p = 0.51								
Test of θ = 0: z = -5.31, p = 0.00								
**Heart disease (heart failure or ischemic heart disease)**								
Southard et al. 2003	51	6.8	8.4	49	6.1	8.4	0.08 [-0.31, 0.47]	2.81
Utriyaprasit 2010	51	-4.2	6.4	52	-2.5	5.0	-0.29 [-0.67, 0.10]	2.83
Witham et al. 2011	43	-0.9	2.7	44	0.0	2.7	-0.33 [-0.75, 0.09]	2.61
Furuya et al. 2014	30	5.4	4.8	30	4.7	3.5	0.16 [-0.34, 0.66]	2.16
Piotrowicz et al. 2016	46	6.7	5.5	23	9.1	7.3	-0.38 [-0.88, 0.12]	2.16
Widmer et al. 2017	37	-2.4	3.7	34	0.6	2.3	-0.95 [-1.44, -0.47]	2.23
Duan et al. 2018	44	1.0	0.5	39	1.1	0.6	-0.22 [-0.65, 0.21]	2.56
Peng et al. 2018	42	6.6	6.3	41	6.8	5.5	-0.03 [-0.46, 0.40]	2.57
Osteresch et al. + Wienbergen et al. 2019 + 2021	138	3.6	3.5	143	4.4	3.5	-0.23 [-0.46, 0.01]	3.98
Snoek et al. 2021	61	2.4	2.7	61	3.0	3.2	-0.20 [-0.55, 0.15]	3.05
Andrade et al. 2021	11	68.0	33.0	12	72.0	16.0	-0.15 [-0.94, 0.64]	1.15
Clays et al. 2021	33	-14.0	9.6	22	1.0	14.3	-1.26 [-1.85, -0.68]	1.79
Snoek et al. 2021	89	3.0	3.5	90	4.0	4.4	-0.25 [-0.54, 0.04]	3.51
Yudi et al. 2021	83	13.7	6.6	85	12.5	5.9	0.19 [-0.11, 0.49]	3.44
Pitta et al. 2022	54	2.9	3.4	56	4.3	4.7	-0.34 [-0.71, 0.04]	2.91
Heterogeneity: τ^2 = 0.07, I^2 = 62.76%, H^2 = 2.69							-0.25 [-0.42, -0.08]	
Test of $\theta_i = \theta_j$: Q(14) = 34.83, p = 0.00								
Test of θ = 0: z = -2.94, p = 0.00								
**Chronic obstructive pulmonary disease**								
Tsai et al. 2017	19	4.0	3.0	17	6.0	3.0	-0.65 [-1.31, 0.01]	1.51
Wan et al. 2017	57	-0.6	3.6	52	-0.3	5.3	-0.07 [-0.44, 0.31]	2.92
Horton et al. 2018 + 2021	82	6.1	3.7	86	5.5	3.8	0.16 [-0.15, 0.46]	3.44
Bentley et al. 2020	8	7.5	4.4	6	8.0	5.6	-0.09 [-1.09, 0.90]	0.79
Benzo et al. 2022	188	4.5	4.1	187	5.7	5.0	-0.27 [-0.47, -0.06]	4.23
Cox et al. 2022	47	-0.2	3.2	49	-0.4	4.7	0.05 [-0.35, 0.45]	2.76
Li et al. 2022	50	8.1	4.8	49	6.9	4.0	0.27 [-0.12, 0.66]	2.78
Spielmanns et al. 2022	30	4.2	3.0	30	6.5	5.1	-0.56 [-1.07, -0.05]	2.11
Heterogeneity: τ^2 = 0.04, I^2 = 54.14%, H^2 = 2.18							-0.10 [-0.31, 0.11]	
Test of $\theta_i = \theta_j$: Q(7) = 15.06, p = 0.04								
Test of θ = 0: z = -0.97, p = 0.33								
**Osteoarthritis (knee or hip)**								
Bossen et al. 2013	85	2.6	5.2	78	3.2	5.0	-0.12 [-0.42, 0.19]	3.41
Bennell et al. 2018	66	2.0	3.0	65	2.8	5.1	-0.19 [-0.53, 0.15]	3.14
O'Brien et al. 2018	37	8.7	7.5	52	9.8	9.8	-0.12 [-0.54, 0.30]	2.62
Li et al. 2020	26	4.0	3.0	25	4.7	4.9	-0.17 [-0.71, 0.37]	1.96
Tore et al. 2022	24	2.7	2.4	24	5.4	2.8	-1.03 [-1.62, -0.43]	1.74
Heterogeneity: τ^2 = 0.03, I^2 = 41.64%, H^2 = 1.71							-0.26 [-0.50, -0.01]	
Test of $\theta_i = \theta_j$: Q(4) = 7.82, p = 0.10								
Test of θ = 0: z = -2.08, p = 0.04								
**Type 2 diabetes**								
Holmen et al. 2014	38	9.2	9.2	20	10.8	10.0	-0.17 [-0.70, 0.37]	1.99
Holmen et al. 2014	39	7.6	6.2	20	10.8	10.0	-0.42 [-0.96, 0.12]	1.98
Varney et al. 2014	38	18.0	12.2	43	18.0	9.7	0.00 [-0.43, 0.43]	2.53
Dobler et al. 2018	98	-1.4	4.3	101	-0.2	4.9	-0.24 [-0.52, 0.04]	3.63
Duruturk et al. 2019	23	9.9	8.1	21	11.3	8.2	-0.17 [-0.75, 0.42]	1.79
Jiwani et al. 2020	11	46.6	12.9	6	44.0	7.4	0.22 [-0.73, 1.16]	0.86
Heterogeneity: τ^2 = 0.00, I^2 = 0.00%, H^2 = 1.00							-0.18 [-0.37, -0.00]	
Test of $\theta_i = \theta_j$: Q(5) = 2.30, p = 0.81								
Test of θ = 0: z = -1.96, p = 0.05								
**Multimorbidity**								
Piette et al. 2011	145	14.2	10.3	146	18.6	10.7	-0.42 [-0.65, -0.19]	4.00
Heterogeneity: τ^2 = 0.00, I^2 = .%, H^2 = .							-0.42 [-0.65, -0.19]	
Test of $\theta_i = \theta_j$: Q(0) = 0.00, p = .								
Test of θ = 0: z = -3.53, p = 0.00								
**Overall**							-0.25 [-0.35, -0.16]	
Heterogeneity: τ^2 = 0.05, I^2 = 53.49%, H^2 = 2.15								
Test of $\theta_i = \theta_j$: Q(39) = 80.30, p = 0.00								
Test of θ = 0: z = -5.16, p = 0.00								
Test of group differences: $Q_b(5)$ = 13.31, p = 0.02								

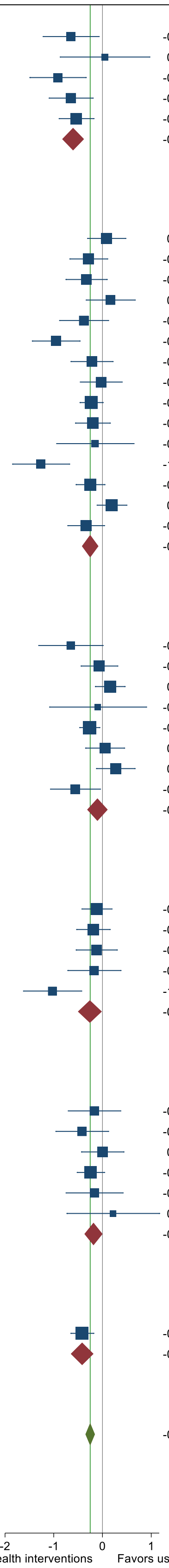

Supplementary Figure 9. Forest plot for anxiety at end-of-intervention

Study	Treatment N	Treatment Mean	Treatment SD	Control N	Control Mean	Control SD	SMD with 95% CI	Weight (%)
**Mental health condition (depression or anxiety)**								
Strom et al. 2013	24	12.9	6.4	24	13.7	5.3	-0.13 [-0.69, 0.42]	2.23
Lambert et al. 2018	25	7.1	3.8	25	10.9	3.7	-1.00 [-1.58, -0.42]	2.07
Wong et al. 2021	39	7.8	3.2	40	8.5	7.8	-0.12 [-0.55, 0.32]	3.41
Young et al. + Drew et al. 2021 + 2022a + 2022b	62	-3.3	3.7	63	-2.1	4.0	-0.31 [-0.66, 0.04]	4.91
Heterogeneity: $\tau^2 = 0.07$, $I^2 = 55.18\%$, $H^2 = 2.23$							-0.36 [-0.71, -0.01]	
Test of $\theta_i = \theta_j$: Q(3) = 6.51, p = 0.09								
Test of $\theta = 0$: z = -2.01, p = 0.04								
**Heart disease (heart failure or ischemic heart disease)**								
Utriyaprasit 2010	51	-3.8	2.6	52	-3.8	2.8	0.01 [-0.37, 0.40]	4.25
Witham et al. 2011	43	0.1	2.3	44	0.0	2.3	0.04 [-0.37, 0.46]	3.70
Furuya et al. 2014	30	5.1	4.4	30	7.6	4.1	-0.58 [-1.09, -0.07]	2.61
Peng et al. 2018	42	6.5	6.0	41	6.8	4.7	-0.05 [-0.48, 0.37]	3.56
Snoek et al. 2021	61	3.4	2.8	61	4.0	3.5	-0.19 [-0.54, 0.17]	4.84
Clays et al. 2021	32	-4.5	7.8	22	1.0	3.2	-0.86 [-1.41, -0.30]	2.22
Snoek et al. 2021	89	1.8	3.1	90	2.4	4.0	-0.17 [-0.46, 0.13]	6.46
Yudi et al. 2021	83	8.4	7.2	85	8.2	6.8	0.03 [-0.27, 0.33]	6.18
Pitta et al. 2022	54	3.2	3.1	56	4.5	4.3	-0.32 [-0.70, 0.05]	4.43
Heterogeneity: $\tau^2 = 0.01$, $I^2 = 22.23\%$, $H^2 = 1.29$							-0.17 [-0.32, -0.03]	
Test of $\theta_i = \theta_j$: Q(8) = 12.75, p = 0.12								
Test of $\theta = 0$: z = -2.36, p = 0.02								
**Chronic obstructive pulmonary disease**								
Tsai et al. 2017	19	4.0	4.0	17	6.0	3.0	-0.55 [-1.20, 0.10]	1.67
Horton et al. 2018 + 2021	81	7.2	4.0	86	6.7	3.8	0.10 [-0.20, 0.41]	6.15
Benzo et al. 2022	188	1.1	1.4	187	1.2	1.4	-0.10 [-0.30, 0.10]	10.35
Cerdan-De-las-heras et al. 2022	27	1.8	2.4	27	4.9	6.8	-0.59 [-1.13, -0.05]	2.38
Cox et al. 2022	47	-1.0	4.9	49	-0.5	5.2	-0.10 [-0.49, 0.30]	4.01
Li et al. 2022	50	34.6	12.3	49	35.3	9.2	-0.06 [-0.45, 0.33]	4.12
Spielmanns et al. 2022	30	4.4	3.5	30	5.3	4.2	-0.23 [-0.73, 0.27]	2.69
Heterogeneity: $\tau^2 = 0.00$, $I^2 = 0.00\%$, $H^2 = 1.00$							-0.11 [-0.25, 0.02]	
Test of $\theta_i = \theta_j$: Q(6) = 7.03, p = 0.32								
Test of $\theta = 0$: z = -1.70, p = 0.09								
**Osteoarthritis (knee or hip)**								
Bossen et al. 2013	85	3.5	4.7	79	4.2	4.8	-0.15 [-0.45, 0.16]	6.06
Bennell et al. 2018	66	2.2	2.8	65	2.1	2.7	0.04 [-0.30, 0.38]	5.14
O'Brien et al. 2018	37	6.4	6.6	52	7.0	8.2	-0.08 [-0.50, 0.34]	3.68
Tore et al. 2022	24	4.3	2.6	24	6.9	3.4	-0.86 [-1.44, -0.27]	2.06
Heterogeneity: $\tau^2 = 0.06$, $I^2 = 58.28\%$, $H^2 = 2.40$							-0.20 [-0.50, 0.11]	
Test of $\theta_i = \theta_j$: Q(3) = 6.92, p = 0.07								
Test of $\theta = 0$: z = -1.27, p = 0.20								
**Type 2 diabetes**								
Jiwani et al. 2020	11	51.7	11.7	6	48.1	8.5	0.32 [-0.63, 1.27]	0.82
Heterogeneity: $\tau^2 = 0.00$, $I^2 = .\%$, $H^2 = .$							0.32 [-0.63, 1.27]	
Test of $\theta_i = \theta_j$: Q(0) = -0.00, p = .								
Test of $\theta = 0$: z = 0.66, p = 0.51								
**Overall**							-0.17 [-0.26, -0.09]	
Heterogeneity: $\tau^2 = 0.01$, $I^2 = 18.08\%$, $H^2 = 1.22$								
Test of $\theta_i = \theta_j$: Q(24) = 36.92, p = 0.04								
Test of $\theta = 0$: z = -3.90, p = 0.00								
Test of group differences: $Q_b(4) = 2.79$, p = 0.59								

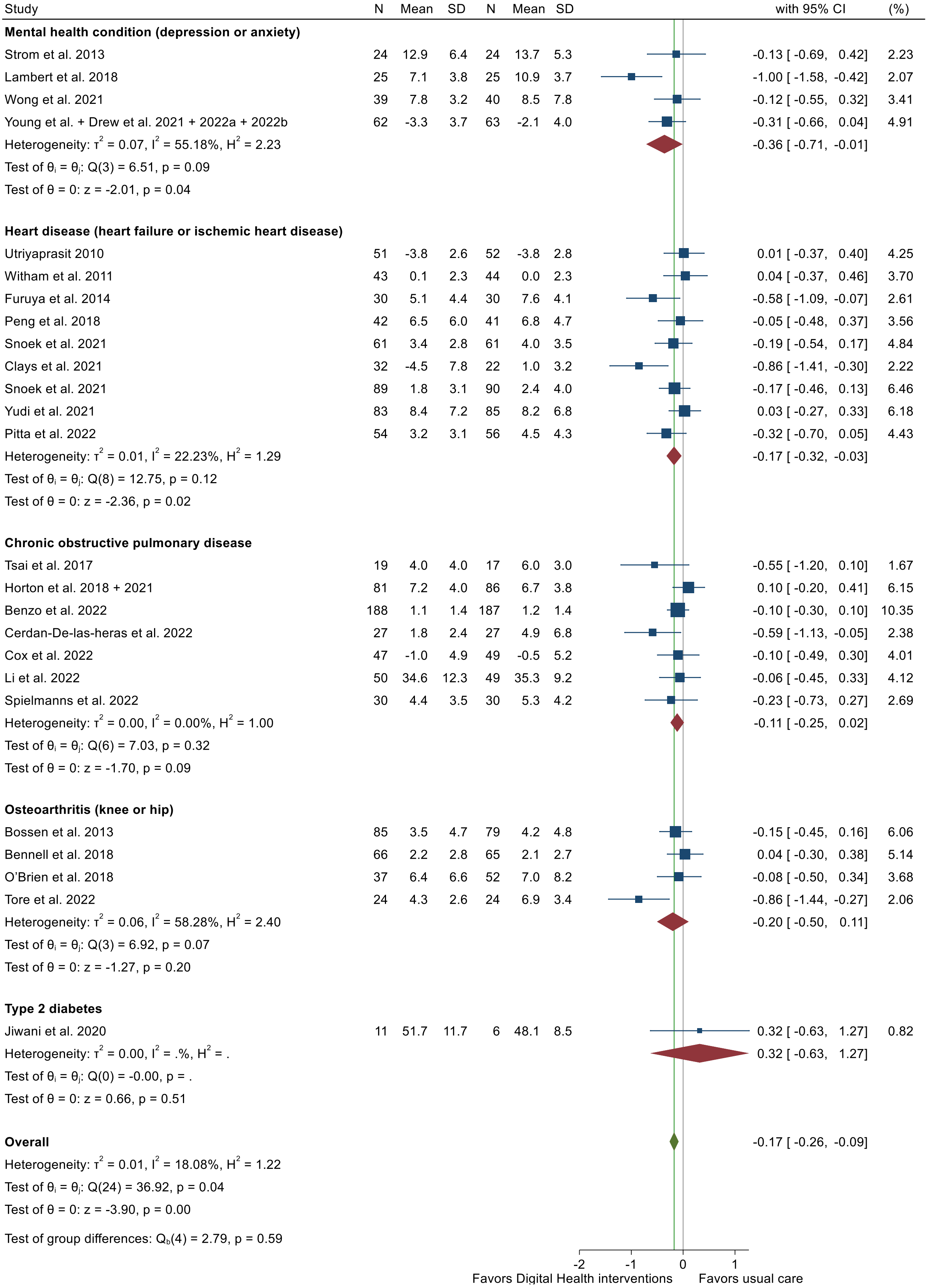

Supplementary Figure 10. Forest plot for health-related quality of life at end-of-intervention

Study	Treatment N	Treatment Mean	Treatment SD	Control N	Control Mean	Control SD	SMD with 95% CI	Weight (%)
**Mental health condition (depression or anxiety)**								
Strom et al. 2013	24	0.2	2.0	24	0.2	1.5	-0.04 [-0.60, 0.52]	1.19
Wong et al. 2021	39	0.7	0.1	40	0.7	0.1	0.00 [-0.44, 0.44]	1.36
Heterogeneity: $\tau^2 = 0.00$, $I^2 = 0.00\%$, $H^2 = 1.00$							-0.01 [-0.36, 0.33]	
Test of $\theta_i = \theta_j$: $Q(1) = 0.01$, $p = 0.91$								
Test of $\theta = 0$: $z = -0.09$, $p = 0.93$								
**Heart disease (heart failure or ischemic heart disease)**								
Hanssen et al. 2007	97	7.3	7.5	96	5.0	8.1	0.30 [0.02, 0.58]	1.57
Tomita et al. 2009	19	14.2	15.3	13	6.8	22.1	0.39 [-0.30, 1.09]	1.01
Witham et al. 2011	43	0.1	0.2	44	0.0	0.2	0.31 [-0.11, 0.73]	1.39
Reid et al. 2012	115	5.8	0.7	108	5.7	0.8	0.13 [-0.13, 0.39]	1.59
Hawkes et al. 2013	141	44.8	11.8	153	46.1	10.7	-0.12 [-0.34, 0.11]	1.63
Furuya et al. 2014	30	43.3	10.6	30	41.0	11.0	0.21 [-0.29, 0.71]	1.27
Frederix et al. 2015	69	2.5	0.5	70	2.3	0.6	0.37 [0.04, 0.71]	1.50
Widmer et al. 2017	37	7.2	1.3	34	2.4	1.6	3.27 [2.56, 3.98]	1.00
Duan et al. 2018	44	3.9	0.4	39	3.3	0.6	1.06 [0.61, 1.52]	1.33
Peng et al. 2018	42	-42.3	53.9	41	-49.6	79.3	0.11 [-0.32, 0.53]	1.38
Avila et al. 2018 + 2019	28	81.3	13.1	26	71.8	13.3	0.71 [0.17, 1.25]	1.21
Gingele et al. 2019	179	0.7	0.3	173	0.6	0.3	0.07 [-0.14, 0.28]	1.65
Piotrowicz et al. 2019	417	91.2	12.8	416	88.9	14.4	0.17 [0.03, 0.30]	1.71
Osteresch et al. + Wienbergen et al. 2019 + 2021	138	78.2	15.0	143	77.1	14.0	0.08 [-0.16, 0.31]	1.62
Turan Kavradim 2020	33	-16.3	16.3	31	-40.1	23.2	1.18 [0.65, 1.70]	1.24
Snoek et al. 2021	61	5.9	0.8	61	5.9	0.8	0.00 [-0.35, 0.35]	1.48
Andrade et al. 2021	11	28.0	33.0	12	22.0	13.0	0.23 [-0.56, 1.03]	0.90
Clays et al. 2021	34	-1.0	14.4	22	1.7	13.8	-0.19 [-0.72, 0.34]	1.23
Haejung et al. 2021	12	82.3	15.3	12	78.0	16.2	0.26 [-0.52, 1.04]	0.92
Radhakrishnan et al. 2021	19	8.2	13.5	19	13.0	19.3	-0.28 [-0.91, 0.34]	1.10
Reid et al. 2021	68	77.7	14.5	67	78.9	10.3	-0.09 [-0.43, 0.24]	1.50
Reid et al. 2021	158	79.9	12.1	156	80.2	11.7	-0.03 [-0.25, 0.20]	1.64
Snoek et al. 2021	89	50.2	7.2	90	48.3	7.5	0.26 [-0.04, 0.55]	1.55
Yudi et al. 2021	83	77.0	14.8	85	79.8	16.1	-0.18 [-0.48, 0.12]	1.54
Chan et al. 2022	70	15.4	1.9	69	15.2	2.0	0.10 [-0.23, 0.43]	1.51
Felker et al. 2022	83	73.4	20.0	82	66.9	23.0	0.30 [-0.01, 0.60]	1.54
Indraratna et al. 2022	76	78.2	15.5	74	75.7	14.5	0.17 [-0.15, 0.48]	1.52
Nagatomi et al. 2022	15	70.3	27.7	15	78.0	23.0	-0.29 [-0.99, 0.41]	1.01
Heterogeneity: $\tau^2 = 0.28$, $I^2 = 91.33\%$, $H^2 = 11.53$							0.28 [0.06, 0.49]	
Test of $\theta_i = \theta_j$: $Q(27) = 135.11$, $p = 0.00$								
Test of $\theta = 0$: $z = 2.55$, $p = 0.01$								
**Chronic obstructive pulmonary disease**								
de Sousa Pinto et al. 2014	23	-46.0	12.0	18	-65.6	10.6	1.68 [0.98, 2.39]	1.00
Vasilopoulou et al. 2014	47	-38.4	20.5	50	-50.2	17.7	0.61 [0.21, 1.02]	1.41
Moy et al. 2015	144	-42.4	17.6	77	-45.0	16.0	0.15 [-0.12, 0.43]	1.57
Cameron-Tucker et al. 2016	35	0.0	6.0	30	0.0	6.0	0.00 [-0.48, 0.48]	1.30
Tsai et al. 2017	19	99.0	16.0	17	90.0	18.0	0.52 [-0.13, 1.17]	1.07
Wan et al. 2017	57	-0.9	9.9	52	-0.7	12.7	-0.02 [-0.39, 0.35]	1.45
Wootton et al. 2018 + 2019	43	40.0	12.0	37	42.0	16.0	-0.14 [-0.58, 0.29]	1.36
Bentley et al. 2020	9	7.6	1.4	6	6.5	2.1	0.59 [-0.41, 1.58]	0.70
Jiménez-Reguera et al. 2020	17	0.5	0.2	19	0.5	0.2	0.00 [-0.64, 0.64]	1.08
Benzo et al. 2021	56	0.2	1.0	63	0.0	0.7	0.23 [-0.13, 0.59]	1.47
Galdiz et al. 2021	41	46.1	24.0	40	51.1	24.6	-0.20 [-0.64, 0.23]	1.37
Robinson et al. 2021	75	-13.1	31.1	78	-15.1	27.9	0.07 [-0.25, 0.39]	1.53
Wang et al. 2021	39	-18.3	3.5	39	-20.9	4.4	0.65 [0.20, 1.10]	1.34
Benzo et al. 2022	188	4.3	1.1	187	3.8	1.2	0.46 [0.26, 0.67]	1.65
Cerdan-De-las-heras et al. 2022	27	51.9	10.6	27	54.0	16.3	-0.15 [-0.68, 0.37]	1.24
Cox et al. 2022	47	2.3	13.6	49	0.2	6.4	0.20 [-0.20, 0.59]	1.42
Li et al. 2022	50	16.3	7.9	49	17.1	7.8	-0.10 [-0.49, 0.29]	1.43
Spielmanns et al. 2022	30	15.1	8.6	30	19.7	6.4	-0.60 [-1.11, -0.09]	1.26
Heterogeneity: $\tau^2 = 0.12$, $I^2 = 73.45\%$, $H^2 = 3.77$							0.19 [-0.01, 0.38]	
Test of $\theta_i = \theta_j$: $Q(17) = 54.73$, $p = 0.00$								
Test of $\theta = 0$: $z = 1.91$, $p = 0.06$								
**Osteoarthritis (knee or hip)**								
Bossen et al. 2013	85	49.4	36.0	80	47.3	35.8	0.06 [-0.25, 0.36]	1.54
Bennell et al. 2017	70	0.8	0.1	69	0.7	0.1	0.99 [0.64, 1.35]	1.48
Bennell et al. 2018	66	0.8	0.1	65	0.8	0.1	0.00 [-0.34, 0.34]	1.49
Li et al. 2018	30	47.2	18.9	31	42.4	16.8	0.27 [-0.23, 0.76]	1.28
O'Brien et al. 2018	37	29.4	9.4	49	33.4	8.9	-0.43 [-0.86, -0.01]	1.37
Bartholdy et al. 2019	19	-3.4	15.5	19	-5.0	15.5	0.10 [-0.52, 0.72]	1.10
Hinman et al. 2019	83	0.7	0.2	82	0.7	0.2	0.00 [-0.30, 0.30]	1.54
Bennell et al. 2020	56	0.8	0.1	54	0.8	0.1	-0.07 [-0.44, 0.31]	1.45
Doiron-Cadrin et al. 2020	12	35.6	6.2	11	36.2	6.8	-0.09 [-0.88, 0.70]	0.90
Li et al. 2020	24	48.7	17.5	24	46.9	13.6	0.11 [-0.44, 0.67]	1.19
Pelle et al. 2020	130	0.7	0.2	166	0.7	0.2	0.05 [-0.18, 0.27]	1.63
Gohir et al. 2021	48	1.2	11.1	57	1.6	10.3	-0.04 [-0.42, 0.34]	1.44
Nelligan et al. 2021	103	0.8	0.2	103	0.7	0.2	0.33 [0.06, 0.60]	1.58
Tore et al. 2022	24	69.4	18.9	24	45.5	24.4	1.08 [0.48, 1.68]	1.14
Heterogeneity: $\tau^2 = 0.11$, $I^2 = 74.78\%$, $H^2 = 3.97$							0.16 [-0.04, 0.37]	
Test of $\theta_i = \theta_j$: $Q(13) = 46.09$, $p = 0.00$								
Test of $\theta = 0$: $z = 1.55$, $p = 0.12$								
**Type 2 diabetes**								
Dyson et al. 2010	21	64.4	12.2	18	70.3	12.2	-0.47 [-1.10, 0.15]	1.10
Plotnikoff et al. 2013	74	0.8	0.0	83	0.8	0.0	-1.00 [-1.33, -0.66]	1.51
Plotnikoff et al. 2013	72	0.8	0.1	42	0.8	0.1	0.11 [-0.26, 0.49]	1.44
Holmen et al. 2014	38	49.5	11.0	20	49.0	10.6	0.05 [-0.49, 0.58]	1.22
Holmen et al. 2014	39	51.3	9.1	20	49.0	10.6	0.23 [-0.30, 0.77]	1.23
van der Weegen et al. 2015	65	45.2	9.5	68	47.0	10.0	-0.18 [-0.52, 0.16]	1.50
Akinci et al. 2018	21	0.8	0.1	22	0.6	0.3	0.84 [0.22, 1.45]	1.12
Vinitha et al. 2019	126	1.0	0.0	122	1.0	0.0	0.00 [-0.25, 0.25]	1.61
Jiwani et al. 2020	11	37.8	4.2	6	39.4	4.2	-0.36 [-1.31, 0.59]	0.74
Waller et al. 2021	197	42.0	22.4	198	43.0	23.1	-0.04 [-0.24, 0.15]	1.66
Heterogeneity: $\tau^2 = 0.17$, $I^2 = 82.39\%$, $H^2 = 5.68$							-0.09 [-0.38, 0.20]	
Test of $\theta_i = \theta_j$: $Q(9) = 42.92$, $p = 0.00$								
Test of $\theta = 0$: $z = -0.60$, $p = 0.55$								
**Multimorbidity**								
Piette et al. 2011	145	38.2	12.0	146	35.8	12.0	0.20 [-0.03, 0.43]	1.63
Bernocchi et al. 2018	48	10.5	12.7	44	0.4	14.5	0.73 [0.31, 1.15]	1.39
Heterogeneity: $\tau^2 = 0.11$, $I^2 = 79.18\%$, $H^2 = 4.80$							0.44 [-0.08, 0.96]	
Test of $\theta_i = \theta_j$: $Q(1) = 4.80$, $p = 0.03$								
Test of $\theta = 0$: $z = 1.64$, $p = 0.10$								
**Overall**							0.18 [0.07, 0.29]	
Heterogeneity: $\tau^2 = 0.17$, $I^2 = 84.38\%$, $H^2 = 6.40$								
Test of $\theta_i = \theta_j$: $Q(73) = 310.10$, $p = 0.00$								
Test of $\theta = 0$: $z = 3.29$, $p = 0.00$								
Test of group differences: $Q_b(5) = 6.11$, $p = 0.30$								

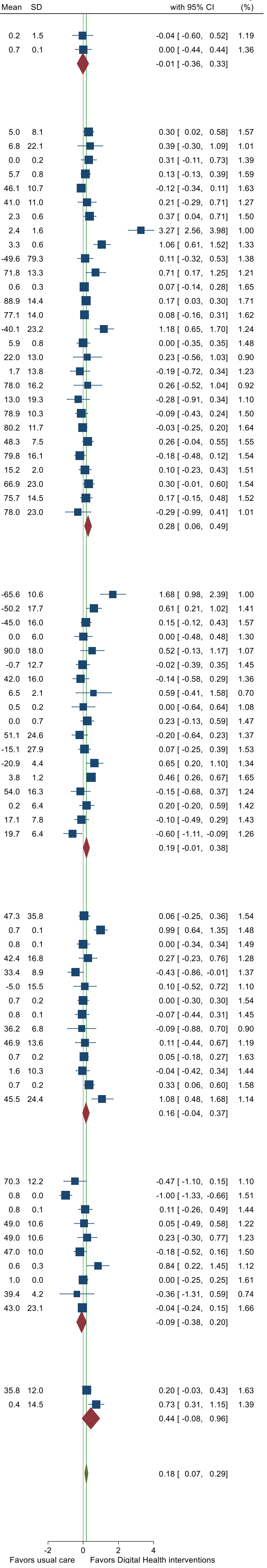

Supplementary Figure 11. Forest plot for adverse events, non-serious at end-of-intervention

Study	Treatment Yes	Treatment No	Control Yes	Control No	Risk ratio with 95% CI	Weight (%)
Eakin et al. 2009	5	223	1	205	4.52 [0.53, 38.35]	0.60
Moore et al. 2009	1	12	1	15	3.08 [0.14, 66.78]	0.30
Anderson et al. 2010	1	145	1	148	1.02 [0.06, 16.16]	0.37
Witham et al. 2011	20	33	15	39	1.36 [0.78, 2.36]	6.41
Bossen et al. 2013	1	101	0	99	1.00 [0.02, 49.91]	0.18
de Sousa Pinto et al. 2014	1	24	0	18	1.00 [0.02, 49.56]	0.19
Eakin et al. 2014	17	134	28	123	0.61 [0.35, 1.06]	6.30
Piotrowicz et al. 2015	1	76	2	33	0.23 [0.02, 2.42]	0.50
Coultas et al. 2016	33	116	23	133	1.50 [0.93, 2.43]	7.63
Chaplin et al. 2016 + 2022	10	41	2	50	5.10 [1.17, 22.13]	1.24
Bennell et al. 2017	22	43	3	64	7.56 [2.38, 24.04]	1.93
Chokshi et al. 2017	0	50	1	56	1.00 [0.02, 49.70]	0.19
Demeyer et al. 2017	54	117	50	122	1.09 [0.79, 1.50]	11.70
Tsai et al. 2017	1	21	0	17	1.00 [0.02, 48.53]	0.19
Wan et al. 2017	15	45	9	45	1.50 [0.72, 3.14]	4.14
Connelly et al. 2017	1	12	0	5	1.00 [0.02, 60.91]	0.17
Connelly et al. 2017	2	10	0	5	2.50 [0.07, 89.63]	0.22
Akinci et al. 2018	0	21	1	23	1.00 [0.02, 48.29]	0.19
Bennell et al. 2018	16	49	16	54	1.08 [0.59, 1.97]	5.63
Deka et al. 2018	1	16	1	16	1.00 [0.02, 47.38]	0.19
Li et al. 2018	0	30	1	32	1.00 [0.02, 48.87]	0.19
Allen et al. 2018	5	139	0	68	6.92 [0.20, 240.74]	0.22
Al-Ghafri et al. 2018	20	102	12	98	1.50 [0.77, 2.93]	4.86
O'Brien et al. 2018	40	20	40	20	1.00 [0.78, 1.29]	13.95
Benson et al. 2019	1	61	0	58	1.00 [0.02, 49.61]	0.19
Hinman et al. 2019	8	79	3	85	2.70 [0.74, 9.83]	1.57
Bennell et al. 2020	9	47	8	46	1.08 [0.45, 2.60]	3.14
Li et al. 2020	8	18	4	21	1.92 [0.66, 5.59]	2.23
Allen et al. 2021	2	230	0	115	2.50 [0.06, 102.35]	0.20
Benzo et al. 2021	1	71	2	72	0.51 [0.05, 5.54]	0.49
Coombes et al. 2021	2	12	1	15	2.29 [0.23, 22.59]	0.53
Gohir et al. 2021	1	66	1	78	1.18 [0.08, 18.49]	0.37
Hsu et al. 2021	1	23	1	23	1.00 [0.02, 48.28]	0.19
Jaarsma et al. 2021	18	287	12	288	1.48 [0.72, 3.01]	4.39
Lim et al. 2021	3	96	1	106	7.18 [0.39, 132.35]	0.33
Nelligan et al. 2021	13	90	5	98	2.60 [0.96, 7.03]	2.52
Robinson et al. 2021	32	43	23	55	1.45 [0.94, 2.23]	8.72
Snoek et al. 2021	1	60	1	60	1.00 [0.06, 15.63]	0.37
Waller et al. 2021	1	196	1	199	3.01 [0.12, 73.12]	0.28
Reid et al. 2021	3	65	2	65	1.48 [0.26, 8.57]	0.88
Reid et al. 2021	2	156	3	153	0.66 [0.11, 3.89]	0.87
Wong et al. 2021	0	39	1	41	1.00 [0.02, 49.20]	0.19
Benzo et al. 2022	1	189	0	187	1.00 [0.02, 50.14]	0.18
Chan et al. 2022	1	71	0	69	1.00 [0.02, 49.70]	0.19
Nagatomi et al. 2022	9	6	7	8	1.29 [0.65, 2.54]	4.72
**Overall**					1.31 [1.11, 1.55]	

Heterogeneity: $\tau^2 = 0.04$, $I^2 = 14.54\%$, $H^2 = 1.17$

Test of $\theta_i = \theta_j$: $Q(44) = 37.21$, $p = 0.76$

Test of $\theta = 0$: $z = 3.15$, $p = 0.00$

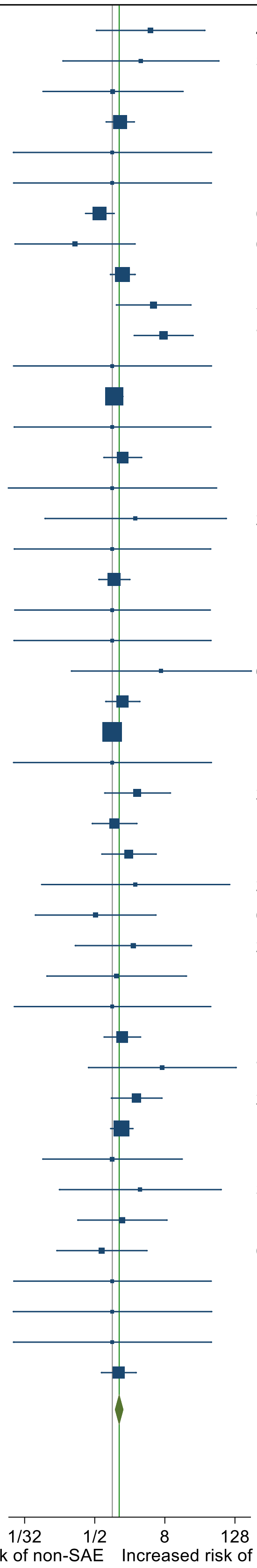

Supplementary Figure 12. Forest plot for adverse events, serious at end-of-intervention

Study	Treatment Yes	Treatment No	Control Yes	Control No	Risk ratio with 95% CI	Weight (%)
Southard et al. 2003	2	51	8	43	0.24 [0.05, 1.08]	1.04
Hanssen et al. 2007	8	148	8	124	0.85 [0.33, 2.19]	2.29
Eakin et al. 2009	4	226	0	206	6.71 [0.33, 138.13]	0.27
Tomita et al. 2009	3	21	1	15	2.00 [0.23, 17.57]	0.52
Anderson et al. 2010	2	144	1	148	2.04 [0.19, 22.27]	0.43
De Greef et al. 2011	1	61	2	30	0.15 [0.01, 2.23]	0.34
Witham et al. 2011	16	37	12	42	1.36 [0.71, 2.59]	4.10
Reid et al. 2012	4	111	9	99	0.42 [0.13, 1.32]	1.67
Bossen et al. 2013	1	101	0	99	1.00 [0.02, 49.91]	0.16
Hawkes et al. 2013	5	212	1	216	9.00 [0.49, 166.15]	0.29
Eakin et al. 2014	4	147	4	147	1.00 [0.25, 3.93]	1.23
Holmen et al. 2014	2	49	1	24	0.98 [0.09, 10.30]	0.44
Holmen et al. 2014	3	47	1	24	1.50 [0.16, 13.70]	0.50
Chow et al. 2015	4	348	1	357	4.07 [0.46, 36.22]	0.51
Frederix et al. 2015	1	68	1	71	3.01 [0.13, 72.19]	0.25
Hornikx et al. 2015	5	10	6	9	0.83 [0.32, 2.15]	2.31
Moy et al. 2015	3	154	0	84	4.08 [0.12, 136.41]	0.20
Piotrowicz et al. 2015	1	78	0	34	1.00 [0.01, 68.93]	0.14
Agboola et al. 2016	1	63	1	61	0.97 [0.06, 15.15]	0.33
Coultas et al. 2016	66	83	72	84	0.96 [0.75, 1.23]	9.53
Chaplin et al. 2016 + 2022	0	51	1	53	1.00 [0.02, 49.47]	0.17
Chokshi et al. 2017	6	34	9	28	0.62 [0.24, 1.56]	2.38
Tsai et al. 2017	2	20	0	17	2.85 [0.11, 72.33]	0.24
Wan et al. 2017	9	51	8	46	1.01 [0.42, 2.44]	2.61
Widmer et al. 2017	4	36	7	33	0.57 [0.18, 1.80]	1.68
Akinci et al. 2018	0	21	1	23	1.00 [0.02, 48.29]	0.17
Bernocchi et al. 2018	7	49	8	48	0.88 [0.34, 2.25]	2.32
Dobler et al. 2018	2	96	1	102	5.06 [0.25, 102.23]	0.28
Li et al. 2018	0	30	1	32	1.00 [0.02, 48.87]	0.17
Peng et al. 2018	1	50	1	50	1.00 [0.02, 49.42]	0.17
O'Brien et al. 2018	11	49	7	53	1.57 [0.65, 3.78]	2.61
Avila et al. 2018 + 2019	3	25	0	25	4.92 [0.24, 100.16]	0.27
Horton et al. 2018 + 2021	18	127	13	129	1.36 [0.69, 2.66]	3.86
Benson et al. 2019	1	61	0	58	1.00 [0.02, 49.61]	0.17
Gingele et al. 2019	18	179	12	173	1.41 [0.70, 2.84]	3.65
Piotrowicz et al. 2019	2	423	2	423	1.00 [0.14, 7.07]	0.63
Osteresch et al. + Wienbergen et al. 2019 + 2021	21	117	29	114	0.75 [0.45, 1.25]	5.46
Bennell et al. 2020	1	57	0	54	1.00 [0.02, 49.55]	0.17
Li et al. 2020	1	27	1	24	0.33 [0.01, 7.56]	0.25
Galdiz et al. 2021	26	20	21	27	1.29 [0.86, 1.94]	6.83
Gohir et al. 2021	0	67	1	80	1.00 [0.02, 50.40]	0.16
Jaarsma et al. 2021	1	304	2	298	0.49 [0.04, 5.40]	0.43
Lim et al. 2021	0	99	2	105	0.34 [0.01, 8.52]	0.24
Nelligan et al. 2021	1	104	1	104	1.00 [0.02, 49.92]	0.16
Robinson et al. 2021	8	67	12	66	0.69 [0.30, 1.60]	2.82
Snoek et al. 2021	13	48	15	46	0.87 [0.45, 1.66]	4.04
Waller et al. 2021	1	196	1	197	1.01 [0.06, 15.96]	0.33
Wang et al. 2021	1	38	2	37	0.50 [0.05, 5.29]	0.44
Andrade et al. 2021	2	12	1	13	2.00 [0.20, 19.62]	0.47
Clays et al. 2021	3	37	0	23	4.21 [0.14, 123.10]	0.22
Reid et al. 2021	2	68	0	67	2.99 [0.12, 72.58]	0.25
Reid et al. 2021	2	158	0	156	2.99 [0.12, 73.28]	0.24
Yudi et al. 2021	32	51	38	47	0.86 [0.60, 1.24]	7.59
Benzo et al. 2022	3	185	6	181	0.50 [0.13, 1.96]	1.22
Chan et al. 2022	1	71	0	69	1.00 [0.02, 49.70]	0.17
Indraratna et al. 2022	22	59	45	38	0.50 [0.33, 0.75]	6.85
Li et al. 2022	20	31	39	10	0.49 [0.34, 0.71]	7.37
Michelsen et al. 2022	23	77	14	35	0.81 [0.46, 1.42]	4.81
Nagatomi et al. 2022	3	12	3	12	1.00 [0.24, 4.18]	1.13
Pitta et al. 2022	1	53	1	57	3.04 [0.13, 71.58]	0.25
Tore et al. 2022	1	25	1	25	1.00 [0.02, 48.45]	0.17
**Overall**					0.89 [0.76, 1.04]	

Heterogeneity: $\tau^2 = 0.05$, $I^2 = 19.13\%$, $H^2 = 1.24$

Test of $\theta_i = \theta_j$: Q(60) = 52.26, p = 0.75

Test of $\theta = 0$: z = -1.48, p = 0.14

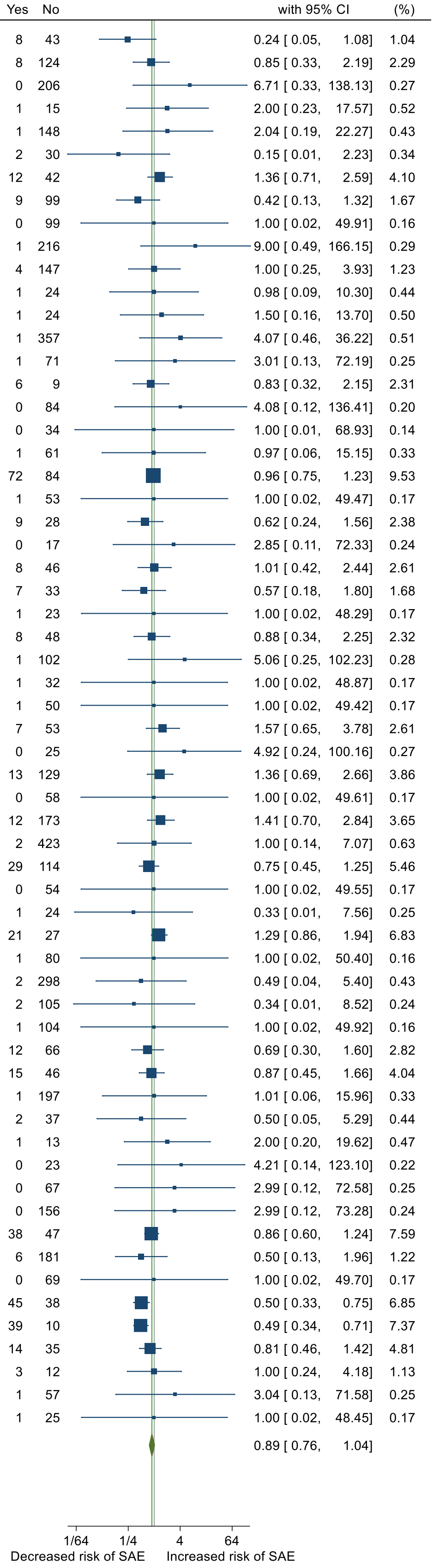

Supplementary Figure 13. Forest plot for objectively measured physical activity at follow-up

	Treatment			Control				
Study	N	Mean	SD	N	Mean	SD	SMD with 95% CI	Weight (%)
**Mental health condition (depression or anxiety)**								
Young et al. + Drew et al. 2021 + 2022a + 2022b	62	0.5	2.2	63	0.5	2.2	0.00 [-0.35, 0.35]	6.74
Heterogeneity: τ^2 = 0.00, I^2 = .%, H^2 = .							0.00 [-0.35, 0.35]	
Test of $\theta_i = \theta_j$: Q(0) = 0.00, p = .								
Test of θ = 0: z = 0.00, p = 1.00								
**Heart disease (heart failure or ischemic heart disease)**								
Reid et al. 2012	115	7392.0	3365.0	108	6750.0	3366.0	0.19 [-0.07, 0.45]	8.42
Chokshi et al. 2017	48	8271.0	3003.0	55	6669.0	3091.0	0.52 [0.13, 0.91]	6.02
Avila et al. 2019	21	8002.0	7998.0	26	6948.0	5751.0	0.15 [-0.41, 0.72]	3.83
Felker et al. 2022	63	3679.4	2926.3	64	2755.5	1872.3	0.37 [0.03, 0.72]	6.73
Heterogeneity: τ^2 = 0.00, I^2 = 0.00%, H^2 = 1.00							0.30 [0.12, 0.48]	
Test of $\theta_i = \theta_j$: Q(3) = 2.35, p = 0.50								
Test of θ = 0: z = 3.35, p = 0.00								
**Chronic obstructive pulmonary disease**								
Moy et al. 2016	154	270.0	2236.2	84	163.0	2297.1	0.05 [-0.22, 0.31]	8.36
Wootton et al. 2018 + 2019	23	-757.0	1392.8	20	0.0	1392.8	-0.53 [-1.13, 0.07]	3.54
Horton et al. 2018 + 2021	26	-309.0	1783.8	25	-275.0	1876.3	-0.02 [-0.56, 0.52]	4.08
Wan et al. 2020	35	-104.0	225.5	40	0.0	225.5	-0.46 [-0.91, -0.00]	5.08
Cerdan-De-las-heras et al. 2022	27	7660.8	3986.3	27	9384.0	7068.4	-0.30 [-0.82, 0.23]	4.21
Cox et al. 2022	47	-2.0	37.5	49	-6.0	38.3	0.10 [-0.29, 0.50]	5.92
Heterogeneity: τ^2 = 0.02, I^2 = 32.57%, H^2 = 1.48							-0.13 [-0.35, 0.08]	
Test of $\theta_i = \theta_j$: Q(5) = 7.19, p = 0.21								
Test of θ = 0: z = -1.22, p = 0.22								
**Osteoarthritis (knee or hip)**								
Bossen et al. 2013	24	361.0	123.7	28	338.0	130.9	0.18 [-0.36, 0.72]	4.11
Heterogeneity: τ^2 = 0.00, I^2 = .%, H^2 = .							0.18 [-0.36, 0.72]	
Test of $\theta_i = \theta_j$: Q(0) = 0.00, p = .								
Test of θ = 0: z = 0.65, p = 0.52								
**Type 2 diabetes**								
De Greef et al. 2011	60	311.0	88.0	32	273.0	107.0	0.40 [-0.03, 0.83]	5.44
Eakin et al. 2014	151	1.4	3.2	151	0.0	3.2	0.45 [0.22, 0.67]	9.16
van der Weegen et al. 2015	65	48.8	23.8	68	42.4	18.9	0.30 [-0.04, 0.64]	6.89
Bender et al. 2017	22	9524.0	3626.0	23	7208.0	2719.0	0.71 [0.12, 1.31]	3.59
Alonso-Dominguez et al. 2019	94	10253.0	4781.0	91	8717.0	5353.0	0.30 [0.01, 0.59]	7.88
Heterogeneity: τ^2 = 0.00, I^2 = 0.00%, H^2 = 1.00							0.39 [0.25, 0.54]	
Test of $\theta_i = \theta_j$: Q(4) = 2.01, p = 0.73								
Test of θ = 0: z = 5.35, p = 0.00								
**Overall**							0.17 [0.04, 0.31]	
Heterogeneity: τ^2 = 0.04, I^2 = 50.85%, H^2 = 2.03								
Test of $\theta_i = \theta_j$: Q(16) = 33.07, p = 0.01								
Test of θ = 0: z = 2.55, p = 0.01								
Test of group differences: $Q_b(4)$ = 18.09, p = 0.00								

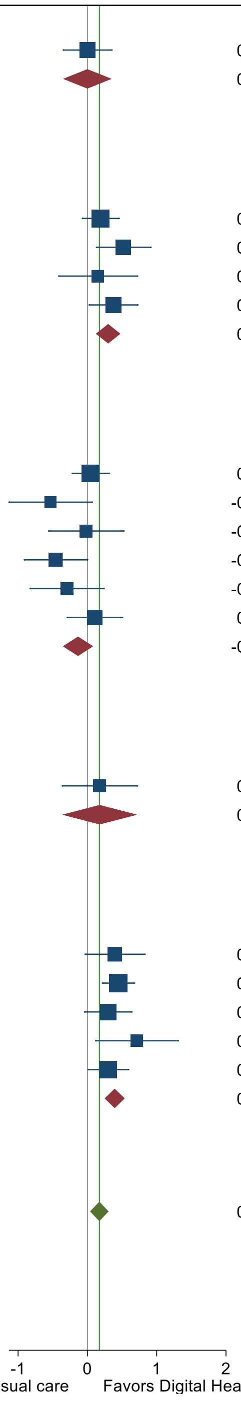

Supplementary Figure 14. Forest plot for objectively measured physical function at follow-up

Study	Treatment N	Treatment Mean	Treatment SD	Control N	Control Mean	Control SD	SMD with 95% CI	Weight (%)
**Heart disease (heart failure or ischemic heart disease)**								
Avila et al. 2019	16	7.9	1.3	18	7.3	1.3	0.47 [-0.19, 1.14]	8.48
Jaarsma et al. 2021	207	456.0	122.0	216	420.0	135.0	0.28 [0.09, 0.47]	15.23
Haejung et al. 2021	7	405.9	58.3	5	414.3	76.6	-0.12 [-1.18, 0.94]	4.88
Heterogeneity: τ^2 = 0.00, I^2 = 0.00%, H^2 = 1.00							0.28 [0.10, 0.46]	
Test of $\theta_i = \theta_j$: Q(2) = 0.86, p = 0.65								
Test of θ = 0: z = 3.05, p = 0.00								
**Chronic obstructive pulmonary disease**								
Wootton et al. 2018 + 2019	35	469.0	98.0	27	432.0	121.0	0.34 [-0.16, 0.84]	10.77
Horton et al. 2018 + 2021	59	248.1	151.9	66	270.0	150.9	-0.14 [-0.49, 0.21]	13.05
Cerdan-De-las-heras et al. 2022	27	415.6	107.9	27	320.4	106.8	0.87 [0.32, 1.42]	10.02
Cox et al. 2022	47	18.0	76.6	49	-2.0	102.7	0.22 [-0.18, 0.62]	12.30
Heterogeneity: τ^2 = 0.12, I^2 = 69.98%, H^2 = 3.33							0.28 [-0.12, 0.69]	
Test of $\theta_i = \theta_j$: Q(3) = 9.76, p = 0.02								
Test of θ = 0: z = 1.39, p = 0.17								
**Osteoarthritis (knee or hip)**								
Allen et al. 2018	142	-1.5	5.9	68	-0.3	7.8	-0.19 [-0.47, 0.10]	13.96
Heterogeneity: τ^2 = 0.00, I^2 = .%, H^2 = .							-0.19 [-0.47, 0.10]	
Test of $\theta_i = \theta_j$: Q(0) = 0.00, p = .								
Test of θ = 0: z = -1.26, p = 0.21								
**Multimorbidity**								
Bernocchi et al. 2018	45	420.0	118.0	35	300.0	125.0	0.98 [0.52, 1.44]	11.31
Heterogeneity: τ^2 = 0.00, I^2 = .%, H^2 = .							0.98 [0.52, 1.44]	
Test of $\theta_i = \theta_j$: Q(0) = 0.00, p = .								
Test of θ = 0: z = 4.15, p = 0.00								
**Overall**							0.29 [0.01, 0.57]	
Heterogeneity: τ^2 = 0.12, I^2 = 76.17%, H^2 = 4.20								
Test of $\theta_i = \theta_j$: Q(8) = 29.08, p = 0.00								
Test of θ = 0: z = 2.06, p = 0.04								
Test of group differences: $Q_b(3)$ = 18.47, p = 0.00								

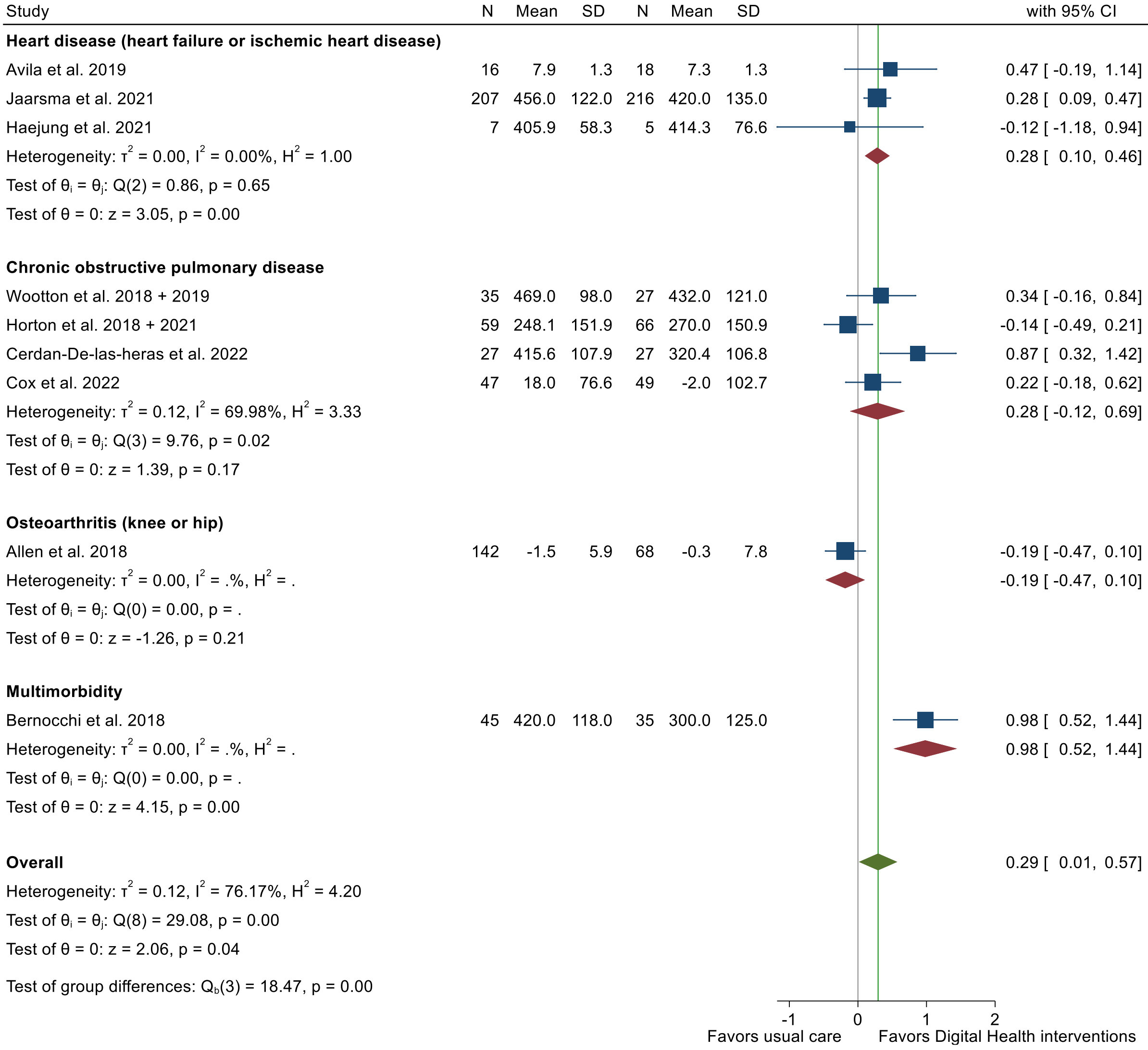

Supplementary Figure 15. Forest plot for objectively measured moderate-to-vigorous physical activity at follow-up

Study	Treatment N	Treatment Mean	Treatment SD	Control N	Control Mean	Control SD	SMD with 95% CI	Weight (%)
**Mental health condition (depression or anxiety)**								
Young et al. + Drew et al. 2021 + 2022a + 2022b	62	0.5	2.2	63	0.5	2.2	0.00 [-0.35, 0.35]	17.32
Heterogeneity: τ^2 = 0.00, I^2 = .%, H^2 = .							0.00 [-0.35, 0.35]	
Test of $\theta_i = \theta_j$: Q(0) = 0.00, p = .								
Test of θ = 0: z = 0.00, p = 1.00								
**Chronic obstructive pulmonary disease**								
Horton et al. 2018 + 2021	26	-5.0	53.2	25	-5.0	42.4	0.00 [-0.54, 0.54]	8.74
Cox et al. 2022	47	-2.0	37.5	49	-6.0	38.3	0.10 [-0.29, 0.50]	14.32
Heterogeneity: τ^2 = 0.00, I^2 = 0.00%, H^2 = 1.00							0.07 [-0.25, 0.39]	
Test of $\theta_i = \theta_j$: Q(1) = 0.09, p = 0.76								
Test of θ = 0: z = 0.42, p = 0.68								
**Type 2 diabetes**								
De Greef et al. 2011	60	19.0	19.0	32	14.0	20.0	0.26 [-0.17, 0.68]	12.82
Eakin et al. 2014	151	1.4	3.2	151	0.0	3.2	0.45 [0.22, 0.67]	28.87
van der Weegen et al. 2015	65	48.8	23.8	68	42.4	18.9	0.30 [-0.04, 0.64]	17.93
Heterogeneity: τ^2 = 0.00, I^2 = 0.00%, H^2 = 1.00							0.38 [0.20, 0.55]	
Test of $\theta_i = \theta_j$: Q(2) = 0.86, p = 0.65								
Test of θ = 0: z = 4.26, p = 0.00								
**Overall**							0.23 [0.06, 0.40]	
Heterogeneity: τ^2 = 0.01, I^2 = 29.45%, H^2 = 1.42								
Test of $\theta_i = \theta_j$: Q(5) = 6.21, p = 0.29								
Test of θ = 0: z = 2.60, p = 0.01								
Test of group differences: $Q_b(2)$ = 5.25, p = 0.07								

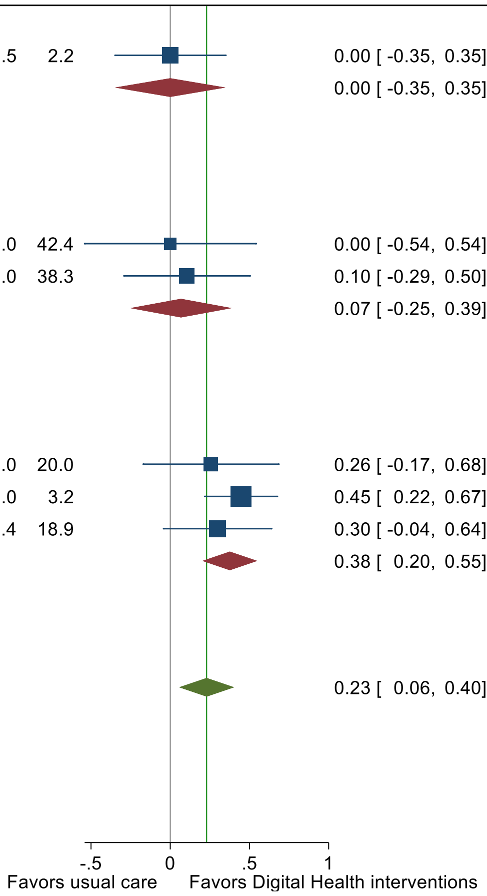

Supplementary Figure 16. Forest plot for daily steps at follow-up

Study	Treatment N	Treatment Mean	Treatment SD	Control N	Control Mean	Control SD	MD with 95% CI	Weight (%)
**Heart disease (heart failure or ischemic heart disease)**								
Reid et al. 2012	115	7392.0	3365.0	108	6750.0	3366.0	642.00 [-241.87, 1525.87]	11.50
Chokshi et al. 2017	48	8271.0	3003.0	55	6669.0	3091.0	1602.00 [421.09, 2782.91]	9.28
Avila et al. 2019	21	8002.0	7998.0	26	6948.0	5751.0	1054.00 [-2880.10, 4988.10]	1.71
Felker et al. 2022	63	3679.4	2926.3	64	2755.5	1872.3	923.90 [70.85, 1776.95]	11.74
Heterogeneity: $\tau^2 = 0.00$, $I^2 = 0.00\%$, $H^2 = 1.00$							962.85 [423.36, 1502.33]	
Test of $\theta_i = \theta_j$: Q(3) = 1.64, p = 0.65								
Test of $\theta = 0$: z = 3.50, p = 0.00								
**Chronic obstructive pulmonary disease**								
Moy et al. 2016	154	270.0	2236.2	84	163.0	2297.1	107.00 [-493.24, 707.24]	13.75
Wootton et al. 2018 + 2019	23	-757.0	1392.8	20	0.0	1392.8	-757.00 [-1591.63, 77.63]	11.89
Horton et al. 2018 + 2021	26	-309.0	1783.8	25	-275.0	1876.3	-34.00 [-1038.52, 970.52]	10.56
Wan et al. 2020	35	-104.0	225.5	40	0.0	225.5	-104.00 [-206.28, -1.72]	16.43
Heterogeneity: $\tau^2 = 0.00$, $I^2 = 0.00\%$, $H^2 = 1.00$							-106.80 [-206.41, -7.20]	
Test of $\theta_i = \theta_j$: Q(3) = 2.84, p = 0.42								
Test of $\theta = 0$: z = -2.10, p = 0.04								
**Type 2 diabetes**								
Bender et al. 2017	22	9524.0	3626.0	23	7208.0	2719.0	2316.00 [448.96, 4183.04]	5.60
Alonso-Dominguez et al. 2019	94	10253.0	4781.0	91	8717.0	5353.0	1536.00 [74.53, 2997.47]	7.53
Heterogeneity: $\tau^2 = 0.00$, $I^2 = 0.00\%$, $H^2 = 1.00$							1832.35 [681.53, 2983.17]	
Test of $\theta_i = \theta_j$: Q(1) = 0.42, p = 0.52								
Test of $\theta = 0$: z = 3.12, p = 0.00								
**Overall**							498.41 [-44.99, 1041.80]	
Heterogeneity: $\tau^2 = 465106.32$, $I^2 = 77.94\%$, $H^2 = 4.53$								
Test of $\theta_i = \theta_j$: Q(9) = 29.94, p = 0.00								
Test of $\theta = 0$: z = 1.80, p = 0.07								
Test of group differences: $Q_b(2) = 25.04$, p = 0.00								

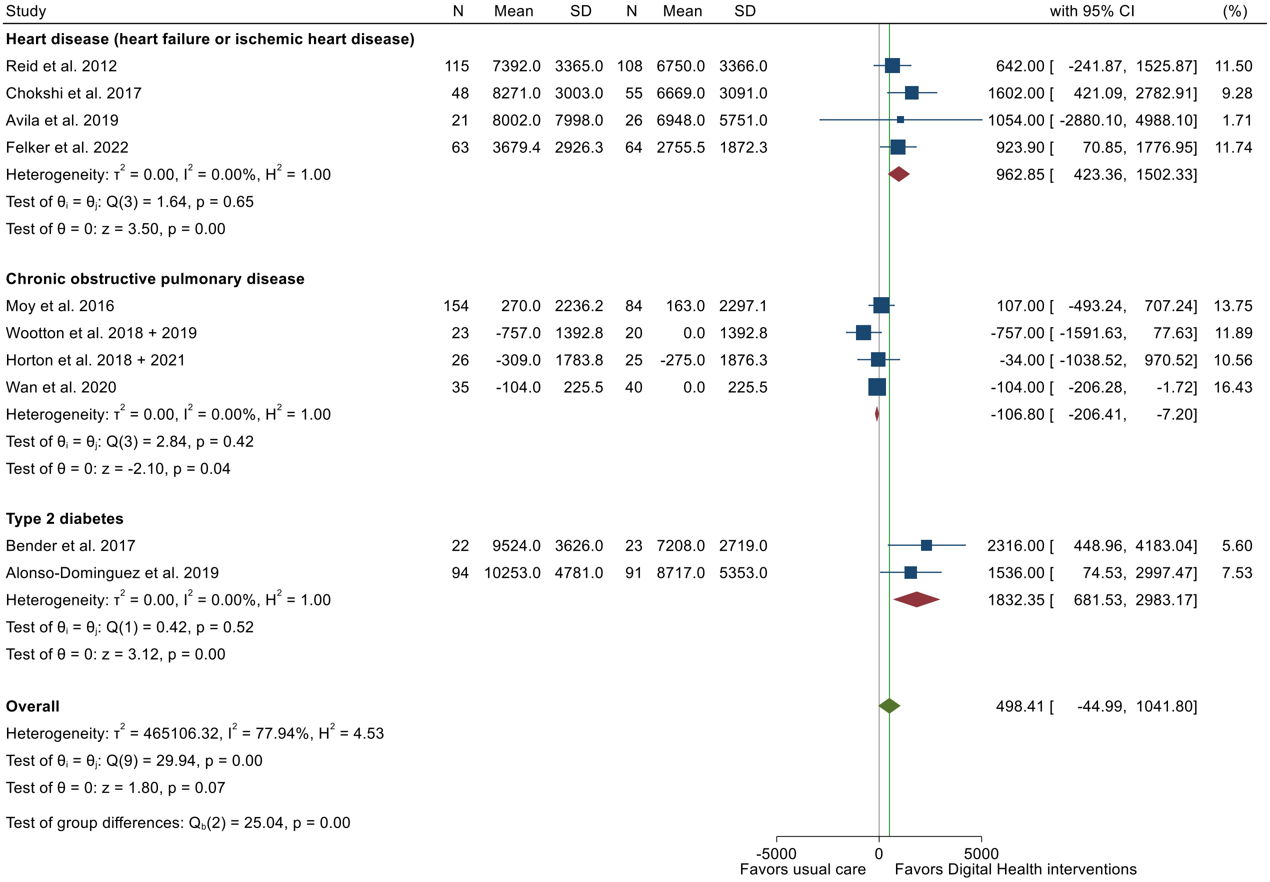

Supplementary Figure 17. Forest plot for six-minute walk test at follow-up

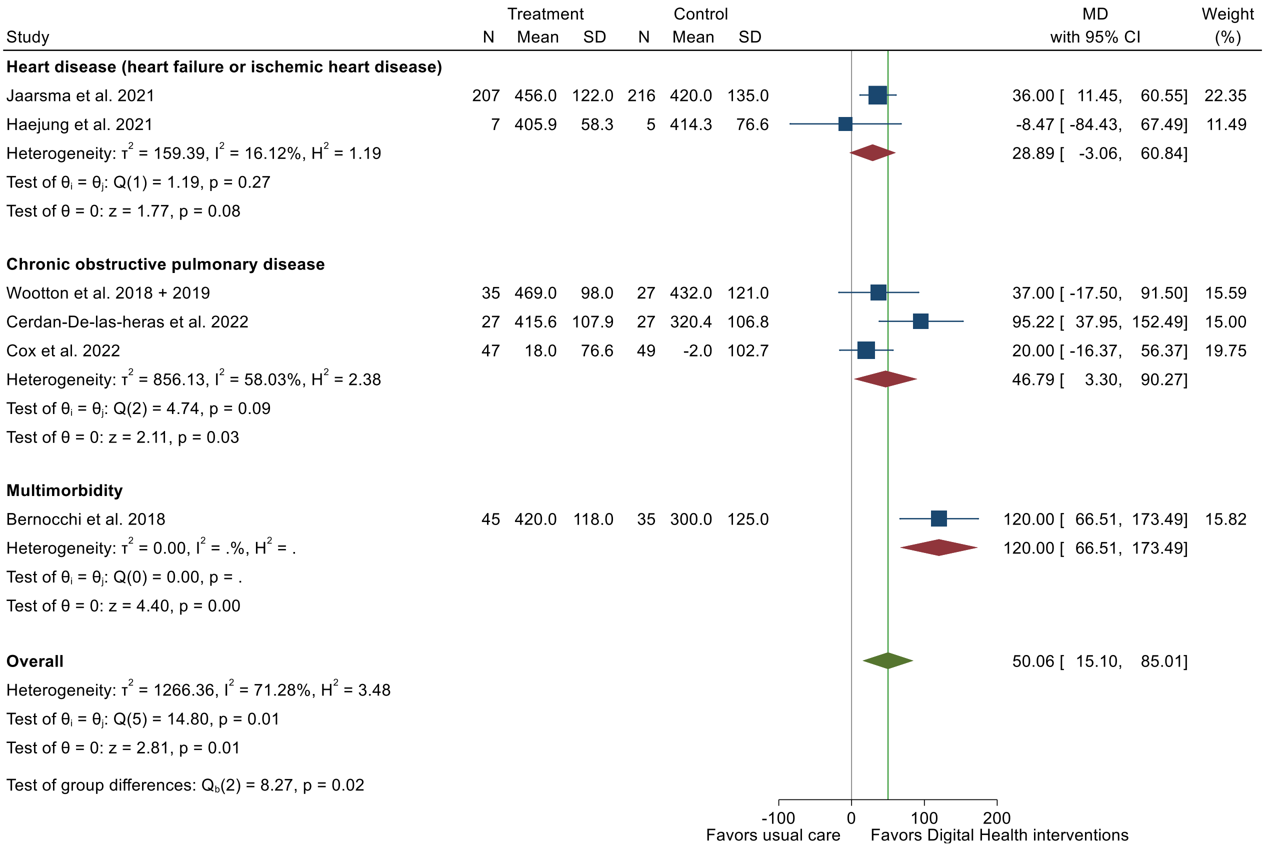

Supplementary Figure 18. Forest plot for subjectively measured physical activity at follow-up

Study	Treatment N	Treatment Mean	Treatment SD	Control N	Control Mean	Control SD	SMD with 95% CI	Weight (%)
**Heart disease (heart failure or ischemic heart disease)**								
Jaarsma et al. 2021	204	0.1	4.1	201	0.0	4.1	0.02 [-0.18, 0.21]	9.04
Haejung et al. 2021	16	236.6	303.1	15	178.7	451.7	0.15 [-0.54, 0.83]	6.39
Osteresch et al. 2021	134	1590.0	1803.7	136	590.0	1074.1	0.67 [0.43, 0.92]	8.85
Snoek et al. 2021	89	5.8	1.7	90	5.2	2.5	0.28 [-0.01, 0.57]	8.64
Heterogeneity: τ^2 = 0.08, I^2 = 78.73%, H^2 = 4.70							0.29 [-0.03, 0.62]	
Test of $\theta_i = \theta_j$: Q(3) = 17.08, p = 0.00								
Test of θ = 0: z = 1.80, p = 0.07								
**Osteoarthritis (knee or hip)**								
Bennell et al. 2018	65	194.0	93.0	62	190.0	82.0	0.05 [-0.30, 0.39]	8.38
Allen et al. 2018	142	8.2	56.3	68	1.2	98.2	0.10 [-0.19, 0.38]	8.66
Hinman et al. 2019	82	193.0	115.0	76	152.0	87.0	0.40 [0.08, 0.71]	8.54
Heterogeneity: τ^2 = 0.01, I^2 = 27.23%, H^2 = 1.37							0.18 [-0.03, 0.40]	
Test of $\theta_i = \theta_j$: Q(2) = 2.76, p = 0.25								
Test of θ = 0: z = 1.68, p = 0.09								
**Hypertension**								
Eakin et al. 2010	225	62.2	213.0	204	74.7	213.0	-0.06 [-0.25, 0.13]	9.05
Bozorgi et al. 2021	58	247.3	96.3	60	102.7	53.3	1.85 [1.42, 2.28]	7.93
Heterogeneity: τ^2 = 1.80, I^2 = 98.43%, H^2 = 63.81							0.89 [-0.99, 2.76]	
Test of $\theta_i = \theta_j$: Q(1) = 63.81, p = 0.00								
Test of θ = 0: z = 0.93, p = 0.35								
**Type 2 diabetes**								
Varney et al. 2014	35	115.0	238.7	36	119.0	239.4	-0.02 [-0.48, 0.44]	7.75
Jennings et al. 2019	195	745.5	2481.4	202	720.9	2393.4	0.01 [-0.19, 0.21]	9.03
Heterogeneity: τ^2 = 0.00, I^2 = 0.00%, H^2 = 1.00							0.01 [-0.17, 0.19]	
Test of $\theta_i = \theta_j$: Q(1) = 0.01, p = 0.92								
Test of θ = 0: z = 0.06, p = 0.95								
**Multimorbidity**								
Bernocchi et al. 2018	45	6.7	53.0	35	-39.8	42.8	0.94 [0.48, 1.40]	7.74
Heterogeneity: τ^2 = 0.00, I^2 = .%, H^2 = .							0.94 [0.48, 1.40]	
Test of $\theta_i = \theta_j$: Q(0) = -0.00, p = .								
Test of θ = 0: z = 4.01, p = 0.00								
**Overall**							0.36 [0.05, 0.66]	
Heterogeneity: τ^2 = 0.26, I^2 = 92.63%, H^2 = 13.57								
Test of $\theta_i = \theta_j$: Q(11) = 99.29, p = 0.00								
Test of θ = 0: z = 2.26, p = 0.02								
Test of group differences: $Q_b(4)$ = 15.11, p = 0.00								

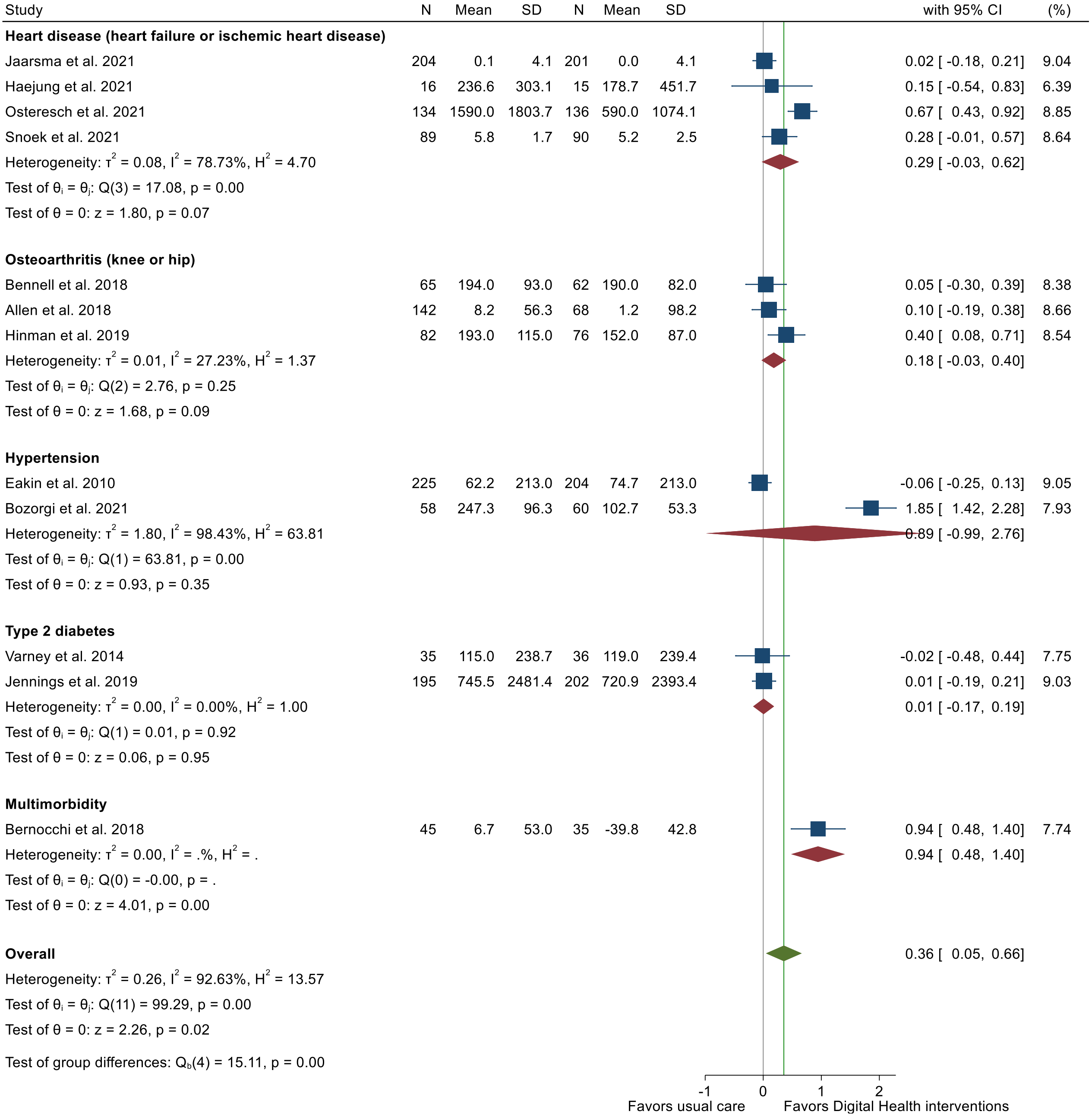

Supplementary Figure 19. Forest plot for subjectively measured physical function at follow-up

Study	Treatment N	Treatment Mean	Treatment SD	Control N	Control Mean	Control SD	SMD with 95% CI	Weight (%)
**Heart disease (heart failure or ischemic heart disease)**								
Reid et al. 2012	115	6.0	0.8	108	5.8	1.0	0.22 [-0.04, 0.48]	17.17
Radhakrishnan et al. 2021	19	7.4	10.7	19	6.5	11.7	0.08 [-0.54, 0.70]	7.28
Heterogeneity: τ^2 = 0.00, I^2 = 0.00%, H^2 = 1.00							0.20 [-0.04, 0.44]	
Test of $\theta_i = \theta_j$: Q(1) = 0.17, p = 0.68								
Test of θ = 0: z = 1.62, p = 0.11								
**Chronic obstructive pulmonary disease**								
Moy et al. 2016	154	-1.2	15.7	84	-1.1	15.8	-0.01 [-0.27, 0.26]	17.08
Heterogeneity: τ^2 = 0.00, I^2 = .%, H^2 = .							-0.01 [-0.27, 0.26]	
Test of $\theta_i = \theta_j$: Q(0) = -0.00, p = .								
Test of θ = 0: z = -0.05, p = 0.96								
**Osteoarthritis (knee or hip)**								
Bossen et al. 2013	75	67.9	38.9	72	62.9	28.0	0.15 [-0.18, 0.47]	14.95
Bennell et al. 2017	66	-18.7	10.2	67	-25.7	11.6	0.64 [0.29, 0.98]	14.10
Bennell et al. 2018	65	-18.7	12.6	62	-15.3	13.5	-0.26 [-0.61, 0.09]	14.07
Hinman et al. 2019	82	-18.1	11.4	76	-20.1	12.5	0.17 [-0.14, 0.48]	15.35
Heterogeneity: τ^2 = 0.10, I^2 = 77.83%, H^2 = 4.51							0.17 [-0.18, 0.52]	
Test of $\theta_i = \theta_j$: Q(3) = 12.86, p = 0.00								
Test of θ = 0: z = 0.96, p = 0.34								
**Overall**							0.14 [-0.06, 0.34]	
Heterogeneity: τ^2 = 0.04, I^2 = 61.02%, H^2 = 2.57								
Test of $\theta_i = \theta_j$: Q(6) = 14.57, p = 0.02								
Test of θ = 0: z = 1.40, p = 0.16								
Test of group differences: $Q_b(2)$ = 1.37, p = 0.50								

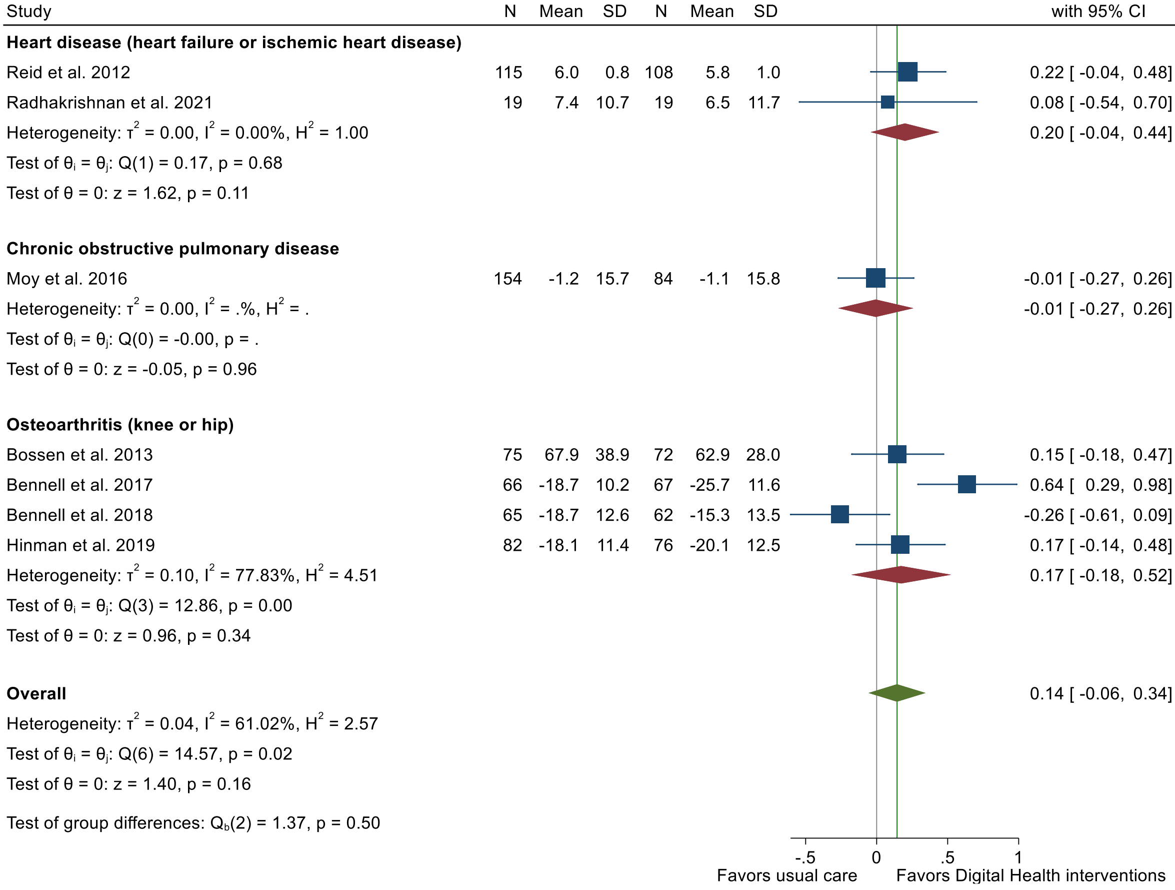

Supplementary Figure 20. Forest plot for depression at follow-up

Study	Treatment N	Treatment Mean	Treatment SD	Control N	Control Mean	Control SD	SMD with 95% CI	Weight (%)
**Mental health condition (depression or anxiety)**								
Young et al. + Drew et al. 2021 + 2022a + 2022b	62	-4.7	4.5	63	-2.3	4.8	-0.51 [-0.87, -0.16]	14.12
Heterogeneity: $\tau^2 = 0.00$, I^2 = .%, H^2 = .							-0.51 [-0.87, -0.16]	
Test of $\theta_i = \theta_j$: Q(0) = 0.00, p = .								
Test of $\theta = 0$: z = -2.84, p = 0.00								
**Heart disease (heart failure or ischemic heart disease)**								
Snoek et al. 2021	89	3.3	3.7	90	3.3	3.7	0.00 [-0.29, 0.29]	18.10
Heterogeneity: $\tau^2 = 0.00$, I^2 = .%, H^2 = .							0.00 [-0.29, 0.29]	
Test of $\theta_i = \theta_j$: Q(0) = 0.00, p = .								
Test of $\theta = 0$: z = 0.00, p = 1.00								
**Chronic obstructive pulmonary disease**								
Horton et al. 2018 + 2021	68	6.3	3.6	73	6.5	3.6	-0.07 [-0.40, 0.26]	15.61
Cox et al. 2022	47	-0.4	5.3	49	-1.3	4.8	0.18 [-0.22, 0.58]	11.98
Heterogeneity: $\tau^2 = 0.00$, $I^2 = 0.00\%$, $H^2 = 1.00$							0.03 [-0.22, 0.28]	
Test of $\theta_i = \theta_j$: Q(1) = 0.90, p = 0.34								
Test of $\theta = 0$: z = 0.23, p = 0.82								
**Osteoarthritis (knee or hip)**								
Bossen et al. 2013	75	2.4	5.1	72	3.0	4.8	-0.12 [-0.44, 0.20]	16.02
Bennell et al. 2018	65	2.7	4.2	62	2.6	3.9	0.02 [-0.32, 0.37]	14.59
Heterogeneity: $\tau^2 = 0.00$, $I^2 = 0.00\%$, $H^2 = 1.00$							-0.05 [-0.29, 0.18]	
Test of $\theta_i = \theta_j$: Q(1) = 0.36, p = 0.55								
Test of $\theta = 0$: z = -0.45, p = 0.66								
**Type 2 diabetes**								
Varney et al. 2014	35	18.0	11.6	36	16.0	11.8	0.17 [-0.29, 0.63]	9.58
Heterogeneity: $\tau^2 = 0.00$, I^2 = .%, H^2 = .							0.17 [-0.29, 0.63]	
Test of $\theta_i = \theta_j$: Q(0) = 0.00, p = .								
Test of $\theta = 0$: z = 0.72, p = 0.47								
**Overall**							-0.06 [-0.22, 0.10]	
Heterogeneity: $\tau^2 = 0.02$, $I^2 = 32.21\%$, $H^2 = 1.48$								
Test of $\theta_i = \theta_j$: Q(6) = 9.13, p = 0.17								
Test of $\theta = 0$: z = -0.76, p = 0.45								
Test of group differences: $Q_b(4) = 7.86$, p = 0.10								

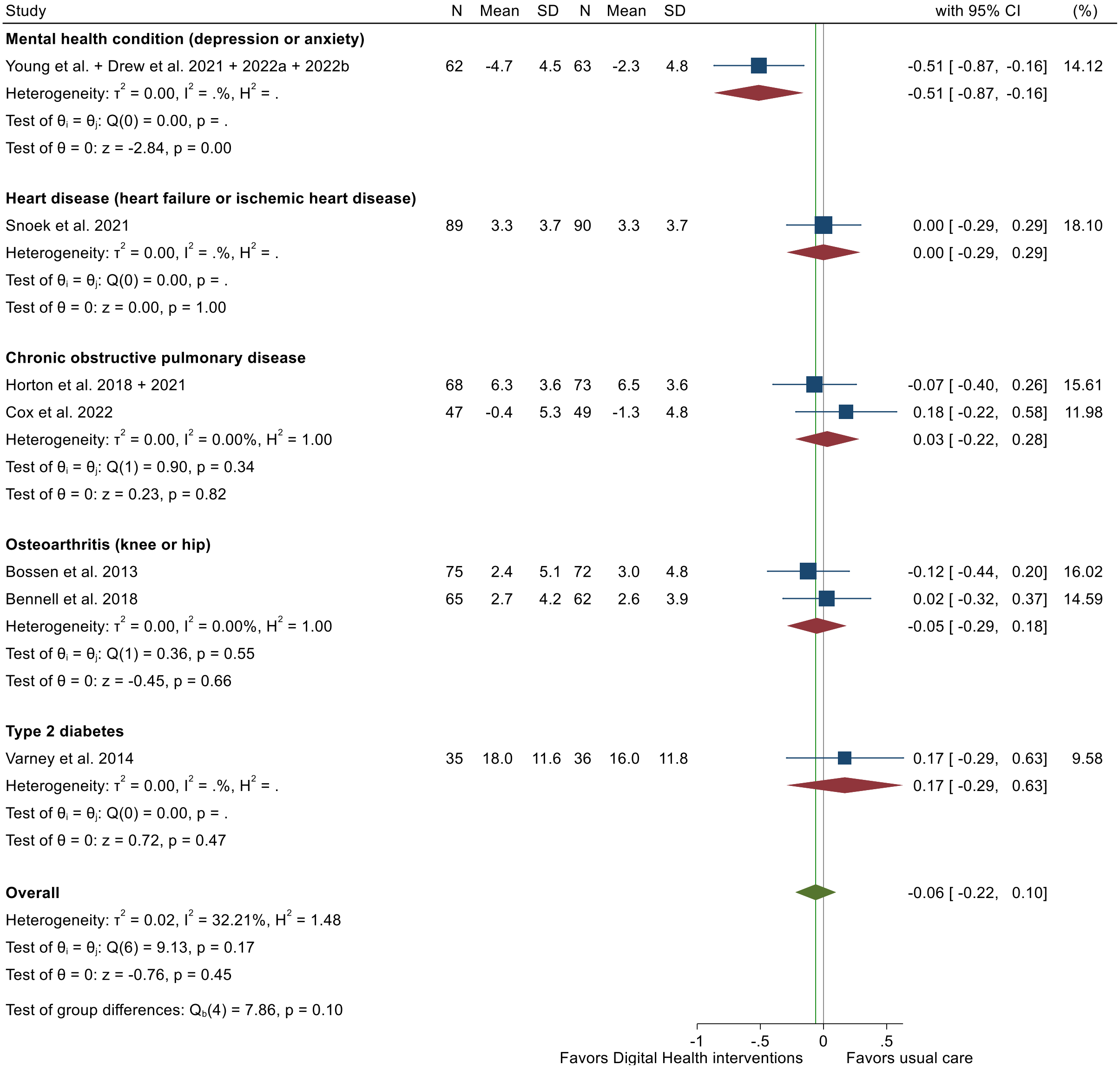

Supplementary Figure 21. Forest plot for anxiety at follow-up

Study	Treatment N	Treatment Mean	Treatment SD	Control N	Control Mean	Control SD	SMD with 95% CI	Weight (%)
**Mental health condition (depression or anxiety)**								
Young et al. + Drew et al. 2021 + 2022a + 2022b	62	-3.8	4.3	63	-2.9	4.2	-0.21 [-0.56, 0.14]	14.35
Heterogeneity: τ^2 = 0.00, I^2 = .%, H^2 = .							-0.21 [-0.56, 0.14]	
Test of $\theta_i = \theta_j$: Q(0) = 0.00, p = .								
Test of θ = 0: z = -1.18, p = 0.24								
**Heart disease (heart failure or ischemic heart disease)**								
Snoek et al. 2021	89	1.7	2.9	90	1.7	3.4	0.00 [-0.29, 0.29]	20.58
Heterogeneity: τ^2 = 0.00, I^2 = .%, H^2 = .							0.00 [-0.29, 0.29]	
Test of $\theta_i = \theta_j$: Q(0) = 0.00, p = .								
Test of θ = 0: z = 0.00, p = 1.00								
**Chronic obstructive pulmonary disease**								
Horton et al. 2018 + 2021	68	7.3	4.3	73	8.0	4.2	-0.15 [-0.48, 0.18]	16.18
Cerdan-De-las-heras et al. 2022	27	2.9	5.7	27	4.5	7.8	-0.23 [-0.76, 0.29]	6.29
Cox et al. 2022	47	-1.3	7.0	49	-1.3	5.2	0.00 [-0.40, 0.40]	11.12
Heterogeneity: τ^2 = 0.00, I^2 = 0.00%, H^2 = 1.00							-0.12 [-0.35, 0.11]	
Test of $\theta_i = \theta_j$: Q(2) = 0.57, p = 0.75								
Test of θ = 0: z = -1.01, p = 0.31								
**Osteoarthritis (knee or hip)**								
Bossen et al. 2013	75	3.1	5.1	72	4.1	5.0	-0.20 [-0.52, 0.12]	16.85
Bennell et al. 2018	65	2.0	2.9	62	2.3	3.3	-0.10 [-0.44, 0.25]	14.63
Heterogeneity: τ^2 = 0.00, I^2 = 0.00%, H^2 = 1.00							-0.15 [-0.39, 0.09]	
Test of $\theta_i = \theta_j$: Q(1) = 0.18, p = 0.67								
Test of θ = 0: z = -1.25, p = 0.21								
**Overall**							-0.12 [-0.25, 0.02]	
Heterogeneity: τ^2 = 0.00, I^2 = 0.00%, H^2 = 1.00								
Test of $\theta_i = \theta_j$: Q(6) = 1.72, p = 0.94								
Test of θ = 0: z = -1.73, p = 0.08								
Test of group differences: $Q_b(3)$ = 0.97, p = 0.81								

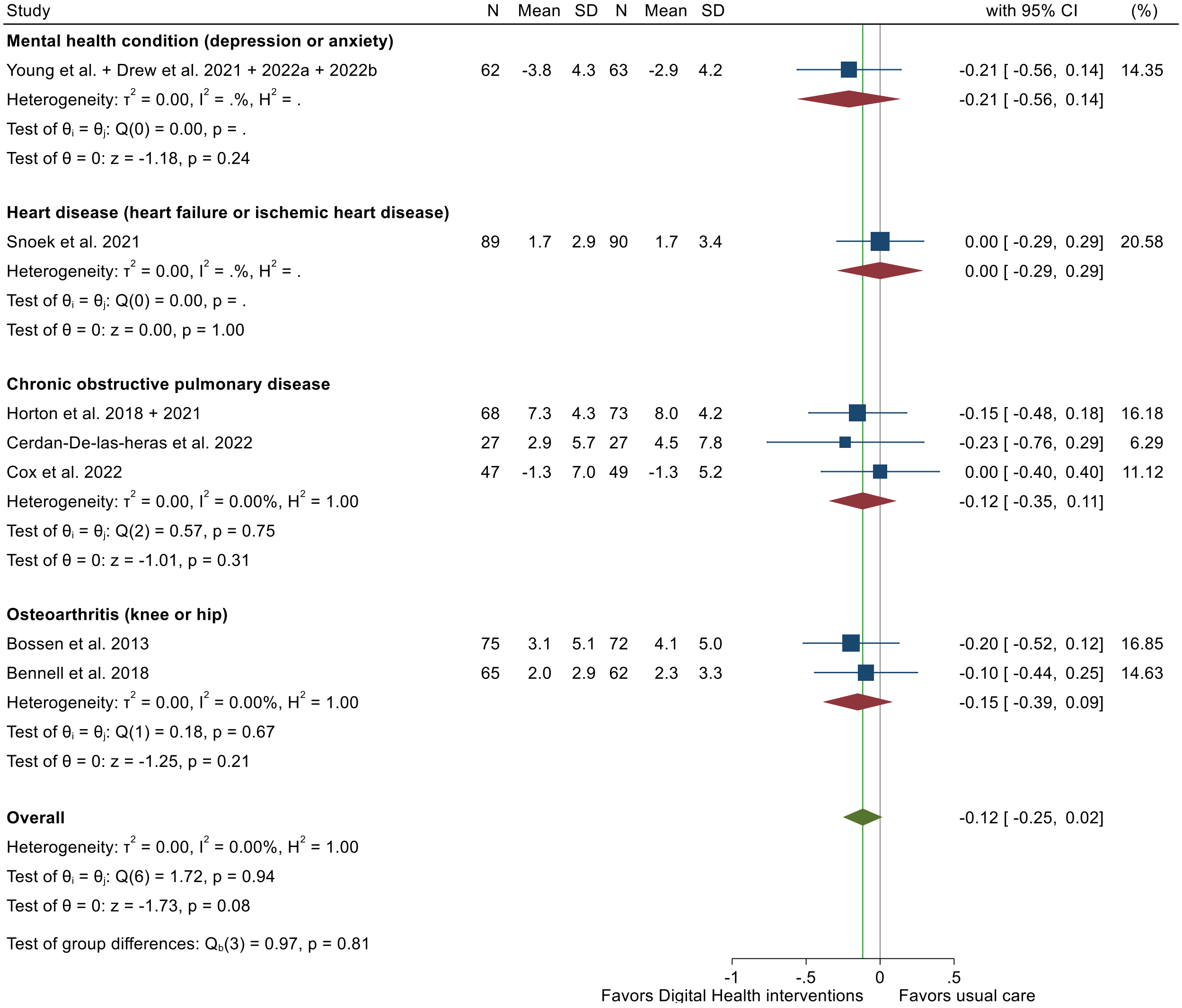

Supplementary Figure 22. Forest plot for health-related quality of life at follow-up

Study	Treatment N	Treatment Mean	Treatment SD	Control N	Control Mean	Control SD	SMD with 95% CI	Weight (%)
**Heart disease (heart failure or ischemic heart disease)**								
Reid et al. 2012	115	5.8	0.6	108	5.6	0.8	0.28 [0.02, 0.55]	7.71
Avila et al. 2019	26	79.9	30.0	25	73.9	40.7	0.17 [-0.38, 0.71]	4.35
Haejung et al. 2021	12	83.8	17.3	12	70.2	22.9	0.65 [-0.14, 1.45]	2.62
Radhakrishnan et al. 2021	19	10.8	16.9	19	15.4	19.6	-0.25 [-0.87, 0.38]	3.65
Snoek et al. 2021	89	50.6	7.2	90	49.0	8.2	0.21 [-0.09, 0.50]	7.30
Felker et al. 2022	82	72.2	21.6	78	67.6	22.8	0.21 [-0.10, 0.52]	7.06
Heterogeneity: $\tau^2 = 0.00$, $I^2 = 0.00\%$, $H^2 = 1.00$							0.22 [0.07, 0.37]	
Test of $\theta_i = \theta_j$: Q(5) = 3.54, p = 0.62								
Test of $\theta = 0$: z = 2.84, p = 0.00								
**Chronic obstructive pulmonary disease**								
Moy et al. 2016	154	-2.5	12.3	84	-1.4	9.1	-0.10 [-0.36, 0.17]	7.68
Wootton et al. 2018 + 2019	38	40.0	17.0	33	47.0	17.0	-0.41 [-0.87, 0.06]	5.10
Wan et al. 2020	35	-3.1	6.7	40	0.0	6.7	-0.46 [-0.91, -0.00]	5.22
Cerdan-De-las-heras et al. 2022	27	49.1	11.7	27	50.6	11.5	-0.13 [-0.66, 0.39]	4.49
Cox et al. 2022	47	0.0	8.9	49	-1.6	9.1	0.18 [-0.22, 0.58]	5.90
Heterogeneity: $\tau^2 = 0.02$, $I^2 = 28.85\%$, $H^2 = 1.41$							-0.16 [-0.37, 0.06]	
Test of $\theta_i = \theta_j$: Q(4) = 5.68, p = 0.22								
Test of $\theta = 0$: z = -1.43, p = 0.15								
**Osteoarthritis (knee or hip)**								
Bossen et al. 2013	75	48.7	34.9	71	47.5	35.0	0.03 [-0.29, 0.36]	6.88
Bennell et al. 2017	66	0.8	0.2	67	0.7	0.2	0.50 [0.15, 0.84]	6.60
Bennell et al. 2018	65	0.8	0.1	62	0.8	0.2	0.00 [-0.35, 0.35]	6.57
Hinman et al. 2019	82	0.7	0.2	76	0.7	0.2	0.00 [-0.31, 0.31]	7.05
Heterogeneity: $\tau^2 = 0.03$, $I^2 = 49.51\%$, $H^2 = 1.98$							0.13 [-0.10, 0.36]	
Test of $\theta_i = \theta_j$: Q(3) = 5.94, p = 0.11								
Test of $\theta = 0$: z = 1.08, p = 0.28								
**Type 2 diabetes**								
van der Weegen et al. 2015	65	44.1	9.5	68	45.8	9.5	-0.18 [-0.52, 0.16]	6.66
Heterogeneity: $\tau^2 = 0.00$, $I^2 = .\%$, $H^2 = .$							-0.18 [-0.52, 0.16]	
Test of $\theta_i = \theta_j$: Q(0) = 0.00, p = .								
Test of $\theta = 0$: z = -1.03, p = 0.30								
**Multimorbidity**								
Bernocchi et al. 2018	45	11.6	12.3	35	0.5	11.9	0.91 [0.45, 1.37]	5.17
Heterogeneity: $\tau^2 = 0.00$, $I^2 = .\%$, $H^2 = .$							0.91 [0.45, 1.37]	
Test of $\theta_i = \theta_j$: Q(0) = 0.00, p = .								
Test of $\theta = 0$: z = 3.87, p = 0.00								
**Overall**							0.09 [-0.06, 0.24]	
Heterogeneity: $\tau^2 = 0.06$, $I^2 = 61.98\%$, $H^2 = 2.63$								
Test of $\theta_i = \theta_j$: Q(16) = 39.65, p = 0.00								
Test of $\theta = 0$: z = 1.17, p = 0.24								
Test of group differences: $Q_b(4) = 22.26$, p = 0.00								

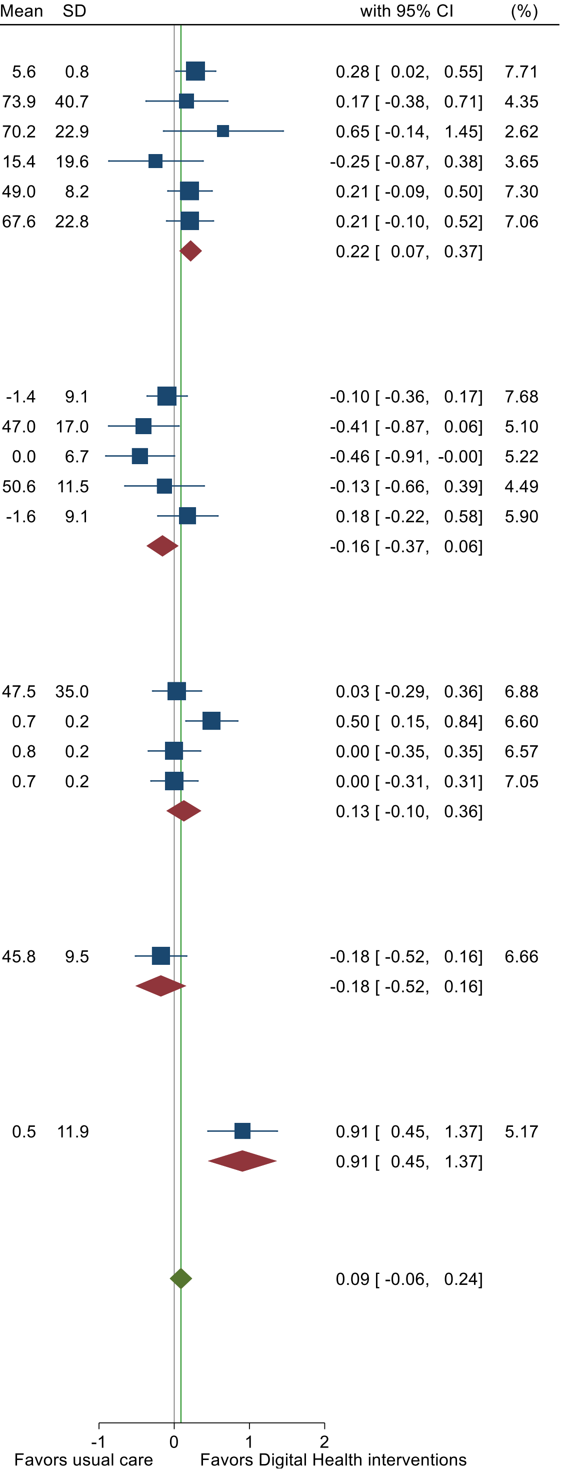

Supplementary Figure 23. Forest plot for adverse events, non-serious at follow-up

Study	Treatment Yes	Treatment No	Control Yes	Control No	Risk ratio with 95% CI	Weight (%)
Bossen et al. 2013	1	101	0	99	1.00 [0.02, 49.91]	2.32
Eakin et al. 2014	17	134	28	123	0.61 [0.35, 1.06]	17.29
Moy et al. 2016	43	111	8	76	2.93 [1.45, 5.94]	16.00
Bennell et al. 2017	27	38	8	62	3.63 [1.78, 7.42]	15.94
Chokshi et al. 2017	0	50	1	56	1.00 [0.02, 49.70]	2.32
Bennell et al. 2018	21	44	20	50	1.13 [0.68, 1.88]	17.68
Wootton et al. 2018 + 2019	1	50	0	46	1.00 [0.02, 49.48]	2.33
Hinman et al. 2019	2	85	1	89	5.02 [0.25, 102.42]	3.61
Jaarsma et al. 2021	5	282	3	285	1.67 [0.40, 6.93]	9.95
Young et al. + Drew et al. 2021 + 2022a + 2022b	4	58	11	52	0.37 [0.12, 1.10]	12.56
**Overall**					1.35 [0.71, 2.54]	

Heterogeneity: $\tau^2 = 0.52$, $I^2 = 68.99\%$, $H^2 = 3.23$

Test of $\theta_i = \theta_j$: Q(9) = 26.56, p = 0.00

Test of $\theta = 0$: z = 0.92, p = 0.36

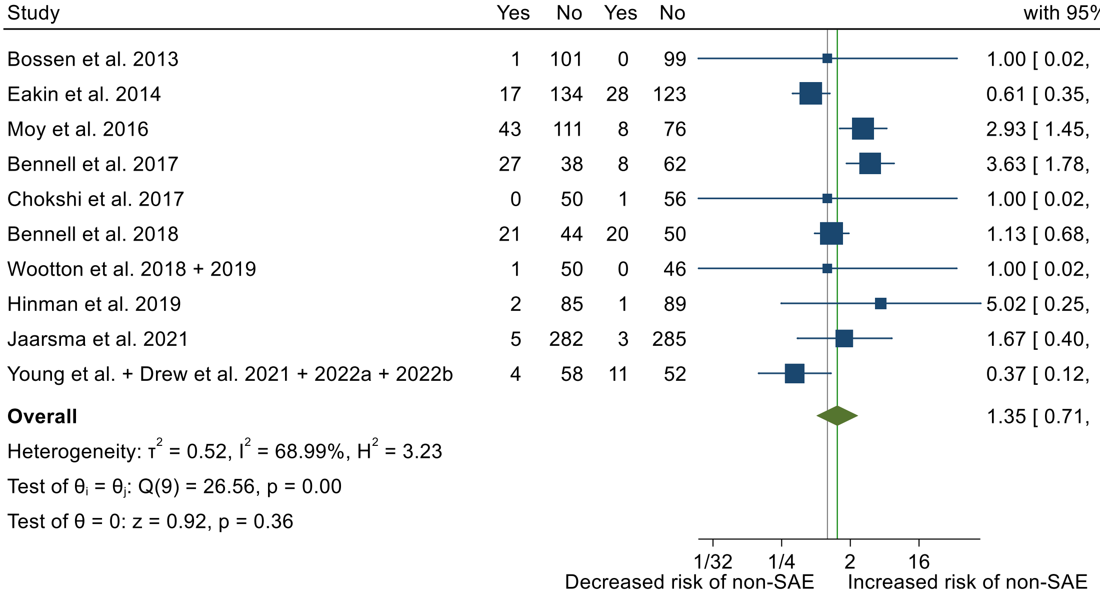

Supplementary Figure 24. Forest plot for adverse events, serious at follow-up

Study	Treatment Yes	Treatment No	Control Yes	Control No	Risk ratio with 95% CI	Weight (%)
Eakin et al. 2010	3	225	2	203	1.35 [0.23, 7.99]	2.91
Reid et al. 2012	4	111	9	99	0.42 [0.13, 1.32]	6.99
Bossen et al. 2013	1	101	0	99	1.00 [0.02, 49.91]	0.60
Eakin et al. 2014	4	147	4	147	1.00 [0.25, 3.93]	4.93
Moy et al. 2016	6	149	2	82	1.63 [0.34, 7.88]	3.70
Chokshi et al. 2017	10	33	13	30	0.77 [0.38, 1.56]	18.40
Bernocchi et al. 2018	3	45	7	37	0.39 [0.11, 1.43]	5.55
Wootton et al. 2018 + 2019	1	50	0	46	1.00 [0.02, 49.48]	0.61
Horton et al. 2018 + 2021	3	142	4	138	0.73 [0.17, 3.22]	4.21
Avila et al. 2019	3	25	0	25	4.92 [0.24, 100.16]	1.02
Jaarsma et al. 2021	3	301	5	293	0.59 [0.14, 2.44]	4.56
Osteresch et al. 2021	17	121	29	114	0.61 [0.35, 1.05]	30.35
Radhakrishnan et al. 2021	1	18	1	18	1.00 [0.07, 14.85]	1.27
Snoek et al. 2021	12	77	10	80	1.21 [0.55, 2.66]	14.91
**Overall**					0.76 [0.56, 1.03]	

Heterogeneity: $\tau^2 = 0.00$, $I^2 = 0.00\%$, $H^2 = 1.00$

Test of $\theta_i = \theta_j$: $Q(13) = 7.18$, $p = 0.89$

Test of $\theta = 0$: $z = -1.79$, $p = 0.07$

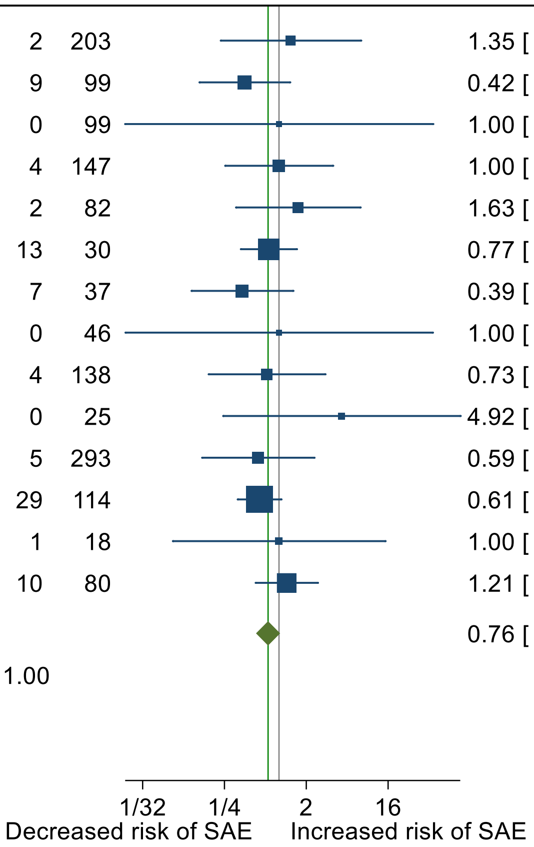

Supplement: Multimedia Appendix 4 [file jmir_v25i1e46439_app4.pdf]
