# Supplementary material for: Benefits and Harms of Digital Health Interventions Promoting Physical Activity in People With Chronic Conditions: Systematic Review and Meta-Analysis
Source: J Med Internet Res. 2023 Jul 6;25:e46439. doi: 10.2196/46439 (PMC10359919; doi:10.2196/46439)
Supplement: Multimedia Appendix 5 [file jmir_v25i1e46439_app5.pdf]

Multimedia Appendix 5: Risk of bias

To manuscript: **Benefits and Harms of Digital Health Interventions Promoting Physical Activity in People with Chronic Conditions: A Systematic Review and Meta-Analysis**

List of risk of bias Figures

Supplementary Figure 1. Risk of bias for each studies with objectively measured physical activity outcome
Supplementary Figure 2. Risk of bias for each studies with objectively measured physical function outcome
Supplementary Figure 3. Risk of bias for subjectively measured physical activity outcome given as percentages
Supplementary Figure 4. Risk of bias for each studies with subjectively measured physical activity outcome
Supplementary Figure 5. Risk of bias subjectively measured physical function outcome given as percentages
Supplementary Figure 6. Risk of bias for each studies with subjectively measured physical function outcome
Supplementary Figure 7. Risk of bias for depression outcome given as percentages
Supplementary Figure 8. Risk of bias for each studies with depression outcome
Supplementary Figure 9. Risk of bias for anxiety outcome given as percentages
Supplementary Figure 10. Risk of bias for each studies with anxiety outcome
Supplementary Figure 11. Risk of bias health-related quality of life outcome given as percentages
Supplementary Figure 12. Risk of bias for each studies with health-related quality of life outcome

Supplementary Figure 1. Risk of bias for each studies with objectively measured physical activity outcome

Study ID	D1	D2	D3	D4	D5	Overall
Agboola et al. 2016	+	+	+	+	-	!
Akinci et al. 2018	+	+	+	+	-	!
Alonso-Dominguez et al. 2019	+	!	!	+	-	-
Avila et al. 2018 + 2019	+	+	+	!	-	!
Bartholdy et al. 2019	+	+	+	+	!	!
Bender et al. 2017	+	+	+	+	+	+
Bentley et al. 2020	+	-	-	-	!	-
Bossen et al. 2013	+	+	+	+	!	!
Chokshi et al. 2017	+	+	+	+	!	!
De Greef et al. 2011	!	+	+	!	!	!
Demeyer et al. 2017	+	!	+	+	!	!
Dyson et al. 2010	!	+	+	+	!	!
Eakin et al. 2014	+	+	+	+	+	+
Frederix et al. 2015	+	+	+	+	+	+
Guiraud et al. 2012	!	!	+	+	!	!
Hochsmann et al. 2019	!	+	+	+	+	!
Hornikx et al. 2015	!	!	!	+	!	!
Lambert et al. 2018	-	-	+	+	!	-
Li et al. 2018	+	+	+	+	-	!
Moy et al. 2015	!	+	+	+	!	!
Piette et al. 2011	+	+	-	-	!	-
Plotnikoff et al. 2013	+	+	-	+	!	-

\+ Low risk
! Some concerns
\- High risk

D1 Randomisation process
D2 Deviations from the intended interventions
D3 Missing outcome data
D4 Measurement of the outcome
D5 Selection of the reported result

Plotnikoff et al. 2013	+	+	-	+	!	-
Poppe et al. 2019	-	+	-	+	!	-
Reid et al. 2012	+	-	-	+	!	-
Tsai et al. 2017	-	+	+	+	!	-
van der Weegen et al. 2015	+	+	!	+	+	!
Vasilopoulou et al. 2014	!	!	+	+	!	!
Wan et al. 2017	+	!	+	+	!	!
Wootton et al. 2018 + 2019	-	+	!	+	!	-
Bailey et al. 2020	+	-	+	+	!	-
Benzo et al. 2021	-	-	-	+	!	-
Connelly et al. 2017	+	-	+	+	!	-
Connelly et al. 2017	+	-	+	+	!	-
Coombes et al. 2021	+	-	!	+	!	-
Li et al. 2020	+	+	+	+	!	!
Robinson et al. 2021	+	+	-	+	+	!
Alghafri et al. 2018	!	+	-	+	+	-
Andrade et al. 2021	!	+	+	+	!	!
Benzo et al. 2022	!	+	+	+	!	!
Cerdan-De-las-heras et al. 2022	!	+	-	+	!	-
Chan et al. 2022	+	+	+	+	!	!
Chaplin et al. 2016 + 2022	!	+	-	-	+	-
Cox et al. 2022	!	!	+	+	+	!
Young et al. + Drew et al. 2021 + 2022a + 2022b	+	+	+	+	!	!
Felker et al. 2022	!	+	-	+	!	-
Horton et al. 2018 + 2021	+	-	-	+	!	-
Radhakrishnan et al. 2021	+	+	+	+	!	!

Reid et al. 2021	+	+	+	+	!	!
Reid et al. 2021	+	+	+	+	!	!
Spielmanns et al. 2022	+	+	!	+	+	!

Supplementary Figure 2. Risk of bias for each studies with objectively measured physical function outcome

Study	D1	D2	D3	D4	D5	Overall
Akinci et al. 2018	+	+	+	+	-	!
Baker et al. 2019	+	+	-	!	+	-
Bentley et al. 2020	+	-	-	-	!	-
Bernocchi et al. 2018	+	!	!	+	+	!
Cameron-Tucker et al. 2016	+	+	+	+	!	!
de Sousa Pinto et al. 2014	!	!	!	!	!	!
Deka et al. 2018	+	+	+	!	!	!
Demeyer et al. 2017	+	!	+	+	!	!
Duruturk et al. 2019	!	+	+	!	!	!
Hornikx et al. 2015	!	!	!	+	!	!
Kwon et al. 2018	!	!	!	+	!	!
Kwon et al. 2018	!	!	!	+	!	!
Moore et al. 2009	!	-	+	+	!	-
Peng et al. 2018	+	!	+	+	!	!
Piotrowicz et al. 2015	-	!	+	+	!	-
Piotrowicz et al. 2019	+	!	+	+	+	!
Tsai et al. 2017	-	+	+	+	!	-
Vasilopoulou et al. 2014	!	!	+	+	!	!
Wan et al. 2017	+	!	+	+	!	!
Wootton et al. 2018 +2019	+	+	!	+	!	!
Allen et al. 2018	!	+	+	+	+	!
Allen et al. 2021	+	+	-	+	+	!
Coultas et al. 2016	+	-	-	+	+	-
Doiron-Cadrin et al. 2020	+	+	+	+	+	+

+ Low risk
! Some concerns
- High risk

D1 Randomisation process
D2 Deviations from the intended interventions
D3 Missing outcome data
D4 Measurement of the outcome
D5 Selection of the reported result

Galdiz et al. 2021	+	+	+	!	!	!
Gohir et al. 2021	+	+	+	+	+	+
Hsu et al. 2021	+	+	+	+	!	!
Jiménez-Reguera et al. 2020	+	!	+	+	!	!
Jaarsma et al. 2021	!	+	!	+	!	!
Li et al. 2021	!	-	+	+	!	-
Robinson et al. 2021	+	+	-	+	+	!
Witham et al. 2011	+	+	+	+	!	!
Andrade et al. 2021	!	+	+	+	!	!
Cerdan-De-las-heras et al. 2022	!	+	-	+	!	-
Chan et al. 2022	+	+	+	+	!	!
Chaplin et al. 2016 + 2022	!	+	-	-	+	-
Clays et al. 2021	+	-	+	!	!	-
Cox et al. 2022	!	!	+	+	+	!
Lee et al. 2021	!	-	!	+	!	-
Horton et al. 2018 + 2021	+	-	-	+	!	-
Indraratna et al. 2022	!	+	+	+	!	!
Li et al. 2022	+	!	+	+	!	!
Nagatomi et al. 2022	!	!	+	+	!	!
Shi et al. 2022	!	!	+	+	!	!
Spielmanns et al. 2022	+	+	!	+	+	!
Tore et al. 2022	!	!	+	+	!	!
Yudi et al. 2021	!	+	+	+	+	!

Supplementary Figure 3. Risk of bias for subjectively measured physical activity outcome given as percentages

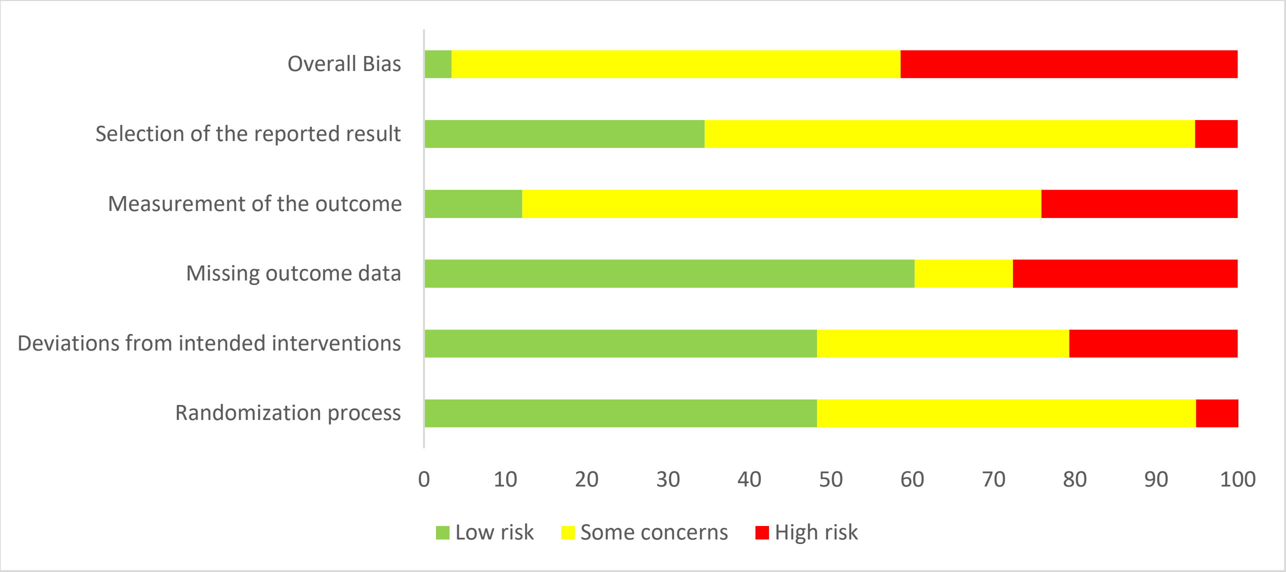

Supplementary Figure 4. Risk of bias for each studies with subjectively measured physical activity outcome

Study	D1	D2	D3	D4	D5	Overall
Baker et al. 2019	+	+	-	+	+	!
Bennell et al. 2018	+	+	+	+	+	+
Benson et al. 2019	+	+	+	!	!	!
Bernocchi et al. 2018	+	!	!	!	+	!
Cameron-Tucker et al. 2016	+	+	+	+	!	!
Chow et al. 2015	+	+	+	!	+	!
Cicolini et al. 2014	+	+	+	!	!	!
Deka et al. 2018	+	+	+	!	!	!
Dobler et al. 2018	!	-	-	!	!	-
Duan et al. 2018	!	-	+	!	!	-
Eakin et al. 2009	!	+	+	!	+	!
Gingele et al. 2019	+	!	!	!	!	!
Glasgow et al. 2012	!	!	+	!	!	!

+ Low risk
! Some concerns
- High risk

D1 Randomisation process
D2 Deviations from the intended interventions
D3 Missing outcome data
D4 Measurement of the outcome
D5 Selection of the reported result

Glasgow et al. 2012	!	!	+	!	!	!
Haller et al. 2018	!	!	!	!	!	!
Hanssen et al. 2007	+	-	-	-	!	-
Hawkes et al. 2013	+	+	+	-	+	!
Hidrus et al. 2020	!	-	-	-	!	-
Hinman et al. 2019	!	+	+	+	+	!
Holmen et al. 2014	-	-	-	-	+	-
Holmen et al. 2014	-	-	-	-	+	-
Jayasree et al. 2019	!	!	-	-	!	-
Jennings et al. 2019	+	+	-	-	!	-
Kim et al. 2006	!	!	+	-	!	-
Kooiman et al. 2018	!	+	+	-	!	-
Liebreich et al. 2009	!	!	+	-	!	-
Namjoo Nasab et al. 2017	!	-	+	-	!	-
Southard et al. 2003	!	!	+	-	-	-
Strom et al. 2013	-	+	+	!	-	-
Tomita et al. 2009	!	!	!	-	!	-
Kavradim 2020	+	!	+	!	!	!
Varney et al. 2014	!	-	-	!	-	-
Vinitha et al. 2019	+	+	!	!	!	!
Widmer et al. 2017	!	-	!	+	!	-
Allen et al. 2018	!	+	+	!	+	!
Allen et al. 2021	+	+	-	!	+	-
Anderson et al. 2010	!	-	-	!	!	-
Bennell et al. 2020	+	+	+	+	!	!
Coultas et al. 2016	+	-	-	!	+	-
Jaarsma et al. 2021	!	+	!	!	!	!
Lim et al. 2021	+	+	+	-	+	!
Nelligan et al. 2021	+	+	+	+	+	+

Pelle et al. 2020	+	+	-	!	!	-
Snoek et al. 2021	+	!	+	!	+	!
Vluggen et al. 2021	+	+	+	!	+	!
Waller et al. 2021	+	!	+	!	+	!
Wang et al. 2021	+	+	+	!	!	!
Wong et al. 2020	+	+	+	!	!	!
Bozorgi et al. 2021	!	!	+	!	+	!
Lee et al. 2021	!	-	-	!	!	-
Lee et al. 2018	!	+	-	!	!	-
Michelsen et al. 2022	+	!	+	!	!	!
O’Brien et al. 2018	+	+	+	!	+	!
Osteresch et al. + Wienbergen et al. 2019 + 2021	!	!	-	!	!	-
Pamungkas et al. 2022	!	!	+	!	!	!
Pitta et al. 2022	+	+	+	!	+	!
Tore et al. 2022	!	!	+	!	!	!
Wong et al. 2021	!	+	+	!	!	!

Supplementary Figure 5. Risk of bias subjectively measured physical function outcome given as percentages

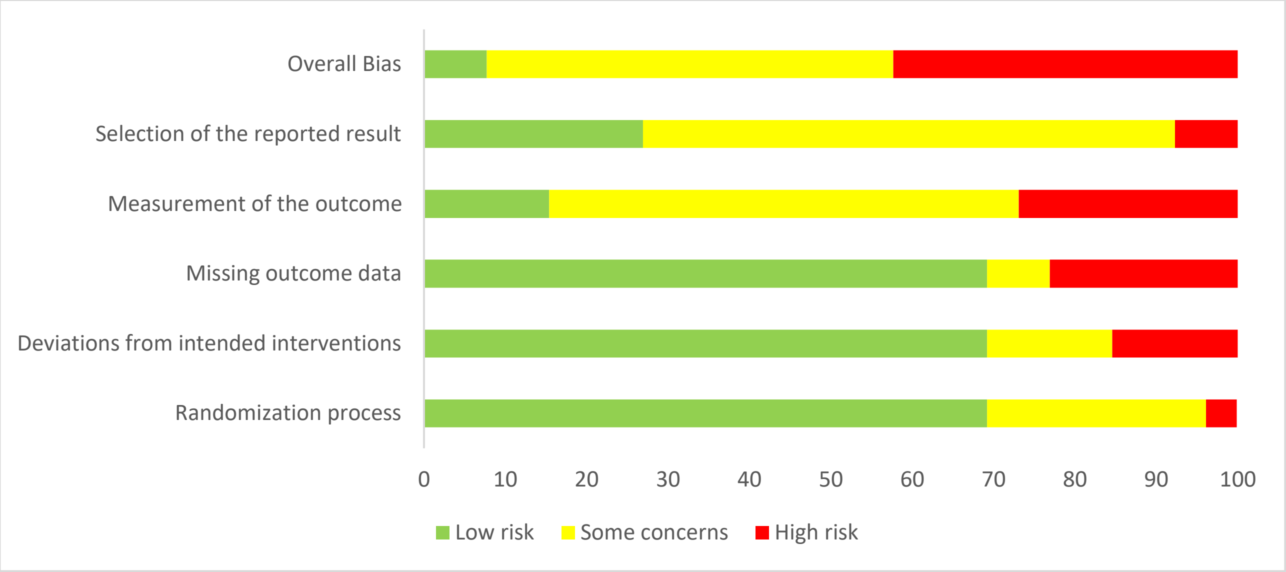

Supplementary Figure 6. Risk of bias for each studies with subjectively measured physical function outcome

Study	D1	D2	D3	D4	D5	Overall
Avila et al. 2018 + 2019	+	+	+	!	+	!
Bartholdy et al. 2019	+	+	+	!	!	!
Bennell et al. 2017	+	+	+	!	+	!
Bennell et al. 2018	+	+	+	+	+	+
Bossen et al. 2013	+	+	+	-	!	-
Haller et al. 2018	!	!	!	!	!	!
Hanssen et al. 2007	+	-	-	-	!	-
Hawkes et al. 2013	+	+	+	-	+	!
Hinman et al. 2019	!	+	+	+	+	!
Li et al. 2018	+	+	+	-	-	-
Moy et al. 2015	!	+	-	!	!	-

\+ Low risk

! Some concerns

\- High risk

D1	Randomisation process
D2	Deviations from the intended interventions
D3	Missing outcome data
D4	Measurement of the outcome
D5	Selection of the reported result

Piette et al. 2011	+	+	-	-	!	-
Reid et al. 2012	+	-	-	-	!	-
Southard et al. 2003	!	!	+	-	-	-
Utriyaprasit 2010	!	-	-	!	!	-
Bennell et al. 2020	+	+	+	+	!	!
Coombes et al. 2021	+	-	!	!	!	-
Jiwani et al. 2020	-	!	+	!	!	-
Li et al. 2020	+	+	+	!	!	!
Nelligan et al. 2021	+	+	+	+	+	+
Pelle et al. 2020	+	+	-	!	!	-
Benzo et al. 2022	!	+	+	!	!	!
O'Brien et al. 2018	+	+	+	!	+	!
Radhakrishnan et al. 2021	+	+	+	!	!	!
Wong et al. 2021	!	+	+	!	!	!
Furuya et al. 2014	+	!	+	!	!	!

Supplementary Figure 7. Risk of bias for depression outcome given as percentages

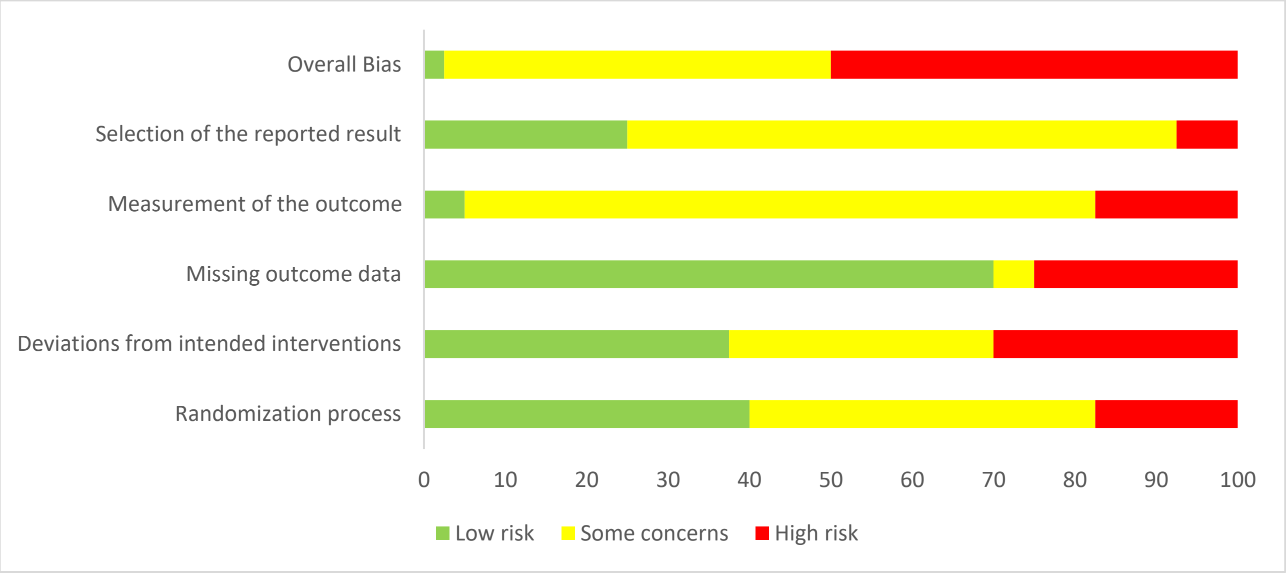

Supplementary Figure 8. Risk of bias for each studies with depression outcome

Study	D1	D2	D3	D4	D5	Overall
Bennell et al. 2018	+	+	+	+	+	+
Bentley et al. 2020	+	-	-	!	!	-
Bossen et al. 2013	+	+	+	-	!	-
Dobler et al. 2018	!	-	-	!	!	-
Duan et al. 2018	!	-	+	!	!	-
Duruturk et al. 2019	!	+	+	!	!	!
Haller et al. 2018	!	!	!	!	!	!
Holmen et al. 2014	-	-	-	-	+	-
Holmen et al. 2014	-	-	-	-	+	-
Lambert et al. 2018	-	-	+	-	!	-
Peng et al. 2018	+	!	+	-	!	-
Piette et al. 2011	+	+	-	-	!	-

+ Low risk
! Some concerns
- High risk

D1 Randomisation process
D2 Deviations from the intended interventions
D3 Missing outcome data
D4 Measurement of the outcome
D5 Selection of the reported result

Piotrowicz et al. 2016	-	-	-	!	!	-
Southard et al. 2003	!	!	+	-	-	-
Strom et al. 2013	-	+	+	!	-	-
Tsai et al. 2017	-	+	+	!	!	-
Utriyaprasit 2010	!	-	-	!	!	-
Varney et al. 2014	!	-	-	!	-	-
Wan et al. 2017	+	!	+	!	!	!
Widmer et al. 2017	!	-	!	+	!	-
Jiwani et al. 2020	-	!	+	!	!	-
Li et al. 2020	+	+	+	!	!	!
Snoek et al. 2021	+	!	+	!	+	!
Witham et al. 2011	+	+	+	!	!	!
Andrade et al. 2021	!	+	+	!	!	!
Benzo et al. 2022	!	+	+	!	!	!
Clays et al. 2021	+	-	+	!	!	-
Cox et al. 2022	!	!	+	!	+	!
Young et al. + Drew et al. 2021 + 2022a + 2022b	+	+	+	!	!	!
Horton et al. 2018 + 2021	+	-	-	!	!	-
Li et al. 2022	+	!	+	!	!	!
O'Brien et al. 2018	+	+	+	!	+	!
Osteresch et al. + Wienbergen et al. 2019 + 2021	!	!	-	!	!	-
Pitta et al. 2022	+	+	+	!	+	!
Snoek et al. 2021	!	!	+	!	+	!
Spielmanns et al. 2022	!	!	+	!	+	!
Tore et al. 2022	!	!	+	!	!	!
Wong et al. 2021	!	+	+	!	!	!

Yudi et al. 2021	!	+	+	!	+	!
Furuya et al. 2014	+	!	+	!	!	!

Supplementary Figure 9. Risk of bias for anxiety outcome given as percentages

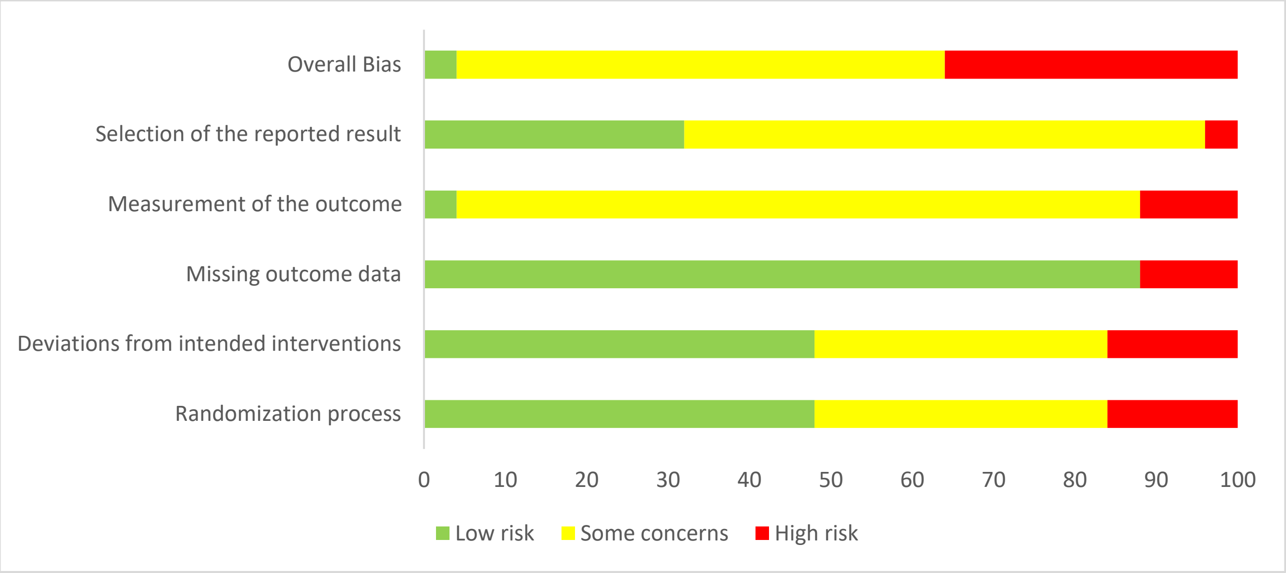

Supplementary Figure 10. Risk of bias for each studies with anxiety outcome

Study ID	D1	D2	D3	D4	D5	Overall
Bennell et al. 2018	+	+	+	+	+	+
Bossen et al. 2013	+	+	+	-	!	-
Lambert et al. 2018	-	-	+	-	!	-
Peng et al. 2018	+	!	+	-	!	-
Strom et al. 2013	-	+	+	!	-	-
Tsai et al. 2017	-	+	+	!	!	-
Utriyaprasit 2010	!	-	-	!	!	-
Jiwani et al. 2020	-	!	+	!	!	-
Snoek et al. 2021	+	!	+	!	+	!
Witham et al. 2011	+	+	+	!	!	!
Benzo et al. 2022	!	+	+	!	!	!
Cerdan-De-las-heras et al. 2022	!	+	-	!	!	-
Clays et al. 2021	+	-	+	!	!	!

\+ Low risk
! Some concerns
\- High risk

D1 Randomisation process
D2 Deviations from the intended interventions
D3 Missing outcome data
D4 Measurement of the outcome
D5 Selection of the reported result

Cox et al. 2022	!	!	+	!	+	!
Young et al. + Drew et al. 2021 + 2022a + 2022b	+	+	+	!	!	!
Horton et al. 2018 + 2021	+	-	-	!	!	-
Li et al. 2022	+	!	+	!	!	!
O’Brien et al. 2018	+	+	+	!	+	!
Pitta et al. 2022	+	+	+	!	+	!
Snoek et al. 2021	!	!	+	!	+	!
Spielmanns et al. 2022	!	!	+	!	+	!
Tore et al. 2022	!	!	+	!	!	!
Wong et al. 2021	!	+	+	!	!	!
Yudi et al. 2021	!	+	+	!	+	!
Furuya et al. 2014	+	!	+	!	!	!

Supplementary Figure 11. Risk of bias health-related quality of life outcome given as percentages

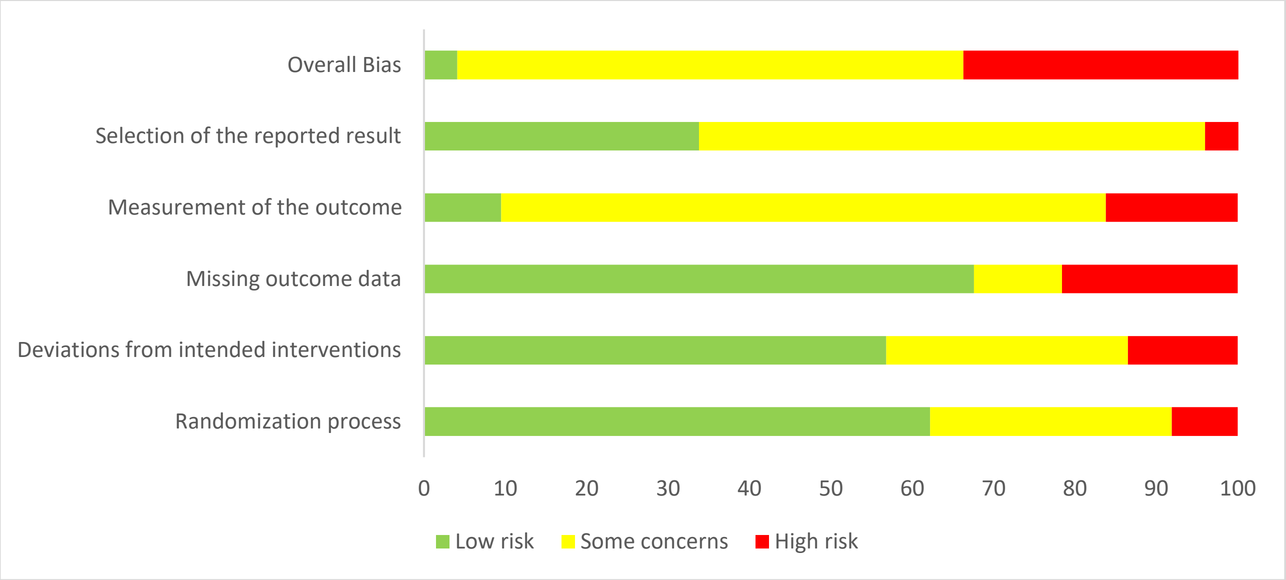

Supplementary Figure 12. Risk of bias for each studies with health-related quality of life outcome

Study	D1	D2	D3	D4	D5	Overall
Akinci et al. 2018	+	+	+	+	+	+
Avila et al. 2018 + 2019	+	+	+	!	+	!
Bartholdy et al. 2019	+	+	+	!	!	!
Bennell et al. 2017	+	+	+	!	+	!
Bennell et al. 2018	+	+	+	+	+	+
Bentley et al. 2020	+	-	-	!	!	-
Bernocchi et al. 2018	+	!	!	!	+	!
Bossen et al. 2013	+	+	+	-	!	-
Cameron-Tucker et al. 2016	+	+	+	+	!	!
de Sousa Pinto et al. 2014	!	!	!	!	!	!
Duan et al. 2018	!	-	+	!	!	-
Dyson et al. 2010	!	!	+	!	!	!

\+ Low risk
! Some concerns
\- High risk

D1 Randomisation process
D2 Deviations from the intended interventions
D3 Missing outcome data
D4 Measurement of the outcome
D5 Selection of the reported result

Frederix et al. 2015	+	+	+	!	+	!
Gingele et al. 2019	+	!	!	!	!	!
Hanssen et al. 2007	+	-	-	-	!	-
Hawkes et al. 2013	+	+	+	-	+	!
Hinman et al. 2019	!	+	+	+	+	!
Holmen et al. 2014	-	-	-	-	+	-
Holmen et al. 2014	-	-	-	-	+	-
Li et al. 2018	+	+	+	-	-	-
Moy et al. 2015	!	+	-	!	!	-
Peng et al. 2018	+	!	+	-	!	-
Piette et al. 2011	+	+	-	-	!	-
Piotrowicz et al. 2019	+	!	+	!	+	!
Plotnikoff et al. 2013	+	+	-	-	+	-
Plotnikoff et al. 2013	+	+	-	-	+	-
Reid et al. 2012	+	-	-	-	!	-
Strom et al. 2013	-	+	+	!	-	-
Tomita et al. 2009	!	!	!	-	!	-
Tsai et al. 2017	-	+	+	!	!	-
Kavradim 2020	+	!	+	!	!	!
van der Weegen et al. 2015	+	+	!	!	+	!
Vasilopoulou et al. 2014	!	!	+	!	!	!
Vinitha et al. 2019	+	+	!	!	!	!
Wan et al. 2017	+	!	+	!	!	!
Widmer et al. 2017	!	-	!	+	!	-
Wootton et al. 2018 + 2019	+	+	+	!	!	!
Bennell et al. 2020	+	+	+	+	!	!

Benzo et al. 2021	-	-	-	!	-	-
Doiron-Cadrin et al. 2020	+	+	+	!	+	!
Galdiz et al. 2021	+	+	!	!	!	!
Gohir et al. 2021	+	+	+	!	+	!
Jiménez-Reguera et al. 2020	+	!	+	!	!	!
Jiwani et al. 2020	-	!	+	!	!	-
Li et al. 2020	+	+	+	!	!	!
Nelligan et al. 2021	+	+	+	+	+	+
Pelle et al. 2020	+	+	-	!	!	-
Robinson et al. 2021	+	+	-	!	+	-
Snoek et al. 2021	+	!	+	!	+	!
Waller et al. 2021	+	!	+	!	+	!
Wang et al. 2021	+	+	+	!	!	!
Witham et al. 2011	+	+	+	!	!	!
Andrade et al. 2021	!	+	+	!	!	!
Benzo et al. 2022	!	+	+	!	!	!
Cerdan-De-las-heras et al. 2022	!	+	-	!	!	-
Chan et al. 2022	+	+	+	!	!	!
Clays et al. 2021	+	-	+	!	!	!
Cox et al. 2022	!	!	+	!	+	!
Felker et al. 2022	!	+	-	!	!	-
Lee et al. 2021	!	-	-	!	!	-
Indraratna et al. 2022	!	+	+	!	!	!
Li et al. 2022	+	!	+	!	!	!
Nagatomi et al. 2022	!	!	+	!	!	!
O’Brien et al. 2018	+	+	+	!	+	!

Osteresch et al. + Wienbergen et al. 2019 + 2021	!	!	-	!	!	-
Radhakrishnan et al. 2021	+	+	+	!	!	!
Reid et al. 2021	+	+	+	!	!	!
Reid et al. 2021	+	+	+	!	!	!
Snoek et al. 2021	!	!	+	!	+	!
Spielmanns et al. 2022	!	!	+	!	+	!
Tore et al. 2022	!	!	+	!	!	!
Wong et al. 2021	!	+	+	!	!	!
Yudi et al. 2021	!	+	+	!	+	!
Furuya et al. 2014	+	!	+	!	!	!
