# Supplementary material for: Benefits and Harms of Digital Health Interventions Promoting Physical Activity in People With Chronic Conditions: Systematic Review and Meta-Analysis
Source: J Med Internet Res. 2023 Jul 6;25:e46439. doi: 10.2196/46439 (PMC10359919; doi:10.2196/46439)
Supplement: Multimedia Appendix 6 [file jmir_v25i1e46439_app6.pdf]

Multimedia Appendix 6: Small study bias

To manuscript: **Benefits and Harms of Digital Health Interventions Promoting Physical Activity in People with Chronic Conditions: A Systematic Review and Meta-Analysis**

List of funnel plots Figures and Table with Egger's or Harbord's test for each outcome

Supplementary Figure 1. Funnel plot for objectively measured physical activity at end-of-intervention
Supplementary Figure 2. Funnel plot for objectively measured physical function at end-of-intervention
Supplementary Figure 3. Funnel plot for subjectively measured physical activity at end-of-intervention
Supplementary Figure 4. Funnel plot for subjectively measured physical function at end-of-intervention
Supplementary Figure 5. Funnel plot for depression at end-of-intervention
Supplementary Figure 6. Funnel plot for anxiety at end-of-intervention
Supplementary Figure 7. Funnel plot for health-related quality of life at end-of-intervention
Supplementary Figure 8. Funnel plot for objectively measured physical activity at follow-up
Supplementary Figure 9. Funnel plot for objectively measured physical function at follow-up
Supplementary Figure 10. Funnel plot for subjectively measured physical activity at follow-up
Supplementary Figure 11. Funnel plot for subjectively measured physical function at follow-up
Supplementary Figure 12. Funnel plot for depression at follow-up
Supplementary Figure 13. Funnel plot for anxiety at follow-up
Supplementary Figure 14. Funnel plot for health-related quality of life at follow-up
Supplementary Table 1. Results of the Egger's and Harbord's test for small study bias for each outcome

Supplementary Figure 1. Funnel plot for objectively measured physical activity at end-of-intervention

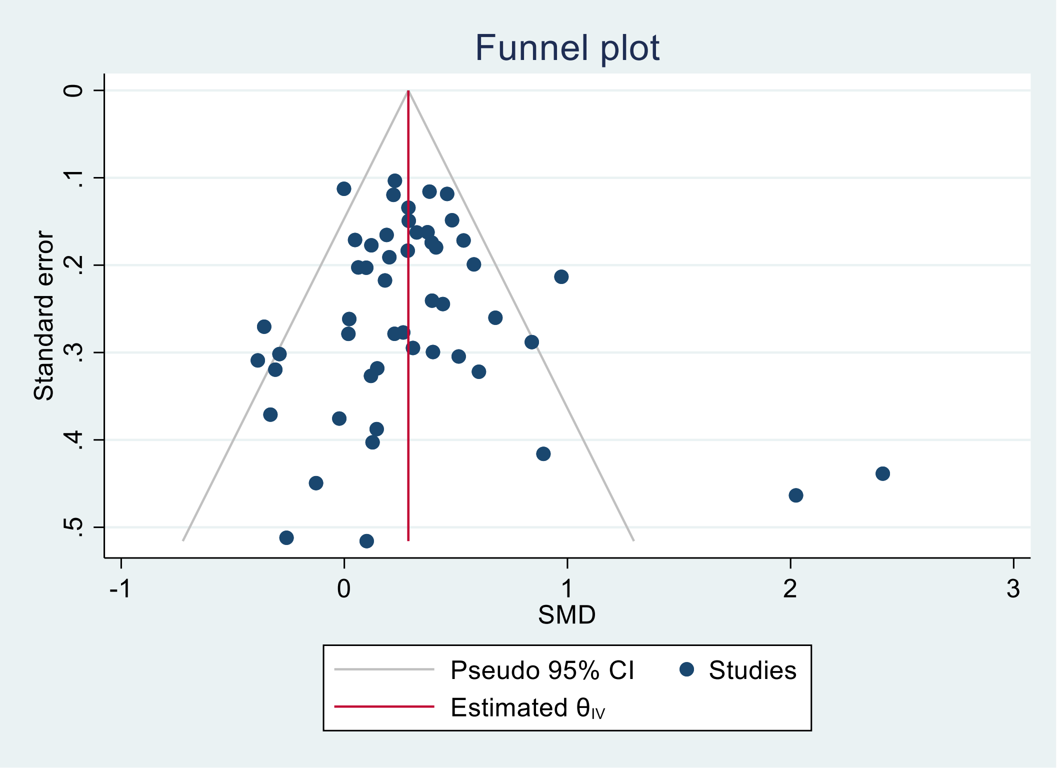

Supplementary Figure 2. Funnel plot for objectively measured physical function at end-of-intervention

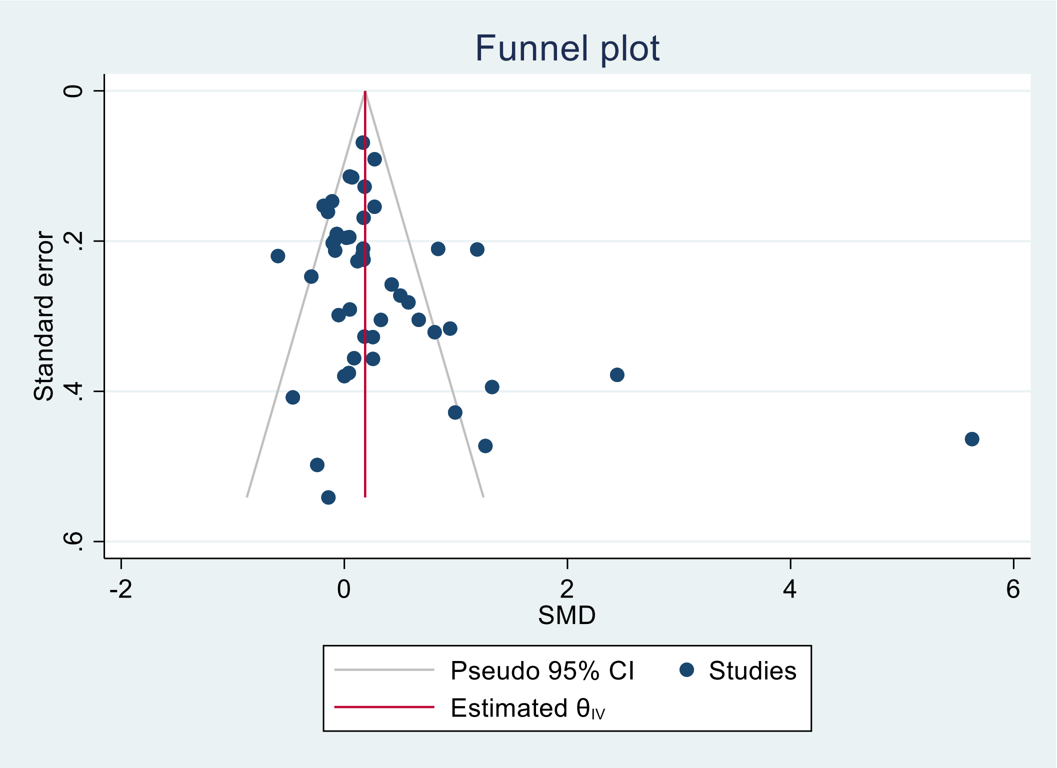

Supplementary Figure 3. Funnel plot for subjectively measured physical activity at end-of-intervention

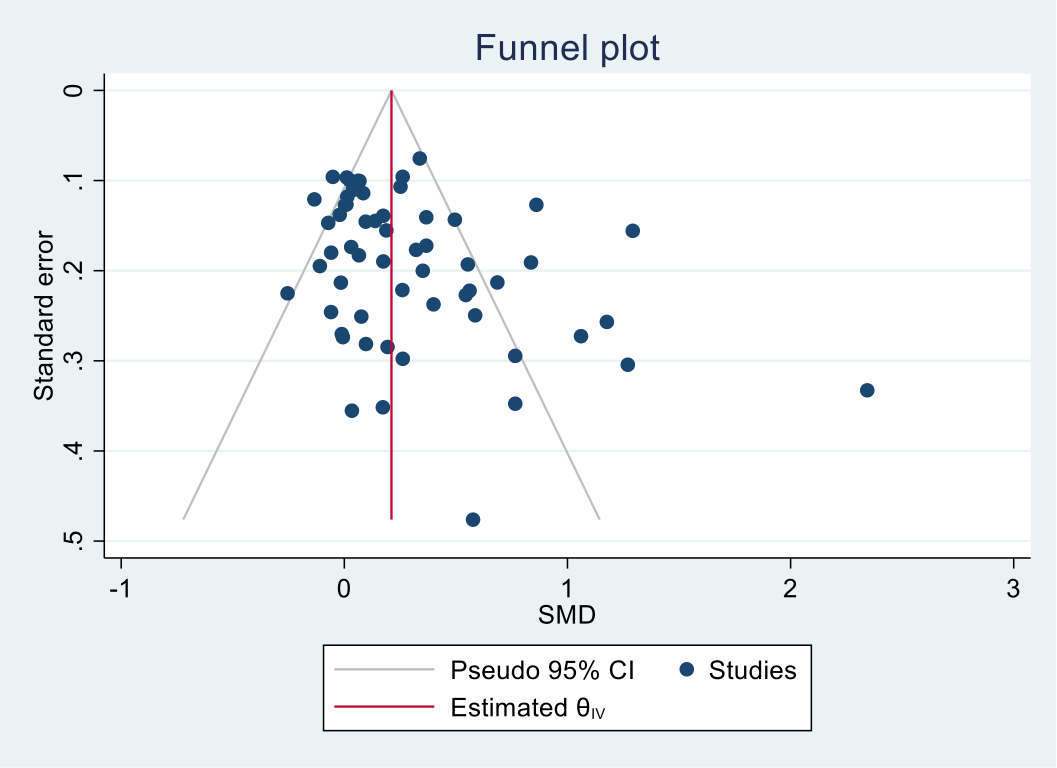

Supplementary Figure 4. Funnel plot for subjectively measured physical function at end-of-intervention

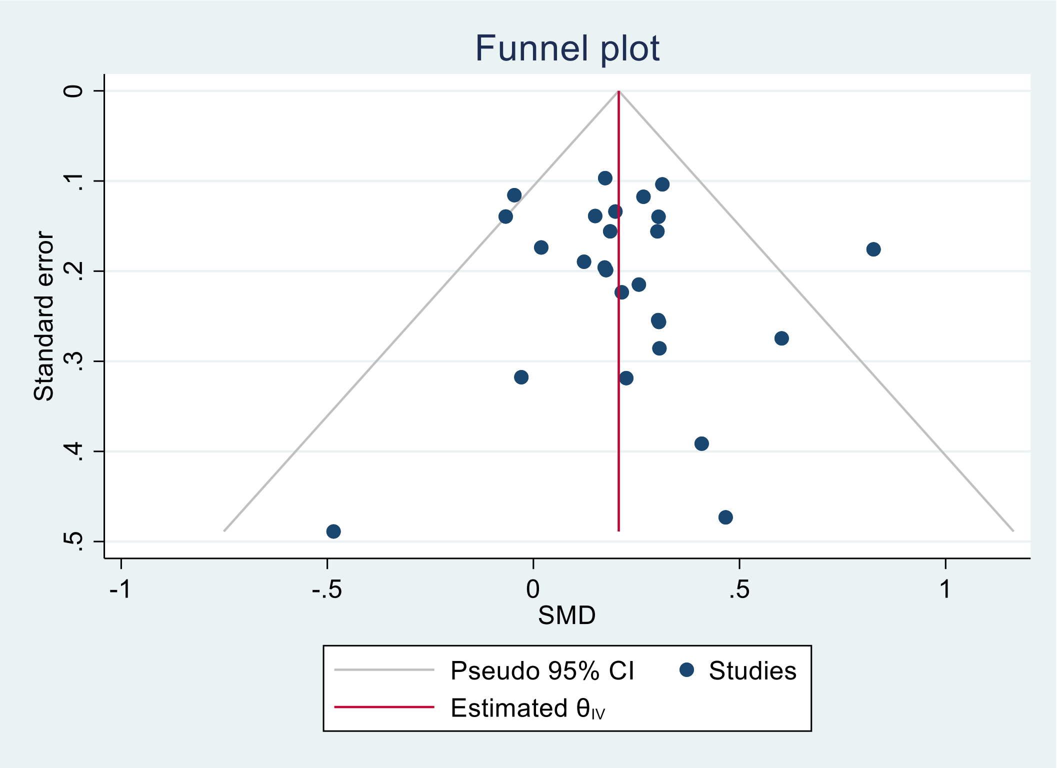

Supplementary Figure 5. Funnel plot for depression at end-of-intervention

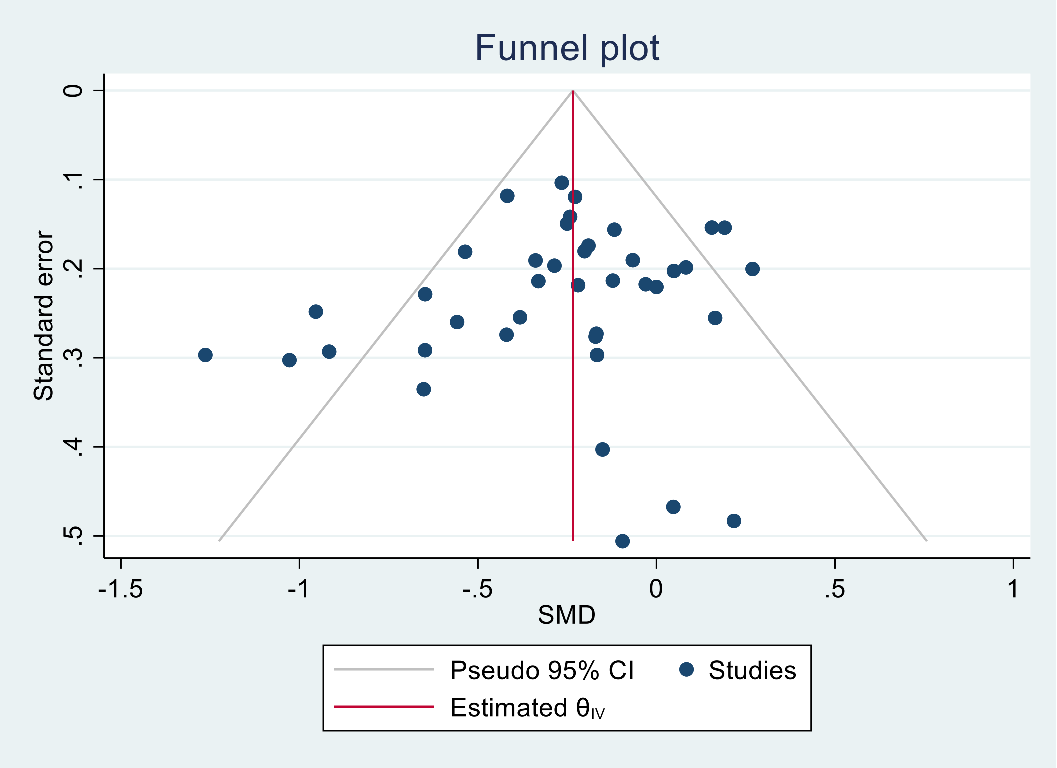

Supplementary Figure 6. Funnel plot for anxiety at end-of-intervention

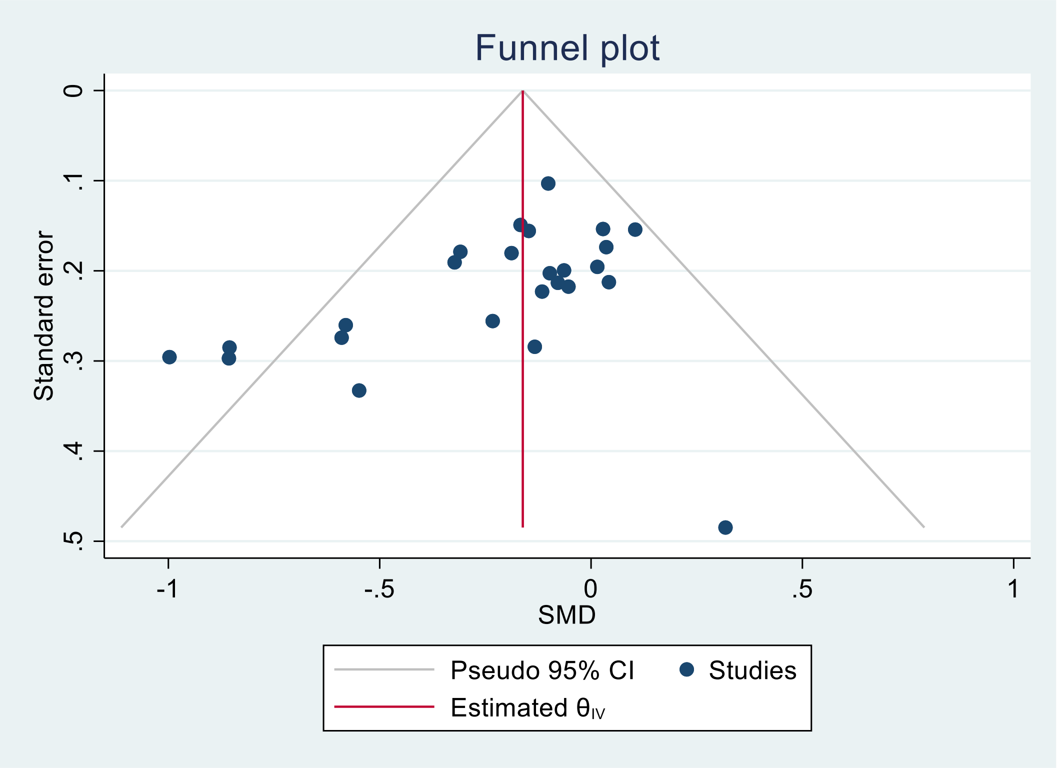

Supplementary Figure 7. Funnel plot for health-related quality of life at end-of-intervention

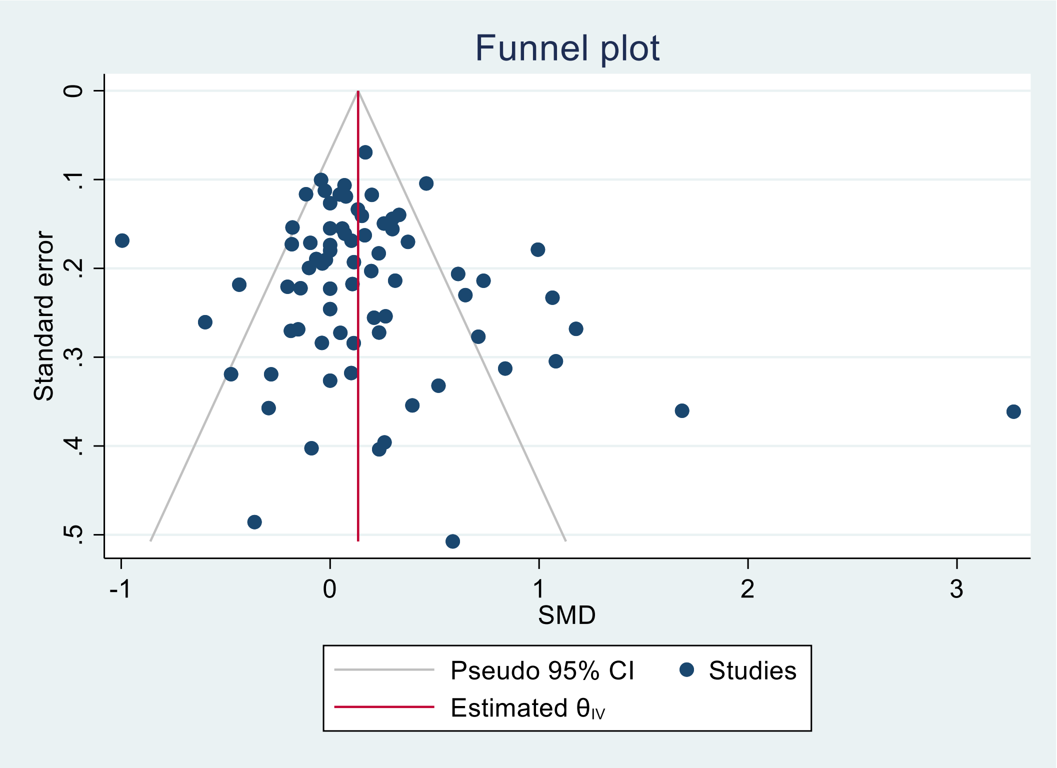

Supplementary Figure 8. Funnel plot for objectively measured physical activity at follow-up

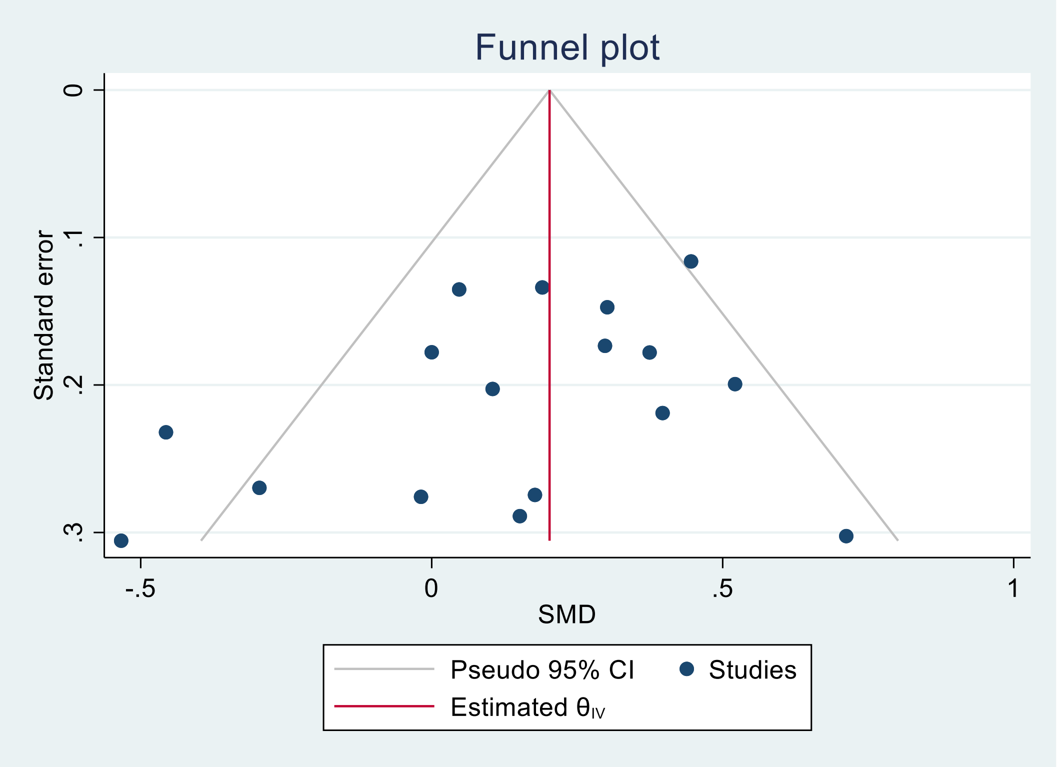

Supplementary Figure 9. Funnel plot for objectively measured physical function at follow-up

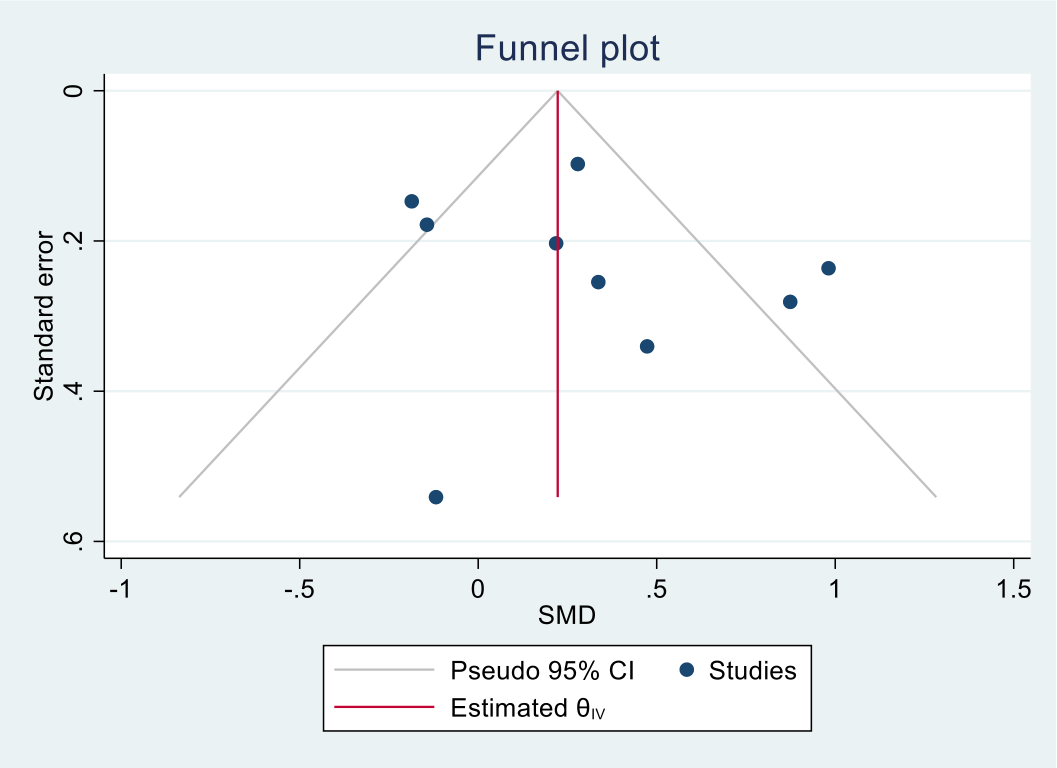

Supplementary Figure 10. Funnel plot for subjectively measured physical activity at follow-up

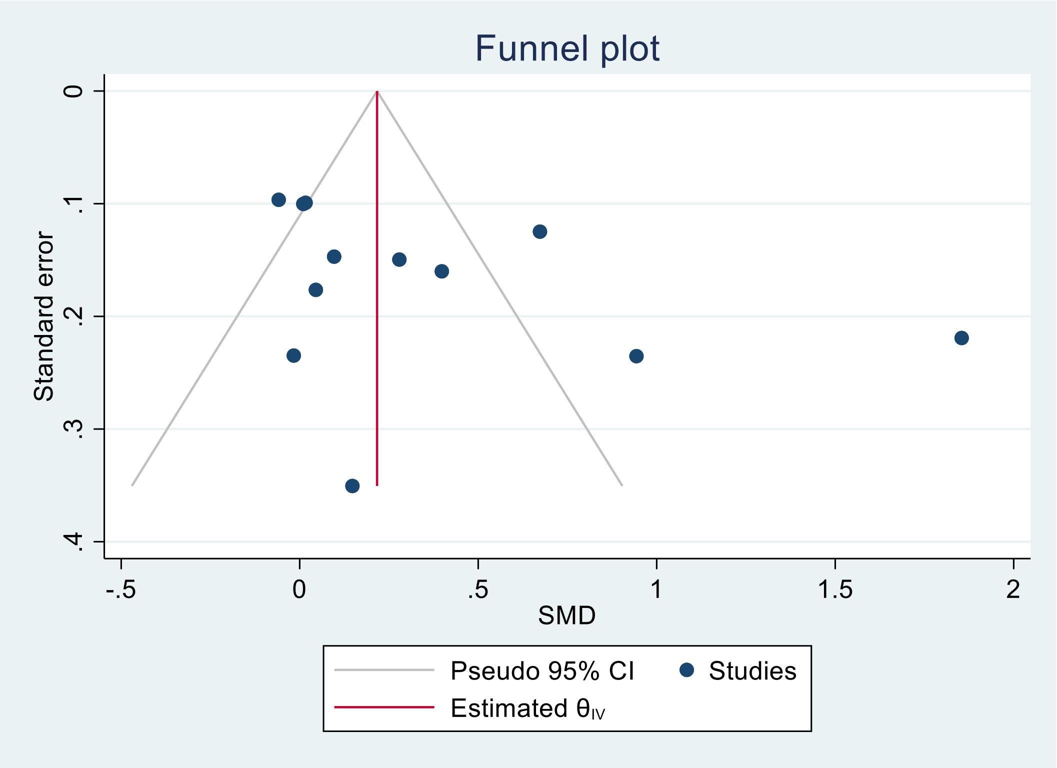

Supplementary Figure 11. Funnel plot for subjectively measured physical function at follow-up

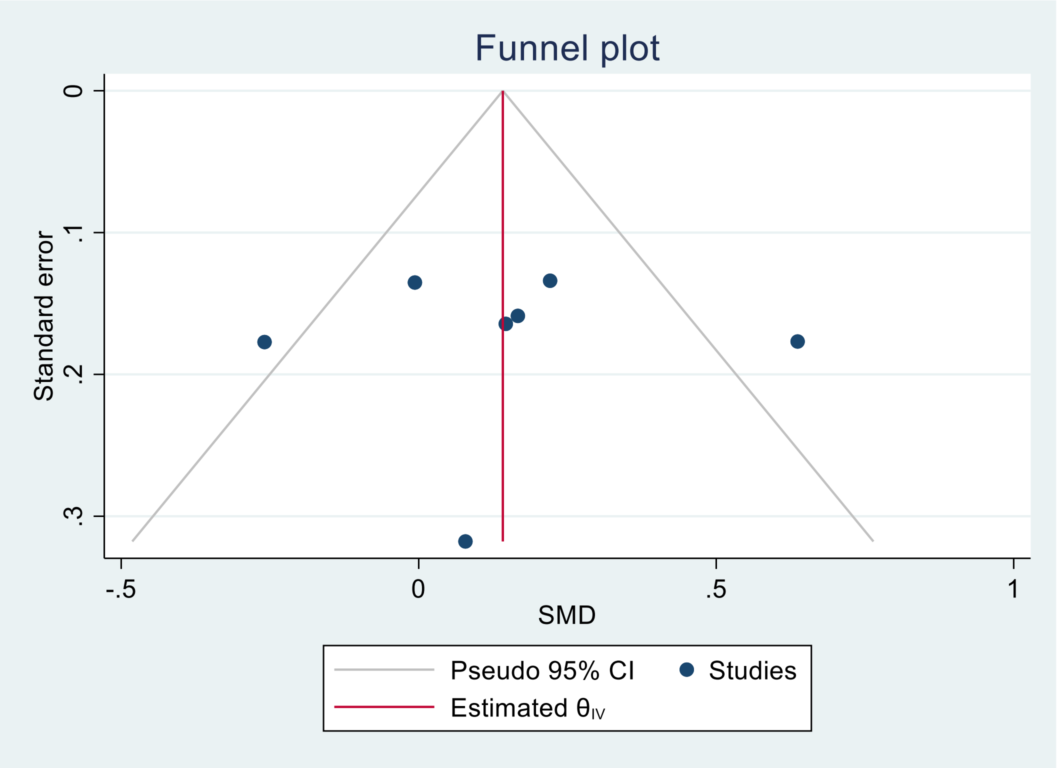

Supplementary Figure 12. Funnel plot for depression at follow-up

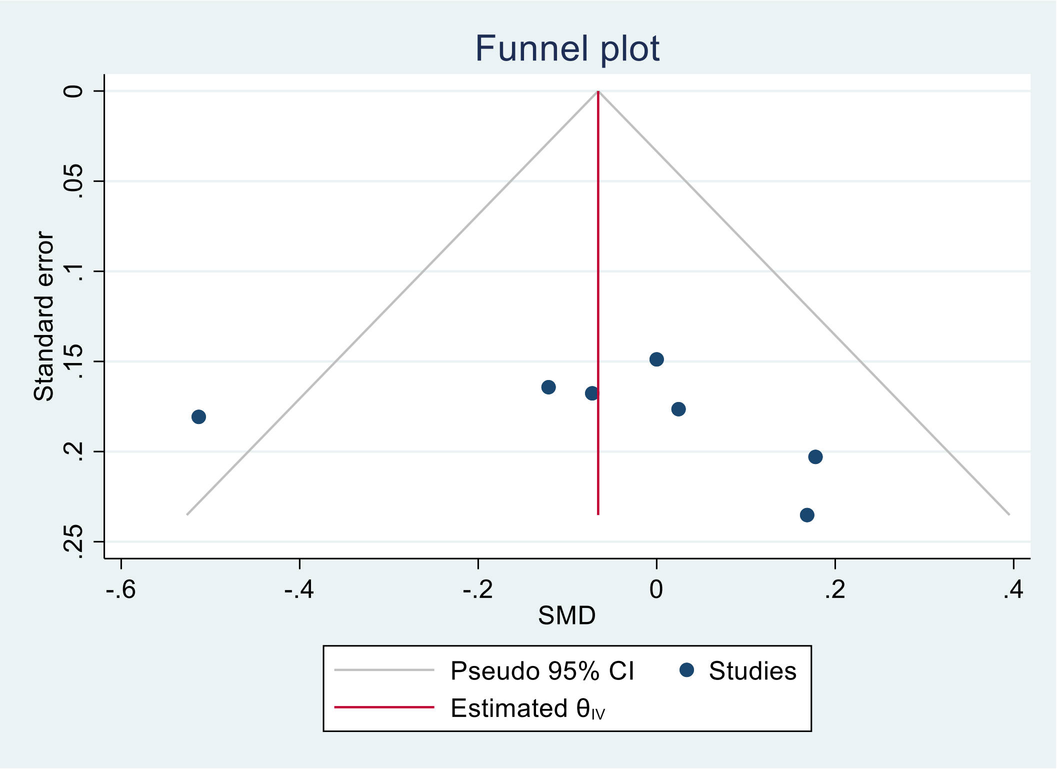

Supplementary Figure 13. Funnel plot for anxiety at follow-up

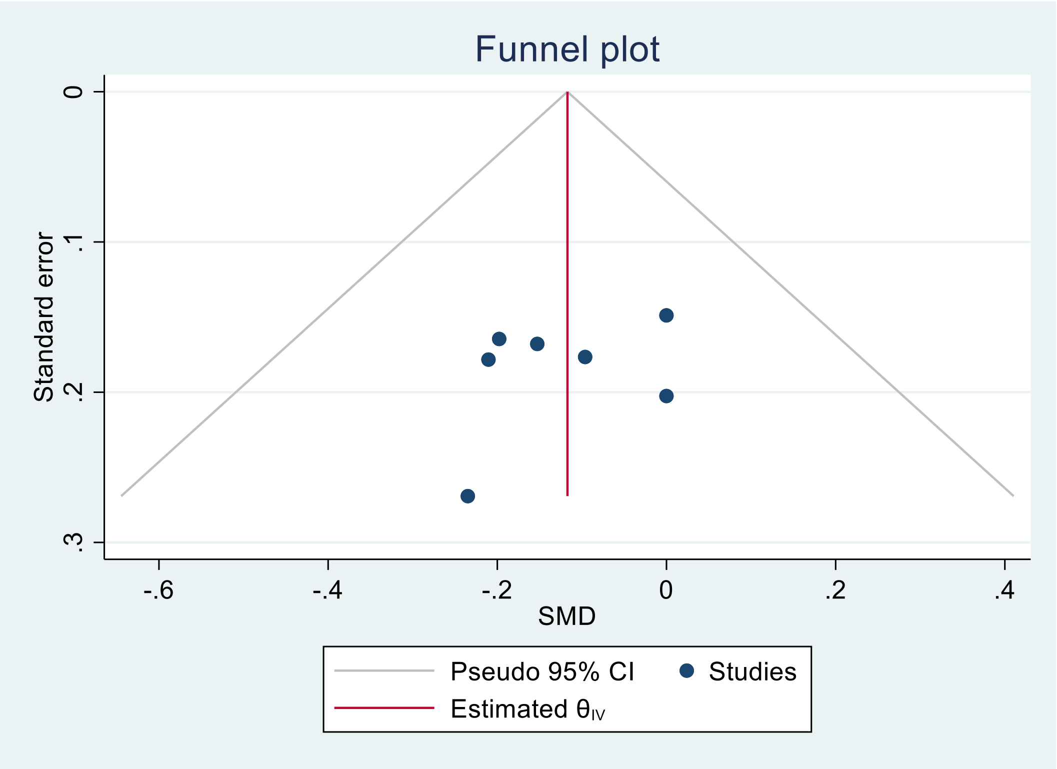

Supplementary Figure 14. Funnel plot for health-related quality of life at follow-up

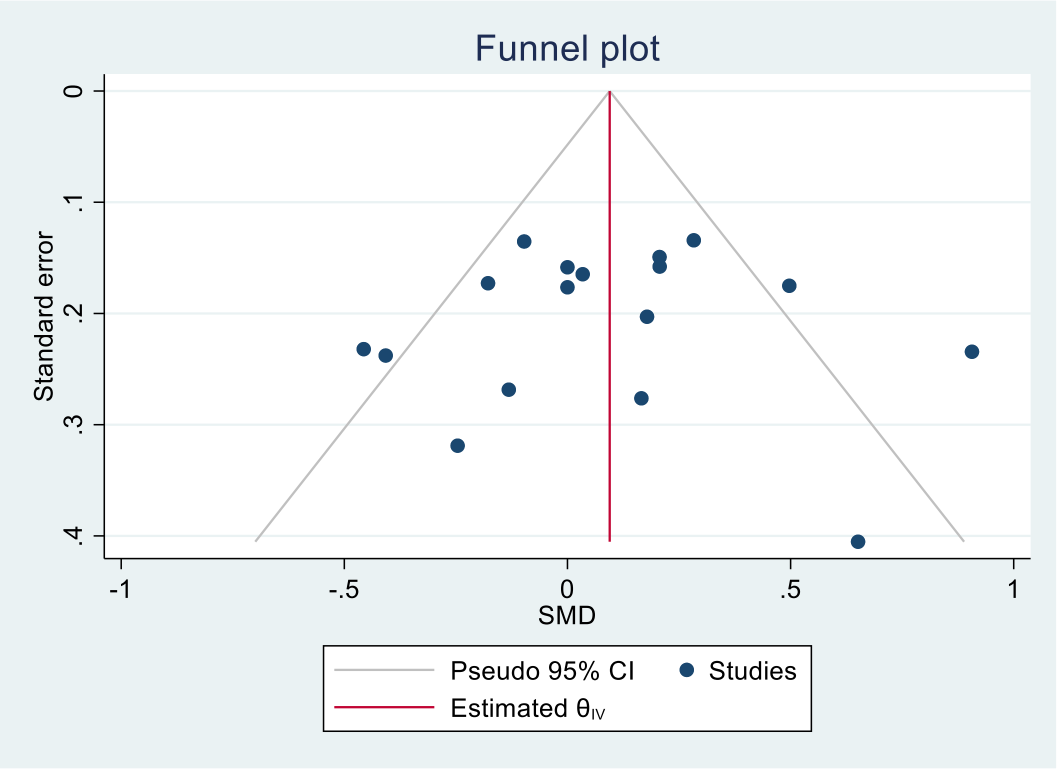

Supplementary Table 1. Results of the Egger's or Harbord's test for small study bias for each outcome

Outcome	Time point	Test	Beta 1	SE beta 1	z-score	Prob>z
Obj PA	End-of-intervention	Egger's	0.23	0.469	0.50	0.616
Subj PA	End-of-intervention	Egger's	2.06	0.65	3.17	0.002
Subj PF	End-of-intervention	Egger's	0.27	0.577	0.46	0.644
DEP	End-of-intervention	Egger's	-0.89	0.643	-1.38	0.168
ANX	End-of-intervention	Egger's	-1.95	0.662	-2.95	0.003
HRQOL	End-of-intervention	Egger's	1.33	0.638	2.08	0.038
NSAE	End-of-intervention	Harbord's	0.10	0.247	0.40	0.689
SAE	End-of-intervention	Harbord's	0.39	0.213	1.82	0.069
Obj PA	Follow-up	Egger's	-1.67	1.093	-1.53	0.127
Obj PF	Follow-up	Egger's	0.79	1.425	0.56	0.578
Subj PA	Follow-up	Egger's	2.38	2.273	1.05	0.295
Subj PF	Follow-up	Egger's	-0.15	2.353	-0.06	0.950
DEP	Follow-up	Egger's	2.8	3.567	0.79	0.432
ANX	Follow-up	Egger's	-1.02	2.373	-0.43	0.667
HRQOL	Follow-up	Egger's	0.09	1.312	0.07	0.947
NSAE	Follow-up	Harbord's	-0.01	0.681	-0.02	0.985
SAE	Follow-up	Harbord's	0.54	0.544	0.99	0.321

SE, Standard error; Obj PA, objectively measured physical activity; Obj PF, objectively measured physical function; Subj PA, subjectively measured physical activity; Subj PF, subjectively measured physical function; DEP depression; ANX anxiety; HRQOL, health-related quality of life
