# Supplementary material for: Benefits and Harms of Digital Health Interventions Promoting Physical Activity in People With Chronic Conditions: Systematic Review and Meta-Analysis
Source: J Med Internet Res. 2023 Jul 6;25:e46439. doi: 10.2196/46439 (PMC10359919; doi:10.2196/46439)
Supplement: Multimedia Appendix 7 [file jmir_v25i1e46439_app7.pdf]

Multimedia Appendix 7: Meta-regression analyses

To manuscript: **Benefits and Harms of Digital Health Interventions Promoting Physical Activity in People with Chronic Conditions: A Systematic Review and Meta-Analysis**

List of meta-regression Tables

Supplementary Table 1. Meta-regression analyses for the participant characteristics variables for the primary outcomes (objectively measured physical activity and physical function) at end-of-intervention
Supplementary Table 2. Meta-regression analyses for the participant characteristics variables for the secondary outcomes (subjectively measured physical activity and physical function) at end-of-intervention
Supplementary Table 3. Meta-regression analyses for the participant characteristics variables for the secondary outcomes (depression, anxiety, and health-related quality of life) at end-of-intervention
Supplementary Table 4. Meta-regression analyses for the intervention characteristics variables for the primary outcomes (objectively measured physical activity and physical function) at end-of-intervention
Supplementary Table 5. Meta-regression analyses for the intervention characteristics variables for the secondary outcomes (subjectively measured physical activity and physical function) at end-of-intervention
Supplementary Table 6. Meta-regression analyses for the intervention characteristics variables for the secondary outcomes (depression, anxiety, and health-related quality of life) at end-of-intervention

Supplementary Table 1. Meta-regression analyses for the participant characteristics variables for the primary outcomes (objectively measured physical activity and physical function) at end-of-intervention

	Objectively measured physical activity						Objectively measured physical function					
Participant characteristics	No	Coefficient	95% CL low	95% CI high	tau2	I2%	No	Coefficient	95% CL low	95% CI high	tau2	I2%
**Mean age**	**49**	**-0.01**	**-0.03**	**-0.00**	**0.03**	**41.27**	46	-0.00	-0.04	0.04	0.67	94.59
**Sex (proportion female)**	59	-0.00	-0.01	0.00	0.04	48.67	45	-0.00	-0.01	0.01	0.70	94.70
**Body-mass-index**	38	0.02	-0.02	0.05	0.05	56.80	35	0.00	-0.12	0.12	0.93	96.23
**Socioeconomic status**	21	-0.00	-0.01	0.00	0.00	0.00	10	0.02	-0.01	0.04	0.51	93.89
**Baseline level of depression**	5	NA	NA	NA	NA	NA	10	0.01	-0.00	0.03	0.01	27.67
**Baseline level of anxiety**	4	NA	NA	NA	NA	NA	8	NA	NA	NA	NA	NA

CI, confidence interval; NA, not applicable; No, Number of comparisons

All analysis are run with random-effects (REML) model; **Bold**, showed a significant difference p<0.05

Supplementary Table 2. Meta-regression analyses for the participant characteristics variables for the secondary outcomes (subjectively measured physical activity and physical function) at end-of-intervention

	Subjectively measured physical activity						Subjectively measured physical function					
Participant characteristics	No	Coefficient	95% CI low	95% CI high	tau2	I2%	No	Coefficient	95% CI low	95% CI high	tau2	I2%
**Mean age**	**57**	**-0.02**	**-0.03**	**-0.00**	**0.11**	**81.73**	25	-0.00	-0.01	0.01	0.01	27.38
**Sex (proportion female)**	58	0.00	-0.00	0.00	0.12	83.38	26	0.00	-0.00	0.01	0.01	15.07
**Body-mass-index**	**43**	**-0.04**	**-0.07**	**-0.01**	**0.10**	**83.50**	18	0.00	-0.02	0.03	0.00	0.00
**Socioeconomic status**	35	0.01	-0.00	0.01	0.08	77.59	20	-0.00	-0.01	0.00	0.01	25.17
**Baseline level of depression**	8	NA	NA	NA	NA	NA	4	NA	NA	NA	NA	NA
**Baseline level of anxiety**	3	NA	NA	NA	NA	NA	0	NA	NA	NA	NA	NA

CI, confidence interval; NA, not applicable; No, Number of comparisons

All analysis are run with random-effects (REML) model; **Bold**, showed a significant difference p<0.05

Supplementary Table 3. Meta-regression analyses for the participant characteristics variables for the secondary outcomes (depression, anxiety, and health-related quality of life) at end-of-intervention

Participant characteristics	Depression						Anxiety						Health-related quality of life					
	**No**	**Coefficient**	**95%CI low**	**95%CI high**	**tau2**	**I2%**	**No**	**Coefficient**	**95%CI low**	**95%CI high**	**tau2**	**I2%**	**No**	**Coefficient**	**95%CI low**	**95%CI high**	**tau2**	**I2%**
**Mean age**	39	0.01	-0.00	0.02	0.03	49.50	24	0.01	-0.00	0.02	0.01	21.20	72	0.00	-0.01	0.02	0.18	85.17
**Sex (proportion female)**	39	-0.00	-0.01	0.00	0.04	50.54	24	-0.00	-0.01	0.00	0.02	30.17	73	-0.00	-0.01	0.00	0.18	84.65
**Body-mass-index**	29	-0.02	-0.05	0.02	0.04	56.30	15	-0.01	-0.05	0.03	0.00	76.32	53	-0.00	-0.04	0.05	0.25	89.27
**Socioeconomic status**	18	0.00	-0.00	0.01	0.02	23043	12	0.00	-0.00	0.01	0.01	25.75	33	0.00	-0.01	0.01	0.16	84.18
**Baseline level of depression**	**14**	**-0.01**	**-0.02**	**-0.00**	**0.05**	**54.02**	7	NA	NA	NA	NA	NA	8	NA	NA	NA	NA	NA
**Baseline level of anxiety**	7	NA	NA	NA	NA	NA	8	NA	NA	NA	NA	NA	7	NA	NA	NA	NA	NA

CI, confidence interval; NA, not applicable; No, Number of comparisons

All analysis are run with random-effects (REML) model; **Bold**, showed a significant difference p<0.05

Supplementary Table 4. Meta-regression analyses for the intervention characteristics variables for the primary outcomes (objectively measured physical activity and physical function) at end-of-intervention

Intervention characteristics	Objectively measured physical activity						Objectively measured physical function					
	No	Coefficient	95% CL low	95% CI high	tau2	I2%	No	Coefficient	95% CL low	95% CI high	tau2	I2%
**Number of digital sessions**	51	-0.00	-0.00	0.00	0.04	45.66	47	-0.00	-0.00	0.00	0.66	94.45
**Number of in-person sessions**	51	-0.03	-0.07	0.01	0.04	46.32	47	-0.00	-0.08	0.08	0.66	94.48
**Intervention frequency**	51	-0.01	-0.03	0.01	0.04	45.93	47	-0.00	-0.09	0.07	0.66	94.09
**Intervention duration**	51	0.00	-0.00	0.01	0.04	46.90	47	-0.00	-0.02	0.01	0.66	94.45
**Type of physical activity**												
Exercise therapy and physical activity	51	-0.07	-0.52	0.37	0.04	48.92	**47**	**1.40**	**0.42**	**2.36**	**0.55**	**93.31**
Physical activity		-0.06	-0.24	0.13	0.04	48.92		**-0.23**	**-0.76**	**0.30**	**0.55**	**93.31**
Cons.: Exercise therapy												
**Intervention phase**												
Phase 2 (After a run-in period or during rehabilitation)	51	0.02	-0.19	0.24	0.04	48.13	47	-0.24	-0.84	0.35	0.66	94.13
Phase 3 (After rehabilitation)		-0.06	-0.27	0.15	0.04	48.13		-0.30	-0.97	0.38	0.66	94.13
Cons.: Phase 1 (without any run-in period or rehabilitation)												
**Use of theory or framework**	50	-0.07	-0.24	0.10	0.04	46.85	46	-0.27	-0.84	0.29	0.66	94.39
**Adherence digital and in-person sessions**	14	0.00	-0.00	0.01	0.03	40.11	21	0.01	-0.01	0.03	1.29	97.19
**Adherence digital sessions**	16	0.00	-0.00	0.01	0.06	58.91	16	0.00	-0.01	0.01	0.04	57.77
**Digital only approach**	51	0.06	-0.11	0.23	0.04	47.12	47	-0.13	-0.62	0.02	0.66	94.26
**Other non-digital adjuvant intervention**	51	-0.11	-0.28	0.06	0.03	44.63	47	0.40	-0.12	0.91	0.63	93.94
**Financial incentive**	51	0.08	-0.31	0.47	0.03	45.80	47	-0.31	-1.47	0.85	0.66	94.26

CI, confidence interval; Cons, Constant; NA, not applicable; No, Number of comparisons
All analysis are run with random-effects (REML) model; **Bold**, showed a significant difference p<0.05

Supplementary Table 5. Meta-regression analyses for the intervention characteristics variables for the secondary outcomes (subjectively measured physical activity and physical function) at end-of-intervention

Intervention characteristics	Subjectively measured physical activity						Subjectively measured physical function					
	No	Coefficient	95% CL low	95% CI high	tau2	I2%	No	Coefficient	95% CL low	95% CI high	tau2	I2%
**Number of digital sessions**	58	-0.00	-0.00	0.00	0.12	83.08	26	0.00	-0.00	0.00	0.01	26.67
**Number of in-person sessions**	58	0.02	-0.01	0.06	0.12	82.66	26	-0.00	-0.04	0.03	0.01	25.92
**Intervention frequency**	58	0.01	-0.02	0.04	0.12	83.35	26	0.00	-0.01	0.02	0.01	25.02
**Intervention duration**	**58**	**-0.01**	**-0.01**	**-0.00**	**0.11**	**81.86**	26	-0.00	-0.01	0.01	0.01	26.73
**Type of physical activity**												
Exercise therapy and physical activity	55	0.10	-0.45	0.70	0.12	82.88	26	-0.09	-0.73	0.56	0.00	9.89
Physical activity		0.15	-0.11	0.41	0.12	82.88	26	-0.17	-0.31	-0.02	0.00	9.89
Cons: Exercise therapy												
**Intervention phase**												
Phase 2 (After a run-in period or during rehabilitation)	58	0.01	-0.36	0.39	0.13	83.75	26	-0.16	-0.43	0.11	0.01	15.51
Phase 3 (After rehabilitation)	58	-0.12	-0.48	0.24	0.13	83.75	26	0.21	-0.06	0.48	0.01	15.51
Cons.: Phase 1 (without any run-in period or rehabilitation)												
**Use of theory or framework**	**58**	**-0.31**	**-0.50**	**-0.11**	**0.09**	**79.87**	26	-0.08	-0.28	0.12	0.01	25.88
**Adherence digital and in-person sessions**	20	0.01	-0.00	0.01	0.02	40.11	10	0.00	-0.01	0.01	0.04	59.48
**Adherence digital sessions**	22	0.00	-0.00	0.01	0.01	23.36	13	0.00	-0.01	0.01	0.02	49.30
**Digital only approach**	58	-0.01	-0.23	0.20	0.12	83.38	26	0.04	-0.17	0.25	0.01	25.56
**Other non-digital adjuvant intervention**	58	0.01	-0.22	0.23	0.12	83.43	26	-0.00	-0.21	0.20	0.01	25.91
**Financial incentive**	58	-0.12	-0.39	0.14	0.12	83.12	26	0.01	-0.22	0.24	0.01	25.95

CI, confidence interval; Cons, Constant; NA, not applicable; No, Number of comparisons

All analysis are run with random-effects (REML) model; **Bold**, showed a significant difference p<0.05

Supplementary Table 6. Meta-regression analyses for the intervention characteristics variables for the secondary outcomes (depression, anxiety, and health-related quality of life) at end-of-intervention

Intervention characteristics	Depression						Anxiety						Health-related quality of life					
	No	Coefficient	95%CL low	95%CI high	tau2	I2%	No	Coefficient	95%CL low	95%CI high	tau2	I2%	No	Coefficient	95%CL low	95%CI high	tau2	I2%
**Number of digital sessions**	40	-0.00	-0.00	0.00	0.04	54.36	25	-0.00	-0.00	0.00	0.01	26.25	74	-0.00	-0.00	0.00	0.17	84.47
**Number of in-person sessions**	40	0.01	-0.02	0.03	0.05	54.83	25	0.02	-0.01	0.05	0.01	21.27	74	-0.00	-0.04	0.34	0.17	84.55
**Intervention frequency**	40	-0.01	-0.03	0.01	0.05	53.89	25	-0.00	-0.02	0.02	0.02	28.04	74	-0.01	-0.03	0.03	0.18	84.43
**Intervention duration**	40	0.00	-0.00	0.01	0.05	55.03	25	-0.00	-0.01	0.01	0.01	23.34	**74**	**-0.01**	**-0.01**	**-5.81**	**0.16**	**83.47**
**Type of physical activity**																		
Exercise therapy and physical activity	40	NA	NA	NA	NA	NA	25	NA	NA	NA	NA	NA	74	0.12	-0.43	0.67	0.16	83.40
Physical activity	40	NA	NA	NA	NA	NA	25	NA	NA	NA	NA	NA	74	-0.23	-0.44	-0.01	0.16	83.40
Cons.: Exercise therapy																		
**Intervention phase**																		
Phase 2 (After a run-in period or during rehabilitation)	40	0.24	0.01	0.48	0.04	48.51	25	0.22	0.01	0.43	0.01	17.64	74	-0.02	-0.30	0.26	0.17	83.76
Phase 3 (After rehabilitation)	40	0.19	-0.05	0.46	0.04	48.51	25	0.18	-0.05	0.40	0.01	17.64	74	-0.23	-0.50	0.04	0.17	83.76
Cons.: Phase 1 (without any run-in period or rehabilitation)																		
**Use of theoretical framework**	39	-0.17	-0.35	0.01	0.04	46.69	24	-0.02	-0.22	0.16	0.01	27.49	73	0.01	-0.08	0.23	0.17	84.19
**Adherence digital and in-person sessions**	13	0.00	-0.00	0.01	0.00	0.07	6	NA	NA	NA	NA	NA	26	0.01	-0.01	0.02	0.08	71.39
**Adherence digital sessions**	15	0.00	-0.01	0.01	0.05	52.66	7	NA	NA	NA	NA	NA	30	0.01	-0.01	0.02	0.25	89.82
**Digital only approach**	40	-0.11	-0.30	0.09	0.05	53.64	25	-0.04	-0.23	0.15	0.01	24.34	74	-0.06	-0.29	0.16	0.17	84.40
**Other non-digital adjuvant intervention**	40	-0.01	-0.22	0.20	0.05	54.65	25	0.15	-0.09	0.39	0.01	26.02	74	0.20	-0.05	0.43	0.17	84.01
**Financial incentive**	40	0.01	-0.36	0.37	0.04	55.17	25	0.06	-0.43	0.56	0.01	24.23	74	-0.03	-0.46	0.39	0.18	84.51

CI, confidence interval; Cons, Constant; NA, not applicable; No, Number of comparisons
All analysis are run with random-effects (REML) model; **Bold**, showed a significant difference $p<0.05$
