# Supplementary material for: Benefits and Harms of Digital Health Interventions Promoting Physical Activity in People With Chronic Conditions: Systematic Review and Meta-Analysis
Source: J Med Internet Res. 2023 Jul 6;25:e46439. doi: 10.2196/46439 (PMC10359919; doi:10.2196/46439)

Multimedia Appendix 8: Sub-group and sensitivity analyses

To manuscript: **Benefits and Harms of Digital Health Interventions Promoting Physical Activity in People with Chronic Conditions: A Systematic Review and Meta-Analysis**

List of sensitivity analyses Tables and sub-group analyses forest plots Figures

Introduction to the Forest plots

Supplementary Table 1. Summary table of sensitivity analyses of studies with a digital only approach for the primary outcomes at end-of-intervention and follow-up

Supplementary Figure 1. Forest plot for objectively measured physical activity at end-of-intervention sub-grouped by primary digital health intervention delivery methods

Supplementary Figure 2. Forest plot for objectively measured physical function at end-of-intervention sub-grouped by primary digital health intervention delivery methods

Supplementary Figure 3. Forest plot for objectively measured physical activity at follow-up sub-grouped by primary digital health intervention delivery methods

Supplementary Figure 4. Forest plot for objectively measured physical function at follow-up sub-grouped by primary digital health intervention delivery methods

Supplementary Figure 5. Forest plot for objectively measured physical activity at end-of-intervention sub-grouped by the methodological quality

Supplementary Figure 6. Forest plot for objectively measured physical function at end-of-intervention sub-grouped by the methodological quality

Supplementary Figure 7. Forest plot for subjectively measured physical activity at end-of-intervention sub-grouped by the methodological quality

Supplementary Figure 8. Forest plot for subjectively measured physical function at end-of-intervention sub-grouped by the methodological quality

Supplementary Figure 9. Forest plot for depression at end-of-intervention sub-grouped by the methodological quality

Supplementary Figure 10. Forest plot for anxiety at end-of-intervention sub-grouped by the methodological quality

Supplementary Figure 11. Forest plot for health-related quality of life at end-of-intervention sub-grouped by the methodological quality

Introduction to the Forest plots

In the following, we have presented a Forest plot for each outcome at end-of-intervention and follow-up. Primary outcomes are objectively measured physical activity and physical function. Secondary outcomes are subjectively measured physical activity and physical function, depression, anxiety, health-related quality of life, and non-serious and serious adverse events.

Each Forest plot summarizes the results of the included studies for the individual outcome, sub-grouped under the included chronic conditions (depression or anxiety (mental health condition), ischemic heart disease or heart failure (Heart disease), chronic obstructive pulmonary disease, knee or hip osteoarthritis (Osteoarthritis), hypertension, type 2 diabetes or multimorbidity (two or more of the conditions)).
The effect size and 95% confidence intervals are displayed for each study (blue square with lines) and overall for each subgroup (red diamond). The effect size in each study is calculated as a standardized mean difference (SMD) to allow for interpretation and pooling across different measurements tools and further given a weight based on the precision of the estimate. Studies with narrower 95% confidence intervals and more precise results get weighted higher. The forest plot also shows the overall effect of the summary estimate across all subgroups and is represented by the green line and green diamond, whereas the grey line represents the null line if there were no effect of the digital health interventions.

Abbreviations:
High, high risk of bias assessed by the Cochrane Risk of Bias 2
Low, low risk of bias assessed by the Cochrane Risk of Bias 2
N, numbers
SD, Standard deviation
SMD, Standardized mean difference
Some concerns, Some concerns of risk of bias assessed by the Cochrane Risk of Bias 2
95% CI, 95% confidence interval

Supplementary Table 1. Summary table of sensitivity analyses of studies with a digital only approach for the primary outcomes at end-of-intervention and follow-up

Primary outcomes	Time point	Number of comparisons	SMD, steps or meters	Effect	95% CI low	95% CI high	I^2
Obj PA	End-of-intervention	29	SMD	0.31	0.23	0.38	0.93%
Obj MVPA	End-of-intervention	7	SMD	0.28	-0.12	0.67	54.35%
Daily steps	End-of-intervention	19	Steps	1012.07	624.15	1400.00	58.45%
Obj PF	End-of-intervention	22	SMD	0.29	0.05	0.54	84.94%
6MWT	End-of-intervention	15	Meters	16.27	2.16	30.38	53.44%
Obj PA	Follow-up	8	SMD	0.14	-0.05	0.32	48.34%
Obj MVPA	Follow-up	NA	NA	NA	NA	NA	NA
Daily steps	Follow-up	6	Steps	483.57	-69.99	1037.12	71.17%
Obj PF	Follow-up	4	SMD	0.12	-0.21	0.45	62.01%
6MWT	Follow-up	2	Meters	28.89	-3.06	60.84	16.12%

CI, Confidence interval; I2, I-squared statistics; NA Not applicable; Obj MVPA, objectively measured moderate-to-vigorous physical activity; Obj PA, objectively measured physical activity; Obj PF, objectively measured physical function; SMD, standardised mean difference; 6MWT, six-minute walk test
All meta-analyses were done with random effects REML model

Supplementary Figure 1. Forest plot for objectively measured physical activity at end-of-intervention sub-grouped by primary digital health intervention delivery methods

Study	Treatment N	Treatment Mean	Treatment SD	Control N	Control Mean	Control SD	SMD with 95% CI	Weight (%)
**Device**								
Dyson et al. 2010	21	1266.0	3212.5	18	-712.0	3212.5	0.60 [-0.03, 1.23]	1.24
Plotnikoff et al. 2013	62	20821.0	7283.5	39	20097.0	7339.2	0.10 [-0.30, 0.50]	2.28
Moy et al. 2015	133	3922.0	2491.0	68	3200.0	2489.0	0.29 [-0.00, 0.58]	3.06
van der Weegen et al. 2015	65	48.2	23.8	68	39.6	19.5	0.39 [0.05, 0.73]	2.67
Chokshi et al. 2017	49	8705.0	3107.0	55	6841.0	3254.0	0.58 [0.19, 0.97]	2.33
Li et al. 2018	30	29.2	32.9	31	10.8	19.2	0.68 [0.17, 1.19]	1.68
Wootton et al. 2018 + 2019	23	4526.0	2527.4	20	5241.0	2276.3	-0.29 [-0.88, 0.30]	1.36
Li et al. 2020	26	7133.3	3603.3	25	6232.7	3086.1	0.26 [-0.28, 0.81]	1.54
Heterogeneity: $\tau^2 = 0.01$, $I^2 = 12.28\%$, $H^2 = 1.14$							0.34 [0.17, 0.50]	
Test of $\theta_i = \theta_j$: Q(7) = 9.87, p = 0.20								
Test of $\theta = 0$: z = 4.09, p = 0.00								
**eHealth**								
Reid et al. 2012	115	7079.0	3159.0	108	6168.0	3159.0	0.29 [0.02, 0.55]	3.31
Bossen et al. 2013	27	361.0	131.2	30	358.0	135.5	0.02 [-0.49, 0.53]	1.67
Chaplin et al. 2016 + 2022	20	6111.7	2464.2	34	5409.4	3377.7	0.23 [-0.32, 0.77]	1.53
Tsai et al. 2017	19	3176.0	2004.0	17	2878.0	2852.0	0.12 [-0.52, 0.76]	1.21
Wan et al. 2017	57	804.0	3954.6	52	0.0	3954.6	0.20 [-0.17, 0.58]	2.43
Connelly et al. 2017	11	30058.0	16116.0	5	34597.0	17675.0	-0.26 [-1.26, 0.74]	0.57
Connelly et al. 2017	10	37083.0	25387.0	5	34597.0	17675.0	0.10 [-0.91, 1.11]	0.57
Akinci et al. 2018	21	7310.3	914.3	22	6535.9	1869.8	0.51 [-0.08, 1.11]	1.35
Lambert et al. 2018	13	97.6	86.4	11	13.0	97.3	0.89 [0.08, 1.71]	0.82
Poppe et al. 2019	26	241.9	47.9	17	267.2	83.1	-0.39 [-0.99, 0.22]	1.32
Young et al. + Drew et al. 2021 + 2022a + 2022b	62	0.8	2.2	63	-0.1	2.2	0.41 [0.06, 0.76]	2.59
Heterogeneity: $\tau^2 = 0.00$, $I^2 = 0.00\%$, $H^2 = 1.00$							0.24 [0.10, 0.38]	
Test of $\theta_i = \theta_j$: Q(10) = 10.32, p = 0.41								
Test of $\theta = 0$: z = 3.27, p = 0.00								
**eHealth + Device**								
Avila et al. 2018 + 2019	24	6469.0	4090.8	26	6408.0	2231.2	0.02 [-0.53, 0.56]	1.53
Robinson et al. 2021	75	672.9	3399.0	78	-639.4	3596.7	0.37 [0.05, 0.69]	2.85
Cox et al. 2022	47	-1.0	25.5	49	-3.0	36.6	0.06 [-0.33, 0.46]	2.28
Heterogeneity: $\tau^2 = 0.01$, $I^2 = 14.11\%$, $H^2 = 1.16$							0.20 [-0.05, 0.45]	
Test of $\theta_i = \theta_j$: Q(2) = 2.01, p = 0.37								
Test of $\theta = 0$: z = 1.60, p = 0.11								
**mHealth**								
De Greef et al. 2011	60	323.0	87.0	32	306.0	102.0	0.18 [-0.24, 0.61]	2.11
Piette et al. 2011	145	4499.0	2612.0	146	3314.0	2516.0	0.46 [0.23, 0.69]	3.59
Plotnikoff et al. 2013	68	21493.0	7289.7	78	20097.0	7339.2	0.19 [-0.13, 0.51]	2.80
Eakin et al. 2014	151	1.4	3.7	151	0.0	3.7	0.38 [0.15, 0.61]	3.64
Agboola et al. 2016	64	14180.0	55038.5	62	8220.0	41592.6	0.12 [-0.23, 0.47]	2.62
Horton et al. 2018 + 2021	26	1074.0	971.8	25	-464.0	2376.6	0.84 [0.28, 1.40]	1.46
Alonso-Dominguez et al. 2019	94	10714.0	4550.0	91	8464.0	4731.0	0.48 [0.19, 0.77]	3.07
Bartholdy et al. 2019	19	-5.6	64.6	19	14.8	64.6	-0.31 [-0.94, 0.32]	1.25
Hochsmann et al. 2019	18	9783.0	1334.0	17	6552.0	1280.0	2.41 [1.55, 3.27]	0.75
Bailey et al. 2020	9	7109.3	2986.7	9	7492.2	2769.1	-0.13 [-1.01, 0.75]	0.72
Andrade et al. 2021	11	6873.0	2819.0	12	6541.0	2225.0	0.13 [-0.66, 0.92]	0.87
Chan et al. 2022	23	10206.0	5534.0	21	8068.0	4994.8	0.40 [-0.19, 0.98]	1.38
Spielmanns et al. 2022	33	5016.3	5397.2	34	3105.1	2789.3	0.44 [-0.04, 0.92]	1.82
Heterogeneity: $\tau^2 = 0.14$, $I^2 = 77.71\%$, $H^2 = 4.49$							0.39 [0.15, 0.64]	
Test of $\theta_i = \theta_j$: Q(12) = 35.64, p = 0.00								
Test of $\theta = 0$: z = 3.14, p = 0.00								
**mHealth + Device**								
Guiraud et al. 2012	19	543.7	144.1	10	266.7	107.4	2.02 [1.12, 2.93]	0.68
Hornikx et al. 2015	12	984.0	1208.0	15	1013.0	1275.0	-0.02 [-0.76, 0.71]	0.97
Bender et al. 2017	22	7483.0	2415.0	23	6735.0	2363.0	0.31 [-0.27, 0.89]	1.41
Demeyer et al. 2017	140	4767.0	3606.7	140	4059.0	2746.7	0.22 [-0.01, 0.45]	3.57
Al-Ghafri et al. 2018	33	7034.0	2408.0	36	6278.0	1270.0	0.39 [-0.08, 0.86]	1.86
Bentley et al. 2020	19	4258.0	2509.1	11	5124.9	2615.2	-0.33 [-1.06, 0.40]	0.99
Benzo et al. 2021	56	-344.8	1657.7	63	-975.8	2596.3	0.28 [-0.08, 0.64]	2.54
Coombes et al. 2021	12	484.6	216.3	13	441.2	342.6	0.15 [-0.61, 0.90]	0.92
Radhakrishnan et al. 2021	19	2887.0	2821.0	19	2541.0	1604.0	0.15 [-0.48, 0.77]	1.26
Benzo et al. 2022	188	7796.1	3337.5	187	7047.5	3248.7	0.23 [0.02, 0.43]	3.86
Cerdan-De-las-heras et al. 2022	27	8999.8	4241.7	27	11984.6	10783.0	-0.36 [-0.89, 0.17]	1.59
Felker et al. 2022	74	3752.0	2902.8	78	2919.6	2167.1	0.32 [0.01, 0.64]	2.84
Heterogeneity: $\tau^2 = 0.00$, $I^2 = 0.00\%$, $H^2 = 1.00$							0.23 [0.12, 0.34]	
Test of $\theta_i = \theta_j$: Q(11) = 23.54, p = 0.01								
Test of $\theta = 0$: z = 4.10, p = 0.00								
**mHealth + eHealth**								
Vasilopoulou et al. 2014	47	349.9	167.6	50	172.5	192.5	0.97 [0.55, 1.39]	2.15
Heterogeneity: $\tau^2 = 0.00$, $I^2 = .\%$, $H^2 = .$							0.97 [0.55, 1.39]	
Test of $\theta_i = \theta_j$: Q(0) = -0.00, p = .								
Test of $\theta = 0$: z = 4.56, p = 0.00								
**mHealth + eHealth + Device**								
Frederix et al. 2015	69	8233.0	5501.0	70	5265.0	5541.4	0.53 [0.20, 0.87]	2.70
Reid et al. 2021	68	225.2	163.0	67	216.9	179.8	0.05 [-0.29, 0.38]	2.71
Reid et al. 2021	158	284.5	205.3	156	284.8	202.3	-0.00 [-0.22, 0.22]	3.69
Heterogeneity: $\tau^2 = 0.06$, $I^2 = 72.88\%$, $H^2 = 3.69$							0.18 [-0.15, 0.51]	
Test of $\theta_i = \theta_j$: Q(2) = 7.14, p = 0.03								
Test of $\theta = 0$: z = 1.08, p = 0.28								
**Overall**							0.29 [0.21, 0.37]	
Heterogeneity: $\tau^2 = 0.03$, $I^2 = 44.69\%$, $H^2 = 1.81$								
Test of $\theta_i = \theta_j$: Q(50) = 107.04, p = 0.00								
Test of $\theta = 0$: z = 7.09, p = 0.00								
Test of group differences: $Q_b(6) = 13.54$, p = 0.04								

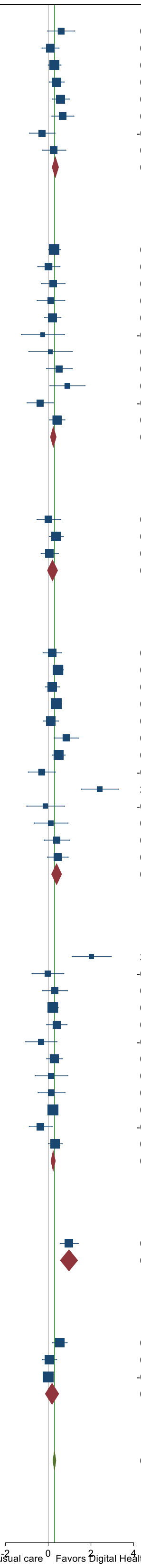

Supplementary Figure 2. Forest plot for objectively measured physical function at end-of-intervention sub-grouped by primary digital health intervention delivery methods

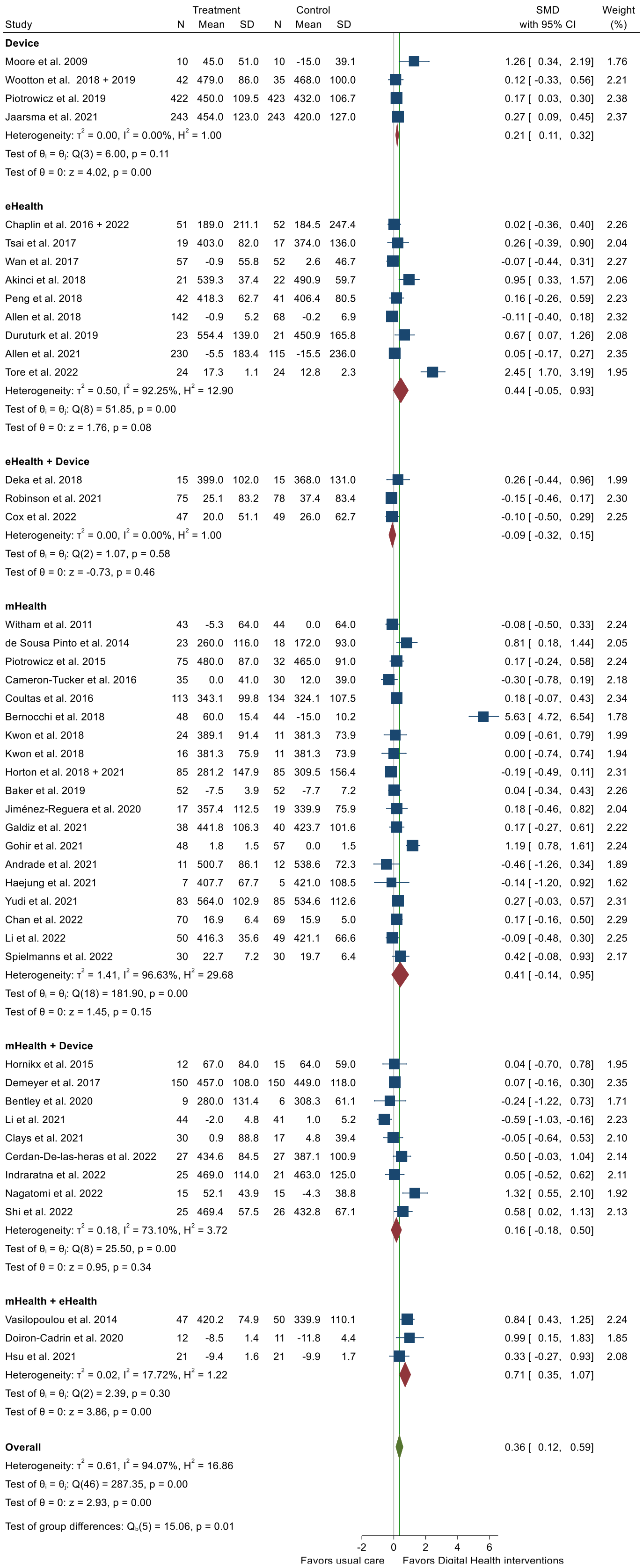

Supplementary Figure 3. Forest plot for objectively measured physical activity at follow-up sub-grouped by primary digital health intervention delivery methods

Study	Treatment N	Treatment Mean	Treatment SD	Control N	Control Mean	Control SD	SMD with 95% CI	Weight (%)
**Device**								
van der Weegen et al. 2015	65	48.8	23.8	68	42.4	18.9	0.30 [-0.04, 0.64]	6.89
Moy et al. 2016	154	270.0	2236.2	84	163.0	2297.1	0.05 [-0.22, 0.31]	8.36
Chokshi et al. 2017	48	8271.0	3003.0	55	6669.0	3091.0	0.52 [0.13, 0.91]	6.02
Wootton et al. 2018 + 2019	23	-757.0	1392.8	20	0.0	1392.8	-0.53 [-1.13, 0.07]	3.54
Heterogeneity: τ^2 = 0.11, I^2 = 74.96%, H^2 = 3.99							0.13 [-0.25, 0.50]	
Test of $\theta_i = \theta_j$: Q(3) = 9.76, p = 0.02								
Test of θ = 0: z = 0.66, p = 0.51								
**eHealth**								
Reid et al. 2012	115	7392.0	3365.0	108	6750.0	3366.0	0.19 [-0.07, 0.45]	8.42
Bossen et al. 2013	24	361.0	123.7	28	338.0	130.9	0.18 [-0.36, 0.72]	4.11
Wan et al. 2020	35	-104.0	225.5	40	0.0	225.5	-0.46 [-0.91, -0.00]	5.08
Young et al. + Drew et al. 2021 + 2022a + 2022b	62	0.5	2.2	63	0.5	2.2	0.00 [-0.35, 0.35]	6.74
Heterogeneity: τ^2 = 0.04, I^2 = 51.40%, H^2 = 2.06							-0.00 [-0.28, 0.27]	
Test of $\theta_i = \theta_j$: Q(3) = 6.14, p = 0.11								
Test of θ = 0: z = -0.00, p = 1.00								
**eHealth + Device**								
Avila et al. 2019	21	8002.0	7998.0	26	6948.0	5751.0	0.15 [-0.41, 0.72]	3.83
Cox et al. 2022	47	-2.0	37.5	49	-6.0	38.3	0.10 [-0.29, 0.50]	5.92
Heterogeneity: τ^2 = 0.00, I^2 = 0.00%, H^2 = 1.00							0.12 [-0.21, 0.45]	
Test of $\theta_i = \theta_j$: Q(1) = 0.02, p = 0.89								
Test of θ = 0: z = 0.72, p = 0.47								
**mHealth**								
De Greef et al. 2011	60	311.0	88.0	32	273.0	107.0	0.40 [-0.03, 0.83]	5.44
Eakin et al. 2014	151	1.4	3.2	151	0.0	3.2	0.45 [0.22, 0.67]	9.16
Horton et al. 2018 + 2021	26	-309.0	1783.8	25	-275.0	1876.3	-0.02 [-0.56, 0.52]	4.08
Alonso-Dominguez et al. 2019	94	10253.0	4781.0	91	8717.0	5353.0	0.30 [0.01, 0.59]	7.88
Heterogeneity: τ^2 = 0.00, I^2 = 0.00%, H^2 = 1.00							0.36 [0.20, 0.51]	
Test of $\theta_i = \theta_j$: Q(3) = 2.61, p = 0.46								
Test of θ = 0: z = 4.42, p = 0.00								
**mHealth + Device**								
Bender et al. 2017	22	9524.0	3626.0	23	7208.0	2719.0	0.71 [0.12, 1.31]	3.59
Cerdan-De-las-heras et al. 2022	27	7660.8	3986.3	27	9384.0	7068.4	-0.30 [-0.82, 0.23]	4.21
Felker et al. 2022	63	3679.4	2926.3	64	2755.5	1872.3	0.37 [0.03, 0.72]	6.73
Heterogeneity: τ^2 = 0.17, I^2 = 73.79%, H^2 = 3.81							0.26 [-0.29, 0.81]	
Test of $\theta_i = \theta_j$: Q(2) = 6.88, p = 0.03								
Test of θ = 0: z = 0.94, p = 0.35								
**Overall**							0.17 [0.04, 0.31]	
Heterogeneity: τ^2 = 0.04, I^2 = 50.85%, H^2 = 2.03								
Test of $\theta_i = \theta_j$: Q(16) = 33.07, p = 0.01								
Test of θ = 0: z = 2.55, p = 0.01								
Test of group differences: $Q_b(4)$ = 5.90, p = 0.21								

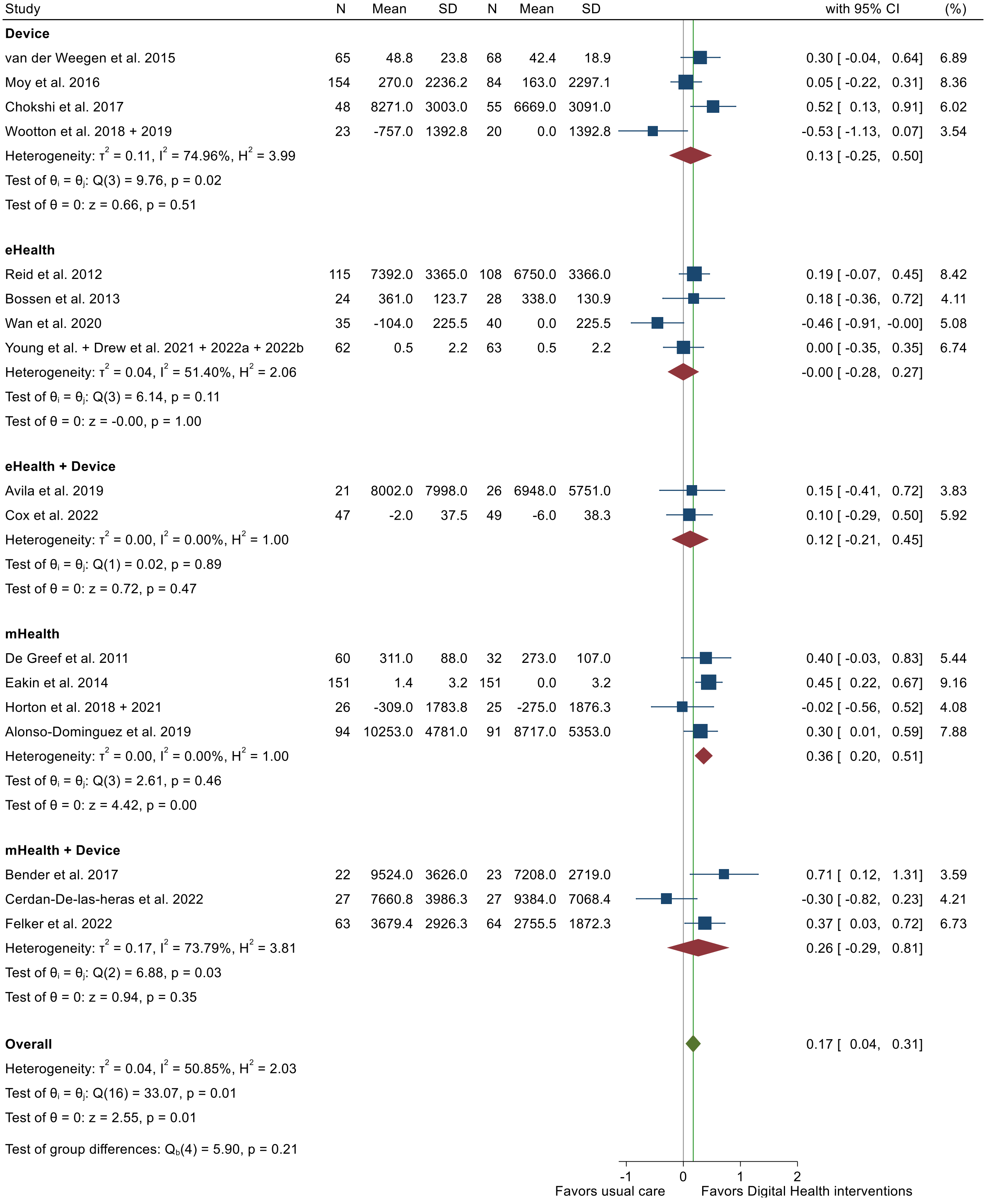

Supplementary Figure 4. Forest plot for objectively measured physical function at follow-up sub-grouped by primary digital health intervention delivery methods

Study	Treatment N	Treatment Mean	Treatment SD	Control N	Control Mean	Control SD	SMD with 95% CI	Weight (%)
**Device**								
Wootton et al. 2018 + 2019	35	469.0	98.0	27	432.0	121.0	0.34 [-0.16, 0.84]	10.77
Jaarsma et al. 2021	207	456.0	122.0	216	420.0	135.0	0.28 [0.09, 0.47]	15.23
Heterogeneity: τ^2 = 0.00, I^2 = 0.00%, H^2 = 1.00							0.29 [0.11, 0.46]	
Test of $\theta_i = \theta_j$: Q(1) = 0.04, p = 0.83								
Test of θ = 0: z = 3.14, p = 0.00								
**eHealth**								
Allen et al. 2018	142	-1.5	5.9	68	-0.3	7.8	-0.19 [-0.47, 0.10]	13.96
Heterogeneity: τ^2 = 0.00, I^2 = .%, H^2 = .							-0.19 [-0.47, 0.10]	
Test of $\theta_i = \theta_j$: Q(0) = 0.00, p = .								
Test of θ = 0: z = -1.26, p = 0.21								
**eHealth + Device**								
Avila et al. 2019	16	7.9	1.3	18	7.3	1.3	0.47 [-0.19, 1.14]	8.48
Cox et al. 2022	47	18.0	76.6	49	-2.0	102.7	0.22 [-0.18, 0.62]	12.30
Heterogeneity: τ^2 = 0.00, I^2 = 0.00%, H^2 = 1.00							0.29 [-0.06, 0.63]	
Test of $\theta_i = \theta_j$: Q(1) = 0.41, p = 0.52								
Test of θ = 0: z = 1.64, p = 0.10								
**mHealth**								
Bernocchi et al. 2018	45	420.0	118.0	35	300.0	125.0	0.98 [0.52, 1.44]	11.31
Horton et al. 2018 + 2021	59	248.1	151.9	66	270.0	150.9	-0.14 [-0.49, 0.21]	13.05
Haejung et al. 2021	7	405.9	58.3	5	414.3	76.6	-0.12 [-1.18, 0.94]	4.88
Heterogeneity: τ^2 = 0.37, I^2 = 83.84%, H^2 = 6.19							0.28 [-0.50, 1.06]	
Test of $\theta_i = \theta_j$: Q(2) = 14.91, p = 0.00								
Test of θ = 0: z = 0.70, p = 0.48								
**mHealth + Device**								
Cerdan-De-las-heras et al. 2022	27	415.6	107.9	27	320.4	106.8	0.87 [0.32, 1.42]	10.02
Heterogeneity: τ^2 = 0.00, I^2 = .%, H^2 = .							0.87 [0.32, 1.42]	
Test of $\theta_i = \theta_j$: Q(0) = 0.00, p = .								
Test of θ = 0: z = 3.11, p = 0.00								
**Overall**							0.29 [0.01, 0.57]	
Heterogeneity: τ^2 = 0.12, I^2 = 76.17%, H^2 = 4.20								
Test of $\theta_i = \theta_j$: Q(8) = 29.08, p = 0.00								
Test of θ = 0: z = 2.06, p = 0.04								
Test of group differences: $Q_b(4)$ = 13.71, p = 0.01								

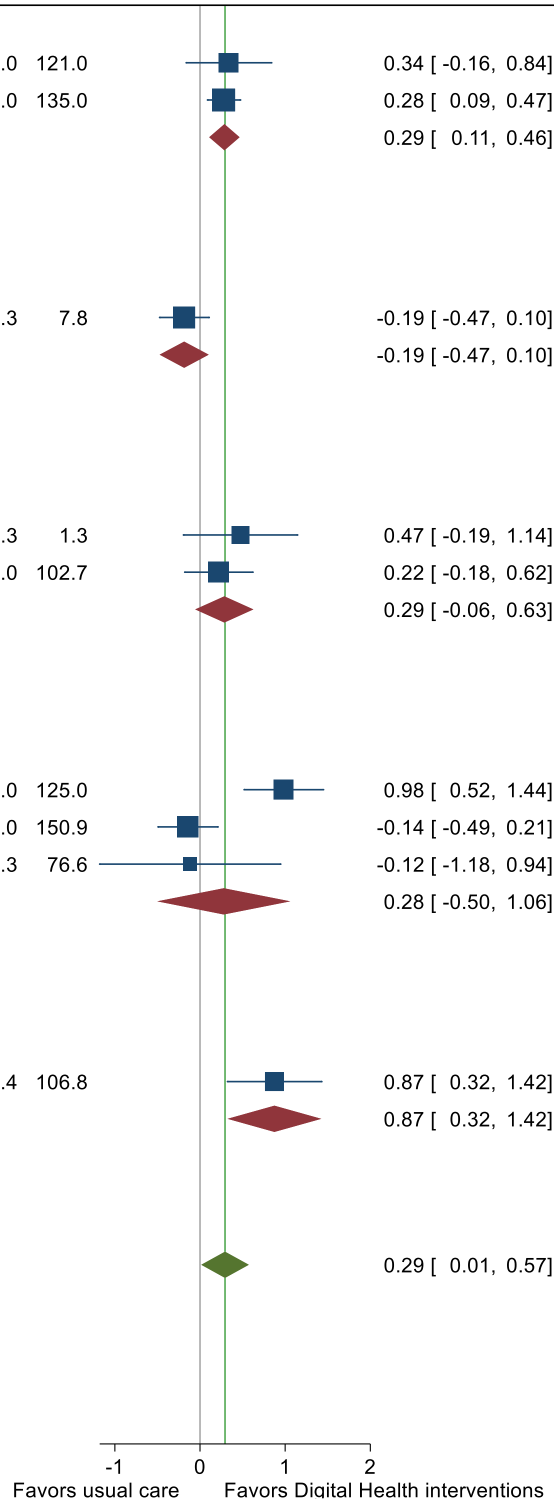

Supplementary Figure 5. Forest plot for objectively measured physical activity at end-of-intervention sub-grouped by the methodological quality

Study	Treatment N	Treatment Mean	Treatment SD	Control N	Control Mean	Control SD	SMD with 95% CI	Weight (%)
**High**								
Piette et al. 2011	145	4499.0	2612.0	146	3314.0	2516.0	0.46 [0.23, 0.69]	3.59
Reid et al. 2012	115	7079.0	3159.0	108	6168.0	3159.0	0.29 [0.02, 0.55]	3.31
Plotnikoff et al. 2013	68	21493.0	7289.7	78	20097.0	7339.2	0.19 [-0.13, 0.51]	2.80
Plotnikoff et al. 2013	62	20821.0	7283.5	39	20097.0	7339.2	0.10 [-0.30, 0.50]	2.28
Chaplin et al. 2016 + 2022	20	6111.7	2464.2	34	5409.4	3377.7	0.23 [-0.32, 0.77]	1.53
Tsai et al. 2017	19	3176.0	2004.0	17	2878.0	2852.0	0.12 [-0.52, 0.76]	1.21
Connelly et al. 2017	11	30058.0	16116.0	5	34597.0	17675.0	-0.26 [-1.26, 0.74]	0.57
Connelly et al. 2017	10	37083.0	25387.0	5	34597.0	17675.0	0.10 [-0.91, 1.11]	0.57
Lambert et al. 2018	13	97.6	86.4	11	13.0	97.3	0.89 [0.08, 1.71]	0.82
Al-Ghafri et al. 2018	33	7034.0	2408.0	36	6278.0	1270.0	0.39 [-0.08, 0.86]	1.86
Wootton et al. 2018 + 2019	23	4526.0	2527.4	20	5241.0	2276.3	-0.29 [-0.88, 0.30]	1.36
Horton et al. 2018 + 2021	26	1074.0	971.8	25	-464.0	2376.6	0.84 [0.28, 1.40]	1.46
Alonso-Dominguez et al. 2019	94	10714.0	4550.0	91	8464.0	4731.0	0.48 [0.19, 0.77]	3.07
Poppe et al. 2019	26	241.9	47.9	17	267.2	83.1	-0.39 [-0.99, 0.22]	1.32
Bentley et al. 2020	19	4258.0	2509.1	11	5124.9	2615.2	-0.33 [-1.06, 0.40]	0.99
Bailey et al. 2020	9	7109.3	2986.7	9	7492.2	2769.1	-0.13 [-1.01, 0.75]	0.72
Benzo et al. 2021	56	-344.8	1657.7	63	-975.8	2596.3	0.28 [-0.08, 0.64]	2.54
Coombes et al. 2021	12	484.6	216.3	13	441.2	342.6	0.15 [-0.61, 0.90]	0.92
Cerdan-De-las-heras et al. 2022	27	8999.8	4241.7	27	11984.6	10783.0	-0.36 [-0.89, 0.17]	1.59
Felker et al. 2022	74	3752.0	2902.8	78	2919.6	2167.1	0.32 [0.01, 0.64]	2.84
Heterogeneity: τ^2 = 0.03, I^2 = 35.13%, H^2 = 1.54							0.23 [0.10, 0.36]	
Test of $\theta_i = \theta_j$: Q(19) = 30.46, p = 0.05								
Test of θ = 0: z = 3.41, p = 0.00								
**Low**								
Eakin et al. 2014	151	1.4	3.7	151	0.0	3.7	0.38 [0.15, 0.61]	3.64
Frederix et al. 2015	69	8233.0	5501.0	70	5265.0	5541.4	0.53 [0.20, 0.87]	2.70
Bender et al. 2017	22	7483.0	2415.0	23	6735.0	2363.0	0.31 [-0.27, 0.89]	1.41
Heterogeneity: τ^2 = 0.00, I^2 = 0.00%, H^2 = 1.00							0.42 [0.24, 0.60]	
Test of $\theta_i = \theta_j$: Q(2) = 0.70, p = 0.71								
Test of θ = 0: z = 4.58, p = 0.00								
**Some concerns**								
Dyson et al. 2010	21	1266.0	3212.5	18	-712.0	3212.5	0.60 [-0.03, 1.23]	1.24
De Greef et al. 2011	60	323.0	87.0	32	306.0	102.0	0.18 [-0.24, 0.61]	2.11
Guiraud et al. 2012	19	543.7	144.1	10	266.7	107.4	2.02 [1.12, 2.93]	0.68
Bossen et al. 2013	27	361.0	131.2	30	358.0	135.5	0.02 [-0.49, 0.53]	1.67
Vasilopoulou et al. 2014	47	349.9	167.6	50	172.5	192.5	0.97 [0.55, 1.39]	2.15
Hornikx et al. 2015	12	984.0	1208.0	15	1013.0	1275.0	-0.02 [-0.76, 0.71]	0.97
Moy et al. 2015	133	3922.0	2491.0	68	3200.0	2489.0	0.29 [-0.00, 0.58]	3.06
van der Weegen et al. 2015	65	48.2	23.8	68	39.6	19.5	0.39 [0.05, 0.73]	2.67
Agboola et al. 2016	64	14180.0	55038.5	62	8220.0	41592.6	0.12 [-0.23, 0.47]	2.62
Chokshi et al. 2017	49	8705.0	3107.0	55	6841.0	3254.0	0.58 [0.19, 0.97]	2.33
Demeyer et al. 2017	140	4767.0	3606.7	140	4059.0	2746.7	0.22 [-0.01, 0.45]	3.57
Wan et al. 2017	57	804.0	3954.6	52	0.0	3954.6	0.20 [-0.17, 0.58]	2.43
Akinci et al. 2018	21	7310.3	914.3	22	6535.9	1869.8	0.51 [-0.08, 1.11]	1.35
Li et al. 2018	30	29.2	32.9	31	10.8	19.2	0.68 [0.17, 1.19]	1.68
Avila et al. 2018 + 2019	24	6469.0	4090.8	26	6408.0	2231.2	0.02 [-0.53, 0.56]	1.53
Bartholdy et al. 2019	19	-5.6	64.6	19	14.8	64.6	-0.31 [-0.94, 0.32]	1.25
Hochsmann et al. 2019	18	9783.0	1334.0	17	6552.0	1280.0	2.41 [1.55, 3.27]	0.75
Li et al. 2020	26	7133.3	3603.3	25	6232.7	3086.1	0.26 [-0.28, 0.81]	1.54
Robinson et al. 2021	75	672.9	3399.0	78	-639.4	3596.7	0.37 [0.05, 0.69]	2.85
Andrade et al. 2021	11	6873.0	2819.0	12	6541.0	2225.0	0.13 [-0.66, 0.92]	0.87
Radhakrishnan et al. 2021	19	2887.0	2821.0	19	2541.0	1604.0	0.15 [-0.48, 0.77]	1.26
Reid et al. 2021	68	225.2	163.0	67	216.9	179.8	0.05 [-0.29, 0.38]	2.71
Reid et al. 2021	158	284.5	205.3	156	284.8	202.3	-0.00 [-0.22, 0.22]	3.69
Young et al. + Drew et al. 2021 + 2022a + 2022b	62	0.8	2.2	63	-0.1	2.2	0.41 [0.06, 0.76]	2.59
Benzo et al. 2022	188	7796.1	3337.5	187	7047.5	3248.7	0.23 [0.02, 0.43]	3.86
Chan et al. 2022	23	10206.0	5534.0	21	8068.0	4994.8	0.40 [-0.19, 0.98]	1.38
Cox et al. 2022	47	-1.0	25.5	49	-3.0	36.6	0.06 [-0.33, 0.46]	2.28
Spielmanns et al. 2022	33	5016.3	5397.2	34	3105.1	2789.3	0.44 [-0.04, 0.92]	1.82
Heterogeneity: τ^2 = 0.07, I^2 = 64.22%, H^2 = 2.79							0.34 [0.20, 0.47]	
Test of $\theta_i = \theta_j$: Q(27) = 73.58, p = 0.00								
Test of θ = 0: z = 4.99, p = 0.00								
**Overall**							0.29 [0.21, 0.37]	
Heterogeneity: τ^2 = 0.03, I^2 = 44.69%, H^2 = 1.81								
Test of $\theta_i = \theta_j$: Q(50) = 107.04, p = 0.00								
Test of θ = 0: z = 7.09, p = 0.00								
Test of group differences: $Q_b(2)$ = 3.16, p = 0.21								

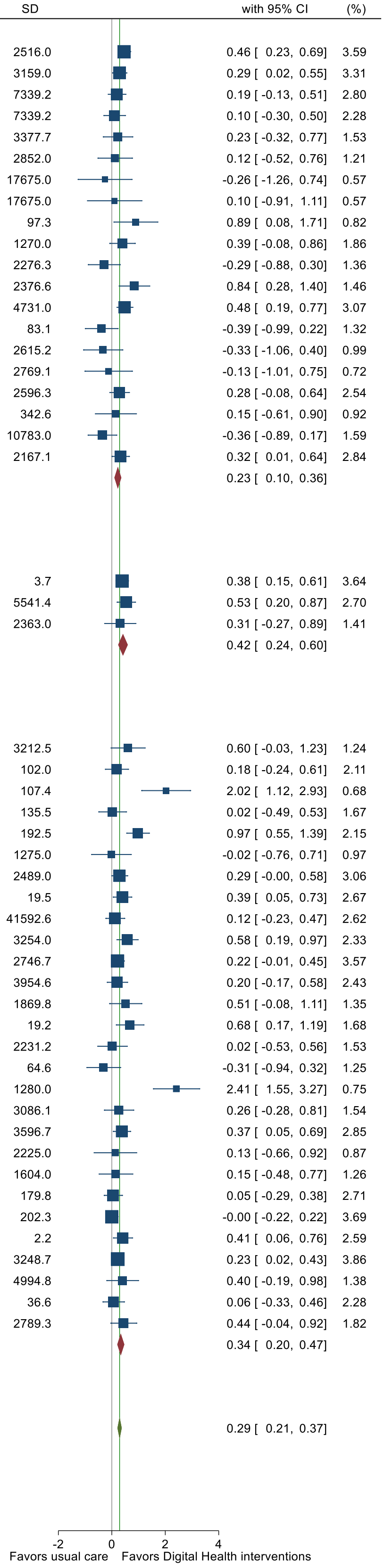

Supplementary Figure 6. Forest plot for objectively measured physical function at end-of-intervention sub-grouped by the methodological quality

Study	Treatment N	Treatment Mean	Treatment SD	Control N	Control Mean	Control SD	SMD with 95% CI	Weight (%)
**High**								
Moore et al. 2009	10	45.0	51.0	10	-15.0	39.1	1.26 [0.34, 2.19]	1.76
Piotrowicz et al. 2015	75	480.0	87.0	32	465.0	91.0	0.17 [-0.24, 0.58]	2.24
Coultas et al. 2016	113	343.1	99.8	134	324.1	107.5	0.18 [-0.07, 0.43]	2.34
Chaplin et al. 2016 + 2022	51	189.0	211.1	52	184.5	247.4	0.02 [-0.36, 0.40]	2.26
Tsai et al. 2017	19	403.0	82.0	17	374.0	136.0	0.26 [-0.39, 0.90]	2.04
Horton et al. 2018 + 2021	85	281.2	147.9	85	309.5	156.4	-0.19 [-0.49, 0.11]	2.31
Baker et al. 2019	52	-7.5	3.9	52	-7.7	7.2	0.04 [-0.34, 0.43]	2.26
Bentley et al. 2020	9	280.0	131.4	6	308.3	61.1	-0.24 [-1.22, 0.73]	1.71
Li et al. 2021	44	-2.0	4.8	41	1.0	5.2	-0.59 [-1.03, -0.16]	2.23
Clays et al. 2021	30	0.9	88.8	17	4.8	39.4	-0.05 [-0.64, 0.53]	2.10
Haejung et al. 2021	7	407.7	67.7	5	421.0	108.5	-0.14 [-1.20, 0.92]	1.62
Cerdan-De-las-heras et al. 2022	27	434.6	84.5	27	387.1	100.9	0.50 [-0.03, 1.04]	2.14
Heterogeneity: τ^2 = 0.05, I^2 = 50.61%, H^2 = 2.02							0.05 [-0.14, 0.25]	
Test of $\theta_i = \theta_j$: Q(11) = 22.69, p = 0.02								
Test of θ = 0: z = 0.53, p = 0.60								
**Low**								
Doiron-Cadrin et al. 2020	12	-8.5	1.4	11	-11.8	4.4	0.99 [0.15, 1.83]	1.85
Gohir et al. 2021	48	1.8	1.5	57	0.0	1.5	1.19 [0.78, 1.61]	2.24
Heterogeneity: τ^2 = 0.00, I^2 = 0.00%, H^2 = 1.00							1.15 [0.78, 1.52]	
Test of $\theta_i = \theta_j$: Q(1) = 0.17, p = 0.68								
Test of θ = 0: z = 6.09, p = 0.00								
**Some concerns**								
Witham et al. 2011	43	-5.3	64.0	44	0.0	64.0	-0.08 [-0.50, 0.33]	2.24
de Sousa Pinto et al. 2014	23	260.0	116.0	18	172.0	93.0	0.81 [0.18, 1.44]	2.05
Vasilopoulou et al. 2014	47	420.2	74.9	50	339.9	110.1	0.84 [0.43, 1.25]	2.24
Hornikx et al. 2015	12	67.0	84.0	15	64.0	59.0	0.04 [-0.70, 0.78]	1.95
Cameron-Tucker et al. 2016	35	0.0	41.0	30	12.0	39.0	-0.30 [-0.78, 0.19]	2.18
Demeyer et al. 2017	150	457.0	108.0	150	449.0	118.0	0.07 [-0.16, 0.30]	2.35
Wan et al. 2017	57	-0.9	55.8	52	2.6	46.7	-0.07 [-0.44, 0.31]	2.27
Akinci et al. 2018	21	539.3	37.4	22	490.9	59.7	0.95 [0.33, 1.57]	2.06
Bernocchi et al. 2018	48	60.0	15.4	44	-15.0	10.2	5.63 [4.72, 6.54]	1.78
Deka et al. 2018	15	399.0	102.0	15	368.0	131.0	0.26 [-0.44, 0.96]	1.99
Kwon et al. 2018	24	389.1	91.4	11	381.3	73.9	0.09 [-0.61, 0.79]	1.99
Kwon et al. 2018	16	381.3	75.9	11	381.3	73.9	0.00 [-0.74, 0.74]	1.94
Peng et al. 2018	42	418.3	62.7	41	406.4	80.5	0.16 [-0.26, 0.59]	2.23
Allen et al. 2018	142	-0.9	5.2	68	-0.2	6.9	-0.11 [-0.40, 0.18]	2.32
Wootton et al. 2018 + 2019	42	479.0	86.0	35	468.0	100.0	0.12 [-0.33, 0.56]	2.21
Duruturk et al. 2019	23	554.4	139.0	21	450.9	165.8	0.67 [0.07, 1.26]	2.08
Piotrowicz et al. 2019	422	450.0	109.5	423	432.0	106.7	0.17 [0.03, 0.30]	2.38
Jiménez-Reguera et al. 2020	17	357.4	112.5	19	339.9	75.9	0.18 [-0.46, 0.82]	2.04
Allen et al. 2021	230	-5.5	183.4	115	-15.5	236.0	0.05 [-0.17, 0.27]	2.35
Galdiz et al. 2021	38	441.8	106.3	40	423.7	101.6	0.17 [-0.27, 0.61]	2.22
Hsu et al. 2021	21	-9.4	1.6	21	-9.9	1.7	0.33 [-0.27, 0.93]	2.08
Jaarsma et al. 2021	243	454.0	123.0	243	420.0	127.0	0.27 [0.09, 0.45]	2.37
Robinson et al. 2021	75	25.1	83.2	78	37.4	83.4	-0.15 [-0.46, 0.17]	2.30
Andrade et al. 2021	11	500.7	86.1	12	538.6	72.3	-0.46 [-1.26, 0.34]	1.89
Yudi et al. 2021	83	564.0	102.9	85	534.6	112.6	0.27 [-0.03, 0.57]	2.31
Chan et al. 2022	70	16.9	6.4	69	15.9	5.0	0.17 [-0.16, 0.50]	2.29
Cox et al. 2022	47	20.0	51.1	49	26.0	62.7	-0.10 [-0.50, 0.29]	2.25
Indraratna et al. 2022	25	469.0	114.0	21	463.0	125.0	0.05 [-0.52, 0.62]	2.11
Li et al. 2022	50	416.3	35.6	49	421.1	66.6	-0.09 [-0.48, 0.30]	2.25
Nagatomi et al. 2022	15	52.1	43.9	15	-4.3	38.8	1.32 [0.55, 2.10]	1.92
Shi et al. 2022	25	469.4	57.5	26	432.8	67.1	0.58 [0.02, 1.13]	2.13
Spielmanns et al. 2022	30	22.7	7.2	30	19.7	6.4	0.42 [-0.08, 0.93]	2.17
Tore et al. 2022	24	17.3	1.1	24	12.8	2.3	2.45 [1.70, 3.19]	1.95
Heterogeneity: τ^2 = 0.85, I^2 = 96.00%, H^2 = 24.99							0.41 [0.09, 0.74]	
Test of $\theta_i = \theta_j$: Q(32) = 232.91, p = 0.00								
Test of θ = 0: z = 2.48, p = 0.01								
**Overall**							0.36 [0.12, 0.59]	
Heterogeneity: τ^2 = 0.61, I^2 = 94.07%, H^2 = 16.86								
Test of $\theta_i = \theta_j$: Q(46) = 287.35, p = 0.00								
Test of θ = 0: z = 2.93, p = 0.00								
Test of group differences: $Q_b(2)$ = 26.82, p = 0.00								

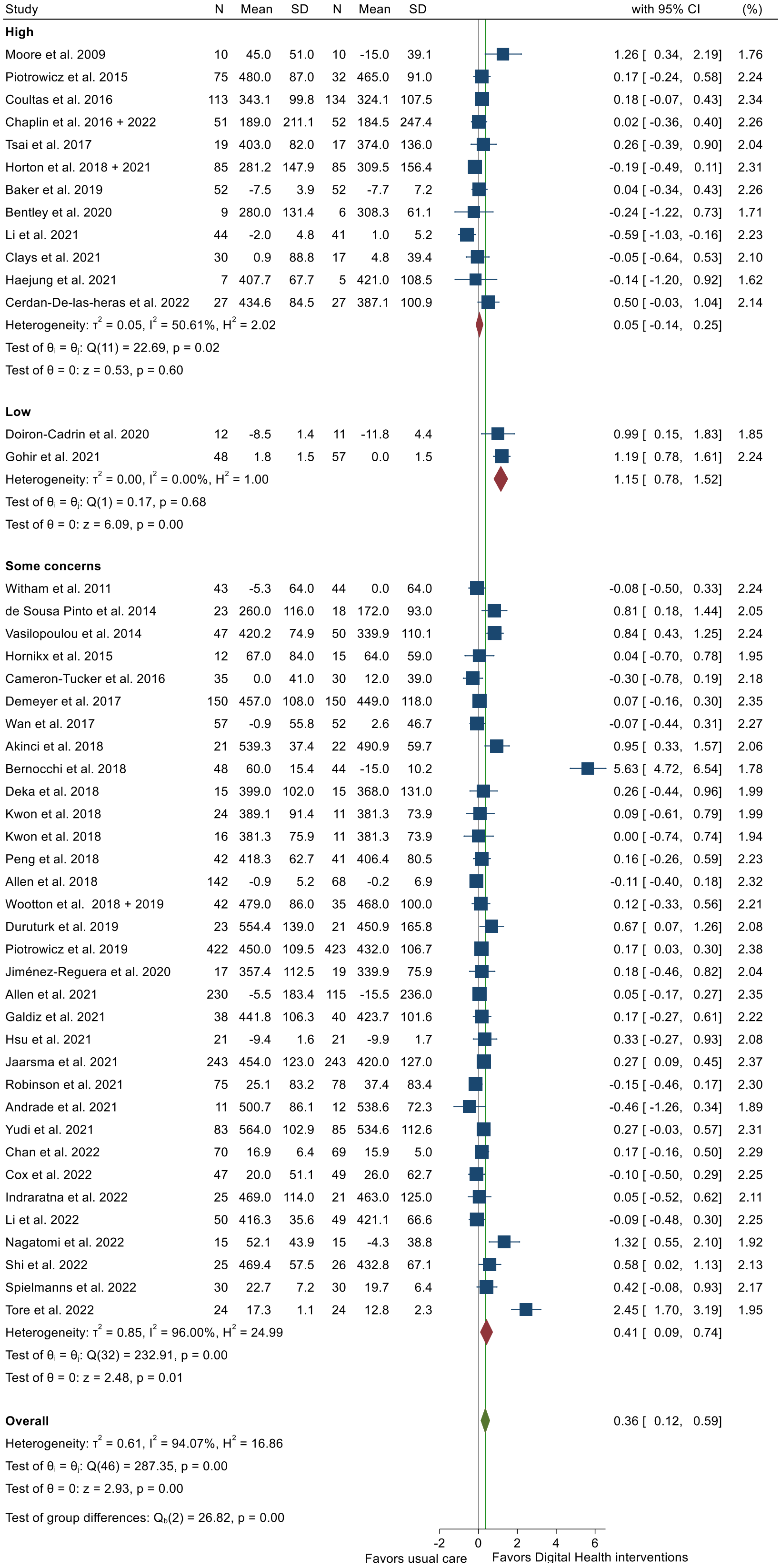

Supplementary Figure 7. Forest plot for subjectively measured physical activity at end-of-intervention sub-grouped by the methodological quality

Study	Treatment N	Treatment Mean	Treatment SD	Control N	Control Mean	Control SD	SMD with 95% CI	Weight (%)
**High**								
Southard et al. 2003	51	208.4	122.4	49	165.0	122.4	0.35 [-0.04, 0.74]	1.71
Kim et al. 2006	28	26.7	10.2	23	14.9	7.7	1.27 [0.67, 1.87]	1.29
Hanssen et al. 2007	156	0.9	66.6	132	0.0	66.6	0.01 [-0.22, 0.24]	2.05
Liebreich et al. 2009	23	654.0	659.0	21	490.0	562.0	0.26 [-0.32, 0.85]	1.31
Tomita et al. 2009	19	1.4	7.9	13	0.0	7.9	0.17 [-0.52, 0.86]	1.12
Anderson et al. 2010	94	-0.0	2.2	117	0.0	1.7	-0.02 [-0.29, 0.25]	1.97
Strom et al. 2013	24	1331.0	990.0	24	1143.0	918.0	0.19 [-0.36, 0.75]	1.36
Holmen et al. 2014	37	-0.2	24.3	20	0.0	24.3	-0.01 [-0.54, 0.53]	1.40
Holmen et al. 2014	40	-0.3	25.0	20	0.0	25.0	-0.01 [-0.54, 0.52]	1.42
Varney et al. 2014	38	183.0	243.4	43	119.0	243.7	0.26 [-0.17, 0.69]	1.62
Coultas et al. 2016	113	0.0	4.0	134	0.0	4.0	0.00 [-0.25, 0.25]	2.01
Namjoo Nasab et al. 2017	30	8.1	1.7	30	5.8	2.6	1.06 [0.53, 1.60]	1.41
Widmer et al. 2017	37	179.2	109.1	34	139.0	87.7	0.40 [-0.07, 0.87]	1.55
Dobler et al. 2018	98	1.2	2.0	101	0.3	1.9	0.50 [0.21, 0.78]	1.95
Duan et al. 2018	44	218.2	141.7	39	136.2	148.0	0.56 [0.13, 1.00]	1.62
Kooiman et al. 2018	36	1.5	3.0	30	0.0	1.8	0.59 [0.10, 1.08]	1.50
Lee et al. 2018	72	3.5	1.8	64	2.9	1.4	0.37 [0.03, 0.70]	1.83
Jayasree et al. 2019	25	229.0	379.5	24	197.5	241.6	0.10 [-0.45, 0.65]	1.37
Jennings et al. 2019	195	906.7	3007.9	202	738.2	2376.4	0.06 [-0.13, 0.26]	2.11
Osteresch et al. + Wienbergen et al. 2019 + 2021	134	1850.0	1695.6	136	608.0	1130.4	0.86 [0.61, 1.11]	2.02
Hidrus et al. 2020	37	357.8	300.7	33	0.0	300.7	1.18 [0.67, 1.68]	1.47
Pelle et al. 2020	214	26.5	17.6	213	26.3	18.1	0.01 [-0.18, 0.20]	2.12
Allen et al. 2021	230	24.6	109.2	115	14.3	143.3	0.08 [-0.14, 0.31]	2.06
Haejung et al. 2021	17	399.8	461.2	17	99.7	282.7	0.77 [0.08, 1.45]	1.13
Heterogeneity: τ^2 = 0.10, I^2 = 76.57%, H^2 = 4.27							0.35 [0.19, 0.50]	
Test of $\theta_i = \theta_j$: Q(23) = 91.87, p = 0.00								
Test of θ = 0: z = 4.47, p = 0.00								
**Low**								
Bennell et al. 2018	66	189.0	111.0	65	186.0	80.0	0.03 [-0.31, 0.37]	1.83
Nelligan et al. 2021	103	157.5	75.6	103	144.5	73.0	0.17 [-0.10, 0.45]	1.97
Heterogeneity: τ^2 = 0.00, I^2 = 0.00%, H^2 = 1.00							0.12 [-0.09, 0.33]	
Test of $\theta_i = \theta_j$: Q(1) = 0.42, p = 0.52								
Test of θ = 0: z = 1.09, p = 0.28								
**Some concerns**								
Eakin et al. 2009	228	71.2	214.4	206	82.2	214.0	-0.05 [-0.24, 0.14]	2.12
Glasgow et al. 2012	169	3307.0	3276.0	66	2882.0	2437.2	0.14 [-0.15, 0.42]	1.95
Glasgow et al. 2012	162	3174.0	3245.6	66	2882.0	2437.2	0.10 [-0.19, 0.38]	1.94
Hawkes et al. 2013	156	208.7	207.9	170	200.8	212.8	0.04 [-0.18, 0.25]	2.08
Cicolini et al. 2014	100	23.0	7.3	98	13.0	8.1	1.29 [0.99, 1.60]	1.90
Chow et al. 2015	352	932.0	1024.2	358	587.0	1013.6	0.34 [0.19, 0.49]	2.18
Cameron-Tucker et al. 2016	35	14.0	26.0	30	16.0	40.0	-0.06 [-0.54, 0.42]	1.52
Bernocchi et al. 2018	48	18.1	64.6	44	-21.3	47.2	0.69 [0.27, 1.10]	1.66
Deka et al. 2018	15	88.2	81.9	15	85.9	44.2	0.03 [-0.66, 0.73]	1.11
Haller et al. 2018	14	8.3	1.5	6	7.3	2.2	0.58 [-0.36, 1.51]	0.79
Allen et al. 2018	142	-11.5	84.4	68	-4.7	112.4	-0.07 [-0.36, 0.22]	1.94
O'Brien et al. 2018	37	179.7	324.8	52	185.3	383.6	-0.02 [-0.43, 0.40]	1.66
Baker et al. 2019	52	3.6	3.0	52	4.0	4.0	-0.11 [-0.49, 0.27]	1.73
Benson et al. 2019	60	0.1	2.0	58	0.0	2.0	0.07 [-0.29, 0.42]	1.79
Gingele et al. 2019	179	4.4	1.9	173	3.9	1.7	0.25 [0.04, 0.46]	2.09
Hinman et al. 2019	83	190.0	91.0	82	172.0	99.0	0.19 [-0.12, 0.49]	1.90
Vinitha et al. 2019	126	10.2	10.7	122	10.1	9.6	0.01 [-0.24, 0.26]	2.02
Turan Kavradim 2020	31	1.6	21.2	31	0.0	21.2	0.08 [-0.42, 0.57]	1.50
Bennell et al. 2020	56	190.5	111.3	54	174.0	71.0	0.17 [-0.20, 0.55]	1.76
Wong et al. 2020	219	18.2	13.5	219	14.8	12.6	0.26 [0.07, 0.45]	2.13
Jaarsma et al. 2021	201	0.1	4.5	203	0.0	4.5	0.02 [-0.17, 0.22]	2.11
Lim et al. 2021	99	71.4	207.9	105	9.0	122.0	0.37 [0.09, 0.64]	1.96
Snoek et al. 2021	61	5008.0	3931.0	61	5265.0	4682.0	-0.06 [-0.41, 0.29]	1.80
Vluggen et al. 2021	111	-0.1	1.0	177	0.0	1.0	-0.13 [-0.37, 0.10]	2.04
Waller et al. 2021	197	2.3	4.1	198	2.0	4.4	0.07 [-0.13, 0.26]	2.11
Wang et al. 2021	39	-1.2	4.8	39	0.0	4.8	-0.25 [-0.70, 0.19]	1.60
Wong et al. 2021	39	1802.0	2398.1	40	757.6	1232.3	0.54 [0.10, 0.99]	1.60
Bozorgi et al. 2021	58	260.9	115.9	60	154.6	135.5	0.84 [0.46, 1.21]	1.75
Michelsen et al. 2022	96	2.6	2.6	48	1.8	2.2	0.32 [-0.03, 0.67]	1.81
Pamungkas et al. 2022	30	6.9	1.4	30	3.8	1.3	2.34 [1.69, 3.00]	1.18
Pitta et al. 2022	54	7.9	1.8	56	6.9	1.9	0.55 [0.18, 0.93]	1.74
Tore et al. 2022	24	1225.6	829.4	24	727.6	360.8	0.77 [0.19, 1.34]	1.32
Heterogeneity: τ^2 = 0.15, I^2 = 87.44%, H^2 = 7.96							0.26 [0.11, 0.41]	
Test of $\theta_i = \theta_j$: Q(31) = 162.65, p = 0.00								
Test of θ = 0: z = 3.44, p = 0.00								
**Overall**							0.29 [0.19, 0.39]	
Heterogeneity: τ^2 = 0.12, I^2 = 83.12%, H^2 = 5.93								
Test of $\theta_i = \theta_j$: Q(57) = 257.05, p = 0.00								
Test of θ = 0: z = 5.52, p = 0.00								
Test of group differences: $Q_b(2)$ = 2.90, p = 0.23								

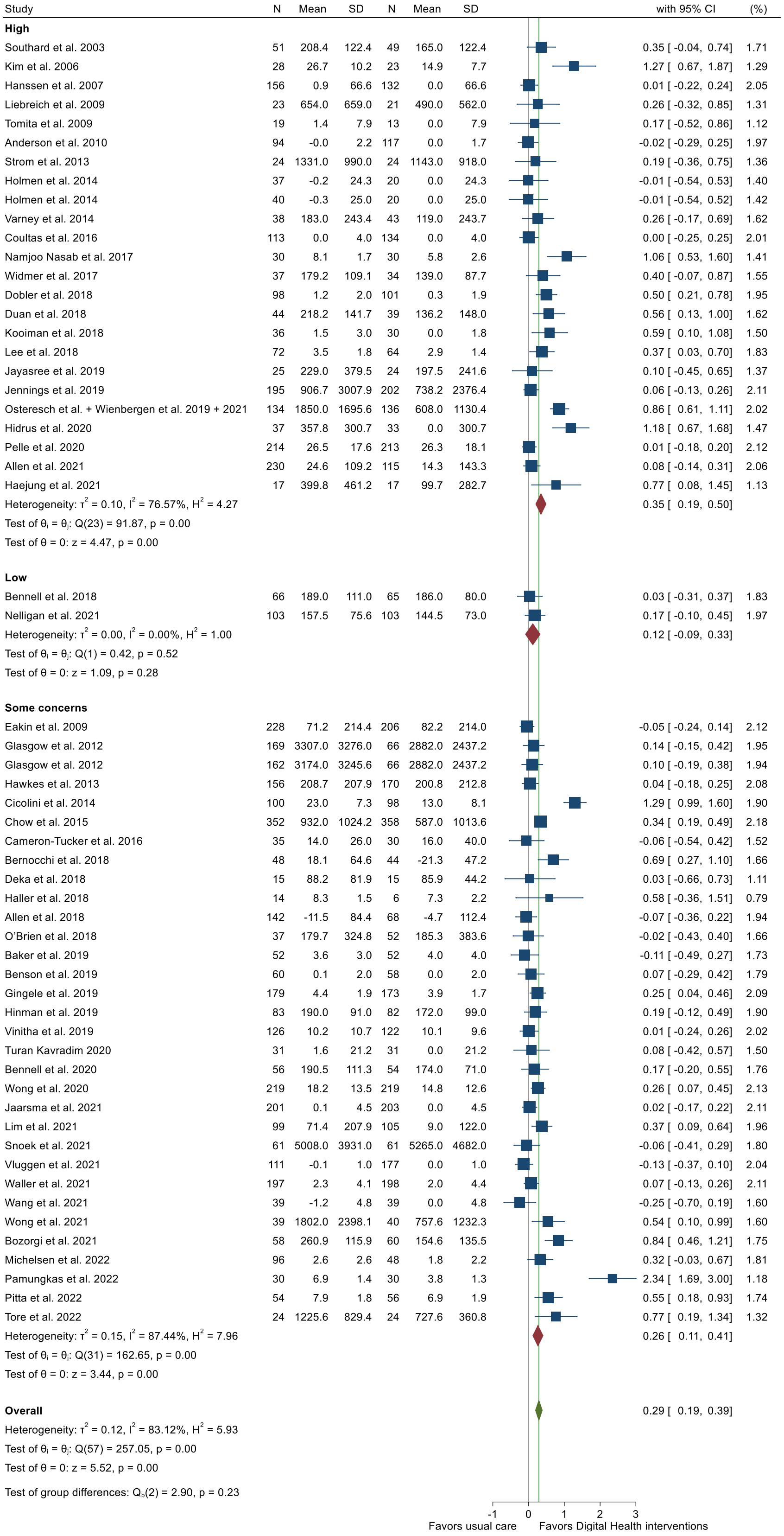

Supplementary Figure 8. Forest plot for subjectively measured physical function at end-of-intervention sub-grouped by the methodological quality

Study	Treatment N	Treatment Mean	Treatment SD	Control N	Control Mean	Control SD	SMD with 95% CI	Weight (%)
**High**								
Southard et al. 2003	51	44.9	14.6	49	42.3	14.6	0.18 [-0.21, 0.57]	3.28
Hanssen et al. 2007	108	6.6	7.8	99	5.5	7.6	0.15 [-0.12, 0.42]	5.63
Utriyaprasit 2010	51	-15.3	8.1	52	-16.7	8.7	0.17 [-0.21, 0.56]	3.36
Piette et al. 2011	145	39.5	11.7	146	36.3	12.2	0.27 [0.04, 0.50]	6.98
Reid et al. 2012	115	5.9	0.9	108	5.7	1.1	0.20 [-0.06, 0.46]	5.91
Bossen et al. 2013	84	67.8	40.2	80	61.3	27.8	0.19 [-0.12, 0.49]	4.78
Moy et al. 2015	145	60.4	22.7	79	61.9	21.4	-0.07 [-0.34, 0.21]	5.60
Li et al. 2018	30	76.0	16.1	31	71.0	16.5	0.30 [-0.20, 0.80]	2.16
Jiwani et al. 2020	11	40.2	8.2	6	45.1	11.9	-0.48 [-1.44, 0.47]	0.64
Pelle et al. 2020	214	62.1	20.8	213	58.6	19.3	0.17 [-0.02, 0.36]	8.67
Coombes et al. 2021	12	93.3	9.1	13	85.8	23.0	0.41 [-0.36, 1.18]	0.98
Heterogeneity: $\tau^2 = 0.00$, $I^2 = 0.00\%$, $H^2 = 1.00$							0.16 [0.07, 0.26]	
Test of $\theta_i = \theta_j$: Q(10) = 6.08, p = 0.81								
Test of $\theta = 0$: z = 3.52, p = 0.00								
**Low**								
Bennell et al. 2018	66	-17.7	18.0	65	-18.0	12.9	0.02 [-0.32, 0.36]	4.06
Nelligan et al. 2021	103	-16.6	13.0	103	-20.7	13.9	0.30 [0.03, 0.58]	5.58
Heterogeneity: $\tau^2 = 0.02$, $I^2 = 38.63\%$, $H^2 = 1.63$							0.18 [-0.10, 0.46]	
Test of $\theta_i = \theta_j$: Q(1) = 1.63, p = 0.20								
Test of $\theta = 0$: z = 1.28, p = 0.20								
**Some concerns**								
Hawkes et al. 2013	142	45.5	10.9	156	46.0	10.7	-0.05 [-0.27, 0.18]	7.11
Furuya et al. 2014	30	72.5	23.9	30	64.5	27.8	0.30 [-0.20, 0.81]	2.13
Bennell et al. 2017	70	-18.3	10.7	69	-27.6	11.7	0.83 [0.48, 1.17]	3.99
Haller et al. 2018	14	83.9	18.3	6	75.8	11.1	0.47 [-0.46, 1.39]	0.68
O'Brien et al. 2018	37	36.5	13.2	51	32.8	15.1	0.26 [-0.17, 0.68]	2.88
Avila et al. 2018 + 2019	28	91.9	7.6	26	84.3	16.1	0.60 [0.06, 1.14]	1.88
Bartholdy et al. 2019	19	-1.8	11.3	19	-4.4	11.3	0.23 [-0.40, 0.85]	1.44
Hinman et al. 2019	83	-18.4	11.3	82	-22.0	12.5	0.30 [-0.00, 0.61]	4.78
Bennell et al. 2020	56	72.4	17.6	54	70.0	21.1	0.12 [-0.25, 0.49]	3.54
Li et al. 2020	24	75.0	13.1	24	70.3	16.9	0.31 [-0.25, 0.87]	1.76
Radhakrishnan et al. 2021	19	3.7	14.1	19	4.1	12.6	-0.03 [-0.65, 0.59]	1.45
Wong et al. 2021	39	-7.0	5.9	40	-8.5	7.8	0.21 [-0.22, 0.65]	2.70
Benzo et al. 2022	188	69.1	12.2	187	65.2	12.7	0.31 [0.11, 0.52]	8.06
Heterogeneity: $\tau^2 = 0.03$, $I^2 = 45.89\%$, $H^2 = 1.85$							0.28 [0.13, 0.44]	
Test of $\theta_i = \theta_j$: Q(12) = 20.87, p = 0.05								
Test of $\theta = 0$: z = 3.67, p = 0.00								
**Overall**							0.21 [0.13, 0.29]	
Heterogeneity: $\tau^2 = 0.01$, $I^2 = 23.61\%$, $H^2 = 1.31$								
Test of $\theta_i = \theta_j$: Q(25) = 30.55, p = 0.20								
Test of $\theta = 0$: z = 5.31, p = 0.00								
Test of group differences: $Q_b(2) = 1.75$, p = 0.42								

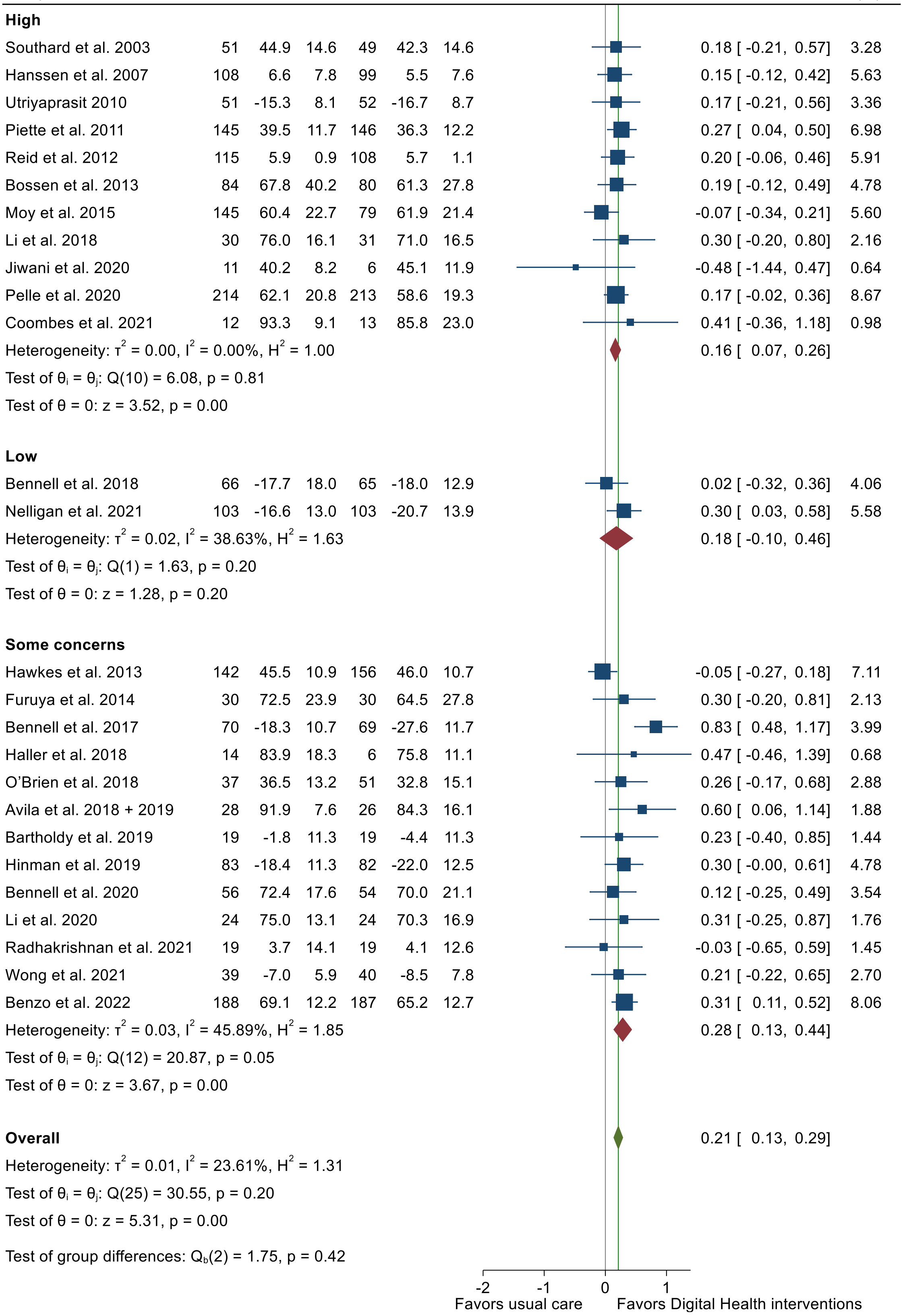

Supplementary Figure 9. Forest plot for depression at end-of-intervention sub-grouped by the methodological quality

Study	Treatment N	Treatment Mean	Treatment SD	Control N	Control Mean	Control SD	SMD with 95% CI	Weight (%)
**High**								
Southard et al. 2003	51	6.8	8.4	49	6.1	8.4	0.08 [-0.31, 0.47]	2.81
Utriyaprasit 2010	51	-4.2	6.4	52	-2.5	5.0	-0.29 [-0.67, 0.10]	2.83
Piette et al. 2011	145	14.2	10.3	146	18.6	10.7	-0.42 [-0.65, -0.19]	4.00
Bossen et al. 2013	85	2.6	5.2	78	3.2	5.0	-0.12 [-0.42, 0.19]	3.41
Strom et al. 2013	24	17.9	11.3	24	24.0	6.9	-0.65 [-1.22, -0.08]	1.83
Holmen et al. 2014	38	9.2	9.2	20	10.8	10.0	-0.17 [-0.70, 0.37]	1.99
Holmen et al. 2014	39	7.6	6.2	20	10.8	10.0	-0.42 [-0.96, 0.12]	1.98
Varney et al. 2014	38	18.0	12.2	43	18.0	9.7	0.00 [-0.43, 0.43]	2.53
Piotrowicz et al. 2016	46	6.7	5.5	23	9.1	7.3	-0.38 [-0.88, 0.12]	2.16
Tsai et al. 2017	19	4.0	3.0	17	6.0	3.0	-0.65 [-1.31, 0.01]	1.51
Widmer et al. 2017	37	-2.4	3.7	34	0.6	2.3	-0.95 [-1.44, -0.47]	2.23
Dobler et al. 2018	98	-1.4	4.3	101	-0.2	4.9	-0.24 [-0.52, 0.04]	3.63
Duan et al. 2018	44	1.0	0.5	39	1.1	0.6	-0.22 [-0.65, 0.21]	2.56
Lambert et al. 2018	25	8.7	4.8	25	12.9	4.2	-0.92 [-1.49, -0.34]	1.82
Peng et al. 2018	42	6.6	6.3	41	6.8	5.5	-0.03 [-0.46, 0.40]	2.57
Horton et al. 2018 + 2021	82	6.1	3.7	86	5.5	3.8	0.16 [-0.15, 0.46]	3.44
Osteresch et al. + Wienbergen et al. 2019 + 2021	138	3.6	3.5	143	4.4	3.5	-0.23 [-0.46, 0.01]	3.98
Bentley et al. 2020	8	7.5	4.4	6	8.0	5.6	-0.09 [-1.09, 0.90]	0.79
Jiwani et al. 2020	11	46.6	12.9	6	44.0	7.4	0.22 [-0.73, 1.16]	0.86
Heterogeneity: $\tau^2 = 0.04$, $I^2 = 46.79\%$, $H^2 = 1.88$							-0.26 [-0.39, -0.13]	
Test of $\theta_i = \theta_j$: Q(18) = 33.15, p = 0.02								
Test of $\theta = 0$: z = -3.90, p = 0.00								
**Low**								
Bennell et al. 2018	66	2.0	3.0	65	2.8	5.1	-0.19 [-0.53, 0.15]	3.14
Heterogeneity: $\tau^2 = 0.00$, $I^2 = .\%$, $H^2 = .$							-0.19 [-0.53, 0.15]	
Test of $\theta_i = \theta_j$: Q(0) = 0.00, p = .								
Test of $\theta = 0$: z = -1.09, p = 0.27								
**Some concerns**								
Witham et al. 2011	43	-0.9	2.7	44	0.0	2.7	-0.33 [-0.75, 0.09]	2.61
Furuya et al. 2014	30	5.4	4.8	30	4.7	3.5	0.16 [-0.34, 0.66]	2.16
Wan et al. 2017	57	-0.6	3.6	52	-0.3	5.3	-0.07 [-0.44, 0.31]	2.92
Haller et al. 2018	14	11.2	3.8	6	11.0	5.2	0.05 [-0.87, 0.96]	0.91
O'Brien et al. 2018	37	8.7	7.5	52	9.8	9.8	-0.12 [-0.54, 0.30]	2.62
Duruturk et al. 2019	23	9.9	8.1	21	11.3	8.2	-0.17 [-0.75, 0.42]	1.79
Li et al. 2020	26	4.0	3.0	25	4.7	4.9	-0.17 [-0.71, 0.37]	1.96
Snoek et al. 2021	61	2.4	2.7	61	3.0	3.2	-0.20 [-0.55, 0.15]	3.05
Andrade et al. 2021	11	68.0	33.0	12	72.0	16.0	-0.15 [-0.94, 0.64]	1.15
Clays et al. 2021	33	-14.0	9.6	22	1.0	14.3	-1.26 [-1.85, -0.68]	1.79
Snoek et al. 2021	89	3.0	3.5	90	4.0	4.4	-0.25 [-0.54, 0.04]	3.51
Wong et al. 2021	39	8.8	3.8	40	11.6	4.7	-0.65 [-1.10, -0.20]	2.44
Yudi et al. 2021	83	13.7	6.6	85	12.5	5.9	0.19 [-0.11, 0.49]	3.44
Young et al. + Drew et al. 2021 + 2022a + 2022b	62	-4.2	4.3	63	-1.8	4.6	-0.54 [-0.89, -0.18]	3.05
Benzo et al. 2022	188	4.5	4.1	187	5.7	5.0	-0.27 [-0.47, -0.06]	4.23
Cox et al. 2022	47	-0.2	3.2	49	-0.4	4.7	0.05 [-0.35, 0.45]	2.76
Li et al. 2022	50	8.1	4.8	49	6.9	4.0	0.27 [-0.12, 0.66]	2.78
Pitta et al. 2022	54	2.9	3.4	56	4.3	4.7	-0.34 [-0.71, 0.04]	2.91
Spielmanns et al. 2022	30	4.2	3.0	30	6.5	5.1	-0.56 [-1.07, -0.05]	2.11
Tore et al. 2022	24	2.7	2.4	24	5.4	2.8	-1.03 [-1.62, -0.43]	1.74
Heterogeneity: $\tau^2 = 0.07$, $I^2 = 63.06\%$, $H^2 = 2.71$							-0.25 [-0.41, -0.10]	
Test of $\theta_i = \theta_j$: Q(19) = 47.00, p = 0.00								
Test of $\theta = 0$: z = -3.20, p = 0.00								
**Overall**							-0.25 [-0.35, -0.16]	
Heterogeneity: $\tau^2 = 0.05$, $I^2 = 53.49\%$, $H^2 = 2.15$								
Test of $\theta_i = \theta_j$: Q(39) = 80.30, p = 0.00								
Test of $\theta = 0$: z = -5.16, p = 0.00								
Test of group differences: $Q_b(2) = 0.14$, p = 0.93								

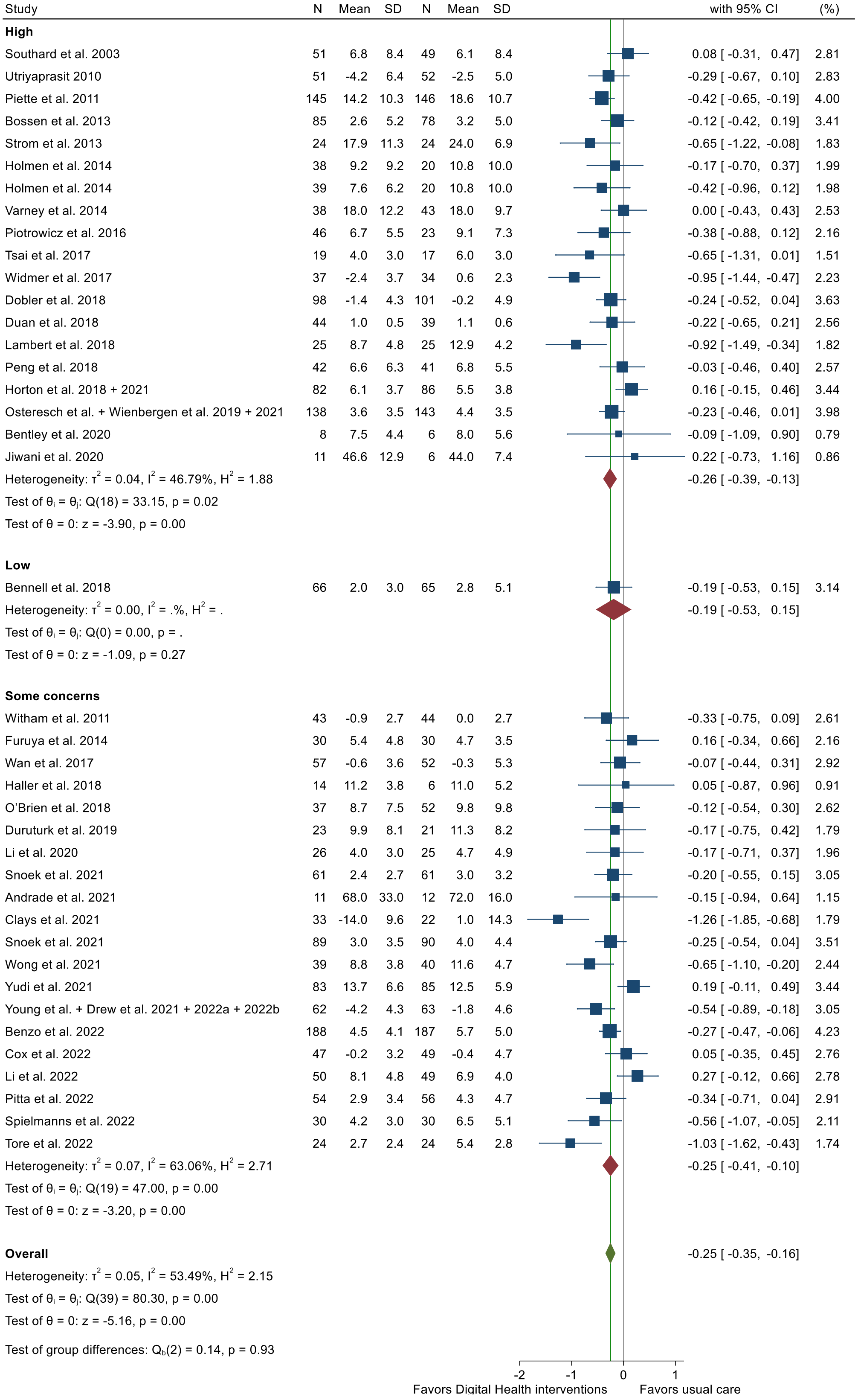

Supplementary Figure 10. Forest plot for anxiety at end-of-intervention sub-grouped by the methodological quality

Study	Treatment N	Treatment Mean	Treatment SD	Control N	Control Mean	Control SD	SMD with 95% CI	Weight (%)
**High**								
Utriyaprasit 2010	51	-3.8	2.6	52	-3.8	2.8	0.01 [-0.37, 0.40]	4.25
Bossen et al. 2013	85	3.5	4.7	79	4.2	4.8	-0.15 [-0.45, 0.16]	6.06
Strom et al. 2013	24	12.9	6.4	24	13.7	5.3	-0.13 [-0.69, 0.42]	2.23
Tsai et al. 2017	19	4.0	4.0	17	6.0	3.0	-0.55 [-1.20, 0.10]	1.67
Lambert et al. 2018	25	7.1	3.8	25	10.9	3.7	-1.00 [-1.58, -0.42]	2.07
Peng et al. 2018	42	6.5	6.0	41	6.8	4.7	-0.05 [-0.48, 0.37]	3.56
Horton et al. 2018 + 2021	81	7.2	4.0	86	6.7	3.8	0.10 [-0.20, 0.41]	6.15
Jiwani et al. 2020	11	51.7	11.7	6	48.1	8.5	0.32 [-0.63, 1.27]	0.82
Cerdan-De-las-heras et al. 2022	27	1.8	2.4	27	4.9	6.8	-0.59 [-1.13, -0.05]	2.38
Heterogeneity: τ^2 = 0.06, I^2 = 54.04%, H^2 = 2.18							-0.20 [-0.43, 0.03]	
Test of $\theta_i = \theta_j$: Q(8) = 16.79, p = 0.03								
Test of θ = 0: z = -1.74, p = 0.08								
**Low**								
Bennell et al. 2018	66	2.2	2.8	65	2.1	2.7	0.04 [-0.30, 0.38]	5.14
Heterogeneity: τ^2 = 0.00, I^2 = .%, H^2 = .							0.04 [-0.30, 0.38]	
Test of $\theta_i = \theta_j$: Q(0) = -0.00, p = .								
Test of θ = 0: z = 0.21, p = 0.84								
**Some concerns**								
Witham et al. 2011	43	0.1	2.3	44	0.0	2.3	0.04 [-0.37, 0.46]	3.70
Furuya et al. 2014	30	5.1	4.4	30	7.6	4.1	-0.58 [-1.09, -0.07]	2.61
O'Brien et al. 2018	37	6.4	6.6	52	7.0	8.2	-0.08 [-0.50, 0.34]	3.68
Snoek et al. 2021	61	3.4	2.8	61	4.0	3.5	-0.19 [-0.54, 0.17]	4.84
Clays et al. 2021	32	-4.5	7.8	22	1.0	3.2	-0.86 [-1.41, -0.30]	2.22
Snoek et al. 2021	89	1.8	3.1	90	2.4	4.0	-0.17 [-0.46, 0.13]	6.46
Wong et al. 2021	39	7.8	3.2	40	8.5	7.8	-0.12 [-0.55, 0.32]	3.41
Yudi et al. 2021	83	8.4	7.2	85	8.2	6.8	0.03 [-0.27, 0.33]	6.18
Young et al. + Drew et al. 2021 + 2022a + 2022b	62	-3.3	3.7	63	-2.1	4.0	-0.31 [-0.66, 0.04]	4.91
Benzo et al. 2022	188	1.1	1.4	187	1.2	1.4	-0.10 [-0.30, 0.10]	10.35
Cox et al. 2022	47	-1.0	4.9	49	-0.5	5.2	-0.10 [-0.49, 0.30]	4.01
Li et al. 2022	50	34.6	12.3	49	35.3	9.2	-0.06 [-0.45, 0.33]	4.12
Pitta et al. 2022	54	3.2	3.1	56	4.5	4.3	-0.32 [-0.70, 0.05]	4.43
Spielmanns et al. 2022	30	4.4	3.5	30	5.3	4.2	-0.23 [-0.73, 0.27]	2.69
Tore et al. 2022	24	4.3	2.6	24	6.9	3.4	-0.86 [-1.44, -0.27]	2.06
Heterogeneity: τ^2 = 0.00, I^2 = 0.00%, H^2 = 1.00							-0.18 [-0.28, -0.09]	
Test of $\theta_i = \theta_j$: Q(14) = 18.63, p = 0.18								
Test of θ = 0: z = -3.80, p = 0.00								
**Overall**							-0.17 [-0.26, -0.09]	
Heterogeneity: τ^2 = 0.01, I^2 = 18.08%, H^2 = 1.22								
Test of $\theta_i = \theta_j$: Q(24) = 36.92, p = 0.04								
Test of θ = 0: z = -3.90, p = 0.00								
Test of group differences: $Q_b(2)$ = 1.55, p = 0.46								

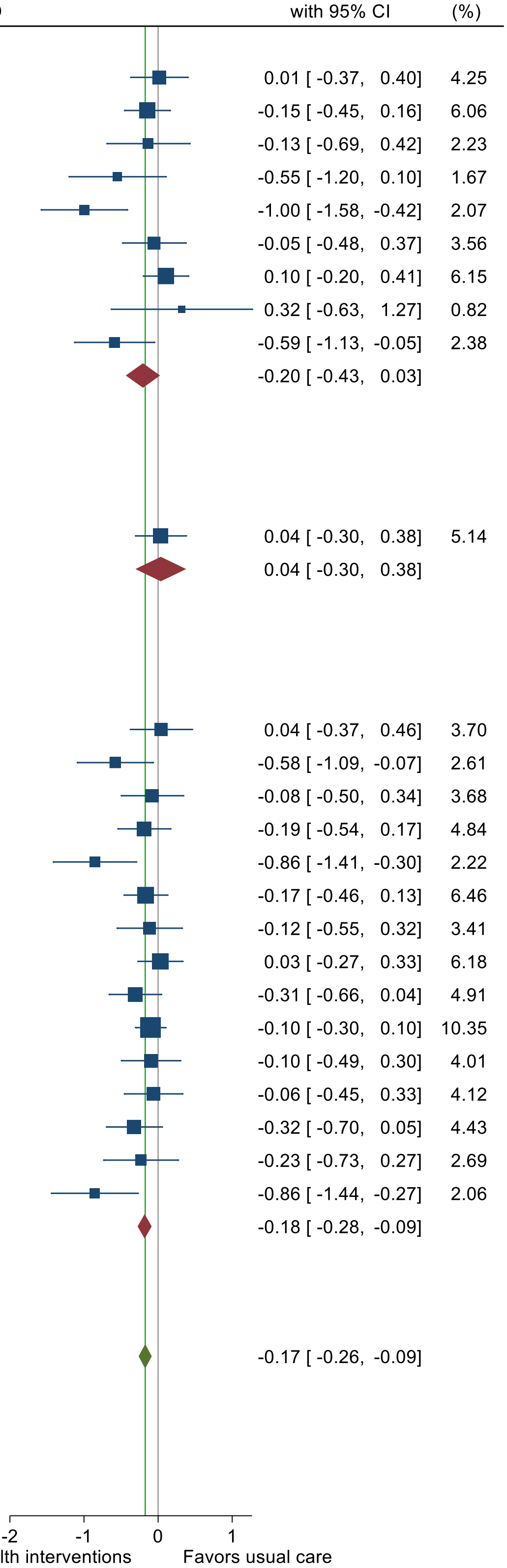

Supplementary Figure 11. Forest plot for health-related quality of life at end-of-intervention sub-grouped by the methodological quality

Study	Treatment N	Treatment Mean	Treatment SD	Control N	Control Mean	Control SD	SMD with 95% CI	Weight (%)
**High**								
Hanssen et al. 2007	97	7.3	7.5	96	5.0	8.1	0.30 [0.02, 0.58]	1.57
Tomita et al. 2009	19	14.2	15.3	13	6.8	22.1	0.39 [-0.30, 1.09]	1.01
Piette et al. 2011	145	38.2	12.0	146	35.8	12.0	0.20 [-0.03, 0.43]	1.63
Reid et al. 2012	115	5.8	0.7	108	5.7	0.8	0.13 [-0.13, 0.39]	1.59
Bossen et al. 2013	85	49.4	36.0	80	47.3	35.8	0.06 [-0.25, 0.36]	1.54
Plotnikoff et al. 2013	74	0.8	0.0	83	0.8	0.0	-1.00 [-1.33, -0.66]	1.51
Strom et al. 2013	24	0.2	2.0	24	0.2	1.5	-0.04 [-0.60, 0.52]	1.19
Plotnikoff et al. 2013	72	0.8	0.1	42	0.8	0.1	0.11 [-0.26, 0.49]	1.44
Holmen et al. 2014	38	49.5	11.0	20	49.0	10.6	0.05 [-0.49, 0.58]	1.22
Holmen et al. 2014	39	51.3	9.1	20	49.0	10.6	0.23 [-0.30, 0.77]	1.23
Moy et al. 2015	144	-42.4	17.6	77	-45.0	16.0	0.15 [-0.12, 0.43]	1.57
Tsai et al. 2017	19	99.0	16.0	17	90.0	18.0	0.52 [-0.13, 1.17]	1.07
Widmer et al. 2017	37	7.2	1.3	34	2.4	1.6	3.27 [2.56, 3.98]	1.00
Duan et al. 2018	44	3.9	0.4	39	3.3	0.6	1.06 [0.61, 1.52]	1.33
Li et al. 2018	30	47.2	18.9	31	42.4	16.8	0.27 [-0.23, 0.76]	1.28
Peng et al. 2018	42	-42.3	53.9	41	-49.6	79.3	0.11 [-0.32, 0.53]	1.38
Osteresch et al. + Wienbergen et al. 2019 + 2021	138	78.2	15.0	143	77.1	14.0	0.08 [-0.16, 0.31]	1.62
Bentley et al. 2020	9	7.6	1.4	6	6.5	2.1	0.59 [-0.41, 1.58]	0.70
Jiwani et al. 2020	11	37.8	4.2	6	39.4	4.2	-0.36 [-1.31, 0.59]	0.74
Pelle et al. 2020	130	0.7	0.2	166	0.7	0.2	0.05 [-0.18, 0.27]	1.63
Benzo et al. 2021	56	0.2	1.0	63	0.0	0.7	0.23 [-0.13, 0.59]	1.47
Robinson et al. 2021	75	-13.1	31.1	78	-15.1	27.9	0.07 [-0.25, 0.39]	1.53
Haejung et al. 2021	12	82.3	15.3	12	78.0	16.2	0.26 [-0.52, 1.04]	0.92
Cerdan-De-las-heras et al. 2022	27	51.9	10.6	27	54.0	16.3	-0.15 [-0.68, 0.37]	1.24
Felker et al. 2022	83	73.4	20.0	82	66.9	23.0	0.30 [-0.01, 0.60]	1.54
Heterogeneity: τ^2 = 0.36, I^2 = 91.14%, H^2 = 11.28							0.25 [-0.00, 0.51]	
Test of $\theta_i = \theta_j$: Q(24) = 146.06, p = 0.00								
Test of θ = 0: z = 1.94, p = 0.05								
**Low**								
Akinci et al. 2018	21	0.8	0.1	22	0.6	0.3	0.84 [0.22, 1.45]	1.12
Bennell et al. 2018	66	0.8	0.1	65	0.8	0.1	0.00 [-0.34, 0.34]	1.49
Nelligan et al. 2021	103	0.8	0.2	103	0.7	0.2	0.33 [0.06, 0.60]	1.58
Heterogeneity: τ^2 = 0.09, I^2 = 70.86%, H^2 = 3.43							0.33 [-0.08, 0.74]	
Test of $\theta_i = \theta_j$: Q(2) = 5.90, p = 0.05								
Test of θ = 0: z = 1.57, p = 0.12								
**Some concerns**								
Dyson et al. 2010	21	64.4	12.2	18	70.3	12.2	-0.47 [-1.10, 0.15]	1.10
Witham et al. 2011	43	0.1	0.2	44	0.0	0.2	0.31 [-0.11, 0.73]	1.39
Hawkes et al. 2013	141	44.8	11.8	153	46.1	10.7	-0.12 [-0.34, 0.11]	1.63
de Sousa Pinto et al. 2014	23	-46.0	12.0	18	-65.6	10.6	1.68 [0.98, 2.39]	1.00
Vasilopoulou et al. 2014	47	-38.4	20.5	50	-50.2	17.7	0.61 [0.21, 1.02]	1.41
Furuya et al. 2014	30	43.3	10.6	30	41.0	11.0	0.21 [-0.29, 0.71]	1.27
Frederix et al. 2015	69	2.5	0.5	70	2.3	0.6	0.37 [0.04, 0.71]	1.50
van der Weegen et al. 2015	65	45.2	9.5	68	47.0	10.0	-0.18 [-0.52, 0.16]	1.50
Cameron-Tucker et al. 2016	35	0.0	6.0	30	0.0	6.0	0.00 [-0.48, 0.48]	1.30
Bennell et al. 2017	70	0.8	0.1	69	0.7	0.1	0.99 [0.64, 1.35]	1.48
Wan et al. 2017	57	-0.9	9.9	52	-0.7	12.7	-0.02 [-0.39, 0.35]	1.45
Bernocchi et al. 2018	48	10.5	12.7	44	0.4	14.5	0.73 [0.31, 1.15]	1.39
O'Brien et al. 2018	37	29.4	9.4	49	33.4	8.9	-0.43 [-0.86, -0.01]	1.37
Avila et al. 2018 + 2019	28	81.3	13.1	26	71.8	13.3	0.71 [0.17, 1.25]	1.21
Wootton et al. 2018 + 2019	43	40.0	12.0	37	42.0	16.0	-0.14 [-0.58, 0.29]	1.36
Bartholdy et al. 2019	19	-3.4	15.5	19	-5.0	15.5	0.10 [-0.52, 0.72]	1.10
Gingele et al. 2019	179	0.7	0.3	173	0.6	0.3	0.07 [-0.14, 0.28]	1.65
Hinman et al. 2019	83	0.7	0.2	82	0.7	0.2	0.00 [-0.30, 0.30]	1.54
Piotrowicz et al. 2019	417	91.2	12.8	416	88.9	14.4	0.17 [0.03, 0.30]	1.71
Vinitha et al. 2019	126	1.0	0.0	122	1.0	0.0	0.00 [-0.25, 0.25]	1.61
Turan Kavradim 2020	33	-16.3	16.3	31	-40.1	23.2	1.18 [0.65, 1.70]	1.24
Bennell et al. 2020	56	0.8	0.1	54	0.8	0.1	-0.07 [-0.44, 0.31]	1.45
Doiron-Cadrin et al. 2020	12	35.6	6.2	11	36.2	6.8	-0.09 [-0.88, 0.70]	0.90
Jiménez-Reguera et al. 2020	17	0.5	0.2	19	0.5	0.2	0.00 [-0.64, 0.64]	1.08
Li et al. 2020	24	48.7	17.5	24	46.9	13.6	0.11 [-0.44, 0.67]	1.19
Galdiz et al. 2021	41	46.1	24.0	40	51.1	24.6	-0.20 [-0.64, 0.23]	1.37
Gohir et al. 2021	48	1.2	11.1	57	1.6	10.3	-0.04 [-0.42, 0.34]	1.44
Snoek et al. 2021	61	5.9	0.8	61	5.9	0.8	0.00 [-0.35, 0.35]	1.48
Waller et al. 2021	197	42.0	22.4	198	43.0	23.1	-0.04 [-0.24, 0.15]	1.66
Wang et al. 2021	39	-18.3	3.5	39	-20.9	4.4	0.65 [0.20, 1.10]	1.34
Andrade et al. 2021	11	28.0	33.0	12	22.0	13.0	0.23 [-0.56, 1.03]	0.90
Clays et al. 2021	34	-1.0	14.4	22	1.7	13.8	-0.19 [-0.72, 0.34]	1.23
Radhakrishnan et al. 2021	19	8.2	13.5	19	13.0	19.3	-0.28 [-0.91, 0.34]	1.10
Reid et al. 2021	68	77.7	14.5	67	78.9	10.3	-0.09 [-0.43, 0.24]	1.50
Reid et al. 2021	158	79.9	12.1	156	80.2	11.7	-0.03 [-0.25, 0.20]	1.64
Snoek et al. 2021	89	50.2	7.2	90	48.3	7.5	0.26 [-0.04, 0.55]	1.55
Wong et al. 2021	39	0.7	0.1	40	0.7	0.1	0.00 [-0.44, 0.44]	1.36
Yudi et al. 2021	83	77.0	14.8	85	79.8	16.1	-0.18 [-0.48, 0.12]	1.54
Benzo et al. 2022	188	4.3	1.1	187	3.8	1.2	0.46 [0.26, 0.67]	1.65
Chan et al. 2022	70	15.4	1.9	69	15.2	2.0	0.10 [-0.23, 0.43]	1.51
Cox et al. 2022	47	2.3	13.6	49	0.2	6.4	0.20 [-0.20, 0.59]	1.42
Indraratna et al. 2022	76	78.2	15.5	74	75.7	14.5	0.17 [-0.15, 0.48]	1.52
Li et al. 2022	50	16.3	7.9	49	17.1	7.8	-0.10 [-0.49, 0.29]	1.43
Nagatomi et al. 2022	15	70.3	27.7	15	78.0	23.0	-0.29 [-0.99, 0.41]	1.01
Spielmanns et al. 2022	30	15.1	8.6	30	19.7	6.4	-0.60 [-1.11, -0.09]	1.26
Tore et al. 2022	24	69.4	18.9	24	45.5	24.4	1.08 [0.48, 1.68]	1.14
Heterogeneity: τ^2 = 0.11, I^2 = 77.61%, H^2 = 4.47							0.14 [0.02, 0.25]	
Test of $\theta_i = \theta_j$: Q(45) = 155.97, p = 0.00								
Test of θ = 0: z = 2.39, p = 0.02								
**Overall**							0.18 [0.07, 0.29]	
Heterogeneity: τ^2 = 0.17, I^2 = 84.38%, H^2 = 6.40								
Test of $\theta_i = \theta_j$: Q(73) = 310.10, p = 0.00								
Test of θ = 0: z = 3.29, p = 0.00								
Test of group differences: $Q_b(2)$ = 1.28, p = 0.53								

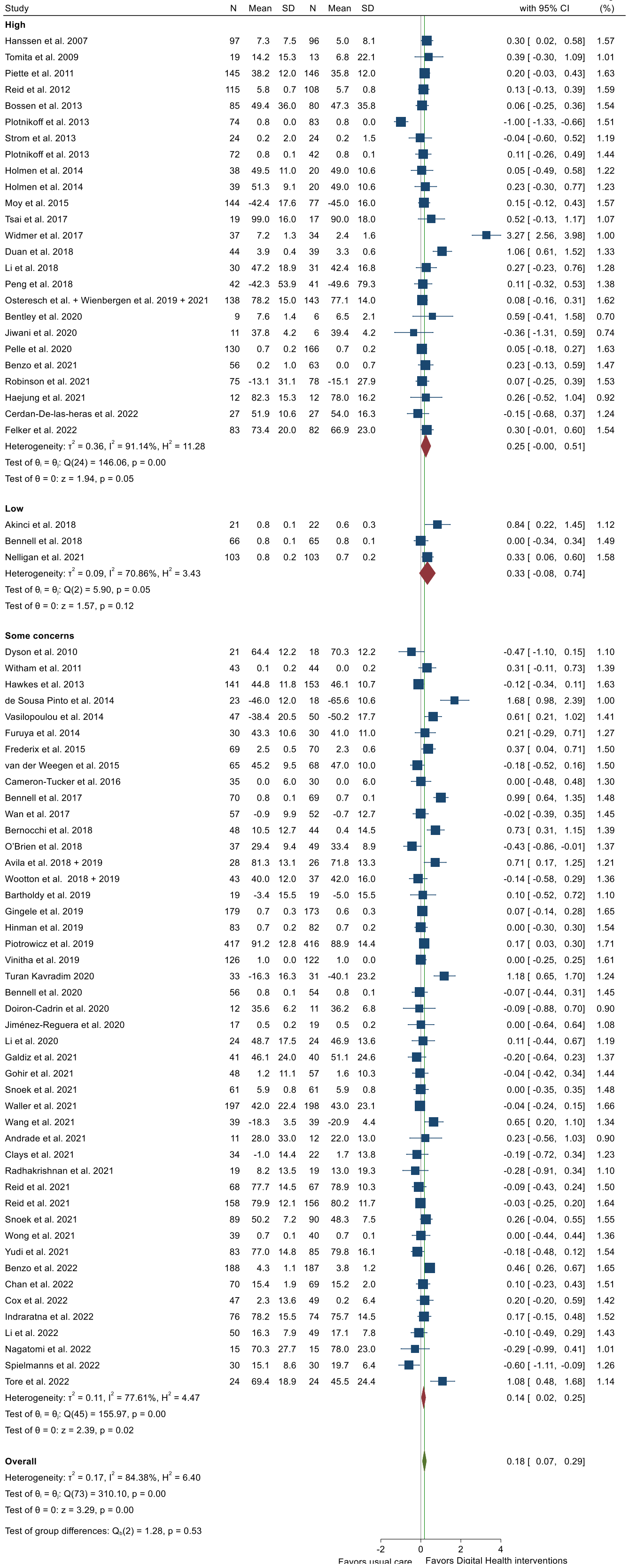

Supplement: Multimedia Appendix 8 [file jmir_v25i1e46439_app8.pdf]
